# Weekends-off efavirenz-based antiretroviral therapy in HIV-infected children, adolescents and young adults (BREATHER): Extended follow-up results of a randomised, open-label, non-inferiority trial

**DOI:** 10.1371/journal.pone.0196239

**Published:** 2018-04-23

**Authors:** Anna Turkova, Cecilia L. Moore, Karina Butler, Alexandra Compagnucci, Yacine Saïdi, Victor Musiime, Annet Nanduudu, Elizabeth Kaudha, Tim R. Cressey, Suwalai Chalermpantmetagul, Karen Scott, Lynda Harper, Samuel Montero, Yoann Riault, Torsak Bunupuradah, Alla Volokha, Patricia M. Flynn, Rosa Bologna, Jose T. Ramos Amador, Steven B. Welch, Eleni Nastouli, Nigel Klein, Carlo Giaquinto, Deborah Ford, Abdel Babiker, Diana M. Gibb, on behalf of the BREATHER (PENTA 16) trial Group

**Affiliations:** 1 MRC CTU, University College London Institute of Clinical Trials and Methodology, London, United Kingdom; 2 Paediatric infectious Diseases Department, Great Ormond Street Hospital, London United Kingdom; 3 Paediatric Department, Our Lady's Children's Hospital, Crumlin, Ireland; 4 Clinical Trials and Infectious Diseases, INSERM/ANRS SC10-US19, Villejuif, France; 5 Research Department, Joint Clinical Research Center, Kampala, Uganda; 6 Paediatric Department, Makerere University, Kampala, Uganda; 7 PHPT-IRD UMI 174, Faculty of Associated Medical Sciences, Department of Medical Technology, Chiang Mai University, Chiang Mai, Thailand; 8 Department of Immunology & Infectious Diseases, Harvard T.H. Chan School of Public Health, Boston, Massachusetts, United States of America; 9 Department of Molecular & Clinical Pharmacology, University of Liverpool, Liverpool, United Kingdom; 10 The HIV Netherlands Australia Thailand Research Collaboration, The Thai Red Cross AIDS Research Center, Bangkok, Thailand; 11 Shupyk National Medical Academy of Postgraduate Education, Kyiv, Ukraine; 12 Kyiv City Centre for Prevention and Control of AIDS, Kyiv, Ukraine; 13 Department of Infectious Diseases, Saint Jude Children's Research Hospital, Memphis, United States; 14 Epidemiology and Infectious Diseases Department, Hospital de Pediatría Dr JP Garrahan, Buenos Aires, Argentina; 15 Department of Paediatrics, Hospital Clinico Universitario San Carlos, Madrid, Spain; 16 Department of Paediatrics, Birmingham Heartlands Hospital, Birmingham, United Kingdom; 17 Department of Virology, University College London Hospitals NHS Foundation Trust, London, United Kingdom; 18 Infection, Immunity and Inflammation Programme, University College London Institute of Child Health, London, United Kingdom; 19 Department of Paediatrics, University of Padova, Padova, Italy; Azienda Ospedaliera Universitaria di Perugia, ITALY

## Abstract

**Background:**

Weekends off antiretroviral therapy (ART) may help engage HIV-1-infected young people facing lifelong treatment. BREATHER showed short cycle therapy (SCT; 5 days on, 2 days off ART) was non-inferior to continuous therapy (CT) over 48 weeks. Planned follow-up was extended to 144 weeks, maintaining original randomisation.

**Methods:**

BREATHER was an open-label, non-inferiority trial. Participants aged 8-24yrs with virological suppression on efavirenz-based first-line ART were randomised 1:1, stratified by age and African/non-African sites, to remain on CT or change to SCT. The Kaplan-Meier method was used to estimate the proportion of participants with viral rebound (confirmed VL≥50 copies/mL) under intent-to-treat at 48 weeks (primary outcome), and in extended follow-up at 96, 144, and 192 weeks. SCT participants returned to CT following viral rebound, 3 VL blips or discontinuation of efavirenz.

**Findings:**

Of 199 participants (99 SCT, 100 CT), 97 per arm consented to extended follow-up. Median follow-up was 185.3 weeks (IQR 160.9–216.1). 69 (70%) SCT participants remained on SCT at last follow-up. 105 (53%) were male, baseline median age 14 years (IQR 12–18), median CD4 count 735 cells/μL (IQR 576–968). 16 SCT and 16 CT participants had confirmed VL≥50 copies/mL by the end of extended follow-up (HR 1.00, 95% CI 0.50–2.00). Estimated difference in percentage with viral rebound (SCT minus CT) by week 144 was 1.9% (90% CI -6.6–10.4; p = 0.72) and was similar in a per-protocol analysis. There were no significant differences between arms in proportions of participants with grade 3/4 adverse events (18 SCT vs 16 CT participants; p = 0.71) or ART-related adverse events (10 vs 12; p = 0.82). 20 versus 8 serious adverse events (SAEs) were reported in 16 SCT versus 4 CT participants, respectively (p = 0.005 comparing proportions between groups; incidence rate ratio 2.49, 95%CI 0.71–8.66, p = 0.15). 75% of SAEs (15 SCT, 6 CT) were hospitalisations for a wide range of conditions. 3 SCT and 6 CT participants switched to second-line ART following viral failure (p = 0.50).

**Conclusions:**

Sustainable non-inferiority of virological suppression in young people was shown for SCT versus CT over median 3.6 years. Standard-dose efavirenz-based SCT is a viable option for virologically suppressed HIV-1 infected young people on first-line ART with 3-monthly VL monitoring.

**Trial registration:**

EudraCT 2009-012947-40

ISRCTN 97755073

ClinicalTrials.gov NCT01641016

## Introduction

An estimated 37 million adults and children, including 4 million young people (aged 15–24 years) are living with HIV worldwide [1]. With scale up of antiretroviral therapy (ART) a growing proportion of perinatally-infected children are surviving into adolescence and beyond, contributing to increasing numbers of youth living with HIV and requiring life-long ART [2]. In the current era of universal life-long ART for all people living with HIV, there is an increased interest in ART reduction strategies that could offer lower cumulative long-term toxicity, better engagement with treatment and substantial costs-savings.

Young people face major challenges growing up with HIV. Adolescents and young adults have poorer adherence, lower rates of virological suppression, higher rates of virological failure [3, 4], higher attrition [5, 6] and ultimately higher mortality than older adults [5, 7]. HIV is now the second leading cause of death and the fourth leading cause of disability among adolescents globally [8]. Social marginalisation, treatment fatigue, stigma and the burden of secrecy around the diagnosis are recognised to be critical factors associated with poor health outcomes [7, 9, 10].

There is an urgent need for effective interventions to help young people stay on treatment and engaged in healthcare [11]. A number of youth-focused interventions, including youth-friendly health services, psychosocial interventions and peer support groups, have been used to improve adherence to ART and retention in care, but with variable impact on health outcomes [6, 7]. While it is likely that a multi-pronged approach is required, and no single intervention will work for all young people short cycle therapy (SCT) offers an innovative approach, with a short weekly ‘breather’ of a two-to-three days off treatment each week, aiming to preserve adherence while improving quality of life and maintaining virological suppression.

Adult studies showed promising short-term results of SCT strategies. In the FOTO (Five On Two Off) US trial, 60 virologically suppressed adults on efavirenz (EFV) and tenofovir disoproxil fumarate (TDF)-based ART were randomised to FOTO versus continuous therapy (CT). All participants remained virologically suppressed over 48 weeks follow-up and expressed a strong preference for SCT [12]. In a larger adult Ugandan trial (57 SCT; 56 CT; most on EFV), there were 11 treatment failures in CT versus 6 in SCT [13]. In a single-arm French trial in 100 predominantly white homosexual adult males with virological suppression, on boosted protease inhibitor (PI) (29%) or non-nucleotide reverse transcriptase inhibitor based ART (79%), 3 days off treatment each week was effective with 96% remaining virologically suppressed at 48 weeks [14].

The BREATHER trial was the first randomised controlled trial (RCT) comparing the efficacy and safety of SCT on EFV-based ART with two days a week off therapy versus CT in older children, adolescents and young adults. The 48 week results showed that SCT was non-inferior to CT for maintenance of virological suppression with a similar safety and resistance profile. There were no differences in low-level viraemia, adverse events, advanced HIV stage events, inflammatory markers, HIV-1 total DNA, or resistance mutations. At the end of the main trial (after the last patient completed 48 weeks follow-up), 90% of participants in the SCT group reported that weekend breaks made life easier than daily ART, mainly due to freedom from needing to carry or take medications, thus enabling easier socialising with peers at weekends [15]. In a qualitative sub-study including 43 young people (51% on SCT), young people on SCT expressed a strong preference for taking weekends-off treatment once they had adapted to a new routine. Many also reported improved adherence to ART during the week [16]. A cost effectiveness analysis showed significant total cost savings and non-significant total health benefits with SCT [17]. In spite of the success of this strategy, there were questions over longer-term efficacy on SCT. Here we report the extended follow-up of the BREATHER trial for an additional 96 weeks, with original randomisation maintained to evaluate the durability of the SCT approach.

## Materials and methods

### Study design and participants

BREATHER [BREaks in Adolescent and child THerapy using Efavirenz and two nRtis] (PENTA 16), was a randomised, parallel-group, open-label, non-inferiority trial which has been previously described [15]. Briefly, eligible participants were aged 8–24 years, with a CD4 cell count of ≥350 cells/μL and a suppressed viral load of <50 copies/mL for at least 12 months on first-line EFV-based ART with two or three nucleoside or nucleotide reverse transcriptase inhibitors (NRTIs). Exclusion criteria included pregnancy, concomitant medications for acute illness or grade 3 or higher creatinine or liver transaminase results. Parents or guardians and older participants provided written consent; young children gave assent appropriate for their age and knowledge of HIV status, as per guidelines for each participating country.

Participants were enrolled from 24 clinical centres in Uganda, Europe, Thailand, South and North America and ethics committees at each site approved the protocol. The primary analysis was performed after the last enrolled participant reached 48 weeks follow-up (end of the main trial phase), as specified in the protocol [15]. The original study was extended for an additional 96 weeks after the completion of the main trial (i.e. minimum 144 weeks planned total follow-up), maintaining the original randomisation. Participants were re-consented for extended follow-up; those on SCT who did not consent or consented to routine data collection only were advised to return to CT. After the last participant reached 144 weeks post-randomisation (end of study, 2nd June 2016) participants were seen for their final visit.

The trial was registered with EudraCT (2009-012947-40), ISRCTN (97755073), and ClinicalTrials.gov (NCT01641016).

### Randomisation and masking

At initiation of the main trial participants were randomized (allocation ratio 1:1) to remain on CT or to change to SCT, while continuing on EFV with two or three NRTIs. Randomisation was done according to a computer-generated randomisation list, stratified by age (8–12, 13–17 and 18–24 years) and site (African vs non-African) [15].

### Procedures

Participants randomised to SCT followed a cycle of five consecutive days on ART and two consecutive days off, usually Saturday-Sunday or Friday-Saturday (referred to herein as weekends-off). Study visits were 12-weekly. At each study visit a clinical assessment was done, including documentation of adverse events and change in HIV disease stage. CD4^+^cell counts and HIV-1 RNA viral load were measured locally at each study visit. Participants with viral load of ≥50 copies/mL were called in for a repeat test on a separate sample within one week; those with a second viral load of ≥50 copies/mL were considered to have a confirmed viral load of ≥50 copies/mL. Participants on SCT with confirmed viral load of ≥50 copies/mL or three single non-sequential viral loads of ≥50 copies/mL in a 12 month period recommenced CT on the same ART. Adherence questionnaires were completed by carers and participants every 12 weeks and included questions on whether a prescribed dose had been missed within the last week. Compliance to the treatment strategy (CT or SCT) was also assessed by separate questions on case report forms at each trial visit. In contrast to the main trial phase, haematology and biochemistry tests were no longer mandated 12-weekly in the extended follow-up phase and were undertaken as per routine practice. Stored plasma samples on patients with viral rebound (two consecutive viral load samples of ≥50 copies/mL) were sent for central resistance testing where local real-time resistance results were unavailable. For HIV pol gene sequencing, nucleic acid extraction followed by nested PCR using outer and inner forward and reverse primers was performed as previously described [15]; amplified pol gene products were then sequenced on an ABI platform (Applied Biosystems®) by Sanger sequencing. An Independent Data Monitoring Committee (IDMC) monitored the trial for safety and efficacy, they reviewed full data on seven occasions during the trial, including twice during the extended follow-up period.

### Outcomes

This report presents the efficacy and safety results for the whole study period. Efficacy and safety analyses were intent-to-treat. The primary outcome was the proportion of participants who had viral rebound (confirmed plasma HIV-1 RNA of ≥50 copies/mL) by week 48 and has previously been reported [15]. Secondary endpoints for the extended follow-up included viral rebound at any time throughout follow up, viral suppression (HIV-1 RNA<50 copies/mL) at the 96 and 144 week time points, change from baseline to 96 and 144 weeks in CD4^+^cell counts and CD4 percent, changes in ART regimen, treatment adherence as assessed by patient completed questionnaires, grade 3 or 4 adverse events, ART treatment-modifying adverse events of any grade, new US Centers for Disease Control (CDC) stage B or C diagnoses or death.

### Statistical methods

The proportions of participants who had viral rebound by 48, 96, 144 and 192 weeks were estimated using Kaplan Meier methods with adjustment for stratification factors, with censoring at the end of study date, last follow-up date or 6 weeks post time-point (e.g. 48+6 = 54 weeks) (the upper bound of the respective assessment window), whichever came earlier. The difference in proportions of participants (SCT minus CT (SCT-CT)) who experienced viral rebound was estimated and two-sided 90% confidence intervals of the difference were obtained with bootstrap standard errors (1000 replicates). The pre-specified non-inferiority margin at 48 weeks was 12% [15]. We compared the hazard of viral rebound at any time during follow up between study arms using a Cox proportional hazard model adjusted for stratification factors, censoring at the end of study date or last follow-up date, whichever came earlier; in a sensitivity analysis (not pre-specified in the Statistical Analysis Plan) we also fitted a survival model assuming a Weibull distribution and allowed for interval-censored data (assuming viral rebound occurred between the last suppressed viral load and the first of 2 HIV-1 RNA of ≥50 copies/mL). In a per-protocol analysis, follow-up was censored if a participant had a break in treatment of more than 7 days, discontinued EFV for more than 7 days or changed to CT for reasons other than viral load failure. Change in CD4^+^ cell counts and percent were calculated using normal linear regression, adjusted for baseline level (as a continuous variable) and stratification factors. The incidence rates of adverse events were compared between the two arms as well as the proportions of young people with an event. Categorical variables were compared with Fisher’s exact tests and rates were calculated using Poisson regression (with standard errors adjusted for clustering within patient). Generalised estimating equations (independent correlation structure) were used to compare self-reported adherence across randomised groups over time. Stata version 14.0 (15.1 for interval-censored survival analysis) was used for all analyses (StataCorp 2013, College Station, Tx, USA: StataCorp LP).

## Results

**Fig 1:**
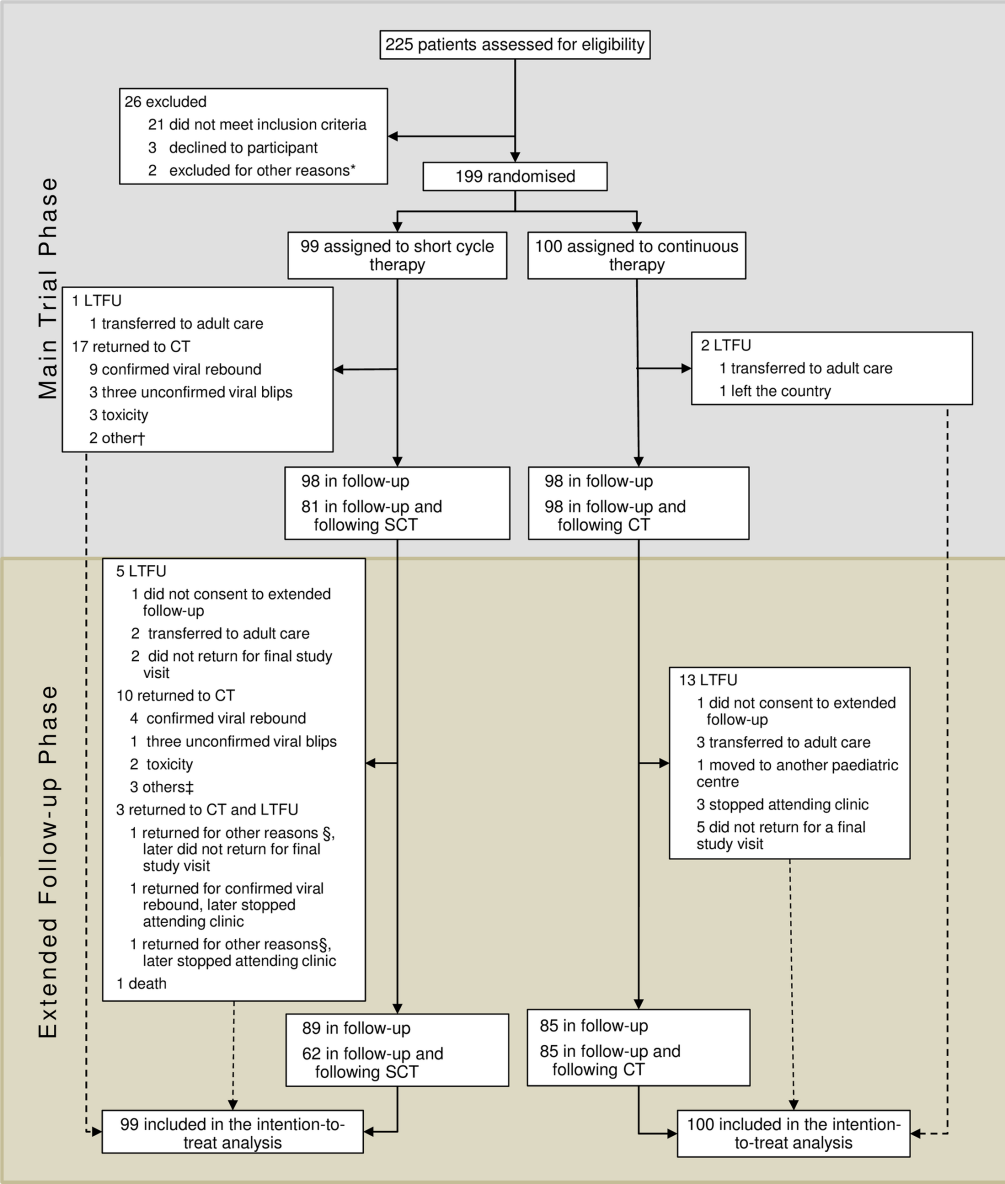
CONSORT diagram. SCT = short cycle therapy, CT = continuous therapy, LTFU = lost to follow-Up. The main trial phase finished when the last enrolled patient reached their 48 week visit, the extended follow-up phase finished when the last enrolled patient reached their 144 week follow-up visit. *One participant was unable to attend the randomisation visit due to a traffic accident and another participant was excluded due to unreliable attendance. †1 returned to CT for poor adherence, 1 changed to eviplera for simplification reasons. ‡2 only consented to routine data collection and were advised to return to CT, 1 changed to eviplera for simplification reasons. §1 only consented to routine data collection and were advised to return to CT, 1 returned to CT for poor adherence. CONSORT checklist is included in the Supporting information (S1 File).

Between April 1, 2011 and June 28, 2013, 225 participants were screened (Fig 1), of whom 199 were randomly assigned (99 to SCT and 100 to CT). 196 participants were followed up to the end of the main trial (31 July 2014), of whom 194 consented to extended follow-up. Among all trial participants median [interquartile range (IQR)] follow-up was 186.1 weeks [162.3–216.1] in the SCT arm and 183.3 weeks [159.8–216.1] in the CT arm; 95 (96%) SCT participants and 92 (92%) CT participants were followed for ≥144 weeks. Median [IQR] number of study visits attended was 17 [15–20]. 89 (90%) SCT participants and 85 (85%) CT attended a final study visit on or after 2 June 2016 (an additional 4 SCT and 5 CT were seen within 12 weeks prior to end of study).

Baseline characteristics of the trial population have been previously reported [15]. Briefly, 35% participants were recruited from Uganda, 24% from Western Europe, 18% from Thailand, 10% from the Ukraine, 7% from the USA and 6% from Argentina. The median age at baseline was 14 years [IQR 12–18], 53% were male, 90% had acquired HIV vertically and the median CD4 count was 735 [IQR 576–968] cells/μL (Table 1). At baseline most participants (85%) had only been exposed to NRTIs and NNRTIs; 15% had also been exposed to PIs, but none had failed first-line ART. Median cumulative ART exposure at baseline was 6.1 years [IQR 1.3–15.5]. Demographics and HIV parameters were well matched between arms, although a lower proportion of young people had CDC stage C disease in the SCT group (13 [13%]) than in the CT group (21 [21%]) (Table 1).

**Table 1:** Baseline characteristics.

	Short cycle therapy	Continuous Therapy	Total
Young people randomised and included	99	100	199
Male	57 (58%)	48 (48%)	105 (53%)
Age (years)	13·7 (11·7–17·7)	14·4 (12·0–17·5)	14·1 (11·9–17·6)
8 to 12	38 (38%)	39 (39%)	77 (39%)
13 to 17	39 (39%)	41 (41%)	80 (40%)
18 to 24	22 (22%)	20 (20%)	42 (21%)
Ethnic origin			
Black (African or other)	58 (59%)	54 (54%)	112 (56%)
White	24 (24%)	17 (17%)	41 (21%)
Asian	15 (15%)	22 (22%)	37 (19%)
Other	2 (2%)	7 (7%)	9 (5%)
Route of infection			
Vertical	90 (91%)	90 (90%)	180 (90%)
Sexual contact	7 (7%)	7 (7%)	14 (7%)
Unknown/other*	2 (2%)	3 (3%)	5 (3%)
CDC stage^§^			
N	16 (16%)	10 (10%)	26 (13%)
A	25 (25%)	25 (25%)	50 (25%)
B	45 (45%)	43 (43%)	88 (44%)
C	13 (13%)	21 (21%)	34 (17%)
Cumulative ART exposure prior to baseline (years)	6.2 (3.8–7.9)	5.9 (4.0–8.4)	6.1 (3.8–8.4)
Baseline regimen is the initial ART regimen	40 (40%)	42 (42%)	82 (41%)
EFV plus:			
Zidovudine, lamivudine†	52 (53%)	53 (53%)	105 (53%)
Tenofovir, lamivudine/ emtricitabine††	25 (25%)	27 (27%)	52 (26%)
Abacavir, lamivudine/ emticitabine†††	22 (22%)	18 (18%)	40 (20%)
Other‡	0 (0%)	2 (2%)	2 (1%)
CD4 percentage	34·5 (29·3–39·0)	34·0 (29·5–38·1)	34·0 (29·5–38·5)
<25%	5 (5%)	6 (6%)	11 (6%)
≥25% to <40%	73 (74%)	76 (76%)	149 (75%)
≥40%	21 (21%)	18 (18%)	39 (20%)
CD4 count (cells/μL)	722·5 (581·0–965·0)	747·3 (575·3–972·8)	735·0 (575·5–967·5)
≥350–500	16 (16%)	12 (12%)	28 (14%)
>500	83 (84%)	88 (88%)	171 (86%)

Data are median (IQR) or n (%).

*Three young people acquired HIV through blood products (1 SCT, 2 CT), two had uncertain mode of transmission (1 CT, 1 SCT).

§One young person (CT) with unknown CDC stage at randomisation.

† Categorized as zidovudine-based.

†† Categorized as tenofovir-based.

††† Categorized as abacavir-based.

‡The remaining NRTI backbones in two patients were 1) zidovudine, lamivudine, tenofovir (categorised as tenofovir-based) and 2) didanosine, abacavir (categorised as abacavir-based).

**Fig 2:**
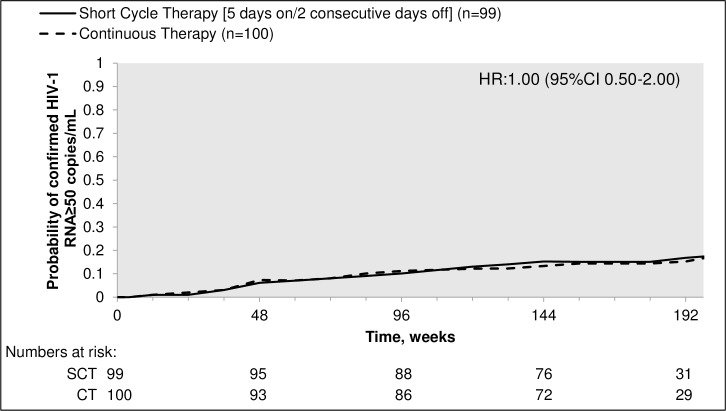
Time to confirmed HIV-1 RNA ≥ 50 copies/mL in the intent-to-treat analysis (adjusted Kaplan-Meier). SCT = short cycle therapy, CT = continuous therapy, HR = adjusted hazard ratio.

**Fig 3:**
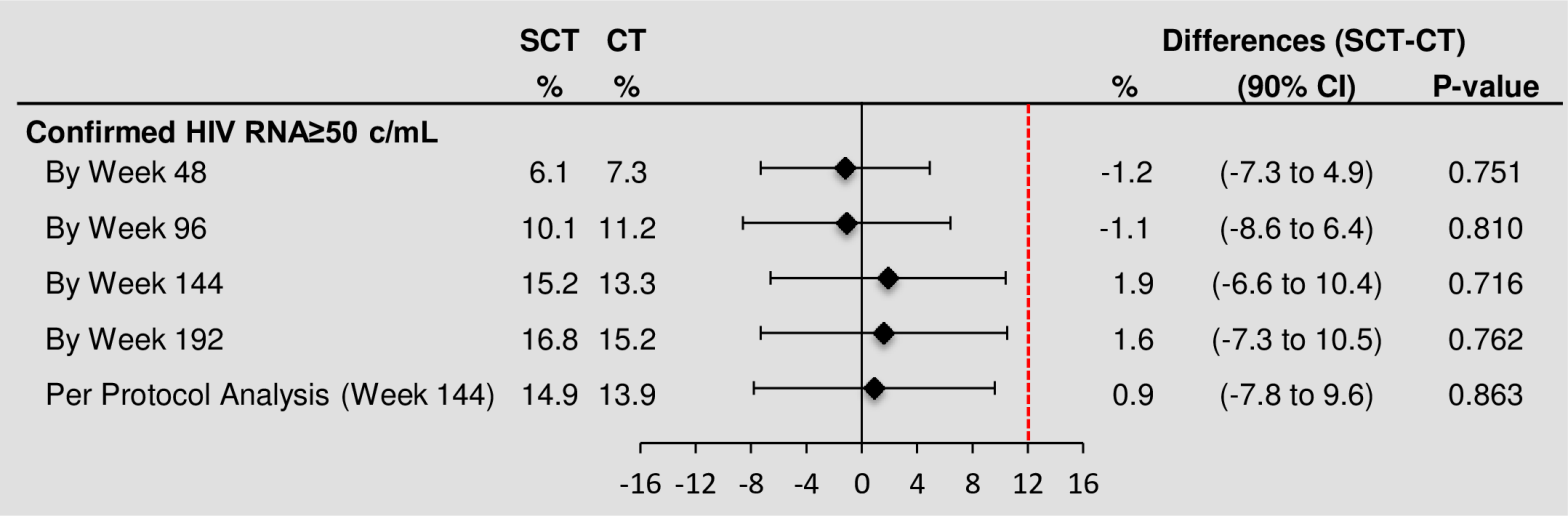
Confirmed HIV-1 RNA ≥ 50 copies/mL by 48, 96, 144 and 192 weeks. SCT = short cycle therapy, CT = continuous therapy.

Thirty-two young people (16 SCT, 16 CT) experienced viral rebound (confirmed HIV-RNA ≥50 copies/mL) by the end of extended follow-up giving rise to a hazard ratio (HR) of 1.00 (95% confidence interval (CI) 0.50–2.00; Fig 2); the hazard ratio under a Weibull model allowing for the interval censoring in the data was similar (HR_W_ = 1.01 (0.50 to 2.02; p = 0.98)). Twenty-eight participants (15 SCT, 13 CT) had confirmed HIV-RNA ≥50 copies/mL by week 144, estimated probability 15% (95% CI 8–23) in the SCT arm versus 13% (95% CI 7–20) in the CT arm, giving an estimated difference (SCT–CT) of 1.9% (90% CI -6.6–10.4, bootstrap test for difference; p = 0.72) (Table 2, Fig 3). Estimated differences were similar at other time points and in the per protocol analysis at week 144 (Fig 3). A pre-specified exploratory analysis examined whether the risk of reaching the primary endpoint was related to type of NRTI considering their intracellular half-life (zidovudine-based vs abacavir-based vs TDF-based, Table 1). A Cox model including randomised arm and baseline NRTI received was fitted; results showed no evidence of an effect of NRTI backbone on the proportions of participants with viral rebound by week 144 after adjusting for treatment strategy (p = 0.46).

**Table 2:** Virology, immunology and secondary endpoints over all follow-up.

	Short cycle therapy	Continuous Therapy	p-value
Young people randomised and included	99	100	
Median weeks from randomisation [IQR] (range)	186.1 [162.3–216.1] (82.0, 264.0)	183.3 [159.8–216.1] (25.0, 262.4)	
**Virology Endpoints**			
Confirmed VL ≥50copies/mL by 144 weeks	15 (15)	13 (13)	0.72
Confirmed VL ≥50copies/mL over all follow-up*	16	16	
During main trial	10	10	
During extended follow up	6	6	
Incidence of confirmed VL≥50 copies/mL over all follow-up per 100 PYs (95% CI)	4.88 (2.99, 7.97)	4.88 (2.99, 7.97)	0.99
Confirmed VL ≥400copies/mL by 144 weeks	9 (9)	8 (8)	0.78
VL <50copies/mL at 96 weeks^×^	77 (92)	76 (93)	1.00
VL <50copies/mL at 144 weeks^×^	71 (93)	71 (90)	0.57
**Other Endpoints**			
**Immunology**			
Mean change in CD4 T+ cell count by 96 weeks, cells/μL	-18.1 (24.3) ‡†	-38.6 (25.0) ‡†	0.56‡
Mean change in CD4% by 96 weeks	-0.1 (0.5) ‡§	-0.6 (0.5) ‡§	0.50‡
Mean change in CD4 T+ cell count by 144 weeks, cells/ μL	16.9 (24.5) ‡†	-19.5 (24.8) ‡†	0.31‡
Mean change in CD4% by 144 weeks	1.0 (0.6) ‡§	0.0 (0.6) ‡§	0.25‡
**Resistance in participants with confirmed VL ≥50copies/ml**			
Number of young people with any major mutations/ Number of young people with available sequences	5/8^∞^	12/13^∞^	
Number without resistance	3	1	
Number with NNRTI mutations only	3	7	
Number with NRTI and NNRTI mutations	2	5	

Data are n (%) or mean change from randomisation (standard error) unless otherwise stated. VL = viral load; NNRTI = non-nucleoside reverse transcriptase inhibitors; NRTI = nucleoside reverse transcriptase inhibitors.

*All follow-up includes all data available until the last randomised patient reached 144 weeks. × Cross-sectional analyses, participants missing viral load at time point are missing outcome.

†79 and 73 young people (YP) in short cycle therapy arm and 75 and 71 YP in continuous therapy arm had CD4 T+ cell count available at baseline and week 96 and baseline and week 144, respectively.

§80 and 75 YP in short cycle therapy arm and 79 and 76 YP in continuous therapy arm had available CD4% at baseline and week 96 and baseline and week 144, respectively.

‡Change in CD4 and CD4% adjusting for baseline CD4/CD4% and stratification factors.

∞6 samples (4SCT, viral loads = 56 copies/mL, 144 copies/mL, 126 copies/mL, 62 copies/mLl and 2CT, viral load = 231 copies/mL, 75 copies/mL) from young people who experienced virological failure in the main trial failed to amplify. 5YP did not have a stored sample available for testing (4 SCT and 1 CT).

Seventeen participants (9 SCT, 8 CT) had confirmed HIV-RNA ≥400 copies/mL by week 144, estimated probability 10% (95% CI 4–15) in the SCT arm versus 8% (95% CI 3–13) in the CT arm [difference (SCT–CT) 1.2% (90% CI -5.8–8.1), bootstrap p = 0.779] (Table 2). In the thirty-two children who had a confirmed HIV RNA ≥50 copies/mL by the end of extended follow-up there were resistance results available on 21 children (8 SCT, 13 CT). 17 participants had major resistance mutations (5 SCT, 12 CT), 10 had resistance to non-nucleoside reverse transcriptase inhibitors (NNRTI) only (3 SCT, 7 CT) and 7 had resistance to both NNRTIs and NRTIs (2 SCT, 5CT) (Table 2). There were no significant differences between groups in change in absolute CD4+ T-cell counts or CD4% at 96 or 144 weeks (Table 2) or at other study weeks (data not shown).

44 (22%) young people changed composition of their ART regimen by the end of extended follow-up (20 SCT, 24 CT). Of these 12 in the SCT arm and 11 in the CT arm changed their third agent; all 12 in the SCT arm returned to CT. An additional, 18 young people in the SCT arm resumed CT and stayed on the same regimen. Reasons for returning to CT were: 14 due to meeting the primary endpoint, 4 due to three viral blips (unconfirmed plasma HIV-1 RNA of ≥50 copies/ml), 5 due to stopping EFV for toxicity and 7 for other reasons (Table 3). 9 (5%) participants switched to second-line therapy (defined as a switch of EFV to another drug class associated with a detectable viral load) during follow-up, 3 from the SCT arm, 6 from the CT arm (Fisher’s exact p = 0.50).

**Table 3:** Treatment changes and actions after viral rebound.

	Short cycle therapy		Continuous Therapy	
Young people assessed for ART after randomisation	99		100	
**ART status at last study visit**				
On initial regimen	79	(80)	76	(76)
*Initial regimen but changed to CT*	*18*	*(18)*	*0*	*(0)*
Changed NRTI backbone only (no change in SCT/CT strategy)	8	(8)	13	(13)
Switched third agent	12	(12)	11	(11)
Switched to second-line*	*3*	*(3)*	*6*	*(6)*
**Return to CT in SCT group**	**30**	**(30)**	-	-
Due to confirmed RNA ≥50 copies/mL	14	(14)	-	-
Due to 3 unconfirmed RNA ≥50 copies/mL	4	(4)	-	-
Due to stopping EFV for toxicity	5	(5)	-	-
Due to other†	7	(7)		
**Treatment changes and suppression after viral rebound**				
**Number of participants with confirmed VL≥50copies/mL**	**16**		**16**	
Re-suppressed after viral rebound‡ (% of participants with confirmed VL≥50copies/mL)	13	(81)	8	(50)
*On the same regimen*	*10*		*6*	
With a switch to second-line*	*3*		*2*	
Did not re-suppress after viral rebound by last follow-up‡	3§	(19)	8	(50)
*On the same regimen as at viral rebound*	*3*		*4*	
With a switch to second-line*	*0*		*4*	

Data are n (%). EFV = efavirenz; SCT = short-cycle therapy; CT = continuous therapy; VL = viral load.

*Switching to second-line was defined as a change in efavirenz for reasons of treatment failure (confirmed viral load ≥ 50 copies per mL).

†Two participants changed to eviplera for simplification reasons (one later virally rebounded), three participants consented to routine data collection only in the extension period and were instructed to return to CT, and 2 were changed to CT due to poor adherence/clinic attendance (one later virally rebounded).

‡All patients changed to CT if following a SCT strategy.

§ Two SCT participants resumed CT prior to viral rebound.

Of the 16 young people randomised to SCT who had a confirmed HIV RNA≥50 copies/mL, 14/16 resumed CT on rebound; of these, 13 re-suppressed: 10 on the same regimen and three with a switch to second-line. Of the three SCT participants who were not suppressed by the last follow-up, two resumed CT prior to rebound (one for non-compliance, another for simplification) (Table 3). Of the 16 young people randomised to CT who had a confirmed HIV RNA≥50 copies/mL, eight re-suppressed: six on the same regimen and two with a switch to second-line.

In 73 (6%) versus 94 (8%) questionnaires completed by young people in the SCT and CT arms respectively, missed doses were reported in the last week (excluding weekend breaks in the SCT arm (p = 0.79)). Reports of missed doses by carers were similar (p = 0.53). Based on ART logs, eight (8%) young people in the SCT arm and 17 (17%) young people in the CT arm had ever had a treatment interruption of three days or more, excluding weekend breaks in SCT arm (p = 0.09) (data not shown).

In the SCT group, 79% of weekend breaks were reported as taken, 99% excluding the time after participants had returned to CT for viral rebound or other reasons. At 3% of visits young people following the SCT strategy reported sometimes delaying restart of ART after the weekend break. Young people in the CT arm reported missing all of their doses for two or more days in a row on at least one occasion at 101 (9%) visits; 3 (3%) CT participants reported missing ART for two or more days in a row at more than half of their clinic visits.

There was one death in the SCT arm due to bacterial sepsis. It was judged by the Endpoint Review Committee to be unrelated to treatment strategy; the patient had returned to CT after 9 weeks of SCT due to viral rebound and re-suppressed on the same regimen, and died after ~4.5 years follow-up. No participants had a new stage C event and four young people had a CDC stage B event (3 in SCT and 1 in CT). By the end of follow-up, 34 participants had experienced 48 grade 3 and 4 events, with no significant difference between arms (Fisher’s exact p = 0.71) (Table 4). Similarly there were no significant differences between arms in the proportion of participants experiencing ART related adverse events (p = 0.82) or treatment modifying adverse events (p = 0.78). More participants experienced serious adverse events (SAEs) in the SCT arm (n = 16) compared to the CT arm (n = 4) (p = 0.005); there were 20 events in SCT versus 8 events in CT (incidence rate ratio 2.49, 95% CI: 0.71–8.66; p = 0.15) (Table 4).

**Table 4:** Adverse events.

	Short cycle therapy		Continuous therapy		Total		P-Value*
Young people randomised and included, N	99		100		199		
**Number of events [Number of YP]**							
Grade 3 and 4 AEs	27	[18]	21	[16]	48	[34]	0.71
ART related AEs	13	[10]	17	[12]	30	[22]	0.82
Treatment modifying AEs	7	[6]	8	[8]	15	[14]	0.78
Serious AEs	20	[16‡]	8	[4]	29	[20]	0.005
New CDC stage B events	3	[3]	1	[1]	4	[4]	0.37
New CDC stage C events	0	[0]	0	[0]	0	[0]	1.00
**Event Rate per 100 PYs**					**Rate ratio†** **(95% CI)**		
Grade 3 and 4 AEs	7.44		5.85		1.28 (0.62, 2.65)		0.50
ART related AEs	3.58		4.73		0.76 (0.32, 1.80)		0.53
Treatment modifying AEs	1.93		2.23		0.87 (0.31, 2.44)		0.79
Serious AEs	5.79		2.23		2.49 (0.71, 8.66)		0.15

YP = young people, PYs = person-years, AEs = adverse events.

*P-values in top half of table compare number of young people experiencing an event between arms;

†Adjusted for stratification factors; incidence rate ratio from Poisson regression model, with CT as the reference category. Fixed-effects model with standard error adjusted for clustering within participant

‡One young person in the short cycle therapy arm died of bacterial sepsis.

A large proportion 21 (75%) of SAEs were hospitalisations (15 SCT, 6 CT); other SAEs included other serious medical conditions (3 SCT, 1 CT), 1 death (SCT), 1 life-threatening event (CT) and 1 foetal malformation (SCT). Of all SAEs, 11 were for infections (8 SCT, 3 CT), 5 for neuropsychiatric disorders (headache, syncope, hemiparesis, intentional self-injury, suicidal ideation) (2 SCT, 3CT), 1 neoplasm (SCT) and 11 other (9 SCT, 2CT) (Table 5). Seven SAEs in 5 participants in the SCT arm occurred more than 24 weeks after the participant had resumed CT (Table 5). The only SAE considered by the Endpoint Review Committee, blind to randomised allocation, to be definitely HIV related was a relapse of Kaposi’s sarcoma in a young person following the SCT strategy. There were a further 13 events (8 in SCT arm, 5 in CT) which were considered to be of uncertain in relationship to HIV.

**Table 5:** Details of serious adverse events.

	Short cycle therapy		Continuous therapy		Total	
Young people randomised and included	99		100		199	
**Number of events [Number of YP]**						
**Hospitalisations**						
**Infections and infestations**	**7**	**[7]**	**2**	**[2]**	**9**	**[9]**
Appendicitis	0	[0]	1	[1]	1	[1]
Cellulitis	0	[0]	1	[1]	1	[1]
Gastroenteritis viral	1	[1]	0	[0]	1	[1]
Infective exacerbation of bronchiectasis	1	[1]	0	[0]	1	[1]
Measles*	1†	[1]	0	[0]	1	[1]
Pharyngeal abscess	1	[1]	0	[0]	1	[1]
Pneumonia bacterial	1	[1]	0	[0]	1	[1]
Pulmonary tuberculosis	1	[1]	0	[0]	1	[1]
Shigella infection	1†	[1]	0	[0]	1	[1]
**Injury, poisoning and procedural complications**	**3**	**[3]**	**0**	**[0]**	**3**	**[3]**
Burns second degree	1†	[1]	0	[0]	1	[1]
Burns second degree	1	[1]	0	[0]	1	[1]
Alcohol poisoning	1	[1]	0	[0]	1	[1]
**Nervous system disorders**	**1**	**[1]**	**1**	**[1]**	**2**	**[2]**
Headache	1	[1]	0	[0]	1	[1]
Syncope	0	[0]	1	[1]	1	[1]
**Respiratory, thoracic and mediastinal disorders**	**2**	**[2]**	**0**	**[0]**	**2**	**[2]**
Epistaxis	1	[1]	0	[0]	1	[1]
Pneumothorax	1	[1]	0	[0]	1	[1]
**Neoplasms benign, malignant and unspecified (incl cysts and polyps)**	**1**	**[1]**	**0**	**[0]**	**1**	**[1]**
Kaposi's sarcoma AIDS related	1	[1]	0	[0]	1	[1]
**Pregnancy, puerperium and perinatal conditions**	**0**	**[0]**	**1**	**[1]**	**1**	**[1]**
Abortion spontaneous	0	[0]	1	[1]	1	[1]
**Psychiatric disorders**	**0**	**[0]**	**1**	**[1]**	**1**	**[1]**
Intentional self-injury	0	[0]	1	[1]	1	[1]
**Reproductive system and breast disorders**	**1**	**[1]**	**0**	**[0]**	**1**	**[1]**
Testicular torsion	1†	[1]	0	[0]	1	[1]
**Surgical and medical procedures**	**0**	**[0]**	**1**	**[1]**	**1**	**[1]**
Contusion of chest	0	[0]	1	[1]	1	[1]
**Total**	**15**	**[13]**	**6**	**[4]**	**21**	**[17]**
**Other medical conditions**						
**Infections and infestations**	**0**	**[0]**	**1**	**[1]**	**1**	**[1]**
Neurosyphilis	0	[0]	1	[1]	1	[1]
**Injury, poisoning and procedural complications**	**1**	**[1]**	**0**	**[0]**	**1**	**[1]**
Toxicity to various agents	1†	[1]	0	[0]	1	[1]
**Nervous system disorders**	**1**	**[1]**	**0**	**[0]**	**1**	**[1]**
Hemiparesis	1	[1]	0	[0]	1	[1]
**Pregnancy, puerperium and perinatal conditions**	**1**	**[1]**	**0**	**[0]**	**1**	**[1]**
Abortion spontaneous	1	[1]	0	[0]	1	[1]
**Total**	3	[3]	1	[1]	4	[4]
**Congenital anomaly or birth defect**						
**Congenital, familial and genetic disorders**	**1**	**[1]**	**0**	**[0]**	**1**	**[1]**
Foetal malformation	1†	[1]	0	[0]	1	[1]
**Fatal**						
**Infections and infestations**	**1**	**[1]**	**0**	**[0]**	**1**	**[1]**
Bacterial sepsis	1†	[1]	0	[0]	1	[1]
**Life-threatening**						
**Psychiatric disorders**	**0**	**[0]**	**1**	**[1]**	**1**	**[1]**
Suicidal ideation	0	[0]	1	[1]	1	[1]
**TOTAL**	**20**	**[16]**	**8**	**[8]**	**28**	**[20]**

YP = young people.

* Young person had measles but a series of related events lead to the hospitalisation. Related events were acute gastroenteritis and probable laryngotracheobronchitis.

† Event occurred more than 24 weeks after the participant had resumed CT

## Discussion

BREATHER was the first trial to demonstrate non-inferiority of SCT in maintaining virological suppression in children, adolescents and young people on standard dose EFV-based ART compared to CT [15]. The extended follow-up with maintained randomisation has confirmed sustainability of the strategy over a median 3.6 years of follow-up. Approximately two-thirds of participants remained on SCT at the end of follow-up and, and of those who switched to CT, nearly 40% did so for reasons other than viral rebound or blips. Importantly, two-thirds (63%) of participants experiencing viral rebound on SCT re-supressed on the same regimen on returning to CT (compared to 38% in CT who re-supressed on the same regimen following viral rebound), and only 3% of SCT participants needed to switch to second-line ART (versus 6% in CT), providing reassurance that the strategy is not compromising durability of first-line ART.

Although exploratory, there was no association between type of baseline NRTIs and virological suppression, suggesting there is a flexibility to choose an age-appropriate NRTI backbone. This can be particularly important for the paediatric population which still faces limited NRTI options, as TDF is not recommended for children and younger teenagers due to bone toxicity concerns, and a safer option tenofovir alafenamide is not yet widely available.

The SCT strategy showed high acceptability by adolescents and young people over a median 1.6 years follow-up [15, 16]. Although initially some participants reported difficulties in adapting to a new routine, most adapted after a relatively short period and preferred SCT over CT [16]. Acceptability was not re-measured during extended follow-up but sites reported that most participants preferred to continue SCT; at the extension of the study only 3 SCT participants chose not to continue with full trial follow-up visits and were instructed to return to CT.

Adherence questionnaires showed similar adherence to prescribed treatment in both trial groups; pre-trial concerns that the message allowing participants to miss a couple days of treatment might compromise adherence appeared unfounded. Notably, young people on SCT participating in the qualitative substudy reported better adherence during the week, and for many the weekend off treatment served as a reminder and a reward [16]. BREATHER results challenge the concept that continuous daily ART is required to maintain virological suppression and demonstrate that five out of seven days of standard dose EFV-based treatment is sufficient.

We found less ART-related adverse events on SCT than CT in the first 48 weeks [15], although the difference was not maintained during extended follow-up. The trial was not powered to show subtle differences in toxicity profile however with an average of 29% less treatment received per week in patients following the SCT strategy there is the potential for lower toxicity [18]. Interestingly, SCT participants in the qualitative sub-study reported relief of perceived side effects at weekends [16]. Future studies in this area will need to include qualitative sub-studies and employ questionnaires targeting tolerability as the qualitative sub-study participants from both arms reported more side effects than they disclosed to health care professionals at clinic visits.

More participants in the SCT arm experienced at least one SAE, mostly due to hospitalisations for a wide range of conditions. Previous adult trials comparing SCT with CT did not show differences in SAEs [12, 13, 19]. As we observed no difference in viral load rebound, CD4 count values, CDC B or C events or severe (grade 4) and potentially life-threatening (grade 3) AEs, a possible explanation is that the lack of blinding led to an increased tendency to admit SCT participants to hospital. The finding that around one third of SAEs in the SCT arm occurred in participants who had resumed continuous treatment at least 24 weeks previously and more than half the SAEs were judged to be unrelated to HIV by the Endpoint Review Committee, who were blind to randomisation, lends support to this explanation for the increased SAEs.

Daily treatment with triple-drug therapy is the accepted gold standard for HIV treatment but treatment fatigue and long-term toxicity can be challenging for many individuals on daily ART and increasing costs with the recently introduced universal life-long access to ART pose difficulties for national programmes in many middle- and low-income countries. There is a growing interest in reducing ART exposure through dual therapy regimens or short cycle therapy with both approaches aiming to maintain effective long term adherence and viral suppression while reducing treatment costs and potential toxicities [19–21]. BREATHER took place in 11 countries in geographically and economically diverse settings, offering the potential for wide generalisability of the results to settings where 12-weekly viral load monitoring is available. We tested the SCT strategy in an age group where the many psychosocial challenges of growing-up and maturing with HIV make adherence to therapy particularly challenging and where new strategies for delivering sustainable ART are most needed. However, the findings can be generalised to the older adult population, some of whom may also benefit from weekends off treatment. A cost-effectiveness analysis to 48 weeks found significant ART cost savings with SCT in settings using generic and branded antiretrovirals and no differences in costs of inpatient or outpatient care or laboratory tests between the arms [17]. If widely accepted, the SCT strategy could offer substantial cost-savings for national programmes.

The trial design and context does preclude generalisability to settings with infrequent viral load monitoring or to other ART regimens. We used 12-weekly “real-time” viral load monitoring, providing opportunity for clinic recall following an increased viral load and for a timely switch to CT with confirmed viral load rebound. We used standard dose EFV-based regimens, hypothesising that, due to a long half-life, EFV would provide sufficient drug pressure during the short break off ART. Although integrase inhibitors and lower dose EFV are likely to gain an increasing share of the third agent market over time, standard dose EFV is forecast to have a 33% share of the market in low- and middle-income countries in 2020 [22]. Children and adults who are stable on EFV-based ART are unlikely to be switched to other regimens. In addition, delays in access to integrase inhibitors for children mean that EFV-based ART will remain the most common first-line ART for children for the foreseeable future. Further pragmatic RCTs are required to evaluate SCT in countries where viral load testing is less frequent and other ART regimens are in use. Interestingly, a single arm French adult trial suggested excellent virological suppression with only four days of treatment each week and with various ART regimens, including PI-based ART; the validity of this 4-day on, 3-day off SCT strategy will be further evaluated in a RCT [23].

In conclusion, our findings, taken together with other available evidence, suggest that standard dose EFV-based SCT with 2 days a week off treatment is a viable alternative option for adherent HIV-1 infected young people who are virologically supressed on first-line ART. The strategy should be considered for implementation in settings with 3-monthly viral load monitoring. Potential benefits of the strategy are alleviated treatment fatigue, improved life style, reduced cumulative toxicity of antiretrovirals over a life-time and costs saved. Future research is required to evaluate the generalisability of this maintenance treatment approach to other ART regimens and to settings with less frequent viral monitoring.

## Supporting information

S1 File CONSORT checklist. (DOCX)

S2 File BREATHER study contributors. (DOCX)

S3 File BREATHER protocol. (PDF)

S1 Table Centres participating in the BREATHER trial. (DOCX)
