# Supplementary material for: Weekends-off efavirenz-based antiretroviral therapy in HIV-infected children, adolescents and young adults (BREATHER): Extended follow-up results of a randomised, open-label, non-inferiority trial
Source: PLoS One. 2018 Apr 23;13(4):e0196239. doi: 10.1371/journal.pone.0196239 (PMC5912750; doi:10.1371/journal.pone.0196239)
Supplement: S2 File — (DOCX) [file pone.0196239.s002.docx]

**Study Contributors**

**BREATHER Trial Management Group:** J Ananworanich, A Babiker, S Bernays, T Bunupuradah, K Butler (chair), A Caelho, J Calvert, S Chalermpantmetagul, K Chokephaibulkit, R Choudhury, A Compagnucci, TR Cressey, C Giaquinto, D Ford, DM Gibb, L Harper, J Inshaw, E Kaudha, J Kenny, H Kizito, N Klein, E Menson, S Montero, CL Moore, V Musiime, A Nanduudu, E Nastouli, M Ndiaye, A Nunn, C O’Leary, L Picault, JT Ramos Amador, T Rhodes, Y Riault, Y Saϊdi, K Scott, J Seeley, S Storey, A Turkova.

**BREATHER Steering Committee:** I Weller (chair), JT Ramos, J Ananworanich, K Butler, P Clayden, J Darbyshire, V Leroy, V Musiime, DM Gibb.

**PENTA Steering Committee:** J-P Aboulker, J Ananworanich, A Babiker, E Belfrage, S Bernardi, R Bologna, D Burger, K Butler, G Castelli-Gattinara , P Clayden, A Compagnucci, TR Cressey, R de Groot, M Della Negra, A De Rossi, A Di Biagio, D Duiculescu (deceased), A Faye, V Giacomet, C Giaquinto (chairperson), DM Gibb, I Grosch-Wörner, M Hainault, L Harper, N Klein, M Lallemant, H Lyall, M Mardarescu, L Marques, MJ Mellado Peña, M Marczynska, D Nadal, E Nastouli, L Naver, T Niehues, D Pillay, J Popieska , JT Ramos Amador, P Rojo Conejo, L Rosado, V Rosenfeldt (deceased), C Rudin, Y Saïdi, M Sharland, HJ Scherpbier, C Thorne, G Tudor-Williams, A Turkova, N Valerius, A Volokha, AS Walker, S Welch.

**Independent Data Monitoring Committee:** A Pozniak (Chair), S Vella, G Chène, T Vesikari.

**Trials Units:**

*MRC Clinical Trials Unit, UK:* A Babiker, J Calvert, R Choudhury, D Ford, DM Gibb, L Harper, J Inshaw, D Johnson, J Kenny, S Martins, S Montero, CL Moore, A Nunn, C O’Leary, K Scott, S Shidfar, A South, J Thompson, S Townsend, A Turkova.

*INSERM SC10-US19, France:* JP Aboulker, A Arulananthan, A Coelho, A Compagnucci, S Léonardo, L Meyer, M Ndiaye, L Picault, Y Riault, Y Saïdi.

*PHPT, Thailand:* S Chailert, S Chalermpantmetagul, TR Cressey, G Jourdain, A Kaewbundi S Le Coeur, N Ngo-Giang-Huong R Peongjakta, K Seubmongkolchai, W Sripaoraya P Sukrakanchana, K Than-in-at, S Thammajitsagul.

**Immunology and Virology Advisory Group:** A De Rossi, N Klein, MA Muñoz Fernandez, E Nastouli, N Ngo, D Pillay.

**Qualitative Substudy Group:** S Bernays, S Paparini, T Rhodes, J Seeley.

**Endpoint Review Committee:** K Butler, DM Gibb, V Musiime.

**Communications:** Magda Conway, CHIVA (Children’s HIV Association).

**Recruiting Sites:**

**Argentina**: Hospital Dr JP Garrahan / Helios Salud, Buenos Aires: R Bologna, J Da Bouza, D Mecikovsky, G Sotera, A Mangano, M Moragas, E Vicentini.

**Belgium**: St. Pierre University Hospital, Brussels: M Hainaut, E Van Der Kelen, S Vandenwijngaert.

**Denmark**: Hvidovre Hospital: V Rosenfeldt, N Valerius, L Jensen.

**Germany**: J W Goethe University Frankfurt: C Koenigs, S Schultze-Strasser, R Linde, K Mantzsch

**Ireland**: Our Lady’s Children’s Hospital, Dublin: K Butler, P Gavin, R Leahy, A Rochford, M Goode, A Walsh, E Hyland, M O’Connor.

**Spain:** I Garcia Mellado Hospital 12 de Octubre, Madrid: P Rojo, D Blázquez, C Epalza, M Fernández, M I Gonzalez Tomé; Hospital Sant Joan De Déu, Barcelona: C Fortuny Guasch, A Noguera Julian, C Estepa Guillén, P Santin Riba, A Murciano Cabeza; Hospital La Fe, Valencia: M D Perez Tamarit, M C Otero Reigada, F Castera Brugada, I Segarra Granell, R Amigo Moreno; Hospital La Paz, Madrid: M J Mellado Peña, M Garcia Lopez Hortelano, M I De José Gomez, L Escosa, T Sáinz Costa; Universitario de Getafe: S Guillen Martin, L M Prieto Tato; Hospital Clínico San Carlos, Madrid: JT Ramos; Biobanco Gregorio Marañon, Madrid: M A Muñoz Fernandez, J L Jimenez Fuentes, C Gómez Rico, A Garcia Torre.

**Thailand:** The HIV Netherlands Australia Thailand Research Collaboration (HIV-NAT): J Ananworanich, T Bunupuradah, S Chanthaburanun, N Kasipong, T Noppakaorattanamanee, T Pitimahajanaka, T Puthanakit, N Thammajaruk,; (PHPT): Kalasin Hospital: D Dornngern, N Kunchanarong, L Mongkun, S Srirojana; Regional Health Promotion Center Region 6, Khon Kaen: K Aue-apisak, S Hanpinitsak, P Kangsavon, K Narksan, M Shevasateanchai, S Tongpua.

**UK**: Great Ormond Street Hospital, London: N Klein, J Kenny, A Turkova, D Shingadia, J Flynn, M Clapson, K Parkes, E Howley, L Spencer-Walsh; Evelina Children's Hospital, London: E Menson, R Cross, C Duncan, V Timms, E Reus, A Callaghan, S Tomlin, E Jones; Institute of Child Health, London: H Poulsom, N Klein, L Carter; Royal Infirmary, Bristol: J Bernatoniene, A Finn, E Clarke, F Manyika, L Hutchison, H Smee, L Ball, K Stevenson; Heartlands Hospital, Birmingham: S Welch, S Hackett, G Gilleran, J Daglish, L Horton, K Gandhi; Queen's Medical Centre, Nottingham: A Smyth, J Smith, A Short, L Fear, S Stafford, S Hodgson, Y Taha; Leicester Royal Infirmary: S Bandi, J Philps, J Bwire, J Gardener; St. George’s Hospital London: K Doerholt, K Prime, M Sharland, S Donaghy, L Spencer-Walsh, S Storey, O Okolo, D Rolfe; London School of Hygiene and Tropical Medicine: S Bernays, T Rhodes, J Seeley. University College London Hospital (Department of Virology): E Nastouli, S Kirk, P Grant, B Ferns, J Garson.

**Uganda**: Joint Clinical Research Centre, Kampala: C Kityo, V Musiime, H Kizito, A Nanduudu, A Drasiku, E Kaudha, S Senyonjo, I Obella, M Odera, P Oronon, H Nakyambadde, P Kyobutungi, O Senfuma, D Eram, J Nkalubo, L Nakiire, M Nabalaama, I Ssewanyana, G Pimundu, P Segonga, B Nakalawa, L Mugarura, A Kwaga, J Kasozi, M Ojok, J Namusanje; MRC/UVRI Uganda Research Unit on AIDS, Entebbe: M Ndagire, S Namukwaya.

**Ukraine**: City AIDS Center, Kiev: A Volokha, I Raus, O Mostovenko, N Chentsova.

**USA:** St. Jude’s Children's Research Hospital, Memphis: P Flynn, R Dallas, T Wride, J Utech, S Ost, A Gaur, K Knapp, N Patel, M Shenep, T Culley, M Griffith, S Carr, C Longserre.
