# Supplementary material for: Weekends-off efavirenz-based antiretroviral therapy in HIV-infected children, adolescents and young adults (BREATHER): Extended follow-up results of a randomised, open-label, non-inferiority trial
Source: PLoS One. 2018 Apr 23;13(4):e0196239. doi: 10.1371/journal.pone.0196239 (PMC5912750; doi:10.1371/journal.pone.0196239)
Supplement: S3 File — (PDF) [file pone.0196239.s003.pdf]

Paediatric European Network for Treatment of AIDS

BREATHER (PENTA 16)

Short-Cycle Therapy (SCT) (5 days on/2 days off) in young people with chronic HIV-infection

ISRCTN97755073
EUDRACT: 2009-012947-40
CTA: 27505/0005/001-0001

Protocol Version 1.10

Protocol date 29th May 2015

Authorised by:

Name:	Ian Weller	Role:	Chair of BREATHER Steering Committee
Signature:		Date:	28th May 2015
Name:	Carlo Giaquinto	Role:	Chair of PENTA Steering Committee
Signature:		Date:	29th May 2015

GENERAL INFORMATION

This document describes the BREATHER trial - a trial conducted by the Paediatric European Network for Treatment of AIDS (PENTA). The protocol should not be used as an aide-memoire or guide for the treatment of other young people with HIV infection. Every care was taken in its drafting, but corrections or amendments may be necessary. These will be circulated to the registered investigators in the trial, but centres entering young people for the first time are advised to contact one of the co-ordinating Trials Units to confirm they have the most up to date version. Clinical problems relating to this trial should be referred to the appropriate Trials Unit, who will transfer these to the appropriate medical expert.

The trial will be jointly co-ordinated and monitored by the **MRC Clinical Trials Unit at University College London, UK; INSERM SC10-US019, France and Program for HIV Prevention and Treatment (PHPT), Thailand.** Liaison between the Trials Units and clinical centres in each country will be similar to that organised for other PENTA trials, with a combination of direct liaison and liaison via a local co-ordinating centre. The trial will be supervised by the BREATHER Trial Management Group (TMG) who will report to the BREATHER Steering Committee (see Appendix 14 for the committee structure within PENTA). The Steering Committee may decide to terminate the trial for any reason including the recommendation of the PENTA Independent Data Monitoring Committee (IDMC).

- **Compliance**

The trial will be conducted in accordance with the protocol, EU Clinical Trials Directive 2001/20/EC Article 2, the principles of Good Clinical Practice (GCP) as laid down by the ICH topic E6 (Note for Guidance on GCP), applicable national regulations and national data protection legislation.

- **Sponsor**

Paediatric European Network for Treatment of AIDS (PENTA) Foundation.

Medical Research Council is co-sponsor for UK sites.

- **Funder**

NIHR Health Technology Assessment (HTA) for UK and Ireland.

Paediatric European Network for Treatment of AIDS (PENTA) - funding from the European Union Seventh Framework Programme (FP7/2007-2015) under EuroCoord grant agreement n° 260694.

- **Authorisation**

Ian Weller, Chair of BREATHER Steering Committee (i.weller@ucl.ac.uk)

Carlo Giaquinto, Chair of PENTA Steering Committee and Head of the PENTA Foundation (carlog@pediatria.unipd.it)

NOTE: Grey text in this document refers to the pilot phase

Main Contacts:

- **Chief Investigator**

Karina Butler, MB
Consultant Paediatrician
Our Lady's Children's Hospital
Crumlin
Dublin 12
Ireland

Tel: + 353 1 4096 338
Fax: + 353 1 4096 376
E-mail: karina.butler@olhsc.ie

- **Trial centres - Trial management, randomisations & SAE notification**

For UK, Ireland, Germany, Thailand (HIV-NAT), Uganda, USA, Ukraine

MRC Clinical Trials Unit at UCL
penta.mrcctu@ucl.ac.uk

Tel: + 44 20 7670 4939
Fax: + 44 20 7670 4814

For Spain, Denmark, Belgium, Argentina

INSERM SC10-US019
penta.sc10@inserm.fr

Tel: + 33 1 4559 5255
Fax: + 33 1 4559 5180

For Thailand (PHPT sites)

Program for HIV Prevention and
Treatment (PHPT)/IRD 174
Email: Suwalai@phpt.org

Tel: +66 (0) 5381 91 25/26/27/28/29
Fax: + 66 (0) 5381 9130

For full details of all trials contacts and committee memberships please see Appendix 16.

CONTENTS

ABBREVIATIONS AND GLOSSARY 6

1 SUMMARY 7

1.1 Aim and Objectives 7
1.2 Design 7
1.3 Population 8
1.4 Outcome measures 8
1.5 Follow-up 8
1.6 Duration 10
1.7 Substudies 11
1.7.1 Virology/Immunology Substudy (Appendix 11) 11
1.7.2 Qualitative Substudy – UK, Ireland, USA and Uganda (Appendix 12) 11
1.7.3 Adherence (MEMS cap) substudy – Selected sites 11
1.8 Schematic Diagram 12
1.9 FLOWSHEET FOR SCT PARTICIPANTS IN PILOT PHASE OF STUDY (first 15 randomisations to SCT). 14
1.10 FLOWSHEET FOR CT PARTICIPANTS IN PILOT PHASE OF STUDY (first 15 randomisations to continuous arm). 15
1.11 FLOWSHEET FOR MAIN PHASE OF STUDY: SCT arm (after pilot fully recruited) 16
1.12 FLOWSHEET FOR MAIN PHASE OF STUDY: CONTINUOUS ART ARM (and those in SCT arm who resume continuous ART) 17
1.13 FLOWSHEET FOR LONG TERM FOLLOW UP: 18

2 BACKGROUND 19

2.1 Introduction 19
2.2 Intermittent therapy 19
2.2.1 CD4 guided treatment interruptions 19
2.2.2 Fixed length treatment interruptions 20
2.2.3 Very short treatment interruptions 20
2.3 Short-Cycle therapy in adults and adolescents 20
2.4 Antiretroviral agents, viral load rebound and resistance 21
2.5 Rationale and objectives 22
2.6 Risks and benefits 23

3 SELECTION OF CENTRES/CLINICIANS 23

3.1 Clinical Sites 23
3.2 Site management 24

4 DESIGN 24

4.1 Design of Trial 24
4.2 Outcome measures- 25
4.3 Data collection and handling 26

5 SELECTION OF YOUNG PEOPLE 26

5.1 Inclusion criteria 26
5.2 Exclusion criteria 27
5.3 Siblings 27
5.4 Co-enrolment guidelines 27
5.5 Number and source of young people 27

6 ENROLMENT AND RANDOMISATION 28

6.1 Screening procedures and pre-randomisation investigations 28
6.1.1 Enrolment and consent 28
6.1.2 Investigations at screening visit 28
6.2 Week 0 visit and randomisation procedure 29

6.2.1 All participants 29

7 TREATMENT OF PATIENTS 29

7.1 Specific Requirements for the Pilot Phase 29

7.2 Management of young people and viral load tests during the Pilot Phase 30

7.3 Specific Requirements for the Main Trial 30

7.4 Management of young people and viral load tests during the Main Trial for the SCT arm 31

7.5 Considerations for the SCT arm for both Pilot Phase and Main Trial 31

7.5.1 Forgetting to stop antiretrovirals....31

7.5.2 Follow-up after re-starting continuous ART due to viral rebound in the SCT arm...31

7.6 The continuous ART arm 31

7.7 Immunisation 32

7.8 Trial products 32

7.9 Dispensing 32

7.10 Modification of trial treatment 32

7.11 Non-trial treatment 33

7.12 End of trial 33

8 ASSESSMENTS AND FOLLOW-UP 33

8.1 Schedule for follow-up 33

8.2 Clinical examination 33

8.3 Antiretroviral therapy 34

8.4 Laboratory tests 34

8.5 Cells and Plasma for storage 34

8.6 Adherence and acceptability 35

8.7 Measures of adherence 35

8.8 Measures of acceptability of treatment strategy and quality of life 35

8.9 Procedures for assessing safety 35

8.10 Long term follow up 36

8.11 Trial closure 36

8.11.1 Planned end of trial 36

8.11.2 Archiving of data 36

9 TRANSFER OF YOUNG PEOPLE AND WITHDRAWAL.... 37

9.1 Participant transfers.... 37

9.2 Transition to adult care 37

9.3 Withdrawal from trial intervention.... 37

10 SAFETY REPORTING.... 37

10.1 Definitions 37

10.2 Institution/Investigator Responsibilities 39

10.3 Investigator Assessment 39

10.4 Trials Units' responsibilities.... 40

11 STATISTICAL CONSIDERATIONS 42

11.1 Method of Randomisation.... 42

11.2 Outcome measures.... 42

11.3 Sample size 42

11.4 Interim analyses.... 44

11.5 Pilot phase.... 45

11.6 Analysis plan.... 45

12 TRIAL MONITORING 46

12.1 Risk assessment.... 46

12.2 Monitoring at the Trials Units.... 46

12.3 Clinical Site Monitoring.... 46

12.4 Confidentiality 47
13 ETHICAL CONSIDERATIONS AND APPROVAL 47
13.1 Ethical approval 47
13.2 Ethical considerations 47
14 REGULATORY ISSUES 48
15 INDEMNITY 48
16 FINANCE 49
17 DISSEMINATION 49
17.1 Publication 49
17.2 Feedback to participants 49
18 PROTOCOL AMENDMENTS 51
19 REFERENCES 58
20 APPENDICES 61

APPENDICES

APPENDIX 1: SAMPLE PATIENT INFORMATION SHEETS 61
APPENDIX 2: SAMPLE CONSENT FORMS 79
APPENDIX 3: SAMPLE GP LETTER 84
APPENDIX 4: ADHERENCE QUESTIONNAIRES 85
APPENDIX 5: ACCEPTABILITY QUESTIONNAIRES 94
APPENDIX 6: BREATHER – QUALITY OF LIFE (PEDSQL™) QUESTIONNAIRES 101
APPENDIX 7: BREATHER – SAMPLE DIARIES FOR RECORDING ANTIRETROVIRALS TAKEN 112
APPENDIX 8: CDC CLASSIFICATIONS 114
APPENDIX 9: TOXICITY GRADINGS 116
APPENDIX 10: TANNER SCALES 130
APPENDIX 11: VIROLOGY/IMMUNOLOGY SUBSTUDY 132
APPENDIX 12: QUALITATIVE SUBSTUDY: YOUNG PEOPLE AND HIV TREATMENT ADHERENCE: A MULTI-METHOD QUALITATIVE STUDY 133
APPENDIX 13: COMMONLY USED ANTIRETROVIRAL DRUGS GROUPED ACCORDING TO THEIR HALF LIVES 170
APPENDIX 14: PENTA COMMITTEE STRUCTURE 172
APPENDIX 15: FLOW CHART FOR MANAGING VIRAL LOADS 173
APPENDIX 16: TRIAL MANAGEMENT GROUP 175
APPENDIX 17: SAMPLE PATIENT INFORMATION SHEETS FOR LONG TERM FOLLOW UP (FOR YOUNG PEOPLE ON SCT) 178
APPENDIX 18: SAMPLE CONSENT FORMS FOR LONG TERM FOLLOW UP (FOR YOUNG PEOPLE ON SCT) 189
APPENDIX 19: SAMPLE PATIENT INFORMATION SHEETS FOR LONG TERM FOLLOW UP (FOR YOUNG PEOPLE ON CT) 193
APPENDIX 20: SAMPLE CONSENT FORMS FOR LONG TERM FOLLOW UP (FOR YOUNG PEOPLE ON CT) 203
APPENDIX 21: SAMPLE CONSENT FORMS FOR ADDITIONAL PLASMA (AND CELLS WHERE AVAILABLE) DURING LONG TERM FOLLOW UP 207

Abbreviations and Glossary

**AE**	Adverse event
**ANRS**	National Agency for Research (France)
**AR**	Adverse reaction
**ART**	Antiretroviral Therapy
**ARV**	Antiretrovirals
**c/ml**	Copies per ml
**Confirmed viral load**	Viral load tested in a separate blood sample (within a week).
**CDC**	Centres for Disease Control
**CI**	Confidence Interval
**CRF**	Case Report Form
**CTA**	Clinical Trials Authorisation (Europe)
**EDTA**	Ethylenediaminetetraacetic acid
**EFV**	Efavirenz
**ERC**	Endpoint Review Committee
**EU**	European Union
**EUDRACT**	European Union Drug Regulatory Agency Clinical Trial
**HAART**	Highly Active Antiretroviral Therapy
**HDL**	High-Density Lipoproteins
**HIV**	Human Immunodeficiency Virus
**HIV NAT**	HIV Netherlands Australia Thailand Research Collaboration
**ICH GCP**	International Conference on Harmonisation (of Technical Requirements for Registration of Pharmaceuticals for Human Use) Good Clinical Practice
**IDMC**	Independent Data Monitoring Committee
**INSERM, SC10-US019**	National Institute for Health and Medical Research (France)
**ISRCTN**	International standard randomised controlled trial number
**JCRC**	Joint Clinical Research Centre (Uganda)
**LDL**	Low-Density Lipoproteins
**MEMS cap**	Medication Event Monitoring Systems caps
**MRC CTU**	Medical Research Council Clinical Trials Unit(UK)
**NHS**	National Health Service (UK)
**NNRTI**	Non-nucleoside Reverse Transcriptase Inhibitor
**NRTI**	Nucleoside reverse Transcriptase Inhibitor
**NVP**	Nevirapine
**PENTA**	Paediatric European Network for Treatment of AIDS
**PHPT**	Program for HIV Prevention and Treatment (Thailand)
**PI**	Protease Inhibitor
**PIS**	Patient information Sheet
**PTI**	Planned Treatment Interruption
**Reproducible viral load**	Viral load retested in the same blood sample
**R&D**	Research & Development
**SAE**	Serious adverse event
**SAP**	Statistical analysis plan
**SAR**	Serious adverse reaction
**SCT**	Short-Cycle Therapy
**SUSAR**	Suspected unexpected serious adverse reaction
**TDM**	Therapeutic Drug Monitoring
**TMF**	Trial Master File
**TMG**	Trial Management Group
**TSC**	Trial Steering Committee
**UAR**	Unexpected adverse reaction
**UCL**	University College London
**VLDL**	Very Low-Density Lipoproteins
**VL**	Viral load
**Young Person**	8-24 years of age

1 SUMMARY

1.1 Aim and Objectives

The overall aim of the BREATHER trial is to evaluate the role of Short-Cycle Therapy (SCT) in the management of HIV-infected young people who have responded well to antiretroviral therapy (ART) and to determine whether young people with chronic HIV infection undergoing Short-Cycle Therapy of five days on ART and two days off maintain the same level of viral load suppression as those on continuous therapy, over 48 weeks.

To assess the advantages and disadvantages of the strategy, the incidence of toxicities, immunological control, resistance mutations, acceptability, quality of life and adherence to the randomised strategy will also be compared.

Importantly, because of insufficient data on short-term viral load rebound after stopping ART in this population, the trial will incorporate an initial pilot phase in selected centres, to assess the safety of the SCT strategy by evaluating detailed HIV-1 RNA profiles of participants on the SCT strategy.
For an overview of the trial design please see schematic diagram 1.8.

1.2 Design

BREATHER is an open, randomised, parallel group phase II/III trial. Young people will be randomised 1:1 into two groups:

1. Continuous ART
2. Short-Cycle Therapy

Pilot Phase
The first participants randomised in the study (15 in the SCT arm) will be included in the pilot phase and will have weekly HIV-1 RNA measurements during the first 3 weeks of the study. Those randomised to the SCT arm and included in the pilot phase should stop taking their antiretrovirals on Saturdays and Sundays i.e. will follow a cycle of 5 days on ART (Monday-Friday) and 2 days off (Saturday-Sunday) during the pilot phase. Recruitment to the continuous arm will run concurrently; young people randomised to continuous ART will continue their current ART regimen.* The IDMC will meet at the end of the pilot phase to review interim data. Recruitment will be on hold until this review has taken place.

Main Trial
Young people randomised to SCT will follow a cycle of 5 days on ART (Monday-Friday or Sunday-Thursday) and 2 days off (Saturday-Sunday or Friday-Saturday)*. Participants randomised to the SCT arm and included in this phase of the trial may choose which 2 days off ART they would prefer and whichever are chosen must be continued throughout the entire time on SCT within the study. Young people randomised to continuous ART will continue their current ART regimen**.

*Young people randomised to SCT can choose to take 2 consecutive days off during the week other than Saturday-Sunday or Friday-Saturday if these are the days that vary from their normal routine (for example, if a young person stays with a different family member on 2 days of the week then those 2 days could be the days taken off ART). The 2 days to be taken off should be decided at enrolment and should be adhered to throughout the study period.

**Young people randomised to continuous ART should only stop or switch all drugs in their ART regimen for virological, immunological or clinical failure according to local practice. However if simplification of the ART regimen or substitution of one drug is deemed necessary for clinical reasons, this may be allowed after discussion with the appropriate Trials Unit.

1.3 Population

A minimum of 160 HIV-1 infected young people, male and female, aged 8 to 24 years on a stable first-line HAART regimen containing at least 2 NRTIs/NtRTIs and EFV, that have an undetectable viral load for at least 12 months and are willing to continue the regimen throughout the study period. Young people on regimens containing NVP or a boosted protease inhibitor with undetectable viral load for at least 12 months wishing to enrol should first switch to EFV, and may be enrolled if they have 2 subsequent HIV-1 RNA measurements <50 copies/ml over a minimum period of 12 weeks.

Young people will be recruited from clinical centres in countries participating in the PENTA; PHPT and HIV-NAT networks (Thailand); and a single centre in Uganda.

1.4 Outcome measures

Pilot Phase Outcome Measure:
HIV-1 RNA ≥50 copies/ml, reproduced on a repeat test of the same sample off ART on the Monday following SCT at any of week 1, 2, 3 (see section 11.5).

Primary Outcome:
- HIV-1 RNA ≥50 copies/ml (**confirmed** on a separate sample within 1 week) at any of week 4, 12, 24, 36 or 48.

Secondary Outcomes:
- HIV-1 RNA <50 copies/ml at 24 and 48 weeks.
- Number of HIV mutations present at week 4, 12, 24, 36 or 48 conferring resistance to drugs taken at randomisation or during the trial.
- Change in CD4 (absolute and percentage) from randomisation to 24 and 48 weeks.
- Change in ART (defined as any change from the ART regimen at randomisation).
- Grade 3 or 4 clinical and laboratory adverse events.
- ART treatment modifying adverse events (all grades).
- New CDC stage B or C diagnosis or death
- Changes in fasting glucose, cholesterol, triglycerides, LDL, HDL and VLDL levels through 48 weeks.
- Adherence, acceptability, and quality of life over 48 weeks as assessed by patient completed questionnaires.
- HIV-1 RNA ≥50 c/ml (confirmed on a separate sample within 1 week) at any time throughout follow up.
- HIV-1 RNA <50 c/ml at 96 and 144 weeks.
- Adherence over the duration of the trial, as assessed by patient completed questionnaires.
- Change in CD4 (absolute and percentage) from randomisation to 96 and 144 weeks.

1.5 Follow-up

All young people will be seen for clinic visits at weeks -4 to -2 (screening), 0 (randomisation), 4, 8 (pilot only), 12, 24, 36 and 48.

Young people in the pilot phase will have 3 additional phlebotomy visits (HIV-1 RNA and blood store only) at weeks 1, 2, 3 and 8. For young people in the SCT arm these blood draws will be on the Monday after the 1st, 2nd and 3rd weekends off ART respectively and before ART recommences; for young people in the continuous arm these blood draws can be at any time during the 1st, 2nd and 3rd weeks.

The clinician may request more frequent visits for young people in either arm, if required. The flowcharts (sections 1.9, 1.10, 1.11, 1.12) indicate the minimum number of visits for protocol completion and data recording. However it is the investigator's responsibility to see participants as frequently as necessary, particularly for the monitoring of adverse events.

Management of young people and viral load tests (see Flow diagram, Appendix 15)

Pilot Phase

Current PCR tests for HIV-1 RNA can occasionally yield spurious results suggestive of low level viremia. During the pilot phase only, any HIV-1 RNA measurement that is detected at ≥50 c/ml at weeks 1, 2 or 3 will be repeated on the SAME SAMPLE to ensure that the result is valid and reproducible.

SCT arm:

Any participant with a reproducible viral load of >50c/ml (2 tests on the SAME sample) after the first weekend off treatment will not have a break over the 2nd weekend. On the Monday visit on week 2 after having taken medications at the weekend, the viral load will be repeated (confirmatory test).

- If this result is ≥50 c/ml then the participant should not undertake any further interruptions.
- If the confirmed test is <50c/ml, then the young person may undergo an interruption over the third weekend. If the young person has a 2nd reproducible viral load >50c/ml (2 tests on the SAME sample) after the third weekend, the young person should not undertake any further interruptions.

CT arm:

Participants with a reproducible viral load ≥50 c/ml (2 tests on the SAME sample), should receive standard clinical care.

Main Trial

SCT arm:

Participants with a HIV-1 RNA measurement ≥50 c/ml will have a confirmatory viral load measurement on a SEPARATE SAMPLE within 1 week. No further interruptions to antiretroviral therapy should be undertaken until the repeat test result is obtained.

Participants with a confirmed viral rebound of ≥50 c/ml should re-commence continuous ART and should not undergo further interruptions to their therapy.

Participants with an isolated increase of HIV-1 RNA ≥50 c/ml and subsequent measurement <50 c/ml can remain on SCT. There can only be a maximum of 3 such occurrences over any 12 month period. After the third increase within 12 months, continuous ART should be resumed with no further interruptions.

CT arm:

Participants with a HIV-1 RNA measurement ≥50 c/ml will have a confirmatory viral load measurement on a SEPARATE SAMPLE within 1 week. Participants with a confirmed viral load ≥50 c/ml, should receive standard clinical care.

Note: Viral load results with a higher cut-off than 50 c/ml e.g. < 80c/ml (due to technical issues, diluted samples or low volume) should not be considered a blip, but participants should still be instructed to return to clinic for a re-test to confirm their viral load is <50c/ml.

1.6 Duration

Young people will be recruited over 27 months and followed until the last randomised participant has completed 48 weeks of follow-up, at which point the main trial will be considered complete. Participants being followed after week 48 should be seen every 12 weeks until the main trial is complete. Participants randomised to the SCT arm should continue to follow the SCT strategy until the main trial is complete unless the clinician or the family have concerns which the clinician should discuss with the appropriate Trials Unit.
Stable and virologically suppressed young people who were randomised to SCT can continue SCT after completion of the main trial if agreed by the clinician and family at least until the results of the main trial become available. Management of HIV-1 RNA viral load should continue as specified in appendix 15.

Long term follow up:
The long-term impact of SCT in HIV-1 infected young people is unknown. Following the young people enrolled in BREATHER for two years after the main trial is complete will provide valuable data on the long-term effects of taking weekends off treatment compared to continuous treatment, and whether there is any long-term impact on subsequent disease progression and efficacy of future treatment.
Routine clinical data will be collected (see flowsheet 1.13) and 12-16 weekly HIV-1 RNA viral load monitoring will be funded for participants in both arms where this is not standard of care at the participating centre. Management of HIV-1 RNA viral load should continue as specified in appendix 15.

1.7 Substudies

1.7.1 Virology/Immunology Substudy (Appendix 11)

Plasma and cell storage will be required in both arms throughout the trial for detailed virological and immunological assessments (see Appendix 11). Flowsheets in section 1.9, 1.10, 1.11 and 1.12 give details of the timing of plasma and cell storage.

1.7.2 Qualitative Substudy – UK, Ireland, USA and Uganda (Appendix 12)

The qualitative substudy will be co-ordinated by London School of Hygiene and Tropical Medicine. This will involve interviews and audio diaries in a subset of 40 participants (20 in UK, Ireland, and the USA, 20 in Uganda with a total of at least 20 in the SCT arm) to gain information on a young person's experiences and feelings towards identity, treatment experience, adherence, transition to adulthood, expectation, and trial experience at the beginning and on nearing completion of the main trial. Focus groups may be conducted if feasible once the main trial is completed.

1.7.3 Adherence (MEMS cap) substudy – Selected sites

The adherence substudy will be carried out in a subset of 60 participants from selected sites (30 SCT, 30 continuous treatment). Additional consent will be sought for enrolment into this substudy which will use Medication Event Monitoring Systems (MEMS™ 6 TrackCap), to measure adherence to the protocol for both arms of the trial. MEMS caps fit standard size medication bottles, and record the time and date of each opening as a presumptive dose. MEMS caps will be used on one of the antiretroviral medication taken the most frequently for two 12-week periods in each participant.

1.8 Schematic Diagram

A minimum of 160 young people on a stable first-line HAART regimen

- On a stable first line HAART regimen containing 2 NRTIs/NtRTIs + EFV and willing to continue on this regimen
- HIV-RNA <50 c/ml for at least the prior 12 months (minimum of 3 measurements)
- No previous virological failure on a HAART regimen
- CD4 cell count ≥350 10^6/L at screening visit

RANDOMISATION

Continuous ART

Pilot (1st 15 young people recruited to continuous arm)
Phlebotomy visit (HIV-1 RNA and blood store only) weeks 1, 2 and 3

Follow-up
Clinic visits – week 4, 8 (pilot only), 12, 24, 36, 48 (then every 12 weeks until the end of the trial)

Short Cycle Therapy (SCT)

Pilot (first 15 children and young people recruited to SCT arm)
- Stop ART on Saturday/Sunday only
- Phlebotomy visit (HIV-1 RNA and blood store only) before ART recommences on Monday of week 1, 2 and 3.

Main Phase
- Stop on Friday/Saturday OR Saturday/Sunday. Participants must keep to the same two days off throughout the trial.

Follow-up
- Main and Pilot clinic visits – week 4, 8 (pilot only), 12, 24, 36, 48 (then every 12 weeks until the end of the trial)

For management of young people and viral load tests see appendix 15

1.9 FLOWSHEET FOR SCT PARTICIPANTS IN PILOT PHASE OF STUDY (first 15 randomisations to SCT).

WEEK	Screening -4 to -2	Randomisation 0	1, 2, 3 (Mon)[f]	4	8	12	24	36	48	Further follow-up	End of study visit
**SCT arm**											
Signed informed consent	X	*(confirm)*									
Clinical assessment [a]	X	X		X	X	X	X	X	X	Every 12 weeks	X
Tanner scales		X					X		X	Every 24 weeks	X
Lipodystrophy assessment		X					X		X	Every 48 weeks	X
Local HIV-1 RNA viral load [g]	X	X	X	X	X	X	X	X	X	Every 12 weeks	X
T cell lymphocyte subsets inc. RO/RA phenotype	X	X		X	X	X	X	X	X	Every 12 weeks	X
Biochemistry [b]	X	X				(X)	(X)	(X)	X	As per local practise	X
Haematology [c]	X	X				X	X	X	X	Every 12 weeks	X
Lipids/glucose [d]		X					X		X	Every 48 weeks	X
Pregnancy Test [e]	X						X		X	Every 24 weeks	X
Plasma storage [g]	X	X	X	X	X	X	X	X	X	Every 12 weeks	X
Cell storage [g]	X	X		X		X	X		X	Every 24 weeks	X
Adherence questionnaire	X			X		X	X	X	X	Every 12 weeks	X
Acceptability Questionnaire		X								If re-start continuous ART	X
PedsQL™ Questionnaire		X					X		X	Every 24 weeks	X

Notes: shaded boxes indicate visits where overnight fasting is required for blood lipids/glucose.
(X) – Indicates optional investigations.
If insufficient blood is drawn, priorities are: local HIV-1 RNA, T cell subsets, plasma store, lipids/glucose, biochemistry, and haematology

(a) Clinical assessment: including height & weight (adjust doses), presence of adverse events and change in HIV disease stage (including clinical lipodystrophy).
(b) Biochemistry: Creatinine, Bilirubin, ALT, AST, Alkaline Phosphatase and Albumin. Calcium and Phosphate at baseline and annually thereafter.
(c) Haematology: Hb, MCV, WBC, Lymphocytes, Neutrophils, Platelets
(d) Lipids/Glucose: Triglycerides, Cholesterol (Total, HDL, LDL, VLDL), Glucose. Overnight fasting required at randomisation and weeks 24 and 48 and then every 48 weeks.
(e) Pregnancy Test: either a blood or urine sample. This test will be performed for all females of childbearing potential at screening and weeks 24 and 48 (then every 24 weeks) and at other time-points if required. The initial pregnancy test must be done within 72 hours of enrolment and its results must be received before randomisation.
(f) Weeks 1,2,3 phlebotomy visits should be before recommencing ART on the Monday; sufficient blood should be sent to the virology laboratory for a repeat measurement of the same sample.
(g) 16ml total blood draw in EDTA for plasma and cell storage: 6.0ml in EDTA if plasma store only (see Manual of Operations for instructions for plasma and cell handling and storage].

1.10 FLOWSHEET FOR CT PARTICIPANTS IN PILOT PHASE OF STUDY (first 15 randomisations to continuous arm).

WEEK	Screening -4 to -2	Randomisation 0	1, 2, 3 [f]	4	8	12	24	36	48	Further follow-up	End of study visit
**SCT arm**											
Signed informed consent	X	*(confirm)*									
Clinical assessment [a]	X	X		X	X	X	X	X	X	Every 12 weeks	X
Tanner scales		X					X		X	Every 24 weeks	X
Lipodystrophy assessment		X					X		X	Every 48 weeks	X
Local HIV-1 RNA viral load [g]	X	X	X	X	X	X	X	X	X	Every 12 weeks	X
T cell lymphocyte subsets inc. RO/RA phenotype	X	X		X	X	X	X	X	X	Every 12 weeks	X
Biochemistry [b]	X	X				(X)	(X)	(X)	X	As per local practise	X
Haematology [c]	X	X				X	X	X	X	Every 12 weeks	X
Lipids/glucose [d]		X					X		X	Every 48 weeks	X
Pregnancy Test [e]	X						X		X	Every 24 weeks	X
Plasma storage [g]	X	X	X	X	X	X	X	X	X	Every 12 weeks	X
Cell storage [g]	X	X		X		X	X		X	Every 24 weeks	X
Adherence questionnaire	X			X		X	X	X	X	Every 12 weeks	X
PedsQL™ Questionnaire		X					X		X	Every 24 weeks	X

Notes: shaded boxes indicate visits where overnight fasting is required for blood lipids/glucose.
(X) – Indicates optional investigations.
If insufficient blood is drawn, priorities are: local HIV-1 RNA, T cell subsets, plasma store, lipids/glucose, biochemistry, and haematology

(a) Clinical assessment: including height & weight (adjust doses), presence of adverse events and change in HIV disease stage (including clinical lipodystrophy).
(b) Biochemistry: Creatinine, Bilirubin, ALT, AST, Alkaline Phosphatase and Albumin. Calcium and Phosphate at baseline and annually thereafter.
(c) Haematology: Hb, MCV, WBC, Lymphocytes, Neutrophils, Platelets
(d) Lipids/Glucose: Triglycerides, Cholesterol (Total, HDL, LDL, VLDL), Glucose. Overnight fasting required at randomisation and weeks 24 and 48 and then every 48 weeks.
(e) Pregnancy Test: either a blood or urine sample. This test will be performed for all females of childbearing potential at screening and weeks 24 and 48 (then every 24 weeks) and at other time-points if required. The initial pregnancy test must be done within 72 hours of enrolment and its results must be received before randomisation.
(f) Weeks 1,2,3 phlebotomy visits at any time during the week; sufficient blood should be sent to the virology laboratory for a repeat measurement of the same sample.
(g) 16ml total blood draw in EDTA for plasma and cell storage: 6.0ml in EDTA if plasma store only (see Manual of Operations for instructions for plasma and cell handling and storage].

1.11 FLOWSHEET FOR MAIN PHASE OF STUDY: SCT arm (after pilot fully recruited)

WEEK	Screening -4 to -2	Randomisation 0	4	12	24	36	48	Further follow-up	End of study visit
**SCT arm**									
Signed informed consent	X	*(confirm)*							
Clinical assessment [a]	X	X	X	X	X	X	X	Every 12 weeks	X
Tanner scales		X			X		X	Every 24 weeks	X
Lipodystrophy assessment		X			X		X	Every 48 weeks	X
Local HIV-1 RNA viral load	X		X	X	X	X	X	Every 12 weeks	X
T cell lymphocyte subsets inc. RO/RA phenotype	X	X	X	X	X	X	X	Every 12 weeks	X
Biochemistry [b]	X	X		(X)	(X)	(X)	X	As per local practise	X
Haematology [c]	X	X		X	X	X	X	Every 12 weeks	X
Lipids/glucose [d]		X			X		X	Every 48 weeks	X
Pregnancy Test [e]	X				X		X	Every 24 weeks	X
Plasma storage [f]	X	X	X	X	X	X	X	Every 12 weeks	X
Cell storage [f]	X	X	X	X	X		X	Every 24 weeks	X
Adherence questionnaire	X		X	X	X	X	X	Every 12 weeks	X
Acceptability Questionnaire		X						If re-start continuous ART	X
PedsQL^{TM} Questionnaire		X			X		X	Every 24 weeks	X

Notes: shaded boxes indicate visits where overnight fasting is required for blood lipids/glucose.
(X) – Indicates optional investigations.
If insufficient blood is drawn, priorities are: local HIV-1 RNA, T cell subsets, plasma store, lipids/glucose, biochemistry, haematology

(a) Clinical assessment: including height & weight (adjust doses), presence of adverse events and change in HIV disease stage (including clinical lipodystrophy).
(b) Biochemistry: Creatinine, Bilirubin, ALT, AST, Alkaline Phosphatase and Albumin. Calcium and Phosphate at baseline and annually thereafter.
(c) Haematology: Hb, MCV, WBC, Lymphocytes, Neutrophils, Platelets
(d) Lipids/Glucose: Triglycerides, Cholesterol (Total, HDL, LDL, VLDL), Glucose. Overnight fasting required at randomisation and weeks 24 and 48 and then every 48 weeks.
(e) Pregnancy Test: either a blood or urine sample. This test will be performed for all females of childbearing potential at screening and weeks 24 and 48 (then every 24 weeks) and at other time-points if required. The initial pregnancy test must be done within 72 hours of enrolment and its results must be received before randomisation.
(f) 16ml total blood draw for plasma and cell storage: 6.0ml in EDTA if plasma store only. [see Manual of Operations for instructions for plasma and cell handling and storage].

1.12 FLOWSHEET FOR MAIN PHASE OF STUDY: CONTINUOUS ART ARM (and those in SCT arm who resume continuous ART)

WEEK	Screening -4 to -2	Randomisation 0	4	12	24	36	48	Further follow-up	End of study visit
**Continuous ART arm**									
Signed informed consent	X	*(confirm)*							
Clinical assessment [a]	X	X	X	X	X	X	X	Every 12 weeks	X
Tanner scales		X			X		X	Every 24 weeks	X
Lipodystrophy assessment		X			X		X	Every 48 weeks	X
Local HIV-1 RNA viral load	X		X	X	X	X	X	Every 12 weeks	X
T cell lymphocyte subsets inc. RO/RA phenotype	X	X	X	X	X	X	X	Every 12 weeks	X
Biochemistry [b]	X	X		(X)	(X)	(X)	X	As per local practise	X
Haematology [c]	X	X		X	X	X	X	Every 12 weeks	X
Lipids/glucose [d]		X			X		X	Every 48 weeks	X
Pregnancy Test [e]	X				X		X	Every 24 weeks	X
Plasma storage [f]	X	X	X	X	X	X	X	Every 12 weeks	X
Cell storage [f]	X	X	X	X	X		X	Every 24 weeks	X
Adherence questionnaire	X		X	X	X	X	X	Every 12 weeks	X
Acceptability Questionnaire								If in SCT arm & re-start continuous ART	If in SCT arm & re-starting continuous ART
PedsQL™ Questionnaire		X			X		X	Every 24 weeks	X

Notes: shaded boxes indicate visits where overnight fasting is required for blood lipids/glucose.
(X) – Indicates optional investigations.
If insufficient blood is drawn, priorities are: local HIV-1 RNA, T cell subsets, plasma store, lipids/glucose, biochemistry, haematology

(a) Clinical assessment: including height & weight (adjust doses), presence of adverse events and change in HIV disease stage (including clinical lipodystrophy).
(b) Biochemistry: Creatinine, Bilirubin, ALT, AST, Alkaline Phosphatase and Albumin. Calcium and Phosphate at baseline and annually thereafter.
(c) Haematology: Hb, MCV, WBC, Lymphocytes, Neutrophils, Platelets
(d) Lipids/Glucose: Triglycerides, Cholesterol (Total, HDL, LDL, VLDL), Glucose. Overnight fasting required at randomisation and weeks 24 and 48, and then every 48 weeks.
(e) Pregnancy Test: either a blood or urine sample. This test will be performed for all females of childbearing potential at screening and weeks 24 and 48 (then every 24 weeks) and at other time-points if required. The initial pregnancy test must be done within 72 hours of enrolment and its results must be received before randomisation.
(f) 16ml total blood draw for plasma and cell storage: 6.0ml in EDTA if plasma store only [see Manual of Operations for instructions for plasma and cell handling and storage].

1.13 FLOWSHEET FOR LONG TERM FOLLOW UP:

TIMEPOINT AFTER FINAL MAIN TRIAL VISIT	Every 12-16 weeks post main trial	Annual long term follow up visit (48 weeks after final main trial visit)	Final follow up visit (Apr 2016-Jul 2016)
**Both arms**			
Clinical assessment [a]	X	X	X
Local HIV-1 RNA viral load [b]	X	X	X
CRF completion	X	X	X
Adherence questionnaire	X	X	X
Plasma and cell[c] storage (6 ml full blood in EDTA plus extra 10ml if cells being stored)		X	X

Notes:

(a) Clinical assessment: including presence of adverse events and change in HIV disease stage. Include CD4 count and document locally

(b) Management of HIV-1 RNA viral load should continue as specified in Appendix 15

(c) Cells storage at applicable sites

2 BACKGROUND

2.1 Introduction

Antiretroviral therapy (ART) has dramatically improved the prognosis for HIV-infected children. It has reduced early morbidity and increased survival, with more than 80% of children expected to reach adulthood [1]. HIV has been transformed from a devastating, rapidly progressive lethal condition into a chronic disease. Now the challenges for the treatment of HIV-infected children are to (i) maximise benefit of ART which prevents illness and encourages growth and development, (ii) minimise long-term drug toxicity and (iii) minimise the development of drug resistance so that children continue to have therapy options as they move through adolescence and into adulthood and (iv) to improve quality of life as much as possible for young people on ART.

Paediatric ART guidelines in 2008 advocate starting ART in infancy (<12 months of age) in all those diagnosed because of high risk of disease progression, following recent results from a randomised control trial [2-5]. Even if ART is not started early, vertically infected children face many more years of ART, often given throughout childhood, compared to adults. Therefore, there is a growing population of older children and young people who have already been on ART for many years and are continuing to face the challenge of taking daily medication [6].

A major challenge for young people with HIV, as for any chronic illness, is maintaining long-term adherence to treatment regimens [7-9]. The importance of adherence to the long term success of ART in maintaining virological suppression and prevention of emergence of resistance has been established [10-17]. However, experience with HIV-infected young people suggests that with current treatment strategies, adherence rates fall far below the 90-95% adherence associated with long term success [18-20]. Furthermore some studies have demonstrated a decline in adolescent adherence over time associated with duration on therapy [18]. Impediments to adherence for young people have been broadly categorised into two main groups; problems with medication such as taste and palatability issues, and adherence difficulties related to social situations [21]. While there have been considerable attempts to improve drug formulations, thus partly addressing the first impediment, the social dimensions are more complex; interference with daily life recurs as a common theme in assessments of poor adherence in young people.

New treatment strategies which promote adherence, minimise development of resistance, reduce long-term drug exposure while improving quality of life are required for young people 'burning out' on daily ART regimens. Approaches to achieve this include: (i) simplification of therapy (i.e. minimising the number of pills or swapping from twice to once-daily dosing), (ii) treatment interruptions (for example, based on levels of CD4 as in the PENTA 11 trial [22]; not currently advocated) and (iii) very short treatment interruptions (particularly at inconvenient times for taking mediation such as weekends) with the aim to maintain viral suppression; one such possible strategy is to give therapy during the week but allow a break at the weekend.

2.2 Intermittent therapy

2.2.1 CD4 guided treatment interruptions

The large phase III SMART trial evaluating a CD4-guided strategy of stopping ART when CD4 was above 350 cells/mm^3 and restarting when CD4 fell below 250 cells/mm^3 in adults was stopped early because of evidence of increased disease progression and cardiovascular events, albeit at low rates, in the interruption arm [23]. Treatment interruption where viral load rebound occurs is now not recommended in adults.

The PENTA 11 phase II trial (a randomised trial which compared CD4-guided PTIs versus continuous therapy in children aged 2 to 15 years) recently reported no significant increase in clinical progression in children undergoing planned treatment interruptions [22]. Further data on CD4 recovery and viral load suppression following reintroduction of continuous ART in all children in this trial are awaited and interruptions using this strategy cannot be currently recommended. However, the initial findings of PENTA 11 provided reassurance for two other paediatric trials currently investigating the impact of treatment interruptions (one evaluating interruptions following early limited ART in infants [5] and the other evaluating CD4 guided interruptions in 600 older children (BANA II trial).

2.2.2 Fixed length treatment interruptions

In adults, trials of fixed length ART schedule of one week on and one week off therapy (WOWO), based on the theory of autoimmunisation [24], showed high virological failure rates in patients following the WOWO strategy compared to continuous or CD4-guided ART [25,26].

2.2.3 Very short treatment interruptions

An alternative approach is to use very short interruptions (Short-Cycle Therapy) such that viral rebound should not occur, thus minimising the emergence of resistance as well as not compromising antiviral efficacy. This concept is based on the notion that >95% adherence may not be necessary for virologic suppression with all ARV regimens and that each ARV combination may have a unique adherence-resistance relationship [12]. Mathematical models of adherence and the emergence of resistance support this and the notion that otherwise strongly adherent patients might miss an acceptable number of doses of selected ARV's before resistance emerges [27].

2.3 Short-Cycle therapy in adults and adolescents

Two phase II trials of SCT in adults have recently been reported. Firstly a small single-arm pilot study of 5 days on, 2 days off ART in adults in the USA showed that long-term suppression of viral load could be achieved [28]. Two of 9 patients on PI-based HAART had confirmed virological rebound by 48 weeks compared with 1/10 patients on NVP-containing HAART and 0/8 patients receiving EFV-based regimens. As a result of this pilot, the randomised FOTO (Five On Two Off) trial in 60 adults with viral load <50 c/ml on TDF/ FTC/EFV was conducted comparing daily ART to a strategy of 5 days on, 2 days off treatment. 24-week results were recently reported, showing that all 25 patients in the FOTO arm and 24 of 28 patients (86%) in the daily arm who reached week 24 had viral load <50 c/ml at this time point [29]. Reasons for not reaching 24 weeks were psychological reasons (n=2), time burden (FOTO arm) (3), pregnancy (1), and loss to follow-up (daily ART arm)(1); of note, all had viral load <50 c/ml at discontinuation. There were 6 blips (viral load 50-500 c/ml) in the FOTO arm and 9 in the daily ART arm to week 24; there were no instances of virological failure (confirmed viral load >400 c/ml).

Secondly, in a Ugandan trial [30], 171 adults who had suppressed viral load <50 c/ml on a 3-drug ART regimen were randomised to receive either a week on, week off ART regimen (n=32; this arm of the study was discontinued early), a five-days on, two-days off schedule (SCT) (n=57) or continuous therapy (n=56). The majority of subjects (94%) received an EFV-based regimen. The trial showed that SCT was not inferior to continuous HAART; there were ten cases of failure in the continuous-therapy group (plus one death) and six in the SCT group (failure defined as VL >1000 c/ml, a decrease in CD4 count from randomisation of >30% or CD4 count <100 cells/mm^3, on 2 consecutive occasions through the 72 weeks of follow-up; or at 72 weeks, VL >400 c/ml or

development of an opportunistic infection). Levels of resistance were no different in the SCT group compared to continuous group. For patients on HAART containing stavudine, there was a significant decrease in the incidence of lactic acidosis in the SCT arm compared to continuous therapy.

The USA based Adolescent Trials Network (ATN) conducted the only study of SCT to date in adolescents, which had a non-randomised single arm design to assess viral load suppression (<400 c/ml, confirmed) on SCT (4 days on HAART, 3 days off) over 48 weeks [31]. Thirty-two participants aged 12 to 24 years of age and on a stable PI-based HAART regimen for at least 12 months were enrolled. Twelve of the 32 (38%) participants had confirmed virological rebound by 48 weeks; 7 out of 15 (47%) of those infected before 9 years of age and 5 out of 17 (30%) in those infected after 9 years of age (p=0.5). However 75% of children had been exposed to 5 or more drugs in the past, with prior history of virological failure. Overall adherence was good (88% of participants with >90% adherence) with no difference in those with or without viral load rebound (p=0.6).

2.4 Antiretroviral agents, viral load rebound and resistance

The plasma half-life of antiretroviral agents in an ART regimen (or intracellular half-life of NRTIs) and their genetic barrier to resistance are important factors which may influence viral load rebound and the development of resistance during a strategy of stopping therapy for 2 days every week. Appendix 13 gives details of the half lives of commonly used antiretroviral drugs, taken from a recent paper by Taylor *et al* [32].

An ART regimen containing three drugs, all with relatively similar long half-lives should maintain therapeutic concentrations of all 3 drugs over 2 days when treatment is stopped, avoid any risk of viral load rebound during the interruption and minimise the development of resistance. The regimen with the most favourable pharmacokinetic profile is TDF/FTC/EFV (median intracellular half-life of 150 hours, 39 hours and plasma half-life of between 36 and 100 hours respectively [32]). Co-formulation of TDF/FTC/EFV [33] in a single pill makes this a very attractive combination for older adolescents and young adults, however TDF is not yet licensed (although it is in frequent use) in children <18 years. However, in the Ugandan trial [30], EFV was given with either d4T/3TC or ZDV/3TC without evidence of inferior virological performance, even though d4T, 3TC and ZDV have shorter half-lives than TDF and FTC. Protease inhibitor drugs (e.g. Lopinavir/ritonavir (Kaletra) the most commonly used PI in children and adolescents) have substantially shorter half-lives, and therefore if a regimen containing 2 NRTIs (with longer half-lives) and a protease inhibitor (PI) (with a shorter half-life) are stopped together, "functional dual NRTI therapy" may result, with a risk of viral load rebound and the development of resistance.

Data on viral dynamics in the first few days following treatment cessation are scarce. Jacobsen et al in a 4-arm study of PTI ± HIV immunisation reported viral rebound to >50 c/ml following treatment interruption at a median (interquartile range) of 15 (8 to 31) days for those with prior PTI and of 21 (13 to 30) days for those without prior PTI [24]. Harrigan et al, whilst assuming a constant rate of viral increase that starts as soon as the patient stops therapy, suggest that many patients stopping therapy (previously suppressed) will have increases in plasma viral HIV RNA of about 0.2 log/day and will only reach detectable levels (>50 c/ml) within 1 to 2 weeks of stopping therapy [34].

A substudy of PENTA 11 evaluated the pharmacokinetics, viral load rebound and resistance profiles in 35 children stopping non-nucleoside reverse transcriptase inhibitor (NNRTI) based ART [35]. 21 children followed a staggered stop (SS) strategy whereby the NNRTI (NVP or EFV) was stopped at randomisation and the remaining 2 NRTI drugs stopped 7-14 days later, and 14 children followed a replacement strategy (RS) whereby the NNRTI was replaced with a PI and all drugs stopped 7-

14 days later. Results of HIV viral load testing in 8 children following SS and 7 following RS strategies show that the majority of children still had undetectable HIV RNA 5-8 days after stopping all drugs (minimum 12 days after stopping the NNRTI) (Table 1). No NNRTI resistance mutations were detected in any of the children in the substudy.

Table 1: Viral loads (c/ml) 5-8 days after 1st interruption, stopping ALL drugs in 15 children in the PENTA 11 PK substudy

Strategy	NNRTI	Days after stopping ALL drugs			
		5	6	7	8
SS	EFV	<50			<50
	NVP	<50, <50	159	<50, 89, 7000	
RS	EFV			<50	<50
	NVP			<50, <50, 52, 707	70

2.5 Rationale and objectives

Adherence issues tend to worsen for older children and adolescents after HIV diagnosis is disclosed and the young person starts taking charge of his/her own medication; self-consciousness and not wanting to be different from peers predominates. There is the additional burden of secrecy around HIV and ART [17], and social and family pressures prevent young people from sharing information of their diagnosis or treatment with friends [36]. The result is often worsening adherence with frequently missed doses, particularly at weekends which are typically times of socialising. Factors contributing to this include alcohol ingestion [18] as well as absence of school and overnight stays with friends at weekends. Therefore a regimen where ARVs need only be taken as part of the daily routine during weekdays could be attractive for older children (>8 years) as well as teenagers and young people (16-24 years) who continue to be followed at paediatric or affiliated adolescent units.

Data from adult studies evaluating the strategy of 5 days on, 2 days off are promising with low rates of virological rebound seen. However, no randomised trial has been undertaken in older children and adolescents, a population which has potentially more to gain in terms of quality of life, long-term adherence to mediation and the potential for better treatment options in adulthood. The effect on overall adherence of allowing 2 days off treatment per week is unknown and it is therefore important to first evaluate the strategy in young people who have a history of good adherence, the argument being that offering this strategy could prevent ad-hoc missed doses occurring. In view of the relatively shorter half-life of protease inhibitors and because both adult trials referred to above were undertaken with EFV-based regimens, enrolment will be limited to children who are on, or are willing to switch to, a regimen containing EFV.

This study aims to assess whether young people with chronic HIV infection undergoing Short-Cycle Therapy of five days on and two days off following complete virological response to first-line ART for at least 12 months maintain the same level of viral load suppression as those on continuous therapy. Importantly, because of insufficient data on short-term viral load rebound after stopping ART in this population, the trial will incorporate an initial pilot phase to assess the safety of the SCT strategy.

2.6 Risks and benefits

The potential risks of the SCT strategy are:

- The main risk is that the SCT strategy will prove ineffective in maintaining viral suppression, either because viral load cannot be maintained below detection levels during 2 days off ART, or due to non-adherence to the strategy by extending the time off treatment. However, if a raised viral load is confirmed on repeat testing (to be done within 1 week) then the participant will be placed back on continuous therapy.
- An additional risk is that young people, who have been fully adherent to ART prior to enrolment in the study, might extend the permitted very short interruptions and that overall adherence could decline. Of note, there is equipoise about whether adherence would be better or worse in the SCT arm compared with the continuous therapy arm as young people in the continuous arm may also not take their treatment regularly. All young people will be given diaries to record when they have taken ART and will be asked to comment about difficulties in remembering to take medication.
  Rebounding viral load comes with the risk of development of resistance (particularly to EFV and 3TC which have low genetic barriers to resistance) and which in turn may limit future therapeutic options. Resistance testing will be performed on all young people who lose virological suppression in either arm at the point of loss of suppression (≥50 c/ml), as well as on any subsequent samples with HIV-1 RNA ≥1000 copies/ml.

The potential benefits of the SCT strategy are:

- Improvement in quality of life by having weekends free from taking medication.
- Improved long-term adherence during the week.
- Decreased long-term toxicity to antiretroviral drugs (particularly relevant for some NRTI drugs e.g. ZDV and TDF).
- Decreased cost which is important to any healthcare service, and in particular for many parts of the world where HIV prevalence is highest.

Young people and carers will be fully informed of possible benefits and known risks by means of a patient information sheet as appropriate and this will be reinforced by discussions with the study research teams at the individual sites prior to enrolment.

Data from the pilot phase have been reviewed by the IDMC who identified no safety concerns and recommended that recruitment continue.

3 SELECTION OF CENTRES/CLINICIANS

3.1 Clinical Sites

Sites invited to enrol young people in BREATHER will usually have previously collaborated in PENTA trials with the MRC Clinical Trials Unit, INSERM, or PHPT. In the case of a new site wishing to participate, the site will need to demonstrate sufficient resources to conduct clinical trial research with young people. MRC Clinical Trials Unit, INSERM or PHPT staff will visit new sites prior to the trial commencing at that site in order to carry out a site set-up visit. This visit should be attended by the principal investigator at that site (the clinician), the clinic nurse(s), representatives from the laboratory and pharmacy, and any other staff who will be involved in the BREATHER trial. At the trial set-up visit the trial management staff should ensure that the following minimum criteria are met before the site enrols patients into the trial:

- The clinician should be experienced in the management of children/young people with HIV or work in close contact with a specialist HIV unit/site.
- Staff involved in the trial should have training in Good Clinical Practice (GCP)

- Pharmacy staff are able to keep detailed records of the trial medications prescribed
- There exists good communication between all of the above departments

Training will be provided for all sites participating in BREATHER. The principal investigator will sign an Investigator's Agreement for that institution on behalf of all staff at that site who will be working on the BREATHER trial. The principal investigator will sign to confirm that:

- The institution has an adequate number of qualified staff and adequate facilities for the foreseen duration of the trial to conduct the trial properly and safely.
- All staff assisting with the trial are adequately informed about the protocol, the investigational products and their trial related duties.
- The trial will be conducted in accordance with the current protocol and changes will only be made when necessary to protect the safety, rights or welfare of patients.
- The trial will be conducted in compliance with the principles of GCP and applicable regulatory requirements.
- The institution will permit monitoring and auditing by the relevant Trials Unit and inspection by the appropriate regulatory authorities. Direct access will be made available to all trial related sites, data/documents and reports.
- The institution will maintain a trial master file (TMF), which will contain essential documents for the conduct of the trial.
- All trial data will be submitted in a timely manner and as described in the protocol.
- All Serious Adverse Events (SAEs) will be reported to the relevant Trials Unit within one working day of the investigator becoming aware of the event. The initial SAE report shall be promptly followed by detailed follow up reports.
- No data on trial patients will be disclosed without the approval of the Trial Steering Committee (TSC).
- All trial related documents will be retained for at least 15 years after the completion of the trial.

In addition and in compliance with the principles of ICH GCP all institutions participating in the trial will complete a delegation log and forward this to the relevant Trials Unit. Each person working on the BREATHER trial must complete a section of this log and indicate their responsibilities. The Trials Unit must be immediately notified of any changes to trial personnel and/or their responsibilities. An up-to-date copy of this log must be stored in the TMF at the institution and also at the Trials Unit.

3.2 Site management

Sites in the United Kingdom and Ireland are managed by the MRC Clinical Trials Unit at UCL. Sites in the USA, Germany, Uganda, Thailand (HIV-NAT) and the Ukraine are managed by the MRC Clinical Trials Unit at UCL in collaboration with national co-ordinators. Sites in Spain, Belgium, Denmark and Argentina are managed by INSERM SC10-US019 in collaboration with national co-ordinators. PHPT sites in Thailand are managed directly by PHPT.

4 DESIGN

4.1 Design of Trial

BREATHER is an open, randomised, parallel group phase II/III trial. Young people will be randomised 1:1 into two groups:

1. Continuous ART

2. Short-Cycle Therapy (SCT)

This is a strategy trial. Blinding of patients to the randomised strategy would not be feasible and would be inappropriate as a key objective is to improve quality of life and adherence during the week by allowing days off at the weekend.

Recruitment of a minimum of 160 young people will be over a total of 27 months. All participants will be followed until the last participant has completed 48 weeks of follow-up. Young people and/or caregivers will be asked to consent to routine clinical data being provided for two years after the main trial is complete. Further information on long term follow can be found in Section 8.10.

Pilot Phase
The first 30 participants randomised in the study will be included in the pilot phase and will have weekly HIV-1 RNA measurements during the first 3 weeks of the study. Those randomised to the SCT arm and included in the pilot phase should stop taking their antiretrovirals on Saturdays and Sundays i.e. will follow a cycle of 5 days on ART (Monday-Friday) and 2 days off (Saturday-Sunday) during the pilot phase. Recruitment to the continuous arm will run concurrently; young people randomised to continuous ART will continue their current ART regimen* The IDMC will meet at the end of the pilot phase to review interim data. Recruitment will be on hold at other centres until this review has taken place.

Main Trial
Young people randomised to SCT will follow a cycle of 5 days on ART (Monday-Friday or Sunday-Thursday) and 2 days off (Saturday-Sunday or Friday-Saturday)*. Participants randomised to the SCT arm and included in the main phase of the trial may choose which 2 days off ART they would prefer and whichever are chosen must be continued throughout the entire time on SCT within the study. Young people randomised to continuous ART will continue their current ART regimen**. Participants recruited to the pilot phase and continuing the SCT strategy can change to Friday-Saturday off in the main study.

*Young people randomised to SCT can choose to take 2 consecutive days off during the week other than Saturday-Sunday or Friday-Saturday if these are the days that vary from their normal routine (for example, if a young person stays with a different family member on 2 days of the week then those 2 days could be the days taken off ART). The 2 days to be taken off should be decided at enrolment and should be adhered to throughout the study period.

**Young people randomised to continuous ART should only stop or switch all drugs in their ART regimen for virological, immunological or clinical failure according to local practice. However if simplification of the ART regimen or substitution of one drug is deemed necessary for clinical reasons, this may be allowed after discussion with the appropriate Trials Unit.

4.2 Outcome measures-

Pilot Phase Outcome Measure:
In order to examine whether SCT strategy results in an increase of viral load when ART is stopped, the pilot phase primary outcome measure is:

- HIV-1 RNA ≥50 copies/ml, reproduced on a repeat test of the same sample off ART on the Monday following SCT at any of week 1, 2, 3 (see section 11.5 for more details).

Main Trial:

Primary Outcome:
In order to compare the proportion of young people with plasma RNA values ≥50 copies/ml on SCT compared to continuous ART over 48 weeks, the primary outcome of the trial is:

- HIV-1 RNA ≥50 copies/ml (confirmed on a separate sample within 1 week) at any of week 4, 12, 24, 36 or 48.

This outcome measure only considers HIV-1 RNA measurements at these time points due to the difference in viral load monitoring in the pilot phase and the main trial. However if a young person enrolled in the pilot phase has HIV-1 RNA ≥50 copies/ml at weeks 1, 2 or 3 (reproducible on the same sample) or at week 8 (confirmed on a separate sample within 1 week), they will be considered as reaching the primary outcome at week 4 and 12 respectively (see section 11.6).

Secondary Outcomes:
The efficacy and safety of SCT and continuous ART will also be compared through the analysis of HIV-1 viral load and T cell subsets measured locally at each protocol visit, the presence of mutations conferring resistance to drugs taken at randomisation or during the trial, changes in the stage of a young person's HIV disease (CDC classification – see Appendix 8) measured at trial entry and each protocol visit, and the presence of adverse events. To assess the advantages and disadvantages of the strategy, acceptability, quality of life and adherence to the randomised strategy will also be compared. Specifically, the secondary outcomes of the trial are:
- HIV-1 RNA <50 c/ml at 24 and 48 weeks.
- Number of HIV mutations present at week 4, 12, 24, 36 or 48 conferring resistance to drugs taken at randomisation or during the trial
- Change in CD4 (absolute and percentage) from randomisation to 24 and 48 weeks
- Change in ART (defined as any change from the ART regimen at randomisation)
- Grade 3 or 4 clinical and laboratory adverse events.
- ART treatment modifying adverse events (all grades).
- New CDC stage B or C diagnosis or death
- Changes in fasting glucose, cholesterol, triglycerides, LDL, HDL and VLDL levels through 48 weeks
- Adherence, acceptability, and quality of life over 48 weeks as assessed by patient completed questionnaires
- HIV-1 RNA ≥50 c/ml (confirmed on a separate sample within 1 week) at any time throughout follow up
- HIV-1 RNA <50 c/ml at 96 and 144 weeks.
- Adherence over the duration of the trial, as assessed by patient completed questionnaires.
- Change in CD4 (absolute and percentage) from randomisation to 96 and 144 weeks.
-

4.3 Data collection and handling

Data will be recorded on CRFs; the top copy/original (or a scan of the original) should be sent to the appropriate Trials Unit for data entry and a copy kept at the local clinical centre. The type of data to be recorded is detailed in the Assessments and Follow up section (section 8). Data from the CRFs will be entered onto databases held at the co-ordinating Trials Units, and exported into Stata for analysis. After completion, adherence and acceptability questionnaires should be sent to the appropriate Trials Unit for data entry.

5 SELECTION OF YOUNG PEOPLE

5.1 Inclusion criteria

- HIV-1 infected young people aged 8 to 24 years inclusive (Young people recruited between the ages of 16-24 must either be in regular physical contact with their clinician or be able to transfer to an adult physician at the same site for follow-up or to an affiliated adult site).
- Parents/carers and/or young people, where applicable, willing to provide informed consent.
- On a stable first-line ART treatment containing at least 2 NRTIs/NtRTIs and EFV for at least 12 months and willing to continue the regimen throughout the study period. Young people on regimens containing nevirapine (NVP) or a boosted protease inhibitor with undetectable viral load for over one year who wish to enrol should switch to EFV. Once they are stable on the EFV containing regimen for more than 12 weeks they may be enrolled (must have 2 subsequent HIV-1 RNA measurements <50 c/ml over a minimum period of 12 weeks). Previous dual therapy and/or substitution of NRTIs is allowed providing any changes were not for disease progression, immunological or virological failure (where virological failure is defined as two successive HIV-1 RNA results>1000 c/ml) subsequent to virological control having been achieved on ART.
- Viral suppression (HIV-1 RNA <50 c/ml) for at least the prior 12 months (at least the last 3 measurements, including screening): young people who have experienced a single viral load >50 but <1000 copies/ml (preceded and followed by VL<50 c/ml) in the last 12 months can be enrolled.
- CD4 cell count ≥350 10^6/L at screening visit.
- Centre must routinely use an assay which detects HIV RNA-1 viral load ≥50 c/ml.

5.2 Exclusion criteria

- Pregnancy or risk of pregnancy in females of child bearing potential.
- Acute illness (young people may be enrolled after illness).
- Receiving concomitant therapy for an acute illness (young people may be enrolled after finishing therapy).
- A creatinine, AST or ALT of grade 3 or above at screening.
- On a regimen including nevirapine or a boosted PI (young people may switch to an EFV based regimen).
- Previous ART monotherapy (except for the prevention of mother-to-child transmission)

5.3 Siblings

If more than one child from the same family is eligible and carers give consent to enrolment into the trial, they will be enrolled to the same arm of the study in order to avoid problems caused by one sibling taking weekend breaks and not the other. Please indicate this in the relevant section of the randomisation form. (See section 11.1).

5.4 Co-enrolment guidelines

Co-enrolment in compatible studies is allowed by the protocol but must first be approved by the Trials Unit. Centres should also adhere to local guidelines concerning co-enrolment in other trials.

5.5 Number and source of young people

It is planned to recruit a minimum of 160 young people from clinical centres in countries participating in the PENTA, PHPT and HIV-NAT networks (Thailand), a centre in Uganda, a centre in the USA and a centre in the Ukraine.

6 ENROLMENT AND RANDOMISATION

6.1 Screening procedures and pre-randomisation investigations

6.1.1 Enrolment and consent

Enrolment will not start until each centre has received ethics and regulatory approval or approvals are imminent.

All participants and parent/carers will be given information about the objectives and rationale of the study and the possible risks (see sample patient information sheets in Appendix 1) unless the participant is unaware of their diagnosis; in this case only the parent/carer need be given the information sheet. Potential participants and parents/carers can consider entry into the trial for a longer period of time if necessary, if the participant continues to meet the eligibility criteria.

Young people should be assessed for entry to the trial based on the eligibility inclusion and exclusion criteria (see section 5.1 and 5.2) and if it is anticipated that HAART could be stopped for two days every week at the next clinic visit in 2-4 weeks' time.

Written informed consent to enter the trial must be obtained from participants and/or parents/carers (including legal authorities) as appropriate after explanation of the aims, methods, benefits and potential hazards of the trial and BEFORE any trial specific procedures are performed (see sample consent form in Appendix 2). All children who are deemed competent by the clinician and are aware of their HIV diagnosis will be given an age-specific information sheet (see Appendix 1) and asked to sign an assent form (see Appendix 2). If the participant is not aware of their diagnosis they will not be asked to give assent. For adolescents, consent should be obtained according to national regulations.

It must be made completely and unambiguously clear to parents and participants that they are free to refuse to participate in the trial, or withdraw their consent at any time and for any reason, without affecting their routine treatment. The signed informed consent form must be kept in the trial site file with a copy in the patient's medical records and one given to the family/participant (or as per local requirements). With the parents' and/or participant's consent, a letter should be sent to the participant's GP informing him/her of the trial and the young person's involvement in it (see Appendix 3).

6.1.2 Investigations at screening visit

Randomisation (week 0) should take place no more than four weeks after the screening visit and ideally as soon as possible after eligibility has been confirmed. At the screening visit a trial number will be assigned and used on all paperwork and labels. A clinical assessment should be completed and the participant and/or carer should complete an adherence questionnaire according to age and knowledge of HIV diagnosis (see flow sheets 1.9, 1.10, 1.11 and 1.12).

Blood will be taken for haematological and biochemical investigations, T cell subsets (including RO/RA phenotype if available), measurement of HIV-1 RNA viral load (the same assay should be used at least throughout the pilot phase and ideally throughout the whole trial, although the assays used may vary across centres according to clinical practice and management), and for plasma and cell storage (if the participant attends a clinical centre where this is possible).

As soon as the biochemistry and immunological/virological results are received and eligibility has been confirmed, the screening form with confirmation of eligibility and receipt of informed consent, clinical history and assessment, haematology, biochemistry and HIV-1 RNA viral load should be faxed to the appropriate Trials Unit.

6.2 Week 0 visit and randomisation procedure

6.2.1 All participants

The eligibility criteria and consent should be re-confirmed verbally and noted on the Randomisation Form. If the parents/carers and/or participant are prepared for Short Cycle Therapy to commence at this visit, contact the appropriate Trials Unit to enrol the participant (see General Information, 'Randomisations & SAE notifications' section at the front of the protocol for telephone and fax numbers). The Trials Units will inform the sites of the young person's allocation immediately (continuous ART or Short Cycle Therapy 5-on / 2-off strategy), which should be entered on the trial register.

A clinical assessment should be completed which will include measurements of height, weight, presence of adverse events, change in HIV disease stage and ethnic origin. The following investigations are to be performed: haematology, biochemistry, glucose and lipid profile (fasting), T cell subsets (plus RO/RA phenotype if available). Blood should be taken for plasma storage (plus cell storage if the participant attends a clinical centre where this procedure is possible or where a courier can be arranged). A quality of life questionnaire (PedsQL™) should be completed for all participants (and carers) and an acceptability questionnaire for those young people randomised to SCT (and carers) (see flow sheets 1.9, 1.10, 1.11 and 1.12). Participants should be given a diary to record when they have taken their ART, which will include a reminder to re-start therapy after the 2 days off ART in the SCT arm (Appendix 7). The diary will be anonymised by the clinical centre staff before being sent to the Trials Units.

For further details on specific requirements for the week 0 visit and randomisation procedures see sections 7.1, 7.3 and 7.6.

7 TREATMENT OF PATIENTS

7.1 Specific Requirements for the Pilot Phase

At least the first 30 participants enrolled should be willing to stop ART on the Saturday-Sunday and be able to attend the hospital for phlebotomy visits and a blood store at weeks 1, 2, and 3 on the Monday (as the first 15 randomised to the SCT arm will have to attend these weekly phlebotomy visits, those randomised to continuous ART can attend at anytime during the week).
If any one of the three Monday visits are due to fall on a public holiday please delay enrolment in the trial.
Participants in the pilot phase should be asked to come to the randomisation visits within 2-4 weeks of the screening visit, having fasted from the previous evening. **Randomisation should only take place when the young person is able to start SCT the following weekend AND attend the subsequent Monday visits.**

Participants in the pilot phase will stop Saturday-Sunday but can change to Friday-Saturday at the end of the pilot phase.

7.2 Management of young people and viral load tests during the Pilot Phase

Current PCR tests for HIV-1 RNA can occasionally yield spurious results suggestive of low level viremia. Any HIV-1 RNA measurement that is detected at ≥50 c/ml at weeks 1, 2 or 3 will be repeated on the **SAME SAMPLE** to ensure that the result is valid and reproducible. All participants in the pilot phase will have an additional visit at week 8.

SCT arm:

Any participant with a reproducible viral load of ≥50c/ml (2 tests on the SAME sample) after the first weekend off treatment will not have a break over the 2nd weekend. On the Monday visit on week 2 after having taken medications at the weekend, the viral load will be repeated (confirmatory test).

- If this result is ≥50 c/ml then the participant should not undertake any further interruptions.
- If the confirmed test is <50c/ml, then the child may undergo a second interruption over the third weekend. If the young person has a 2nd reproducible viral load ≥50c/ml (2 tests on the SAME sample) after the third weekend, the young person should not undertake any further interruptions.

For further details please see appendix 15

Once the 15th participant randomised to the SCT arm has been recruited to the pilot phase screening and randomisation will be put on hold in both arms. The IDMC will meet at the end of the pilot phase when the 15th participant randomised to the SCT arm has completed 3 weeks (or 4 weeks if they require a confirmatory viral load for a sample at 3 weeks), to review the information collected and make recommendations to the Trial Steering Committee about the continuation of the trial (see section 11.5).

Young people in both arms enrolled during the pilot phase will continue to be followed as per randomised arm in the main phase of the trial.

Continuous ART arm:

Participants will be seen during weeks 1, 2 and 3 for blood store and viral load measurement (not necessarily on a Monday). If a viral load result of ≥50c/ml is reproducible, clinic staff should discuss adherence to continuous ART with the young person.

7.3 Specific Requirements for the Main Trial

Participants enrolled in the main phase of the trial will be able to choose the days off (Friday-Saturday or Saturday-Sunday)*. Whichever is chosen must be continued throughout the entire time on SCT within the study.

*Consecutive days off alternative to Saturday-Sunday or Friday-Saturday can be chosen to better suit a young person's normal routine.

The two days off ART treatment indicated by the participant at screening should be confirmed on the Trial Entry form. It should be made very clear that the chosen 2 days off should remain the same throughout the length of the trial.

Participants in the main phase of the trial should be asked to come to the randomisation visits as soon as possible following the screening visits (once eligibility has been confirmed and within a maximum of 4 weeks), having fasted from the previous evening.

A full clinic visit should be scheduled for 4 weeks after the randomisation visit i.e. a week 4 visit.

7.4 Management of young people and viral load tests during the Main Trial for the SCT arm

Participants with a HIV-1 RNA measurement ≥50 c/ml will have a confirmatory viral load measurement on a **SEPARATE SAMPLE** within 1 week. No further interruptions to antiretroviral therapy should be undertaken until the repeat test result is obtained.

Participants with a confirmed viral rebound of ≥50 c/ml should re-commence continuous ART and should not undergo further interruptions to their therapy.

Participants with an isolated increase of HIV-1 RNA ≥50 c/ml and subsequent measurement <50 c/ml can remain on SCT. There can only be a maximum of 3 such occurrences over any 12 month period. After the third increase within 12 months, continuous ART should be resumed with no further interruptions.

Note: Viral load results with a higher cut-off than 50 c/ml e.g. <80c/ml (due to technical issues, diluted samples or low volume) should not be considered a blip, but participants should still be instructed to return to clinic for a re-test to confirm their viral load is <50c/ml.

For further details please see Appendix 15.

7.5 Considerations for the SCT arm for both Pilot Phase and Main Trial

7.5.1 Forgetting to stop antiretrovirals

If a patient in the SCT arm forgets to stop taking their medication for one or both of the days during their pre-defined break they will need to continue with taking the medication during the 5 days on and then stop at the next, pre-defined break.

7.5.2 Follow-up after re-starting continuous ART due to viral rebound in the SCT arm

The participant should be seen at weeks 4 and 12 and then 12-weekly (see flowsheet section 1.12). ART should be continued without any further days off. Participants in the SCT arm who restart therapy should complete an acceptability questionnaire at the point of restarting treatment.

Any changes in the antiretroviral regimen are at the discretion of the clinician but should be discussed with the appropriate Trials Unit. Participants resuming continuous ART should follow standard local practice at subsequent visits, including checking ART doses at every visit and adjusting for height and weight as necessary and should be followed until the end of the trial. Forms should be completed at each follow up visit.

7.6 The continuous ART arm

Young people randomised to continuous ART should continue on their current antiretroviral therapy and should follow standard local practice at subsequent visits, including checking ART doses at every visit and adjusting for body surface area or weight as necessary.
A full clinic visit should be scheduled for 4 weeks after the randomisation visit i.e. a week 4 visit.

If the HIV-1 RNA increases to ≥50 c/ml at any time, the participant should be brought in for a confirmatory measurement on a separate sample within 1 week and should be managed as per standard clinical care.

7.7 Immunisation

If a viral load increase has occurred within two weeks of the participant having received an immunisation, the confirmatory viral load test should be performed immediately after the young person is two weeks post vaccination. The young person should go back to continuous treatment following the viral load increase and should remain on ART until the result of the confirmatory second sample is known.

7.8 Trial products

Regimens containing 2 NRTIs/NtRTIs and EFV will be included.

SCT Arm
At the end of each 2–day-SCT, participants must re-start the same regimen for the following 5 days.

Both Arms
For both arms, if a change in antiretroviral therapy is indicated the appropriate Trials Unit should be contacted and this should be recorded on the appropriate CRF (including reasons and dates of any changes).

7.9 Dispensing

Routine supplies of ART will be used; clinical trial labels will be supplied for participants in the SCT arm. A dispensing log should be completed for each patient. Clinics must check that families have an ABC alert card if the participant is taking abacavir (ABC).

7.10 Modification of trial treatment

For both arms, if a change in antiretroviral therapy is indicated, the appropriate Trials Unit should be contacted. If simplification of the ART regimen is deemed necessary for clinical reasons during follow-up, this may be allowed, but must be discussed with the appropriate Trials Unit. **Switching of all drugs in the current ART regimen should only occur if there is immunological, virological or clinical failure (according to local clinical practice)**, at which point a new regimen should be chosen, this applies for the SCT arm who should re-start continuous therapy in this situation. Resistance tests or TDM may be used to inform change of regimen, according to local clinical practice.

Hypersensitivity reactions with ABC rarely occur after the first 6 weeks of therapy. However if the participant has a suspected hypersensitivity reaction and ABC is stopped (as per standard guidelines), rechallenge with ABC should NEVER take place. If therapy with ABC has been discontinued for any reason and restarting therapy is under consideration, the reason for discontinuation must be established to assess whether the patient had any symptoms of a hypersensitivity reaction. If a hypersensitivity reaction cannot be excluded, ABC must not be restarted. However restarting ABC after a scheduled SCT break is safe.

All other drugs should be modified (if required) as per standard clinical practice.

7.11 Non-trial treatment

No concomitant medication for an acute illness should be taken at the time of study enrolment. Medication required for a concurrent illness during the study should be documented on the appropriate form.

7.12 End of trial

Stable and virologically suppressed young people who were randomised to SCT can continue SCT after completion of the main trial if agreed by the clinician and family at least until the results of the main trial become available. 12-16 weekly HIV-1 RNA viral load monitoring will be funded by the PENTA Foundation where this is not local standard of care. Management of HIV-1 RNA viral load should continue as specified in appendix 15.

The trial will be considered to have ended once all participants have had their final long term follow up visits. See section Trial Closure 8.11.

8 ASSESSMENTS AND FOLLOW-UP

Complete details of local laboratory issues and logistics are given in the Manual of Operations. Please also refer to flow sheets 1.9, 1.10, 1.11, 1.12 and 1.13 for the specific requirements at each visit.

8.1 Schedule for follow-up

Participants being followed after week 48 should be seen every 12 weeks, until the last young person enrolled has completed 48 weeks of follow-up. Participants randomised to the SCT arm should continue to follow the SCT strategy until the end of the main trial unless the clinician or the family have concerns which they should discuss with the appropriate Trials Unit.

The clinician may request more frequent visits for participants in either arm, if required. The flowcharts (section 1.9, 1.10, 1.11, 1.12) indicate the minimum number of visits for protocol completion and data recording. However it is the investigator's responsibility to see participants as frequently as necessary, particularly for the monitoring of adverse events.

Participants should be <u>fasting</u> at week 0, 24 and 48 in order to obtain fasting lipids and glucose values.

8.2 Clinical examination

A clinical examination must be performed at screening, randomisation and at identified follow-up protocol visits (see flowcharts 1.9, 1.10, 1.11, 1.12). At each visit the following should be recorded:

- Body weight and height
- All adverse events since last protocol visit, including in particular haematological abnormalities, pancreatitis, diarrhoea, clinical lipodystrophy, acute illnesses
- Change in HIV disease stage since last protocol visit

A physician assessment of lipodystrophy assessment should be performed at week 0 and repeated every 24 weeks until the end of the study. Tanner scales should also be completed at these time-points (Appendix 10).

Pregnancy tests should be performed for all females of childbearing potential at screening (week -4 to -2) and repeated every 24 weeks until the end of the trial and at other time-points if required. The pregnancy test at screening must be done within 72 hours of enrolment and its results must be received before randomisation. The pregnancy test can be either a urine sample or blood sample test. If a pregnancy test performed during the trial is positive please contact and discuss with the appropriate Trials Unit.

Ethnic origin data will be collected because it is known that ethnicity is a factor in the concentration levels of efavirenz [39].

8.3 Antiretroviral therapy

Prescriptions of antiretroviral therapy and any alterations to prescribed doses should be recorded on the CRF. Doses should be checked at every visit and adjusted for body surface area, or weight as appropriate.

8.4 Laboratory tests

Laboratory tests for efficacy and safety monitoring will include:

Haematology:	Haemoglobin, MCV, platelets<br>white cell count, neutrophil and lymphocyte counts
Biochemistry:	Creatinine, albumin, total bilirubin, ALT (SGOT), AST (SGPT),<br>alkaline phosphatase, calcium, phosphate.
Lipids/glucose:	Triglycerides, cholesterol (total, LDL, HDL, VLDL), glucose<br>*(participants should be <u>fasting</u> overnight at randomisation, weeks 24 & 48 and then every 48 weeks during the main trial)*
Lymphocyte subsets:	CD3 (absolute and percentage)<br>CD3+CD4 (absolute and percentage*)<br>CD3+CD8 (absolute and percentage*)<br>Total lymphocyte count (if measured by immunology laboratory)<br>* including RA/RO phenotype
Virology:	HIV-1 RNA (viral load) using an ultrasensitive assay. It is also planned to do resistance testing on stored samples.

8.5 Cells and Plasma for storage

6ml of EDTA blood will be collected throughout the trial for plasma storage for viral load testing and analyses as part of the virology/immunology substudy (Appendix 11), and other HIV related tests as appropriate. Plasma will be stored at weeks -4 to -2 (screening), week 0 (randomisation), 4, 8 (pilot only), 12, 24, 36 and 48 from all participants in both arms. Plasma will also be stored at weeks 1, 2 and 3 for participants in the pilot phase. (See flowsheets)

In addition, in centres who have capability, cells from all participants will be stored at weeks -4/-2 (screening), 0, 4, 12, 24, 48 and every 24 weeks until the end of the trial at –80º C for subsequent assessments as part of the virology/immunology substudy (see Appendix 11). An extra 10ml of whole blood is required at the time points for cell storage.

8.6 Adherence and acceptability

All carers and children, where appropriate, will be asked to complete adherence and acceptability questionnaires in accordance with flow charts 1.9, 1.10, and 1.11.
Adherence and acceptability questionnaires should be completed without conferring with friends or relatives and all questions should be answered even if the carer/young person feels them to be irrelevant. The appointed person at the site should check each questionnaire for its completeness, ensuring that the correct date of completion and patient identifiers are present. The Research Nurse should approach young people at appropriate clinical visits to complete a questionnaire.

For children unaware of their diagnosis or not deemed to be sufficiently competent by the paediatrician, the parents or carer should complete a questionnaire on behalf of the child.

8.7 Measures of adherence

Close monitoring of viral load is likely to rapidly identify non-adherence to ART in both arms. Additionally each participant and/or caregivers (as appropriate depending on age and knowledge of HIV diagnosis) should complete an adherence questionnaire at weeks -2, 4, 12, 24, 36 and 48.

Adherence to strategy could be affected if the young people recruited to the SCT arm decide to extend the permitted weekend-only interruptions or forget to stop taking the medication on the days off. To combat this potential issue, clinic staff will be instructed to stress the importance of keeping to the strategy within the trial. Additionally the patients/parents/carers (as appropriate) will complete a diary which will assist in reminding participants to re-start therapy after the 2 days off ART (Appendix 7). Participants in the continuous ART arm will also complete a diary to look at adherence to their ART regimen 7 days a week.

Clinical teams may organise for text message reminders to be sent to participants' mobile to assist with adherence. This should only be done if the participant thinks this will be beneficial and agrees to this.

Further adherence measures include using MEMS cap. For further details see section 1.7.3.

8.8 Measures of acceptability of treatment strategy and quality of life

Participants randomised to the SCT arm (as appropriate depending on age and knowledge of HIV diagnosis) should complete a self-administered acceptability questionnaire at randomisation (week 0) and at the final visit if following the SCT strategy, or at the re-start of continuous therapy. Where a parent/carer is involved, they should complete the relevant questionnaire.

Quality of life (PedsQL™) questionnaires should be completed by all participants (regardless of which arm they are in) at randomisation (week 0) and then every 24 weeks.

8.9 Procedures for assessing safety

A clinical examination should be performed at screening, week 0 and at all follow-up protocol visits. At each visit any adverse event since the last protocol visit should be recorded (see toxicity

grading table, Appendix 9). Any event fitting the ICH definition of serious (see section 10.1) should be reported to the Trials Unit on an SAE form within one working day of the site being aware of the event. All other adverse events should be reported on the Follow-up CRF.

Further details on reporting of adverse events are presented in section 10.

8.10 Long term follow up

The long-term impact of SCT in HIV-1 infected young people is unknown. Following the young people enrolled in BREATHER for two years after the main trial is completed will provide valuable data on the long-term effects of taking weekends off treatment compared to continuous treatment, and whether there is any long-term impact on subsequent disease progression and efficacy of future treatment.
Routine clinical data will be collected and 12-16 weekly HIV-1 RNA viral load monitoring will be funded for participants in both arms where this is not local standard of care. Adherence questionnaires will be completed every 12-16 weeks (see flowsheet 1.13). A plasma sample and cells (where applicable) will be stored at the annual (48 weeks after the final main study visit) and final long term follow up visit. Management of HIV-1 RNA viral load should continue as specified in appendix 15.

Investigators will continue to be responsible for safety reporting as in the main trial (see section 10).

8.11 Trial closure

8.11.1 Planned end of trial

The main trial will be considered complete after the last patient to be enrolled reaches 48 weeks of follow-up. However, results will not be available for approximately 6 months after completion of the main trial when all data have been received and analysed by the Trials Units. The date of the expected completion of the main trial will be communicated to the participating centres approximately 3-6 months beforehand.

The TSC will continue observational follow-up of all patients enrolled in the trial for a further two years after the main trial is completed providing consent is given. .
The trial will be considered to have ended once all participants have had their final long term follow up visits.
The sponsor, or the sponsor’s representative in each country, will notify the national regulatory body and ethics committee of the closure of the trial. The Principal Investigator at each site will be responsible for notifying local ethics boards and any other local bodies, such as R&D departments.

8.11.2 Archiving of data

Data will be stored for 15 years and will be treated in accordance with the UK Data Protection Act of 1998. Blood samples will be shipped to a central storage facility after completion of the sub studies.

9 TRANSFER OF YOUNG PEOPLE AND WITHDRAWAL

9.1 Participant transfers

For young people moving from the site at which they enrolled to a new site, every effort should be made for them to be followed at the new site and for this site to take over responsibility for the patient. If the site was not listed on the initial CTA, an appropriate Principal Investigator will need to be identified and regulatory and ethics approvals obtained at the new site. A set-up visit will be carried out at the new site and copies of all trial-related documents will be provided. The care giver/participant will need to sign a new consent form at the new site. Until this happens, the follow-up of the patient within the trial remains the responsibility of the recruiting site.

9.2 Transition to adult care

Young people recruited between the ages of 16-24 must either be in regular contact with their clinician or be able to transfer to an adult physician at the same site for follow-up or have follow-up at an affiliated adult site (where ethics and R&D have been obtained).
When a young person becomes 18 (or at an earlier age if required by national regulations) during the trial, they must give consent if this has not been gained previously.

9.3 Withdrawal from trial intervention

Caregivers and young people have the right to withdraw from the allocated intervention (continuous therapy or short cycle therapy) at any time for any reason. Should a caregiver or young person decide to withdraw from either the protocol and allocated intervention, from routine follow-up data, or withdraw all data, a complete evaluation should be made with an explanation of why the young person is withdrawing, unless consent is specifically withheld. If clinicians wish to withdraw a young person they should discuss this first with the appropriate Trials Unit.

Wherever possible, participants should continue to be followed for clinical and laboratory assessments and information recorded 12 weekly unless the caregiver and the young person explicitly withdraw consent for observational follow-up. If possible the 48 week T-cell subsets and plasma stores should be obtained.

10 SAFETY REPORTING

ICH GCP requires that both Investigators and Sponsors follow specific procedures when notifying and reporting adverse events/reactions in clinical trials. These procedures are described in this section of the protocol. Section 10.1 lists definitions, section 10.2-3 gives details of the institution/investigator responsibilities and section 10.4 provides information on the Trials Units' responsibilities (on behalf of the sponsor).

Please note the changes from previous PENTA protocols on the reporting of HIV-related signs and symptoms: these are now considered AEs or SAEs as appropriate.

10.1 Definitions

The definitions of the EU Directive 2001/20/EC Article 2 based on ICH GCP apply in this trial protocol. These definitions are given in the table below.

Table 2: Definitions

Term	Definition
**Adverse Event (AE)**	Any untoward medical occurrence in a patient or clinical trial subject to whom a medicinal product has been administered including occurrences which are not necessarily caused by or related to that product.
**Adverse Reaction (AR)**	Any untoward and unintended response to an investigational medicinal product related to any dose administered.
**Unexpected Adverse Reaction (UAR)**	An adverse reaction, the nature or severity of which is not consistent with the information about the medicinal product in question set out in the summary of product characteristics for that product.
**Serious Adverse Event (SAE) or Serious Adverse Reaction (SAR) or Suspected Unexpected Serious Adverse Reaction (SUSAR)**	Respectively any adverse event, adverse reaction or unexpected adverse reaction that:<br>• results in death<br>• is life-threatening*<br>• requires hospitalisation or prolongation of existing hospitalisation**<br>• results in persistent or significant disability or incapacity<br>• consists of a congenital anomaly or birth defect<br>• other medically important/clinically significant events***

*The term 'life-threatening' in the definition of 'serious' refers to an event in which the patient was at risk of death at the time of the event; it does not refer to an event which hypothetically might have caused death if it were more severe.

**Hospitalisation is defined as an inpatient admission, regardless of length of stay, even if the hospitalisation is a precautionary measure for continued observation. Hospitalisations for a pre-existing condition (including elective procedures that have not worsened) do not constitute an SAE. Therefore, if an admission is due to a change of HAART regimen without any adverse event, this will not constitute an SAE.

***Medical judgement should be exercised in deciding whether an AE/AR is serious in other situations. Important AE/ARs that are not immediately life threatening or do not result in death or hospitalisation but may jeopardise the subject or may require intervention to prevent one of the other outcomes listed in the definition above, should also be considered serious.

Disease progression (refer to appendix 8 for CDC classifications) which meets the seriousness criteria or death as a result of disease progression are now considered to be SAEs and should be reported as a SAE. They should be graded according to the guidelines in the toxicity table (appendix 9) under 'Clinical Symptoms not otherwise specified in this table'.

Any change in social circumstances or admissions to hospital due to such a change will not constitute an SAE.

Pregnancies are notable events and should be reported on an SAE form.

10.2 Institution/Investigator Responsibilities

All SAEs and pregnancies must be reported by the investigator to the appropriate Trials Unit on a Serious Adverse Event (SAE) form within one working day of the investigator being aware of the event. **The investigator has a responsibility to report all such events for the entire duration of follow up in the trial.**

All other AEs/ARs should be reported on the follow-up forms.

The severity (i.e. intensity) of all AEs/ARs (serious and non-serious) in this trial should be graded using the toxicity gradings in Appendix 9.
Where a pregnancy event has occurred in a participant on SCT, the participant should be instructed to return to continuous therapy.

10.3 Investigator Assessment

(a) Seriousness
When an AE/AR occurs the investigator responsible for the care of the patient must first assess whether the event is serious using the definition given in Table 2. If the event is serious, then an SAE form must be completed and the Trials Unit notified.

(b) Causality
The Investigator must assess the causality of all serious events/reactions in relation to the trial therapy using the definitions in Table 3. There are 5 categories: unrelated, unlikely, possible, probable and definitely related. If the causality assessment is unrelated or unlikely to be related the event is classified as a SAE. If the causality is assessed as either possible, probable or definitely related then the event is classified as a SAR. If a drug is at least possibly related to an AE, this should be noted on the follow up form.

(c) Expectedness
If the event is a SAR the Investigator must assess the expectedness of the event. If a SAR is assessed as being unexpected it becomes a SUSAR. The definition of an unexpected adverse reaction (UAR) is given in Table 3 and is one not previously reported in the current summary of product characteristics. Please see manual of operating procedures for a summary of expected toxicities.

Table 3: Definitions of causality

Relationship	Description	Event Type
**Unrelated**	There is no evidence of any causal relationship	SAE
**Unlikely**	There is little evidence to suggest there is a causal relationship (e.g. the event did not occur within a reasonable time after administration of the trial medication). There is another reasonable explanation for the event (e.g. the patient's clinical condition, other concomitant treatment).	SAE
**Possible**	There is some evidence to suggest a causal relationship (e.g. because the event occurs within a reasonable time after administration of the trial medication). However, the influence of other factors may have contributed to the event (e.g. the patient's clinical condition, other concomitant treatments).	SAR
**Probable**	There is evidence to suggest a causal relationship and the	SAR

	influence of other factors is unlikely.	
**Definitely**	There is clear evidence to suggest a causal relationship and other possible contributing factors can be ruled out.	SAR

(d) Notification

The appropriate Trials Unit should be notified within one working day of the investigator becoming aware of an event that requires expedited reporting. Investigators should notify the appropriate Trials Unit of all SAEs occurring during the trial.

Notification Procedure:

1. The SAE form must be completed by the Investigator (consultant named on the signature list and delegation of responsibilities log who is responsible for the patient's care), with due care being paid to the grading, causality and expectedness of the event as outlined above. In the absence of the responsible investigator the form should be completed and signed by a member of the site trial team. The responsible investigator should subsequently check the SAE form, make changes as appropriate, sign and then re-fax to the appropriate Trials Unit as soon as possible. The initial report shall be followed by detailed, written reports as appropriate.

2. Send the SAE form by fax to the appropriate Trials Unit:

   MRC Clinical Trials Unit **Fax Number: + 44 (0) 20 7670 4814**
   INSERM SC10-US019 **Fax Number: + 33 (0) 1 45 59 51 80**
   PHPT **Fax Number: + 66 (0) 5381 9130**

3. Follow-up of events: Patients must be followed-up until clinical recovery is complete and laboratory results have returned to normal or baseline, or until the event has stabilised. Follow-up should continue after completion of protocol treatment if necessary. Follow-up information should be noted on a further SAE form by ticking the box marked 'follow-up' and faxing to the appropriate Trials Unit as information becomes available. Extra annotated information and/or copies of test results may be provided separately. The patient must be identified by trial number and date of birth only. The patient's name should not be used on any correspondence.

4. Staff at the institution must notify their local research ethics committee of the event (as per the institution's standard local procedure).

10.4 Trials Units' responsibilities

Medically qualified staff at the Trials Units and the Chief Investigator (or a medically qualified delegate) will review all SAE reports received. The causality assessment given by the local Investigator at the hospital cannot be overruled and in the case of disagreement, both opinions will be provided in any subsequent reports.

The MRC Clinical Trials Unit at UCL, INSERM SC10 and PHPT are undertaking the duties of trial sponsor and are responsible for pharmacovigilance in the countries they co-ordinate and are responsible for the reporting of SUSARs and other SARs to the regulatory authorities of countries in which the trial is taking place and the national research ethics committees as appropriate. In certain instances this responsibility will be delegated to a national co-ordinator.

The Trials Units will also keep all investigators informed of any safety issues that arise during the course of the trial.

Trials Units will comply with any further reporting requirements of the countries they co-ordinate.

Figure 1: Safety Reporting Flowchart

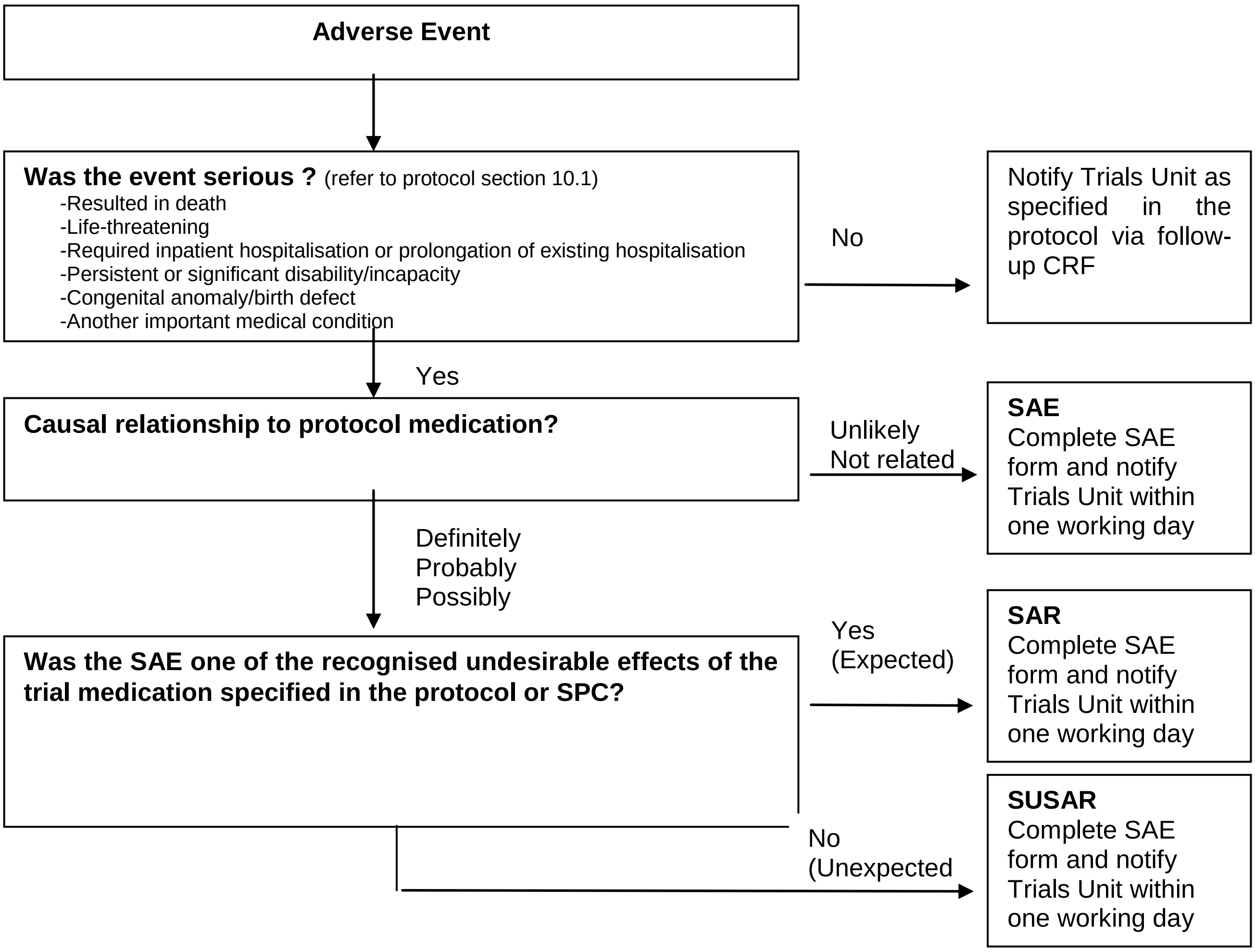

11 STATISTICAL CONSIDERATIONS

11.1 Method of Randomisation

Randomisation (1:1) will be performed centrally by the MRC Clinical Trials Unit, according to a computer-generated randomisation list, using random permuted blocks, stratified by age at randomisation (8-12, 13-17, 18-24 years) and by African vs. non-African country.

11.2 Outcome measures

Pilot Phase Outcome Measure:
- HIV-1 RNA ≥50 copies/ml, reproduced on a repeat test of the same sample off ART on the Monday following SCT at any of week 1, 2, 3 (see section 11.5 for more details).

Primary Outcome:
- HIV-1 RNA ≥50 c/ml (confirmed on a separate sample within 1 week) at any of week 4, 12, 24, 36 or 48 (see section 11.6 for more details)

Secondary Outcomes:
- HIV-1 RNA <50 c/ml at 24 and 48 week.
- Number of HIV mutations present at week 4, 12, 24, 36 or 48 conferring resistance to drugs taken at randomisation or during the trial
- Change in CD4 (absolute and percentage) from randomisation to 24 and 48 weeks
- Change in ART (defined as any change from the ART regimen at randomisation)
- Grade 3 or 4 clinical and laboratory adverse events.
- ART treatment modifying adverse events (all grades).
- New CDC stage B or C diagnosis or death
- Changes in fasting glucose, cholesterol, triglycerides, LDL, HDL and VLDL levels through 48 weeks
- Adherence, acceptability, and quality of life over 48 weeks as assessed by patient completed questionnaires
- HIV-1 RNA ≥50 c/ml (confirmed on a separate sample within 1 week) at any time throughout follow up.
- HIV-1 RNA <50 c/ml at 96 and 144 weeks.
- Adherence over the duration of the trial, as assessed by patient completed questionnaires.
- Change in CD4 (absolute and percentage) from randomisation to 96 and 144 weeks.

11.3 Sample size

This phase II/III trial will enrol a minimum of 160 young people, at least 80 per arm.

Assuming 90% of young people in the continuous ART arm and in the SCT arm maintain HIV-1 RNA <50 c/ml to week 48, 155 young people will provide at least 80% power to exclude a difference of 12% between the two arms (i.e. to exclude suppression rates of less than 78% in the SCT arm) (one-sided alpha = 0.05) [37]. A minimum of **160 (80 per arm) young people will be enrolled** to allow for loss to follow-up (in previous PENTA trials loss to follow-up has been less than 3%).

The power calculations are based on the assumption that 90% of patients in the continuous ART arm will remain virally suppressed to week 48. Any decrease in this percentage is likely to under

power the study and could lead to an equivocal result, as could other changes to the assumptions. The table below gives an indication of the power to exclude a difference of 12% between the two arms for different sample sizes and levels of suppression in the continuous ART arm:

Total sample size	Power by level of suppression in control arm					
	90% suppressed	89% suppressed	88% suppressed	87% suppressed	86% suppressed	85% suppressed
160	80	77	74	72	69	67
170	82	79	76	74	72	69
180	84	81	79	76	74	72
190	86	83	81	78	75	74
200	87	85	82	80	78	76
210	89	86	84	82	79	77
220	90	88	85	83	81	79

A sample size of 220 participants would increase the power of the study by at least 10% for varying levels of suppression. The TSC and IDMC therefore agreed that the study should remain open to randomisation until the end of the defined recruitment period even should the total number of participants enrolled exceed 160, as this would enhance the power of the study as indicated in the table above. The total recruitment target should be at least 160 but not exceed 220 participants.

The trial is not formally powered to detect differences between continuous ART and SCT, but to exclude substantial virological disadvantages (i.e. HIV-1 RNA suppression rates (<50 c/ml) of less than 78%) to the SCT arm by following a 5 days on, 2 day off strategy i.e. a non-inferiority trial.

A non-inferiority margin of 12% was chosen to represent a clinically acceptable difference in the rate of virological suppression <50 c/ml between the two arms, and to allow the trial to be adequately powered and feasible to conduct based on estimates of available young people followed in PENTA centres.

11.4 Interim analyses

The trial will be reviewed by the PENTA Independent Data Monitoring Committee (IDMC). No member of the PENTA or BREATHER Steering Committees or any clinician (investigator) responsible for the clinical care of trial participants may be a member of the IDMC. The IDMC will review the trial in all aspects, including the number of participants to be recruited. The IDMC reviewed the data from the pilot phase and identified no safety concerns and recommended recruitment continue. It will continue to review data at regular intervals (approximately every 6 months), in strict confidence, considering the findings from other relevant studies, and will advise the TSC. During the main phase of the trial they will review the trial in all aspects including data from both arms. The IDMC should inform the Chair of the TSC if, in their view, the results provide either:

(a) unequivocal evidence* which is likely to convince a broad range of HIV clinicians, including the study investigators, that, one of the two treatment strategies (Continuous Therapy or Short-Cycle Therapy) is performing poorly for all participants or for a particular category of participants, and there is a reasonable expectation that this new evidence will materially influence patient management;

or

(b) good evidence* that Continuous Therapy is superior to Short-Cycle Therapy in terms of the primary outcome and the non-inferiority of Short Cycle Therapy is extremely unlikely to be demonstrated with continued enrolment and/or follow-up.

* The criteria for the strength of evidence cannot be defined precisely and is left to the judgement of the IDMC. However, as an example, if in an interim analysis the 99% confidence interval for the hazard ratio for the primary outcome excluded 1, this may be considered as providing good evidence of a difference in risk between the two groups. If, the 99.9% confidence interval also excluded 1, this may be regarded as providing unequivocal evidence of a difference between the two groups.

11.5 Pilot phase

Once the 15th participant randomised to the SCT arm has been recruited to the pilot phase, recruitment and randomisation will be put on hold in both arms.

If full data (weekly viral loads for first 3 weeks) are not obtained on all young people in the SCT arm of the pilot phase, further participants will be enrolled in the pilot phase. This will be assessed in real-time rather than waiting until the end of the pilot phase.

The IDMC will review the results of the pilot phase and consider the findings in conjunction with other relevant studies. Formal stopping criteria for the pilot phase, e.g. the Haybittle-Peto rule, will not be used as the pilot phase is not assessing the efficacy of the SCT strategy compared to the continuous therapy. The aim of the pilot phase is to make sure the SCT strategy does not result in a high proportion of young people with increased viral loads (≥50 c/ml) off ART in the first few weeks. Therefore the following situation seen in the SCT arm would cause concern regarding continued enrolment:

- 5 (or more) participants who have an HIV-1 RNA ≥50 c/ml (validated by a HIV-1 RNA ≥50 c/ml on the same sample) at weeks 1, 2 or 3 would following weekend interruption and before restarting ART give evidence that viral load suppression rates on an SCT strategy in general would be <90% (10/15 suppressed = 66%, 95% exact CI=38%, 88%). This would be considered an unacceptable rate of viral load suppression.

If 4 (or less participants) have an HIV-1 RNA ≥50 c/ml (validated by a HIV-1 RNA ≥50 c/ml on the same sample) at weeks 1, 2 or 3 would give some evidence that viral load suppression rates on an SCT strategy would be >90% (11/15 suppressed = 73%. 95% exact CI=45%, 92%) and therefore would provide reassurance to continue enrolment.

There will not be any further recruitment until this review has taken place. Following the review of the pilot phase, the IDMC will make recommendations to the TSC, who will decide whether to continue the trial. If the decision is to continue the trial, recruitment will re-commence.

11.6 Analysis plan

Intention-to-treat analyses will be performed on all randomised participants. Statistical methods include:

- Descriptive statistics for the summary of baseline characteristics
- Fishers exact test and logistic regression models for the analysis of binary outcome variables
- Analysis of variance and linear regression models for the analysis of continuous outcome variables, adjusting for baseline
- Poisson regression for the analysis of the incidence rate of clinical/adverse events
- Log rank test and proportional hazards regression models for the analysis of time to event variables

The incidence rate of clinical/adverse events will be summarised by body system and randomised arm. First events (in terms of time to first event) and all events will be considered.

Major resistance mutations will be defined by the current IAS-USA list.

The primary outcome for the main study is HIV-1 RNA ≥50 c/ml (confirmed on a separate sample within 1 week) at any of week 4, 12, 24, 36 or 48. This outcome only considers HIV-1 RNA measurements at these time points due to the difference in viral load monitoring in the pilot phase and the main trial. However if a young person enrolled in the pilot phase has an HIV-1 RNA ≥50 copies/ml at weeks 1, 2 or 3 (reproducible on the same sample) or at week 8 (confirmed on a separate sample within 1 week) they will be considered as reaching the primary outcome at week 4 and 12 respectively. All participants will be analysed in the arm to which they were randomised (intention to treat).

A full Statistical Analysis Plan (SAP) will be developed as a separate document.

12 TRIAL MONITORING

12.1 Risk assessment

A risk assessment has been performed to assess the impact of trial participation on the rights and safety of patients, and the reliability of trial results. This has guided the development of procedures in the trial with respect to informed consent, confidentiality and trial monitoring.

12.2 Monitoring at the Trials Units

Data received at each of the Trials Units will be checked for missing or unusual values (range checks) and checked for consistency within participants over time. If any such problems are identified, a missing data report of the problematic data will be sent to the local site by password protected e-mail for checking and confirmation or correction, as appropriate – any data which are changed should be crossed through with a single line and initialled. The amended data should be returned to the appropriate Trials Unit and the amended data should also be filed in the notes at site. The Trials Units will send reminders to sites under their management for any overdue and missing data.

12.3 Clinical Site Monitoring

The agreement with each investigator will include permission for trial-related monitoring, audits, ethics committee review and regulatory inspections by providing direct access to source data/documents. Consent from parents/carers and young people, if appropriate, for direct access to data will also be obtained.

Personal medical data may be reviewed at clinical centres by properly authorised individuals from the Trials Units as part of monitoring and/or audit of the trial, but such information will be treated as strictly confidential and will in no circumstances be made publicly available. All clinical centres will be visited at least once during the trial and following data will be validated from source documents:

- eligibility and signed consent
- clinical disease progression to new CDC C event or death
- HIV-1 RNA viral loads ≥50 c/ml (primary end point only)
- a random sample of CD4 measurements
- a random sample of laboratory results
- a random sample of original records of antiretroviral prescriptions (with batch numbers)
- a random sample of clinical data

- all original records of antiretroviral prescriptions (with batch numbers) for all young people participating in the qualitative substudy.

12.4 Confidentiality

The young person's anonymity will be maintained. On all CRFs and specimens, participants must not be identified by their names. The investigator will be asked to keep a separate confidential Trial Register which matches the participant's trial number with their name and should be maintained by the investigator in strict confidence and kept for 15 years.

13 ETHICAL CONSIDERATIONS AND APPROVAL

13.1 Ethical approval

The BREATHER trial will be conducted in full conformance with the principles of the current version of the Declaration of Helsinki and with the local laws and regulations concerning clinical trials.

At each site, one clinician (Principal Investigator) will take on overall responsibility for the conduct of the trial.

The protocol, the informed consent documents and the questionnaires will be formally approved by the regulatory authority, the necessary ethics committees in each participating country as well as local research and development department at each clinical site. Sites will then be supplied with the required documentation for submission of local approvals.

Before each site can start the trial, the Principal Investigator at each site must send a signed copy of the Investigator's Agreement to Participate, agreeing to the Terms and Conditions of participation in the trial.

Parental/carer and/or the participant's written consent/assent will be obtained where appropriate (Appendix 2).

All information collected during the BREATHER trial will be confidential. Names will not be used.

The right of the patient to refuse to participate in the trial without giving reasons must be respected. After the patient has entered the trial, the clinician must remain free to give alternative treatment to that specified in the protocol, at any stage, if he/she feels it to be in the best interest of the patient. However, the reason for doing so should be recorded and the patient will remain within the trial for the purpose of follow-up and data analysis according to the treatment strategy to which they have been allocated. Similarly, the patient must remain free to withdraw at any time from the protocol treatment and trial follow-up without giving reasons and without prejudicing his/her further treatment. Patients who become 18 years of age (or earlier if required by national requirements) whilst on the trial will be required to sign a consent form if one has not already been signed by them.

13.2 Ethical considerations

- The main risk associated with undertaking this study is that the strategy will prove ineffective in maintaining viral suppression and that viral evolution with the emergence of resistance will occur. If a raised viral load is confirmed on repeat testing (to be done within

1 week) then the participant will be placed back on continuous therapy. Participants enrolled into the pilot phase should only be seen at centres where HIV-1 viral load test results are available within 2 days.

- There is the small potential risk that resistance developed during the study might render a drug or class of drugs ineffective which could in turn limit future therapeutic options. Resistance testing should be performed on all participants who lose virological suppression in either arm at the point of loss of suppression (≥50 c/ml), and any subsequent samples with HIV-1 RNA ≥1000 c/ml.
- An additional risk is that young people, who have prior to enrolment in the study been fully adherent, might extend the permitted very short interruptions and that overall adherence could decline.
- Additional visits and blood tests are required for the trial, with intensity at the enrolment stage. If the participant is included in the SCT pilot phase, they will need to attend extra phlebotomy visits for the first 3 weeks of the trial. Those randomised to SCT arm in the main phase of trial will have only 1 viral load taken at the first week; however there are clinic visits at week 4 and 12 before resuming 3 monthly visits for all participants.
- For those participants requiring local anaesthetic cream this can be applied prior to taking blood.
- Centres will only be approached to join the trial if <50 c/ml cut-off viral load assay is routinely used.
- This is a randomised trial and participants will not be able to choose which treatment approach they follow.
- This is a novel approach to ART in young people, and it is not known yet whether this is a safe strategy.

14 REGULATORY ISSUES

This trial has been registered with competent authorities in participating countries and has been granted a Clinical Trial Authorisation (CTA). The CTA reference is 2009-012947-40.

15 INDEMNITY

In consideration of the agreement by the Principal Investigator at each centre to supervise the trial, the PENTA Foundation undertakes to indemnify the Principal Investigator at each PENTA centre and the institutions which participate in the trial and their employees and agents in respect of any claims made against them by any third party which arises out of or as a result of the supervision or conduct of the trial (including any claim arising in respect of the technical procedures described in the protocol which participants would not have been exposed but for their participation in the trial). Full details of the Indemnity agreement are given in a separate document. Cover against claims arising from medical negligence is not included.

For UK Sites only
The co-sponsor of the trial in the UK is the Medical Research Council (MRC). University College London is now the employer of staff at the MRC Clinical Trials Unit (now MRC CTU at UCL), the co-ordinator of the trial. University College London holds insurance against claims from participants for injury caused by their participation in this clinical trial. Participants may be able to claim compensation if they can prove that UCL has been negligent. However, as this clinical trial is being carried out in a hospital, the hospital continues to have a duty of care to the participant of the clinical trial. University College London does not accept liability for any breach in the hospital's duty of care, or any negligence on the part of hospital employees. This applies whether the hospital is

an NHS Trust or otherwise. Participants may also be able to claim compensation for injury caused by participation in this clinical trial without the need to prove negligence on the part of University College London or another party. Participants who sustain injury and wish to make a claim for compensation should do so in writing in the first instance to the Chief Investigator, who will pass the claim to the University College London's Insurers, via the University College London's office. Hospitals selected to participate in this clinical trial must provide clinical negligence insurance cover for harm caused by their employees and a copy of the relevant insurance policy or summary can be provided on request.

16 FINANCE

Financial research support will be provided to the clinical centres for additional visits and sample collection for routine visits, and will be provided on a national level. Drug will continue to be supplied by each participating centre.

Where approved by ethics, a small sum in vouchers will be given after attending each of the 3 weekly visits during the pilot phase, SCT arm only (ie a total of £30 maximum for the 3 weeks) to compensate for the time required to attend clinic. All related travel expenses for visits additional to routine visits will also be provided.

17 DISSEMINATION

17.1 Publication

The BREATHER TMG will be responsible for preparing the manuscript for rapid publication. High priority will be given to this and it would be anticipated that a report would be completed within six months of a decision to stop the trial. The final publication will require the approval of the BREATHER TMG and TSC. No other publications, including all or any part of the results, either written or verbal, will be made before the definitive manuscript has been agreed and accepted for publication without the prior approval of the BREATHER TMG and TSC. Individual clinicians must not publish data concerning their patients that are directly relevant to questions posed by the study until the Trial Management Group has published its report.

Responsibility for data analysis and publication for BREATHER will reside within the PENTA network and be governed by PENTA policies.

The trial will be registered with an internationally accepted clinical trials register (controlled-trials.com) and the unique identification number (ISRCTN) allocated to this trial will be attached to all publications resulting from this trial.

17.2 Feedback to participants

A PENTA newsletter will be circulated to sites on an annual basis informing all patients of trials currently recruiting and in follow-up.

Participants will be notified of the date of the end of the trial by letter, given by their clinic team.

After the specific trial results are available, an information sheet in lay terms will be provided for every trial participant to explain the results of the trial. This will be distributed to the Principal Investigators at each site so that the clinician can go through it with the young person and/or parent/carer to explain the outcome of the trial.

18 PROTOCOL AMENDMENTS

Changes to protocol version 1.9 creating version 1.10 May 2015
Major changes:
Section 1.4, 4.2 and 11.2: The following additional secondary outcome measures were added:

- HIV-1 RNA ≥50 c/ml (confirmed on a separate sample within 1 week) at any time throughout follow up.
- HIV-1 RNA <50 c/ml at 96 and 144 weeks.
- Adherence over the duration of the trial, as assessed by patient completed questionnaires.
- Change in CD4 (absolute and percentage) from randomisation to 96 and 144 weeks.

Section 1.13 and 8.10: The participants will not complete acceptability questionnaires during long term follow up.

Sections 1.5, 7.4:

- The timeframe for 3 occurrences of isolated viral load requiring a return to continuous ART from SCT was amended from the lifetime of the study to within 12 months. Viral load results with a higher cut-off than 50 c/ml e.g. < 80c/ml (due to technical issues, diluted samples or low volume) should not be considered a blip, but participants should still be instructed to return to clinic for a re-test to confirm their viral load is <50c/ml.

Section 1.13, 8.10: These sections have been updated to include an additional plasma storage sample to be taken 48 weeks after the final main study visit. At sites where this can be done, cells are also to be stored at this point and at the final visit.

Appendix 12: Sample patient information sheets, informed consent forms and assent forms were added for the non trial participants that will take part in the focus groups as part of the qualitative substudy.

Appendix 21: Sample patient information sheets, informed consent forms and assent forms for additional samples during long term follow up.

Minor changes:

General information: Updates made to main contacts: MRC CTU at UCL contact details

Changes to protocol version 1.7 creating version 1.9 1st April 2014 (the changes made from version 1.7 to 1.8 12 March 2014 are as outlined below except that it was planned that adherence questionnaires would be done once a year during the long term follow up. It was subsequently decided that adherence questionnaires should be done every 12-16 weeks and acceptability questionnaires at re-starting continuous ART or at the end of the study. It was also decided that a CRF should be completed every 12-16 weeks rather than once a year during the long term follow up, and that tanner scales and routine FBC and biochemistry results did not need to be collected. Flowsheet 1.13, section 8.10 and the new information sheets and consent forms in appendices 17-20 were updated to reflect this. Version 1.8 was approved by the Irish Medicines Board before this update was made, but not reviewed or approved in the other countries)

Major changes:

General Information, Abbreviations, sections 3.2, 10.4, 15, appendix 16: The MRC CTU became part of UCL on 1st August 2013. All MRC CTU employees are now employed by UCL and UCL is responsible for providing indemnity for sites in the UK.
General Information, Abbreviations, section 3.2, 10.3, appendix 16: INSERM-SC10 became INSERM SC10-US019
Section 1.6, 1.7.2, 7.12, 8.11.1, appendix 12: End of trial definition changed from once last participant enrolled has been followed for 48 weeks (now defined as completion of main trial) to once all participants have had their final long term follow up visits
Section 1.6, 1.13, 4.1, 8, 8.10, 8.11.1, appendix 15: The TSC recommended that participants are followed for 2 years after completion of the main trial (long term follow up). A flowsheet has been generated for this long term follow up including completion of adherence questionnaires every 12-16 weeks, completion of acceptability questionnaires at re-starting continuous ART or at the end of the study, and storage of a plasma sample at the final visit for future HIV-related tests
Section 1.6, 1.13, 7.12, 8.10, appendix 15: Management of HIV-1 RNA viral load should continue as specified in appendix 15 if the participant continues follow up in the trial after the main trial is completed
Section 7.12, appendix 12: The TSC recommended that young people be allowed to stay on SCT between the main trial completion and publication of the results provided that they are virologically suppressed, the clinician and family agree, and 12-16 weekly viral load monitoring can be performed

Appendices 17-20: New information sheets added for long term follow up in SCT (appendix 17) and CT (appendix 19) arms for parents/carers, young adults, young people, children and children taking medicines long term (unaware of diagnosis), and new consent forms for parents/carers and young adults and assent forms for young people/children for SCT (appendix 18) and CT (appendix 20) arms

Minor changes:

General Information: Clarification that funding by PENTA from current EU grant is available until 2015
General information, section 3.2: Updates made to participating centres and MRC CTU contact details
Sections 2.6, 8.1, 8.4, 8.11.2, 10.3, appendix 11: minor clarifications to text
Section 10.1, 10.2: Clarification that pregnancies are notable events and should be reported on an SAE form, and that if a participant on the SCT becomes pregnant they should return to CT
Section 8.10, 10.2: Safety reporting to continue as per protocol for entire duration of follow up within the trial
Appendix 11: Proviral DNA will not be quantified in young people in the pilot phase of the SCT arm at weeks 1, 2, and 3 as PBMCs are required for this but only plasma was collected at these visits. Low level viral loads will be done to <10 c/ml rather than <3 c/ml as this is what is currently feasible
Appendix 12 – qualitative sub study consent forms – clarification that there will be 2-3 interviews (rather than just two)
Appendix 16: Other updates to contact details (non-MRC CTU)

Changes to protocol version 1.6 creating version 1.7 24 April 2013

Major changes:

Sections 1.3, 1.8, 4.1, 5.5, 11.3, Appendix 1, Appendix 3: Sample size updated to a minimum of 160 participants
The TSC and IDMC supported a proposal to enrol as many young people as possible in the time available, even if this exceeds 160. They agreed that this would enhance the power of the study and allow continuing collaboration with new centres.
Section 1.6, 4.1: Recruitment period updated to 27 months, i.e. up until end of June 2013
Section 11.3: Justification for updating sample size to a minimum of 160 and a maximum of 220 participants added
Section 2.6, 8.5, 13.2: Minor re-wording to clarify that not all resistance tests will need to be done centrally and may be performed locally where feasible.
Appendix 11: Clarification that resistance testing will be performed locally and that if this is not possible, or the amino acid sequence cannot be provided in a FASTA file format, they will be performed centrally.
The TMG agreed that centralised viral load testing on samples ≥50 c/ml would introduce non-random bias and that the variation between assays that exists in reality should not systematically bias randomised comparisons.
Section 11.4: Stopping criteria updated
Appendix 12: Inclusion of data collection with non-trial participants; focus groups with non-trial participants and interviews with carers. These and the associated information sheets and consent forms will have ethical approval sought from LSHTM)

Minor changes:

Section 1.6: Clarification that the end of trial will be once the last participant randomised has completed 48 weeks of follow-up.
Section 1.7.3: Minor re-wording
Section 1.8: Clarification that participants that have completed 48 weeks of follow-up will be seen every 12 weeks thereafter until the end of the trial.
Section 4.3: A scan of the original CRF may be sent to the Trials Unit for data entry for some centres
Section 5.5: There will be single centre in the Ukraine, therefore wording updated
Section 6.1.2: Clarification that RA/RO phenotypes to be collected if available
Section 7.5.2: Minor re-wording
Section 11.1: Update to 24 years for 18-21 year age strata (not updated in protocol version 1.6 in error)
Section 11.4: Updated wording regarding IDMC review of pilot and outcome
Section 18: Clarification in Changes to protocol version 1.5 creating version 1.6 29 June 2012 that the Qualitative Substudy information sheets were updated as described (and not the main trial information sheets)
Appendix 12: Clarifications that Dr Sarah Bernays will also be managing the qualitative study, trial participants must be aged 10-24 year olds, three interviews will be conducted where feasible, exploring participants' interest in contributing an audio diary in the latter half of the trial will not necessarily need to be at around 36 weeks, focus groups will be conducted in Uganda (with trial participants), incentives will not be provided for focus groups but food and refreshments will be provided and travel costs reimbursed
Appendix 15: Clarifications added to flow chart for managing viral loads regarding viral load results reported as <XX c/ml where XX>50
Appendix 16: Marc Lallemant changed to Tim Cressey in TMG. Anna Turkova added to TMG.

Changes to protocol version 1.5 creating version 1.6 29 June 2012

Major changes:

Abbreviations and Glossary, sections 1.3, 2.5, 5.1, 9.2, Appendix 12 Qualitative Substudy information sheets for parents/carers, young adults and young people: Upper age limit for young person/people eligible for trial entry increased to 24 years of age

Sections 1.6 and 4.1: Recruitment period updated to 24 months in total

Appendix 12: Addition of USA to Qualitative Substudy

Minor changes:

General Information, sections 3.2, 5.5, Appendix 14 and Appendix 16: Participating centres updated
Sections 4.2 and 11.6: Correction of typing error – young people with a HIV-1 RNA ≥50 copies/ml at week 8 (pilot phase only) must have a repeat test on a separate sample within 1 week
Appendix 15: Clarification that participants in the CT arm should have a repeat VL on a separate sample within 1 week if any VL ≥ 50. Clarification that if a participant enrolled to the pilot phase had a confirmed blip at any of weeks 1, 2 or 3 in the pilot phase, then they would need to return to CT if they have 2 unconfirmed blips in the main phase of the trial.

Changes to protocol version 1.4 creating version 1.5 19th December 2011

Major changes:

Text relating to the pilot phase only now appears in grey; including in the flow charts 1.09, 1.10 and Appendix 15, and has been removed in the Sample Patient Information Sheets (Appendix 1).
Section 2.6 Risks and benefits: The statement 'Safety in the pilot study will be assured before moving to the main trial' has been replaced with 'Data from the pilot phase has been reviewed by the IDMC who identified no safety concerns and recommended that recruitment continue'. This statement has also been added to the Patient Information Sheets in Appendix 1.
Section 6.1.1 Enrolment and consent: Minor re-wording of text removing reference to pilot.

The requirement for local HIV-1 RNA viral load measurement at randomisation (week 0) has been removed affecting: flowsheets in section 1.11 and 1.12, and section 6.2.

Screening visit (section 6.1.2): Previously stated screening should take place no more than four weeks prior to randomisation (week 0) and ideally two weeks before. This has been changed to state that randomisation (week 0) should take place no more than four weeks after screening and ideally as soon as possible after eligibility has been confirmed. This change is also reflected in section 7.3.

For young people randomised to SCT, a comment has been added in sections 1.2, 4.1 and 7.3 to state that if alternative consecutive days to Saturday-Sunday or Friday-Saturday are taken off ART to better suit a particular young person's normal routine then the days should be decided at enrolment and remain constant throughout the study period. This has also been added to the Patient Information Sheets in Appendix 1 stating that young people/parents/carers should discuss this with their nurse/doctor prior to enrolment.

Changes to the inclusion and exclusion criteria (affecting sections 5.1 and 5.2):
Participants can be on a regimen containing NtRTIs (i.e. 2 NRTIs/NtRTIs and EFV). This has been clarified in sections 1.3, 1.8, 5.1, and 7.8.
Previously, young people who had experienced a single viral load >50 but <400 copies/ml in the last 12 months could be enrolled. This has been changed to state that young people who have experienced a single viral load >50 but <1000 copies/ml in the last 12 months can be enrolled

(provided at least 3 measurements <50 copies/ml are available from the last 12 months, including screening).
Participants are no longer required to have started HAART naïve. Previous dual therapy and/or substitution of NRTIs is allowed providing any changes were not for disease progression, immunological or virological failure. The definition of virological failure has been clarified. Previous ART monotherapy (except for the prevention of mother-to-child transmission) has specifically been added as an exclusion criterion.

Appendix 1 – An information sheet for children unaware of their diagnosis has been added
The role of the Research Ethics committee has been modified in the parents/carers, young adult, and young people information sheets to state that their task is to check the study and make sure as far as possible; no harm comes to anyone from being part of the study.

Appendix 2 – Sample Consent Forms: Statement regarding use of anonymised blood samples in consent forms for parent/carers and young adults has been amended to state that they will only be used for ethically approved studies. Assent form for children unaware of diagnosis added

Appendix 5 – Acceptability questionnaire for carers – Weekends off group – Restarting continuous ART or at end of study: Question 5 reworded into two parts (a and b) to ask about the difference for the child before and after the weekend. Question 6 reworded into two parts (a and b) to ask about the difference for the carer before and after the weekend.
Acceptability questionnaire for young people – Weekends off group – Restarting continuous ART or at the end of study: Additional question introduced at beginning to ask who gives the young person their medicines (for consistency with the other questionnaires). Question 3 reworded into two parts (a and b) to ask about the difference for the young person before and after the weekend. Additional question introduced after this (now Q4) to ask about difference before and after the study.

Appendix 12 - Qualitative Substudy – Patient Information Sheets - Wording regarding timing of interviews modified; first interview to take place in early stages of the trial and second to take place towards the end of the trial. Clarification that a third interview may be conducted if feasible and that travel expenses to attend interviews will be reimbursed.
A witness signature section has been added to all the consent forms relating to this substudy in the case that the participant/carer is unable to sign and uses a thumbprint.

Minor changes:

Minor re-wording for clarification: general information; 1.6; 1.7.3; 5.1; 6.1.1; 6.2; Appendix 11
Updates to contact details: general information; Appendix 16

Changes to protocol version 1.3 creating version 1.4 21st January 2010

Major changes:

Prof. Ian Weller, Chair of BREATHER Steering Committee added to authorisation of protocol.
Compliance and funder details changed
Section 1.2, 1.5, 1.8, 1.9 -1.12, 4.1, 4.2, 7.1, 7.2, 7.6 PIS: Addition of viral load measurements and blood stores at weeks 1, 2, 3 and 8 for participants randomised to continuous ART in the pilot phase
Section 1.9-1.12, 8.5: Reduction of timepoints for cell storage

Section 4.2, 11.6: clarification of main trial primary outcome measures for participants enrolled in the pilot phase

Minor changes and corrections:

General information: Reference to EuroCoord added
Flowsheet 1.10: Flowsheet for CT Participants in the Pilot phase added
Appendix 2: Minor re-wording of the consent forms
Appendix 16: IDMC membership added

Changes to protocol version 1.1 creating version 1.3 8th December 2010.

Note: This protocol is named version 1.3 8th December 2010 to avoid confusion with protocol version 1.2 dated 12th August 2010 which was submitted to the Thai Research Ethics Committee in error.

Major changes:

General information: committee membership and medical experts moved to Appendix 16
Section 1.9, 1.10, 1.11: Tanner stage examination moved from screening to randomisation visit. Calcium and phosphate now only at baseline and annually thereafter. Biochemistry to be carried out as per local practice.
Section 3.2, Uganda and Romania added to list of participating countries.
Section 5.2 : additions to exclusion criteria: creatinine, AST or ALT of grade 3 or above at screening; NVP or boosted PI regimen.
Section 5.3, specified that siblings can be allocated into the same arm.
Section 7.3, clarification that participants in pilot can change days off from Saturday/Sunday to Friday/Saturday, after the pilot if they wish; removal of specification that morning ART must be not be taken.
Section 8.2, collection of ethnic origin justified and reference [39] added.
Section 8.7: clarification of use of diary to provide information on adherence.
Appendix 1: Deletion of reference to text-back; clarification of where information is country/substudy specific.
Appendix 2: Addition of consent to receiving text messages, where possible.
Appendix 4 and 5. Adherence and Acceptability questionnaires revised.
Appendix 7. An alternative design for the participant diary added.
Appendix 9. Toxicity grading tables updated with August 2009 revision.

Contacts and references updated.

Minor changes made for clarification; Page 1, Sections 1.2, 1.3, 1.5, 1.9, 1.10, 1.11, 2.2.1, 2.4, 5.1, 5.2, 6.2.1, 7.1, 8, 8.2, 8.4, 8.5, 8.7, 8.8, 8.10.1, 10.4, 11.4, Appendices 14 and 15.

Changes to protocol version 1.0 creating **version 1.1 12th April 2010:**

Major changes:
1) Viral load cut off of 10,000 taken out of section 1.8, 7.2 and patient information sheets.
2) For pilot phase, option of fri/sat off ART and the attending phlebotomy visit on a Tuesday has been taken out so that the viral load is taken before recommencing ART (i.e Sat/sun off ART, viral load taken on Monday before ART recommences).
3) Clarification of how the young people in the pilot phase will be handled in terms of the primary outcome.
4) Week 1 phlebotomy visit in main trial taken out (flowsheet 1.10 amended)
5) Centralised viral loads to be measured at end of trial (section 8.5 and appendix 11).
6) Change of lipodystrophy assessment from screening visits to week 0 (flowsheets 1.9-1.11, section 8.2).

Changes to structure and minor changes to text:

7) Text about pilot phase and main trial specific requirements taken out of sections, 1.8, 4.2, 4.3, 6.2.2 and 6.2.3 and added to sections 1.2, 1.4, 1.5, 4.1, 4.2 and section 7 (7.1-7.5).
8) Thailand HIV-NAT to take part in adherence MEMS cap sub study (section 1.7.3).
9) Changes to wording of inclusion criteria (items 3 and 4).
10) Procedures for assessing efficacy text moved from section 8.9 to 4.3.
11) Clarification in text to sections 8.7.and 8.8 to state that both careers and participants should complete questionnaires, where applicable.
12) Clarification that week 8 visit is for the pilot phase SCT arm only, sections 1.5 and 8.5 have been amended).
13) Abbreviations and Glossary updated.
14) Contacts updated.

19 REFERENCES

1) Gibb DM, Duong T, Tookey PA *et al*. Decline in mortality, AIDS, and hospital admissions in perinatally HIV-1 infected children in the United Kingdom and Ireland. *BMJ* 2003; 327(7422):1019.
2) Working Group on Antiretroviral Therapy and Medical Management of HIV-Infected Children. Guidelines for the use of antiretroviral agents in pediatric HIV infection. Rockville: National Institutes of Health 2008.
3) Welch S, Gibb D, Lyall EGH, Sharland M. PENTA guidelines for the use of antiretroviral therapy in paediatric infection - 2009 accepted for publication in HIV medicine. Available from: www.pentatrials.org
4) World Health Organization. Antiretroviral therapy for HIV infection in infants and children : towards universal access : recommendations for a public health approach. Geneva: World Health Organization; 2007.
5) Violari A, Cotton MF, Gibb DM, Babiker AG, Steyn J, Madhi SA, et al. Early antiretroviral therapy and mortality among HIV-infected infants. The New England Journal of Medicine. 2008 Nov 20;359(21):2233-44.
6) Foster C, Judd A, Tookey P, Tudor-Williams G, Dunn D, Shingadia D, et al. Young people in the UK and Ireland with perinatally acquired HIV: the paediatric legacy for adult services. AIDS Patient Care and STDs 2008.
7) Rianthavon P, Ettenger RB. Medication non-adherence in the adolescent renal transplant recipient: a clinician's viewpoint. *Pediatr Transplant* 2005; 9(3):398-407.
8) Smith BA, Shuchman M. Problem of nonadherence in chronically ill adolescents: strategies for assessment and intervention. *Curr Opin Pediatr* 2005; 17(5):613-8.
9) Fiese BH, Everhart RS. Medical adherence and childhood chronic illness: family and daily management skills and emotional climate as emerging contributors. *Curr Opin Pediatr* 2006; 18(5):551-7.
10) Paterson DL, Swindells S, Mohr *et al*. Adherence to protease inhibitor therapy and outcomes in patients with HIV infection. *Ann Intern Med* 2000; 133(1):21-30.
11) Nachega JB, Hislop M, Dowdy DW *et al*. Adherence to nonnucleoside reverse transcriptase inhibitor-based HIV therapy and virologic outcomes. *Ann Intern Med* 2007; 146(8):564-73.
12) Bangsberg DR, Kroetz DL, Deeks SG. Adherence-resistance relationships to combination HIV antiretroviral therapy. *Curr HIV/AIDS Rep* 2007; 4(2):65-72.
13) Wagels T, Amiet R, Battegay M *et al*. Predictive value of adherence in patients starting highly active antiretroviral treatment for HIV infection. *Swiss Med Wkly* 2004; 134(45-46):678-80.
14) Casado JL, Sabido R, Perez-Elias MJ *et al*. Percentage of adherence correlates with the risk of protease inhibitor (PI) treatment failure in HIV-infected patients. *Antivir Ther* 1999; 4(3):157-61.
15) Chesney MA, Morin M, Sherr L. Adherence to HIV combination therapy. *Soc Sci Med* 2000; 50(11):1599-605.
16) Deeks SG. Determinants of virological response to antiretroviral therapy: implications for long-term strategies. Clin Infect Dis 2000; Suppl 2:S177-84.
17) Reddington C, Cohen J, Baldillo A *et al*. Adherence to medication regimens among children with human immunodeficiency virus infection. *Pediatr Infect Dis J* 2000; 19(12):1148-53.
18) Murphy DA, Belzer M, Durako SJ *et al*. Longitudinal antiretroviral adherence among adolescents infected with human immunodeficiency virus. *Arch Pediatr Adolesc Med* 2005; 159(8):764-70.
19) Martin S, Elliott-DeSorbo DK, Wolters PL *et al*. Patient, caregiver and regimen characteristics associated with adherence to highly active antiretroviral therapy among HIV-infected children and adolescents. *Pediatr Infect Dis* 2007; 26(1):61-7.
20) Gross R, Yip B, Lo Re V 3rd *et al*. A simple dynamic measure of antiretroviral therapy adherence predicts failure to maintain HIV-1 suppression. *J infect Dis* 2006; 194(8):1108-14.

21) Gibb DM, Goodall RL, Giacomet V *et al*. Adherence to prescribed antiretroviral therapy in human immunodeficiency virus-infected children in the PENTA 5 trial. *Pediatr Infect Dis J* 2003; 22(1):56-62.
22) Gibb D, Compagnucci A, Green H, Lallemant M, Saidi Y, Ngo-Giang-Huong N, et al. Treatment interruption in children with chronic HIV-infection: the results of the Paediatric European Network for Treatment of AIDS (PENTA) 11 trial Ninth International Congress on Drug Therapy in HIV Infection. Glasgow, UK 2008.
23) Strategies for Management of Antiretroviral therapy (SMART) Study Group: El-Sadr WM, Lundgren JD, Neaton JD *et al*. CD4+ count-guided interruption of antiretroviral treatment. *N Engl J Med* 2006;355(22):2283-96.
24) Jacobson JM, Bucy RP, Spritzler J *et al*. Evidence That Intermittent Structured Treatment Interruption, but Not Immunization with ALVAC-HIV vCP1452, Promotes Host Control of HIV Replication: The Results of AIDS Clinical Trials Group 5068. *JID* 2006; 194:623-632.
25) Ananworanich J, Nuesch R, Le Braz M *et al*. Failures of 1 week on, 1 week off antiretroviral therapy in a randomized trial. *AIDS* 2003; 17(15):F33-7.
26) Caridello PG, Hassink E, Ananworanich J *et al*. A prospective, randomized trial of structured treatment interruption for patients with chronic HIV type 1 infection. *Clin Infect Dis* 2005; 40(4):594-600.
27) Smith RJ. Adherence to antiretroviral HIV drugs: how many doses can you miss before resistance emerges? *Proc Biol Sci* 2006; 273(1586):617-24.
28) Cohen C J et al. Pilot Study of a novel short-interruption strategy: 48 week results of the five-days-on, two days off (FOTO) study. HIV Clin Trials 2007 Jan-Feb; 8(1): 19-23.
29) Cohen C, Colson A, Pierone G, DeJesus E, Kinder F, Elion R, et al. The FOTO study: 24-week results support the safety of a 2-day break on efavirenz-based antiretroviral therapy. Journal of the International AIDS Society. 2008;11
30) SJ Reynolds, C Kityo, S Ssali *et al*. A Randomized, Controlled, Non-inferiority Trial of Two Short Cycle Intermittent Regimens Compared to Continuous Antiretroviral Therapy for the Treatment of Chronic HIV Infection in Uganda. Presented at: 48th Interscience Conference on Antimicrobial Agents and Chemotherapy (ICAAC); October 25-28, 2008; Washington, USA. Abstract H-1250.
31) Rudy B, Sleasman J, Kapogiannis B *et al*. Short-Cycle Therapy in Adolescents after Continuous Therapy with Established Viral Suppression: The Impact on Viral Load Suppression. AIDS Research and Human Retroviruses. 2009 Jun;25(6):555-61.
32) Taylor S, Boffito M, Khoo S *et al*. Stopping antiretroviral therapy. AIDS 2007; 21:1673-1682.
33) Frampton JE, Croom KF. Efavirenz/emtricitabine/tenofovir disoproxil fumarate: triple combination tablet. *Drugs* 2006;66:1501-1512.
34) Harrigan PR, Whaley M and Montaner JSG. Rate of HIV-1 RNA rebound upon stopping antiretroviral therapy. *AIDS Fast Track* 1999; 13: F59-F62.
35) Cressey TR, Green H, Khoo S, Treluyer JM, Compagnucci A, Saidi Y, et al. Plasma drug concentrations and virologic evaluations after stopping treatment with nonnucleoside reverse-transcriptase inhibitors in HIV type 1-infected children. Clin Infect Dis. 2008 May 15;46(10):1601-8.
36) Dorrell J. Being Young and HIV Positive: The experiences of young people living with HIV since birth: a research study in progress. Alive and kicking: growing up with HIV. A National Conference held by the Children &Young People HIV Network 2007 Friday 16 November The British Library Conference Centre, London 2007.
37) Machin D, Campbell M, Fayes P *et al*. Sample sizes tables for clinical studies (second edition). Blackwell Science 1997; Chapter 5, equation 5.7, p 101.
38) Lee, KJ, Shingadia D, Pillay D, Walker AS, Riordan A, Menson E, Duong T, Tudor-Williams G, Gibb DM on behalf of the Collaborative HIV Paediatric Study (CHIPS) Steering Committee. Transient viral load increases in HIV-infected children in the UK and Ireland: what do they mean? Antiviral Therapy 2007; 12(6): 949-956.

39) Stöhr W, Back D, Dunn D, Sabin C, Winston A, Gilson R, Pillay D, Hill T, Ainsworth J, Pozniak A, Leen C, Bansi L, Fisher M, Orkin C, Anderson J, Johnson M, Easterbrook P, Gibbons S, Khoo S. Factors influencing efavarinz and nevaripine plasma concentration: effect of ethnicity, weight and co-medication. Antiviral Therapy 2008; 13(5):675-85.

20 APPENDICES

APPENDIX 1: SAMPLE PATIENT INFORMATION SHEETS

I. Parents/carers
II. Young adults (16 years and over)
III. Young people (11 to 15 years)
IV. Children (8 to 10 years)
V. Children (unaware of diagnosis) – for use at local care team's discretion

All to be printed on local headed paper, include page numbers and include local requirements

Please use the following guidelines to amend the sections in italics on the sheets:

(*) Country specific,
(**) Only relevant if participating in MEMS Cap sub-study.

BREATHER
Study Title: Short-Cycle Therapy (SCT) (5 days on / 2 days off) in young people with chronic HIV-infection

AN INFORMATION SHEET FOR PARENTS/CARERS

We would like to invite you and the child in your care to participate in our research study. Before you decide, it is important for you to understand why we want to carry out this study and what it will mean for you both. Please take your time to read the following information and feel free to discuss it with your friends, relatives and your doctor or clinic nurse. Please ask if you need more information or if anything is not clear. Take as much time as you need to decide whether or not you wish your child to take part.

1) The BREATHER study
The study that your child is being invited to join is called BREATHER. It is organised by PENTA, an independent organisation that concentrates specifically on treatment issues for children and young people infected with HIV. This study will include children and young people from many different countries.

BREATHER is a study that will compare two different ways of taking HIV (antiretroviral) medicines: either taking the medicines every day (as your child is doing now) or taking the medicines during the week for 5 days and then having a break for 2 days (i.e. no antiretroviral medicines) at the weekend. The latter, where the young person has a 2 day break in their treatment each week is called **Short Cycle Therapy (SCT).**

As you will have been told, taking antiretroviral medicines every day without missing a dose is important. This is to stop the virus becoming resistant which it can do if the virus levels in the blood are not low enough. However, some of the medicines that are now available remain in the blood for a longer time, so doctors and scientists are now working towards new ways for people to take the medicines and make it easier for people. **In this study, we will be examining whether it is safe to stop taking medicines for 2 days at weekends and whether they still work as well as when they are taken every day**.

2) Why has my child been chosen?
Your child has been asked to take part in this study because some of the antiretroviral medicines that he/she is taking stay in the blood for a longer time, and because the virus has been undetectable in his/her blood for at least a year. We would like at least 160 young people to take part. If your child joins the study a computer will pick at random which group he/she will be in so it is fair.

The two groups are:
- to continue to take antiretroviral medicines every day without any interruptions **(continuous therapy group).**
- to take medicines during the week but stop taking them at weekends (you and your child can choose whether to stop on either Friday and Saturday or Saturday and Sunday, although you must stick to the days chosen once you have decided which are best for you **(SCT group).** If you/your child would prefer to take 2 consecutive days off during the week other than Friday and Saturday or Saturday and Sunday, please speak to the study nurse or doctor.

Your child has a 50% chance of being in the SCT group (stopping medicines at weekends).

Your child's viral load will be monitored very closely, especially at the beginning of the study. If for any reason, his/her anti-HIV treatment needs to be changed this will be done by their doctor, in the usual way, and will not be affected by the group they are in.

3) Participation
It is totally up to you and your child whether or not he/she takes part in this study. It is your right to decide not to take part if you wish. This will not affect your child's treatment now or in the future in any way. If you do decide to take part we will describe the study to you and go through this information sheet together. We will then give a copy to you. You will then be asked to sign a consent form to show you have understood the information and agreed to take part. **You are free to withdraw your child from the study at any time without giving a reason**. Withdrawing from the study will not affect your child's medical care. We hope, however, that if you do withdraw you would give us a reason and will allow us to continue collecting information from your child's clinical notes. This will help us when we look at the results of the study.

As part of the study, blood samples will be stored for up to 15 years. We would hope that if your child withdrew from the study, you would still allow us to use these samples. If, however, you did not feel that this was appropriate then we will destroy the samples.

4) What will happen to my child if he/she decides to take part?
Visits and duration: All young people who join the study will initially be seen for two clinic visits in the space of one month before they begin the study to make sure it is OK for them to start the study.

To help remember to stop taking medicines at weekends and re-start after the 2 day break, a diary will be given out for completion.
In addition the nurses at your clinic can arrange for text message reminders to be sent to you or your child if you think this may help. ***(*)***

SCT group
Young people enrolled in the SCT group will be asked to come for a clinic visit four weeks after randomisation and then your child's visits to clinic will be every 3 months as usual.

SCT group - viral load testing
If at any time your child's viral load is more than 50 copies per ml they will be asked to provide a second blood sample for repeat testing within 1 week. If the result of the second test is also more than 50 copies per ml, they will be asked to start taking their antiretroviral medicines every day for the rest of the study and will not be able to have any more weekend breaks.

Continuous treatment group: If your child is put in the group continuing to take antiretroviral medicines every day they will have to come for an additional clinic visit 1 month after enrolling in the study. Your child's visits to clinics will then be every 3 months as usual. Children and young people on the continuous treatment group should not stop their anti-HIV medicines at any time.

Both groups: At clinic visits your child's height and weight will be measured, and the doctor will look for signs of any illnesses. The doctor will also monitor the onset of puberty every 6 months (called tanner scales) as puberty can sometimes be delayed by HIV infection.
Blood will be collected and stored at each visit. These will be tested at the end of the study to find out what happens when the medicines are stopped at weekends. We would like your agreement that stored blood samples can be used in future for tests which help us learn more about HIV.

The study will follow each young person until all participants have completed one year of follow up. At this point the study will end and if your child is in the SCT group they will go back to taking medicines every day. However, normal clinic visits will continue for routine monitoring of your child's HIV.

5) Adherence and Acceptability and Quality of Life questionnaires
We are very interested in finding out what children, young people and families feel about the way the study is going and how it is affecting their well-being. Therefore over the course of the study we will ask you and your child to complete questionnaires designed to measure adherence to medication, acceptability of the way medicines are taken and quality of life.

We would like to carry out an additional study in 60 young people in the trial using MEMS (Medication Event Monitoring Systems) caps. This is an electronic device fitted to medication bottles which records the time and date of each opening.
If you and your child agree to take part in this part of the study you will be given a MEMS cap to fit to your medication bottle. Your doctor or nurse will explain how it works and how to use it.
You must only open the MEMS caps to withdraw the medicine at the times you are going to give the dose. You and your child must not open the container at any other time or transfer medications to other containers. Your child will need to take the MEMS bottles with them if they are staying away from home. ***(**)***

6) What are the possible benefits of taking part?
It is hoped that short cycle therapy (SCT) will:

- lead to a less restrictive way of life as your child may be able to stop taking medicines at the weekend.
- lead to fewer long term side-effects from taking antiretroviral medicines for your child.

We cannot promise that participating in the study will directly help your child. For example, if he/she is in the group staying on medicines every day, nothing will change except for some extra clinic visits. However, all the information we get might help children and young people with HIV in the future and may mean your child would also have the opportunity to stop medicines at weekends eventually.

7) What are the possible disadvantages and risks of taking part?

- You might find it harder to give antiretrovirals during the week and then remember to stop them at weekends if your child is in the SCT group.
- Not taking antiretrovirals everyday can lead to resistance. This means that the medicines may not work so well anymore and new medicines will be needed, particularly if you forget to give the medicines during the week when your child should be taking them. It is also important that if your child is in the group that is to continue taking their medicines every day that they take them properly every time.
- Your child will have to visit the clinic more often than normal even if they are in the group that has to continue taking medicines every day.

8) Pregnancy and anti-HIV medicines
Some combinations of anti-HIV medicines might harm an unborn child. If your daughter could become pregnant, she must have a pregnancy test before entering the study and she must use effective contraceptives including condoms or other barrier contraception if she is having sexual intercourse. If your son is having sexual intercourse he must use condoms.

9) Who has reviewed the study?
This study has been looked at by an independent group of people (a Research Ethics Committee). Their task is to check the study and make sure as far as possible; no harm comes to anyone from being part of the study. This study has been reviewed and given favourable opinion by a Research Ethics Committee. A second group (the Independent Data Monitoring Committee) will meet regularly during the trial and will recommend whether the trial should continue as planned or should stop. Information from these meetings will be summarised in the PENTA Newsletter for families involved in PENTA studies. A pilot study with close monitoring of viral load results over the first 3 weeks of stopping medicines at weekends has been completed. The independent data monitoring committee looked at the results of this pilot phase and were happy for the larger study to continue.

10) Confidentiality
All information collected during the BREATHER study will be kept completely confidential and names will not be used. Your child will only be identified using a study number and date of birth. Your child’s hospital notes will only be made available to study staff and confidentiality will be

maintained at all times. If the results of this study are reported or published your child's name will not be used. The study will be conducted in compliance with the Data Protection Act, 1998. Data will be archived for 15 years and only anonymised data will be sent outside the European Economic Area (EEA).

11) What happens at the end of the study?
After the study has ended all young people in the SCT group will go back to taking their medication every day. You and your child's doctor will continue to make decisions about your child's medicines once the study has finished. If stopping medicines at the weekend is found to be a safe and effective way of treating children and young people with HIV who are taking certain antiretrovirals then this may continue to be part of their clinical care. We would also like to continue to collect routine data about your child after the study has ended so we can look at any long term effects of the study.

12) Compensation arrangements
The PENTA Foundation has made arrangements for compensation should your child come to any harm from participating because of being in this study. However, if your child is harmed due to someone's negligence then you may have grounds for legal action for compensation against the hospital where the negligence occurred, but you may have to pay your legal costs.
Your child's GP will be informed of his/her participation in the study with your permission. Your hospital doctor is not receiving any personal payment for carrying out this research.

13) Patient contact
If you have any concerns or other questions about the study or the way it has been carried out, or if your child has an injury or illness during the study, contact either the investigator (Dr. ________________/ PENTA Team *Phone Number here*) or the hospital where the study is being carried out:
Dr ……………………………….
Dr………………………………..
Nurse …………………………

Thank you for taking the time to consider this study. Please ask any questions and let us know if there are things that you do not understand or would like more information about.

BREATHER
Study Title: Short-Cycle Therapy (SCT) (5 days on / 2 days off) in young people with chronic HIV-infection

AN INFORMATION SHEET FOR YOUNG ADULTS

We would like to invite you to participate in our research study. Before you decide, it is important for you to understand why we want to carry out this study and what it will mean for you. Please take your time to read the following information and feel free to discuss it with your friends, relatives and your doctor or clinic nurse. Please ask if you need more information or if anything is not clear. Take as much time as you need to decide whether or not you wish to take part.

1) The BREATHER study
The study that you are being invited to join is called BREATHER. It is organised by PENTA, an independent organisation that concentrates specifically on treatment issues for children and young people infected with HIV. This study will include young people from many different countries.

BREATHER is a study that will compare two different ways of taking HIV (antiretroviral) medicines: either taking the medicines every day (as you are doing now) or taking the medicines during the week for 5 days and then having a rest for 2 days (i.e. no medicine at the weekend). Where you have a short break in your treatment each week is called **Short Cycle Therapy (SCT).**

As you know, taking antiretrovirals every day without missing a dose is important. This is to stop the virus becoming resistant (i.e. the medicines do not work as well as they should). However, some of the medicines that are now available remain in the blood for a longer time, so doctors and scientists are now working towards new ways for people to take the medicines and make it easier for people. **In this study, we will be examining whether it is safe to stop taking medicines for 2 days at weekends and whether they still work as well as when they are taken every day**.

2) Why have I been chosen?
You have been asked to take part in this study because some of the antiretroviral medicines that you are taking stay in the blood for a longer time, and because the virus has been undetectable in your blood for at least a year. We would like at least 160 young people to take part. If you join the study a computer will pick at random which group you will be in so it is fair.

The two groups are:

- to continue to take antiretroviral medicines every day without any interruptions **(continuous therapy group).**
- to take medicines during the week but stop taking them at weekends (you can choose whether to stop on either Friday and Saturday or Saturday and Sunday, although you must stick to the days chosen once you have decided which are best for you **(SCT group).** If you would prefer to take 2 consecutive days off during the week other than Friday and Saturday or Saturday and Sunday, please speak to the study nurse or doctor.

You have a 50% chance of being in the SCT group (stopping medicines at weekends).

Your viral load will be monitored closely. If for any reason, your anti-HIV treatment needs to be changed this will be done by their doctor, in the usual way, and will not be affected by the group you are in.

3) Participation
It is totally up to you whether or not you take part in this study. It is your right to decide not to take part if you wish. If you don’t want to take part it will not affect your treatment now or in the future in any way. If you do decide to take part we will describe the study and go through this information

sheet which we will then give to you. We will also ask you to sign a consent form to show you have understood the information and agreed to take part. **You are free to withdraw from the study at any time without giving a reason**. Withdrawing from the study will not affect your medical care. We hope, however, that if you do withdraw you would give us a reason and will allow us to continue to collect information from your normal hospital visits. This will help us when we look at the results of the study.

As part of the study, blood samples will be stored for up to 15 years. We would hope that if you withdrew from the study you would still allow us to use these samples. If, however, you did not feel that this was appropriate then we would destroy them.

4) What will happen to me if I decide to take part?

Visits and duration: When you join the study you will initially be seen for two clinic visits in the space of one month to make sure it is OK for you to start the study.

To help remember to stop taking medicines at weekends and re-start after the 2 day break a diary will be given out for completion.
In addition the nurses at your clinic can arrange for text message reminders to be sent to you if you think this may help. ***(*)***

SCT group
Young people enrolled in the SCT group will be asked to come for a clinic visit four weeks after randomisation and then your visits to clinic will be every 3 months as usual.

SCT group - viral load testing
In the pilot phase and in the main study, if at any time your viral load is more than 50 copies per ml you will be asked to provide a second blood sample for repeat testing within 1 week. If the result of the second test is also more than 50 copies per ml, you will be asked to start taking your antiretroviral medicines every day for the rest of the study and will not be able to have any more weekend breaks.

Continuous Treatment Group: If you are put in the group continuing to take antiretroviral medicines every day you will then have to come back for an additional clinic visit 1 month after enrolling in the study. Your visits to clinics will then be every 3 months as usual. If you are in this group you will not be allowed to stop your anti-HIV medicines at any time.

Both groups: At clinic visits your height and weight will be measured, and the doctor will look for signs of any illnesses. The doctor will also monitor the onset of puberty every 6 months as puberty can sometimes be delayed by HIV infection.
Blood will be collected and stored at each visit. These will be tested at the end of the study to find out what happens when the medicines are stopped at weekends. We would like your agreement that stored blood samples can be used in future for tests which help us learn more about HIV.

You will be followed in the study until all young people taking part have completed one year of follow-up. At this point the study will end. Normal clinic visits will then continue for routine monitoring of your HIV and you will go back to taking medicines everyday.

5) Adherence and Acceptability and Quality of Life questionnaires
We are very interested in finding out what young people and families feel about the way the study is going and how it is affecting your quality of life. Therefore over the course of the study we will ask you to complete questionnaires designed to measure adherence to medication, acceptability of the way you are taking your medications and quality of life.

We would like to carry out an additional study in 60 young people in the trial using MEMS (Medication Event Monitoring Systems) caps. This is an electronic device fitted to medication bottles which records the time and date of each opening.
If you agree to take part in this part of the study you will be given a MEMS cap to fit to your medication bottle. Your doctor or nurse will explain how it works and how to use it.
You must only open the MEMS caps to withdraw the medicine at the times you are going to take the dose. You must not open the container at any other time or transfer medications to other containers. You will need to take the MEMS bottles with you if you are staying away from home. ***(**)***

6) What are the possible benefits of taking part?
It is hoped that short cycle therapy (SCT) will:

- lead to a less restrictive way of life as you may be able to stop taking medicines at the weekend.
- lead to fewer long term side-effects from taking antiretroviral medicines for you.

We cannot promise that participating in the study will help you directly. For example, if you are in the group staying on medicines every day, nothing will change except for some extra clinic visits. However, all the information we get might help children and young people with HIV in the future and may mean you would also have the opportunity to stop medicines at weekends eventually.

7) What are the possible disadvantages and risks of taking part?

- You might find it harder to take antiretrovirals during the week and then remember to stop them at weekends if you are in the SCT group.
- Not taking your medicines everyday can lead to resistance. This means that the medicines may not work so well anymore and new medicines will be needed, particularly if you forget to take the medicines during the week when you should be taking them. It is also important that if you are in the group that is to continue taking your medicines every day that you take them properly every time.
- You will have to visit the clinic more often than normal even if you are in the group that has to continue taking medicines every day.

8) Pregnancy and anti-HIV medicines
Some combinations of anti-HIV medicines might harm an unborn child. If you are female and could become pregnant, you must have a pregnancy test before entering the study and you must use effective contraceptives including condoms or other barrier contraception if you are having sexual intercourse. If you are male and having sexual intercourse you must use condoms.

9) Who has reviewed the study?
This study has been looked at by an independent group of people (a Research Ethics Committee). Their task is to check the study and make sure as far as possible; no harm comes to anyone from being part of the study. This study has been reviewed and given favourable opinion by a Research Ethics Committee. A second group (the Independent Data Monitoring Committee) will meet regularly during the trial and will recommend whether the trial should continue as planned or should stop. Information from these meetings will be summarised in the PENTA Newsletter for families involved in PENTA studies.
We have completed a pilot study with close monitoring of viral load results over the first 3 weeks of stopping medicines at weekends. The Independent Data Monitoring Committee looked at the results of this pilot and were happy for the larger study to continue.

10) Confidentiality
All information collected during the BREATHER study will be kept completely confidential and your name will not be used. You will only be identified using a study number and date of birth. Your hospital notes will only be made available to study staff and confidentiality will be maintained at all times. If the results of this study are reported or published your name will not be

used. The study will be conducted in compliance with the Data Protection Act, 1998. Data will be archived for 15 years and only anonymised data will be sent outside the European Economic Area (EEA).

11) What happens at the end of the study?
After the study has ended all the participants in the SCT group will go back to taking medication every day. Your doctor will continue to make decisions about your medicines once the study has finished. If stopping medicines at the weekend is found to be a safe and effective way of treating children and young people with HIV who are taking certain anti-HIV medicines then this may continue to be part of your clinical care. We would also like to continue to collect routine data about you after the study has ended so we can look at any long term effects of the study.

12) Compensation arrangements
The PENTA Foundation has made arrangements for compensation should you come to any harm because of being in this study. However, if you are harmed due to someone's negligence then you may have grounds for legal action for compensation against the hospital where the negligence occurred, but you may have to pay your legal costs.
Your GP will be informed of your participation in the study with your permission.
Your hospital doctor is not receiving any personal payment for carrying out this research.

13) Patient contact
If you have any concerns or other questions about the study or the way it has been carried out, or if you have an injury or illness during your participation, contact either the investigator (Dr ___________________/ PENTA team *Phone Number here*) or the hospital where the study is being carried out:

Dr ……………………………….
Dr………………………………..
Nurse ………………………….

Thank you for taking the time to consider this study. Please ask any questions and let us know if there are things that you do not understand or would like more information about.

BREATHER
Study Title: Short-Cycle Therapy (SCT) (5 days on / 2 days off) in young people with chronic HIV-infection

AN INFORMATION SHEET FOR YOUNG PEOPLE.

We would like to invite you to join our research study to find out if it is safe to have weekends free from taking your anti-HIV medicines.

Before you decide, it is important that you to understand why we want to carry out this study and what it will mean for you. Please take your time to read the following information and feel free to discuss it with your friends, relatives and your doctor or clinic nurse. Please ask if you need more information or if there is anything that is not clear. Take as much time as you need to decide whether or not you wish to take part.

1) What is the study?
The study that you are being invited to join is called BREATHER. It is organised by PENTA, an independent organisation that answers questions about treatment issues for children and young people infected with HIV. This study will include young people from many different countries.

BREATHER is a study that will compare two different ways of taking HIV (antiretroviral) medicines: either taking the medicines all of the time (continuous therapy - as you are doing at the moment) or taking the medicines during the week for 5 days and then having a rest for 2 days (i.e. no medicine at the weekend). This second strategy where you have a short break in your treatment each week is called **Short Cycle Therapy (SCT).**

2) Why is it being done?
As you know, taking anti-HIV medicines every day without missing a dose is important. This is to stop the virus becoming resistant (i.e. the medicines do not work as well as they should). However, some of the medicines that are now available remain in the blood for a longer time, so doctors and scientists are now working towards new ways for people to take the medicines and make it easier for people. **In this study, we will be examining whether it is safe to stop taking medicines for 2 days at weekends and whether they still work as well as when they are taken every day**.

3) Why have I been chosen?
You have been asked to take part in this study because some of the antiretroviral medicines that you are taking stay in the blood for a longer time, and because the virus has been undetectable in your blood for at least a year. We would like at least 160 young people to take part. If you join the study a computer will pick at random which group you will be in so it is fair.

The two groups are:
- to continue to take antiretroviral medicines every day without any interruptions **(continuous therapy group).**
- to take medicines during the week but stop taking them at weekends (you can choose whether to stop on either Friday and Saturday or Saturday and Sunday, although you must stick to the days chosen once you have decided which are best for you **(SCT group).** If you would prefer to take 2 consecutive days off during the week other than Friday and Saturday or Saturday and Sunday, please speak to the study nurse or doctor.

You have a 50% chance of being in the SCT group (stopping medicines at weekends).

4) Do I have to take part?
No, it is totally up to you whether or not you take part in this study. If you do, your doctor will ask you to sign a form giving your agreement to take part (assent). You will be given a copy of this information sheet and your signed form to keep. If you don't want to take part it will not affect your treatment now or in the future in any way.

If you do decide to take part **you can withdraw from the study at any time without giving a reason**. Withdrawing from the study will not affect your medical care. We hope, however, that if you do withdraw you would give us a reason and will allow us to continue to collect information from your normal hospital visits. This will help us when we look at the results of the study.

As part of the study, your blood samples will be stored for up to 15 years. We hope that if you withdraw from the study you would still allow us to use these samples. If, however, you are not happy to do this then we would destroy them.

5) What will happen to me if I decide to take part?

Clinic visits
When you join the study you will be seen for two clinic visits in the space of one month to make sure it is OK for you to start the study.

To help remember to stop taking medicines at weekends and re-start after the 2 day break we will give you a diary to fill in.
The nurses at your clinic can also arrange for text message reminders to be sent to you if you think this may help. ***(*)***

The doctor will measure the amount of virus in your body (viral load) during the study to make sure that your medicines are working properly. If the level of virus gets too high, you will be asked to come back for a second test within one week. If the result of the second test is still the same, you will be asked to start taking your medicines every day for the rest of the study and you will not be able to have any more weekend breaks.

Continuous Treatment Group: If you are in the group continuing to take antiretroviral medicines every day you will have to come back 1 month after enrolling in the study. Your visits to clinics will then be every 3 months as usual. If you are in this group you should not stop your anti-HIV medicines at any time.

Both Groups:
At clinic visits your height and weight will be measured, and the doctor will look for signs of any illnesses. The doctor will also monitor the onset of puberty every 6 months as puberty can sometimes be delayed by HIV infection. Blood will be collected and stored at each visit. These will be tested at the end of the study to find out what happens when the medicines are stopped at weekends. We would like your agreement that stored blood samples can be used in future for tests which help us learn more about HIV.

Your doctor will follow you until all the young people taking part in the study have been followed up for one year. At this point the study will end, but normal clinic visits will continue for routine monitoring of your HIV and you will go back to taking medicines every day.

6) Adherence, acceptability and Quality of Life questionnaires
We are very interested in finding out what young people and families feel about the way the study is going and how it is affecting your life and quality of life. Therefore during the study we will ask

you to complete questionnaires asking you about when you are taking your medication, whether you are happy with the study and how it affects you.

We would like to carry out an additional study in 60 young people in the trial using MEMS (Medication Event Monitoring Systems) caps. This is an electronic device fitted to medication bottles which records the time and date of each opening.
If you agree to take part in this part of the study you will be given a MEMS cap to fit to your medication bottle. Your doctor or nurse will explain how it works and how to use it.
You must only open the MEMS caps to withdraw the medicine at the times you are going to take the dose. You must not open the container at any other time or transfer medications to other containers. You will need to take the MEMS bottles with you if you are staying away from home. **(**)**

7) What are the possible benefits of taking part?
We hope that short cycle therapy will:
- make things easier in your life as you may be able to stop taking medicines at the weekend if you are in the SCT group.
- you might experience fewer long term side-effects from taking the medicines.

We cannot promise that joining in with the study will help you directly. For example, if you are in the group staying on medicines every day, nothing will change except for some extra clinic visits. However, all the information we get might help young people with HIV in the future and may mean you would also have the opportunity to stop medicines at weekends eventually.

8) What are the possible disadvantages and risks of taking part?
- You might find it harder to take antiretrovirals during the week and then remember to stop them at weekends if you are in the SCT group.
- Not taking antiretrovirals everyday can lead to resistance. This means that the medicines may not work so well anymore and new medicines will be needed, particularly if you forget to take the medicines during the week when you should be taking them. It is also important that if you are in the group that is to continue taking your medicines every day that you take them properly every time.
- You will have to visit the clinic more often than normal even if you are in the group that has to continue taking medicines every day.

9) Pregnancy and anti-HIV medicines
Some combinations of anti-HIV medicines might harm an unborn child. If you are female and could become pregnant, you must have a pregnancy test before entering the study and you must use effective contraceptives including condoms or other barrier contraception if you are having sexual intercourse. If you are male and having sexual intercourse you must use condoms.

10) Who has reviewed the study and who will monitor its progress?
This study has been looked at by an independent group of people (a Research Ethics Committee). Their task is to check the study and make sure as far as possible; no harm comes to anyone from being part of the study. This study has been reviewed and given favourable opinion by a Research Ethics Committee. A second group (the Independent Data Monitoring Committee) will meet regularly during the trial and will recommend whether the trial should continue as planned or should stop. Information from these meetings will be summarised in the PENTA Newsletter for families involved in PENTA studies.
We have completed a pilot study with close monitoring of viral load results over the 1st 3 weeks of stopping medicines at weekends. The Independent Data Monitoring Committee looked at the results of this pilot and were happy for us to continue with the larger study.

11) Will my taking part in the study be kept confidential?
All information collected during the BREATHER study will be kept completely confidential and your name will not be used. You will only be identified using a study number and date of birth. Your hospital notes will only be made available to study staff and confidentiality will be maintained at all times. If the results of this study are reported or published your name will not be used. The study will be conducted in compliance with the Data Protection Act, 1998. Data from the study will be kept for 15 years.

12) What happens at the end of the study?
After the study has ended you will go back to taking your medication every day. You and your doctor will continue to make decisions about your treatment. If stopping your medicines at the weekend is found to be a safe and a good way of treating children and young people with HIV who might be able to continue with having a treatment break for 2 days at weekends. We would also like to continue to collect routine data about you after the study has ended so we can look at any long term effects of the study.

13) Patient contact
If you have any concerns or other questions about the study or the way it has been carried out, or if you have an injury or illness during your participation, contact either the investigators (Dr. ________________/ PENTA team *Phone Number here*) or the hospital where the study is being carried out:

Dr ……………………………….
Dr………………………………..
Nurse …………………………

Thank you for taking the time to consider this study. Please ask any questions and let us know if there are things that you do not understand or would like more information about.

BREATHER
Study Title: Short-Cycle Therapy (SCT) (5 days on / 2 days off) in young people with chronic HIV-infection

AN INFORMATION SHEET FOR CHILDREN

We would like to invite you to join our study. Before you decide, it is important for you to understand why this study is being done and what it will mean for you.
Please take your time to read this information sheet and decide whether or not your wish to take part in this study.
Please ask your parents and doctor or nurse if you need more information or if you have questions.
Take as much time as you need to decide whether or not you wish to take part.

1) What is research?
Research is the way we try to find out the answers to questions about science and medicine. In this study we want to understand more about different ways that you can take your anti-HIV medicines that might make it easier for you.

2) What is the study?
The study is for children and young people who are very good at taking anti-HIV medicines. We will ask at least 160 children and young people to take part from many different countries.

We will look at two different ways of taking anti-HIV medicines.

The two ways are:
- To continue to take your medicines every day (as you do now)
- To take medicines during the week but stop taking them at weekends. This is called Short-cycle therapy (SCT). You can choose whether to stop on either Friday and Saturday, OR, Saturday and Sunday, and you must stick to the same days chosen for the whole study. If you want to stop on a different two days during the week that are not Friday and Saturday, or Saturday and Sunday, talk to your nurse or doctor.

If you take part in the study a computer will pick which group you will be in. This means we have a fair test.

3) Why is this study being done?
We want to know more about what happens if children and young people do not take their anti-HIV medicines at weekends and if it is OK. It is important to understand that at the moment you should take your anti-HIV medicines every day without missing a dose. We want to do a study to find out if it is ok to not take anti-HIV medicines at the weekend.

4) Why have I been chosen?
You have been asked to take part in this study because you have done well on your medicines and because of the type of medicines that you are taking.

5) Is this research study OK to do?
This research study has been checked by a group of people called a Research Ethics Committee. They make sure that the research is safe and fair. This project has been checked by the (name here) Research Ethics Committee.
During the study a group of doctors and scientists regularly check that everything is alright and safe to continue, they are called an Independent Data Monitoring Committee (IDMC). They have checked the results from some young people who have already taken part and have said it is OK to continue.

6) Do I have to take part?
It is up to you and your family to choose whether you take part in this study. It is absolutely fine if you do not want to take part. But if you decide to take part in the study you can change your mind at any time during the study without giving a reason. Nobody will be cross with you. Your doctor will still look after you as they are now. We hope, however, that if you did stop taking part in the study we would still be able to check how you are doing by collecting information from your normal hospital visits.

7) What happens to me if I take part?
If you take part you will be asked to come to the clinic every 3 months as normal to make sure you are OK. At the start of the study we will ask you to come for extra clinic visits. Your doctor or nurse will tell you when you have to come to clinic.
The doctor will make sure you are keeping well and will take some of your blood for testing.

We will give you a diary to help you remember when to stop and start your medications. Your doctor or nurse can also arrange for a text message to be sent to you if you want to.

We will take a blood test each time you come to the clinic. The blood samples will be kept and tested at the end of the study to find out what happens when the medicines are stopped at weekends.

We are also interested in finding out how easy you find it to remember to take your medicines, and how it affects your life so we will ask you to fill in some questionnaires during the study.

We may also ask you to use a MEMs cap which is placed on top of your medicine bottle and allows us to look at when you take your medicines. ***(**)***

After the study has ended if you are in the SCT group, you will go back to taking your medicines every day.

8) How will joining in help me?
- You have a 50:50 chance that you will not have to take medicines at weekends – for example making sleep-overs easier.
- You will take fewer tablets and might have fewer problems with taking the medicines if you are in the group having weekends off.

We cannot promise the study will help you directly. For example, if you are in the group staying on medicines every day, nothing will change except for some extra clinic visits.

However, all the information we get might help children with HIV in the future and may mean your doctor might decide that it is also OK for you to stop medicines at weekends after the study has finished.

9) Are there any risks?

- You might find it harder to remember to take medicines during the week and to remember to stop taking your medicines at the weekend, if you are in the weekend stopping group.
- The medicines may not fight the virus as well as they do when you take them every day. This might also happen if you forget to take the medicines during the week when you should be taking them.
- You will have to visit the clinic more often than normal.

10) Will my details be kept private if I take part?

We would like to tell your family doctor that you are taking part in this study. No one else will know unless it is necessary.
Your name will be changed to a special study number and date of birth.

11) The study results

At the end of the study we will tell you how the study went for young people in both the weekend stopping group and the “taking your medicines as usual” group.

12) Patient contact

If you have any questions about the study, these are the phone numbers of the doctors and nurses involved:

Dr ……………………………….
Dr………………………………..
Nurse …………………………..

Thank you for taking the time to think about this study. Please ask any questions and let us know if there are things that you do not understand or you would like to talk to someone else about.

BREATHER
Study Title: Short-Cycle Therapy (SCT) (5 days on / 2 days off) in young people taking medicines long term

AN INFORMATION SHEET FOR CHILDREN

Why are we doing the study?

This study is to find out more about how you can take your medicines and how to keep children and young people like you healthy.

Why do you want me to take part?

We would like you to take part because you see the doctors and nurses in this clinic and you have to take medicines every day.

Do I have to take part?

No, it is up to you and your family. You can decide that you don't want to take part at any time and *you don't have to tell us why*.

What happens to me in the study?

If you take part you will be put into one of 2 groups.
A computer program will decide which group you are in:

- Group 1: You'll continue taking all your medicines as you usually do

- Group 2: You won't be given some of your medicines at the weekend.

You will be asked to come to the clinic every 3 months as normal to make sure you are OK. At the start of the study we will ask you to come for 2 extra clinic visits. Your doctor or nurse will tell you when you have to come to clinic.
We would also like to do a blood test at each visit. You can have this done at the same time as the blood tests you have in clinic, so you won't need to have another needle.
No matter what group you are in, if at any time your doctor tells you to take your medicines every day, you should do this.

Will anyone know I am taking part in the study?

We won't tell anyone apart from your family doctor that you are taking part in the study, and we won't give your name or address to anyone else. You'll have a special code number that will be used on any information about you.

Who has given you permission to do this study?

Before we started the study we got permission from an ethics committee. They make sure that no harm can come to anyone from being part of the study.

Who can I talk to if I have some questions about the study?

Names of Study Doctor and Nurse plus contact numbers to be added here.

Thank you very much for reading this information sheet

APPENDIX 2: SAMPLE CONSENT FORMS

I. Parents/carers
II. Young adults (16 years and over)
III. Assent form for young people (aware of diagnosis)
IV. Assent form for children (unaware of diagnosis) For use if corresponding information sheet is used

All to be printed on local headed paper

Consent Form for parents/carers

Title of Study: BREATHER - Short Cycle Therapy (SCT) (5 days on / 2 days off) in young people with chronic HIV-infection.

Please write your initials in each box if you agree:

I have read and understood the information sheet on the BREATHER study, version…, dated……..	
I understand the potential benefits and disadvantages of my child participating in this study.	
The details of this study have been explained by: Dr………………………………… who has answered my questions satisfactorily.	
I agree to my child's routine blood results and clinical information being included in continued follow up after the study has ended.	
I know that my child can be withdrawn from the allocated group at any time without it affecting his/her care.	
I know that my child can be withdrawn from the study follow-up and assessment at any time without it affecting his/her care.	
I agree to linked anonymised blood samples being taken and processed during the study and to be stored for ethically approved studies so that further work can be done to help understand more about HIV and effective treatment	
I understand that relevant sections of my child's medical notes and data collected during the study may be looked at by individuals from the PENTA group, from national authorities or from the hospital, where it is relevant to my child taking part in this research. I give permission for these individuals to have access to my child's records which will be treated as strictly confidential.	
I agree that anonymised direct quotes from the acceptability and/or adherence questionnaires may be used.	
If invited, I agree to take part in the adherence study (using MEMS cap).	
If invited, I agree to receive text messages.	
I agree that my child should take part in this study comparing continuous therapy with short cycle therapy (breaks at weekends).	

Name of child:_______________________Name of Parent/Guardian: ______________________

Signature of Parent/Guardian:______________________________Date:_______

Name of clinician:_______________________________________

Signature of clinician:_____________________________________Date:_______

I would/would not *(please circle)* like my child's GP to be notified about participation in this study.

Signature of Parent/Guardian:______________________________Date:_____________________

Name of GP:___________________**Contact address of GP**______________________________

CONSENT FORM FOR YOUNG ADULTS

Title of Study: PENTA16 - Short Cycle Therapy (SCT) (5 days on / 2 days off) in young people with chronic HIV-infection.

Please write your initials in each box if you agree:

I have read and understood the information sheet on the BREATHER study, version…, dated……..	
I understand the potential benefits and disadvantages of participating in this study.	
The details of this study have been explained by: Dr………………………………who has answered my questions satisfactorily.	
I agree to my routine blood results and clinical information being included in continued follow up after the study has ended.	
I know that I can withdraw from the allocated group at any time without it affecting my care.	
I know that I can withdraw from the study follow-up and assessment at any time without it affecting my care.	
I agree to linked anonymised blood samples being taken and processed during the study and to be stored for ethically approved studies to help understand more about HIV and effective treatment	
I understand that relevant sections of my medical notes and data collected during the study may be looked at by individuals from the PENTA group, from national authorities or from the hospital, where it is relevant to my taking part in this research. I give permission for these individuals to have access to my records which will be treated as strictly confidential.	
I agree that anonymised direct quotes from the acceptability and/or adherence questionnaires may be used.	
If invited, I agree to take part in the adherence study (using MEMS cap).	
If invited, I agree to receive text messages.	
I agree to take part in this study comparing continuous therapy with short cycle therapy (breaks at weekends).	

Name of participant______________________________

Signature of participant___________________________Date_______________

Name of person taking consent______________________

Signature of person taking consent________________________ Date______________

I would/would not like *(please circle)* my GP to be notified about my participation in this study

Signature of Parent______________________________ Date_______________

Name of GP ___________________________________

Contact address of GP____
____

Assent Form

Title of Study: BREATHER - Short-Cycle Therapy (SCT) (5 days on / 2 days off) in young people with chronic HIV-infection.

Please tick yes or no to the questions below

1. Have you read (or had read to you) the information about this study?	YES ☐ NO ☐
2. Has somebody explained the study to you?	YES ☐ NO ☐
3. Do you understand what the study is about?	YES ☐ NO ☐
4. Have you asked all the questions you want?	YES ☐ NO ☐
5. Have your questions been answered in a way you understand?	YES ☐ NO ☐
6. Do you understand how the study might help you?	YES ☐ NO ☐
7. Do you understand the risks of taking part?	YES ☐ NO ☐
8. Do you understand it's OK to stop taking part in the study at any time?	YES ☐ NO ☐
9. Do you understand that if you take part you may not be in the group that stops taking medicines at weekends?	YES ☐ NO ☐
10. Do you agree that your blood samples (without your name on) can be used during the study and stored for studies which will help us understand more about the virus?	YES ☐ NO ☐
11. Are you happy to take part?	YES ☐ NO ☐

If any answers are **'no'** or you don't want to take part, **don't sign your name**.

If you **do** want to take part, you can sign your name below.

Your name________________________ Date _____________

If invited, I agree to take part in the adherence study (using MEMS cap).	YES ☐ NO ☐
If invited, I agree to receive text messages	YES ☐ NO ☐

The doctor who explained the project to you needs to sign too:

Doctor's name (print) ____________________ Sign __________________Date________

Assent Form

Title of Study: BREATHER - Short-Cycle Therapy (SCT) (5 days on / 2 days off) in young people taking medicines long term

Please tick yes or no to the questions below

1. Have you read (or had read to you) the information about this study?	YES ☐ NO ☐
2. Have you asked all the questions you want?	YES ☐ NO ☐
3. Do you understand what the study is about?	YES ☐ NO ☐
4. Do you understand that if you take part you may be in the group that carries on taking medicines at weekends as usual?	YES ☐ NO ☐
5. Can we take some blood from you at each clinic visit?	YES ☐ NO ☐
6. Are you happy to take part?	YES ☐ NO ☐

If any answers are **'no'** or you don't want to take part, **don't sign your name**!

If you **do** want to take part, you can sign your name below.

Your name________________________ Date _____________

The doctor who explained the project to you needs to sign too:

Doctor's name (print) ____________________

Sign __________________ Date________

APPENDIX 3: SAMPLE GP LETTER

GP Letter

(To be printed on local headed paper)

Date:

BREATHER: Short-Cycle Therapy (SCT) (5 days on / 2 days off) in young people with chronic HIV-infection.

ISRCTN 97755073

Dear Dr ____________

The parents of ……………………………. (and ……………………… herself/himself) have agreed to participate in the BREATHER study. This is a multicentre, randomised clinical trial by the PENTA network (Paediatric European Network for the Treatment of AIDS).

The overall aim of the BREATHER trial is to evaluate the role of Short-Cycle Therapy (SCT) in the management of HIV-infected young people who have responded well to antiretroviral therapy.

The specific aim of the study is to determine whether young people with chronic HIV infection undergoing Short-Cycle Therapy of five days on and two days off maintain the same level of viral load suppression as those on continuous therapy. Acceptability, quality of life and adherence to randomised strategy will be compared.

The study will enrol at least 160 children and young people from the PENTA centres.

This young person has been allocated to receive

- ☐ continuous therapy
- ☐ SCT (5 days on/2 days off)

I enclose a summary of the trial and a patient information sheet. You will be kept up to date with your patient's progress but if you have any concerns or questions regarding this study please contact the responsible doctor:

Dr ______________________________ at __________________________(Hospital)

Tel: ______________________________

Kind regards,

Name
Position

Enc. Trial Summary
Patient Information Sheets

APPENDIX 4: ADHERENCE QUESTIONNAIRES

Screening

- Adherence questionnaire for young people in both groups at screening
- Adherence questionnaire for carers in both groups at screening

Follow-up

- Adherence questionnaire for young people in the SCT group (weekends-off) at follow-up
- Adherence questionnaire for carers in the SCT group (weekends-off) at follow-up
- Adherence questionnaire for young people in the CT group (continuous) at follow-up
- Adherence questionnaire for carers in the CT group (continuous) at follow-up

Young People
Both Groups
Screening

BREATHER

ADHERENCE QUESTIONNAIRE FOR YOUNG PEOPLE
Both Groups – SCREENING

**Trial number:** __ __ __ __ __ __ __ __ __ ☐☐☐☐		Date of birth: / /
**Male** ☐ **Female** ☐	Date of assessment: / /	Date of last clinic visit: / /

We know that it can be difficult taking HIV medicines every day. We are interested in finding out what it is like for you. Being honest about whether or not you take your medicines may help others in the future. Please tick the answer that best describes what is happening to you. Please only think about the HIV medicines you are taking, and not any other medicines. Thank you for your help

1. Some people find that they forget to take their medicines at the weekend. Did you miss any of your HIV medicines last weekend (Friday, Saturday or Sunday)?
   - Yes ☐
   - No ☐

2. When was the last time you missed any HIV medicine doses? *(please tick one box)*
   - Within the last week ☐
   - 1-2 weeks ago ☐
   - 3-4 weeks ago ☐
   - 1-3 months ago ☐
   - Not at all since last visit ☐

3. Some people find that they aren't able to take medicines every day. Have you missed all of your HIV medicines for two days or more in a row since your last clinic visit?
   - Yes ☐
   - No ☐

4. Please mark on the line below the amount of HIV medicine doses you have taken since your last clinic visit.
   e.g. 0% would mean "I haven't taken any of my HIV medicine doses" and 100% would mean "I have taken all of my HIV medicine doses"

   0% (None) – 10% – 20% – 30% – 40% – 50% – 60% – 70% – 80% – 90% – 100% (All)

5. Did you complete this form on your own?
   - Yes ☐
   - No ☐

   If No, who else was involved? (e.g parent, nurse, friend)

   ____

Thank you for taking the time to complete this form. Please add any comments that you may have here:

Carers
Both Groups
Screening

BREATHER

ADHERENCE QUESTIONNAIRE FOR CARERS
Both Groups – SCREENING

**Trial number:** __ __ __ __ __ __ __ __ __ ☐☐☐☐		**Date of birth:** / /
**Male** ☐ **Female** ☐	**Date of assessment:** / /	**Date of last clinic visit:** / /

We know that it can be difficult giving HIV medicines to your child every day. We are interested in finding out what it is like for you and your family. Being honest about whether or not your child takes their medicines may help others in the future. Please tick the answer that best describes what is happening as far as you know. Please only think about the HIV medicines your child is taking, and not any other medicines.

If your child is responsible for taking their own medicines then you do not need to complete this questionnaire. Thank you for your help.

1. What is your relationship to the child? ____________________
2. Who gives medicines to your child? (eg. you/ your partner/ other family member) ____________________
3. Some people find that they forget to take their medicines at the weekend. Did your child miss any of their HIV medicines last weekend (Friday, Saturday or Sunday)?
   Yes ☐
   No ☐
4. When was the last time the child missed any HIV medicine doses? *(please tick one box)*
   Within the last week ☐
   1-2 weeks ago ☐
   3-4 weeks ago ☐
   1-3 months ago ☐
   Not at all since last visit ☐
5. Some people find that they aren't able to take medicines every day. Has your child missed all their HIV medicines for two days or more in a row since their last clinic visit?
   Yes ☐
   No ☐
6. Please mark on the line below the amount of HIV medicine doses your child has taken since their last clinic visit.
   e.g. 0% would mean "They haven't taken any of their HIV medicine doses" and 100% would mean "They have taken all of their HIV medicine doses"

   0% (None) | 10% | 20% | 30% | 40% | 50% | 60% | 70% | 80% | 90% | 100% (All)
7. Did you complete this form alone?
   Yes ☐
   No ☐ If No, who else was involved? (e.g. nurse, doctor) ____________________

Thank you for taking the time to complete this form. Please add any comments that you may have here:

Young People
Weekends off
Follow-Up

BREATHER

ADHERENCE QUESTIONNAIRE FOR YOUNG PEOPLE

Weekends off Group – FOLLOW-UP

**Trial number:** __ __ __ __ __ __ __ __ __ ☐☐☐☐		**Date of birth:** / /
**Male** ☐ **Female** ☐	**Date of assessment:** / /	**Week no:** *(tick one)* 4 ☐ 12 ☐ 24 ☐ 36 ☐ 48 ☐ other ☐

We know that it can be difficult taking HIV medicines every day. We are interested in finding out what it is like for you. Being honest about whether or not you take your medicines may help others in the future. Please tick the answer that best describes what is happening to you. Please only think about the HIV medicines you are taking, and not any other medicines. Thank you for your help

1. **Which HIV medicine doses are you supposed to miss as part of this trial?** ***(Please tick 4 boxes for a twice daily regimen, and 2 boxes for a once daily regimen)***

	AM	PM
Friday	☐	☐
Saturday	☐	☐
Sunday	☐	☐

2. **Since your last clinic visit did you miss all the doses you were supposed to miss?**
   Yes ☐ *Go to Q4*
   No ☐ *Go to Q3*

3. **a. If no, how many weekend breaks did you take?** *(write number in box)* ☐

   b. Please give reasons for not taking a break: *(Tick all that apply)*
   Told not to miss weekend doses by doctor ☐
   Didn't want to miss weekend doses ☐
   Forgot to miss weekend doses ☐
   Didn't understand about missing weekend doses ☐
   Other ☐

   If other, please specify here:

4. **Since your last clinic visit, did you always restart your HIV medicine doses immediately after your weekend breaks?**
   Yes ☐ *Go to Q6*
   No ☐ *Go to Q5*

5. **If no, after how many weekends did you not restart your doses immediately after the weekend break?** *(write number in box)* ☐

6. **When was the last time you missed any HIV medicines apart from those allowed at the weekend?** *(please tick one box)*
   Within the last week ☐
   1-2 weeks ago ☐
   3-4 weeks ago ☐
   1-3 months ago ☐
   Not at all since last visit ☐

7. **Please mark on the line below the amount of HIV medicine you have taken since your last clinic visit (please ignore any breaks that are allowed at the weekend).**
   e.g. 0% would mean "I haven't taken any of my HIV medicine doses" and 100% would mean "I have taken all of my HIV medicine doses when I was supposed to (i.e. taken the breaks as my doctor told me")

0% (None) — 10% — 20% — 30% — 40% — 50% — 60% — 70% — 80% — 90% — 100% (All)

Please turn over

8. Did you complete this form alone?

Yes ☐

No ☐ **If No, who else was involved?**
(e.g parent, nurse, friend)

____

Thank you for taking the time to complete this form. Please add any comments that you may have here:

Carers
Weekends off
Follow-Up

BREATHER

ADHERENCE QUESTIONNAIRE FOR CARERS
Weekends off Group – FOLLOW-UP

**Trial number:** _ _ _ _ _ _ _ _ _ _ ☐☐☐☐		**Date of birth:** / /
**Male** ☐ **Female** ☐	**Date of assessment:** / /	**Week no:** *(tick one)* 4 ☐ 12 ☐ 24 ☐ 36 ☐ 48 ☐ other ☐

We know that it can be difficult giving HIV medicines to your child every day. We are interested in finding out what it is like for you and your family. Being honest about whether or not your child takes their medicines may help others in the future. Please tick the answer that best describes what is happening as far as you know. Please only think about the HIV medicines your child is taking, and not any other medicines.

If your child is responsible for taking their own medicines then you do not need to complete this questionnaire. Thank you for your help.

1. **What is your relationship to the child?** ______________________

2. **Who gives medicines to your child?** **(eg. you/ your partner/ other family member)** ______________________

3. **Which HIV medicine doses are they supposed to miss as part of this trial?** ***(Please tick 4 boxes for a twice daily regimen, and 2 boxes for a once daily regimen)***

	AM	PM
Friday	☐	☐
Saturday	☐	☐
Sunday	☐	☐

4. **Since their last clinic visit did your child miss all the doses they were supposed to miss as part of this trial?**
   Yes ☐ ***Go to Q6***
   No ☐ ***Go to Q5***

5. **a. If no, how many weekend breaks did your child take?** ***(write number in box)*** ☐

   b. Please give reasons for not taking a break: (***Tick all that apply)***
   Told not to miss weekend doses by doctor ☐
   Didn't want to miss weekend doses ☐
   Forgot to miss weekend doses ☐
   Didn't understand about missing weekend doses ☐
   Other ☐

   If other, please specify here:

6. **Since their last clinic visit, did your child always restart their HIV medicine doses immediately after their weekend breaks?**
   Yes ☐ ***Go to Q8***
   No ☐ ***Go to Q7***

7. **If no, after how many weekends did your child not restart their doses immediately after the weekend break?** ***(write number in box)*** ☐

8. **When was the last time your child missed any HIV medicines apart from those allowed at the weekend?** ***(please tick one box)***
   Within the last week ☐
   1-2 weeks ago ☐
   3-4 weeks ago ☐
   1-3 months ago ☐
   Not at all since last visit ☐

Please turn over

9. Please mark on the line below the amount of HIV medicine doses your child has taken since their last clinic visit (please ignore any breaks that are allowed at the weekend).
e.g. 0% would mean "They haven't taken any of their HIV medicine doses" and 100% would mean "They have taken all of their HIV medicine doses when they were supposed to (i.e. taken the breaks as directed by the doctor")

0% (None)	10%	20%	30%	40%	50%	60%	70%	80%	90%	100% (All)

10. Did you complete this form alone?

Yes ☐
No ☐

If No, who else was involved?
(e.g nurse, doctor)

____

Thank you for taking the time to complete this form. Please add any comments that you may have here:

Young people
Continuous
Follow-up

BREATHER

ADHERENCE QUESTIONNAIRE FOR YOUNG PEOPLE
Continuous group – FOLLOW-UP

**Trial number:** _ _ _ _ _ _ _ _ _ ☐☐☐☐		**Date of birth:** / /
**Male** ☐ **Female** ☐	**Date of assessment:** / /	**Week no:** *(tick one)* 4 ☐ 12 ☐ 24 ☐ 36 ☐ 48 ☐ other ☐

We know that it can be difficult taking HIV medicines every day. We are interested in finding out what it is like for you. Being honest about whether or not you take your medicines may help others in the future. Please tick the answer that best describes what is happening to you. Please only think about the HIV medicines you are taking, and not any other medicines. Thank you for your help

1. Some people find that they forget to take their medicines at the weekend. Did you miss <u>any</u> of your HIV medicines last weekend (Friday, Saturday or Sunday)?
   Yes ☐
   No ☐

2. When was the last time you missed <u>any</u> HIV medicines? *(please tick one box)*
   Within the last week ☐
   1-2 weeks ago ☐
   3-4 weeks ago ☐
   1-3 months ago ☐
   Not at all since last visit ☐

3. Some people find that they aren't able to take medicines every day. Have you missed <u>all</u> of your HIV medicines for two days in a row or more since your last clinic visit?
   Yes ☐
   No ☐

4. Please mark on the line below the amount of HIV medicine doses you have taken since your last clinic visit.
   e.g. 0% would mean "I haven't taken any of my HIV medicine doses" and 100% would mean "I have taken all of my HIV medicine doses"

   0% (None) — 10% — 20% — 30% — 40% — 50% — 60% — 70% — 80% — 90% — 100% (All)

5. Did you complete this form on your own?
   Yes ☐
   No ☐
   If No, who else was involved? (e.g parent, nurse, friend) ____________

Thank you for taking the time to complete this form. Please add any comments that you may have here:

Carers
Continuous
Follow-up

BREATHER

ADHERENCE QUESTIONNAIRE FOR CARERS
Continuous group – FOLLOW-UP

**Trial number:** __ __ __ __ __ __ __ __ __ ☐☐☐☐			Date of birth: / /
**Male** ☐ **Female** ☐	Date of assessment: / /	Week no: *(tick one)*	4 ☐ 12 ☐ 24 ☐ 36 ☐ 48 ☐ other ☐

We know that it can be difficult giving HIV medicines to your child every day. We are interested in finding out what it is like for you and your family. Being honest about whether or not your child takes their medicines may help others in the future. Please tick the answer that best describes what is happening as far as you know. Please only think about the HIV medicines your child is taking, and not any other medicines.

If your child is responsible for taking their own medicines then you do not need to complete this questionnaire. Thank you for your help.

1. **What is your relationship to the child?** ____________________

2. **Who gives medicines to your child?** (eg. you/ your partner/ other family member) ____________________

3. **Some people find that they forget to take their medicines at the weekend. Did your child miss any of their HIV medicine doses last weekend (Friday, Saturday or Sunday)?**
   Yes ☐
   No ☐

4. **When was the last time your child missed any HIV medicine doses?** *(please tick one box)*
   Within the last week ☐
   1-2 weeks ago ☐
   3-4 weeks ago ☐
   1-3 months ago ☐
   Not at all since last visit ☐

5. **Some people find that they aren't able to take medicines every day. Has your child missed all their HIV medicines for two days in a row or more since their clinic last visit?**
   Yes ☐
   No ☐

6. **Please mark on the line below the amount of HIV medicine your child has taken since their last clinic visit.**
   e.g. 0% would mean "They haven't taken any of their HIV medicine doses" and 100% would mean "They have taken all of their HIV medicine doses"

   0% (None) – 10% – 20% – 30% – 40% – 50% – 60% – 70% – 80% – 90% – 100% (All)

7. **Did you complete this form on your own?**
   Yes ☐
   NO ☐
   If No, who else was involved? (e.g parent, nurse, friend) ____________________

Thank you for taking the time to complete this form. Please add any comments that you may have here:

APPENDIX 5: ACCEPTABILITY QUESTIONNAIRES

For **carers** to complete:

I. Acceptability questionnaire **for carers** for young people of the SCT group: randomisation (week0)

II. Acceptability questionnaire **for carers** for young people of the SCT group: on re-starting continuous ART or at end of study

For **participants** to complete:

III. Acceptability questionnaire **for young people** in the SCT group: randomisation (week0)

IV. Acceptability questionnaire **for young people** in the SCT group: on re-starting continuous ART or at end of study

Acceptability questionnaire for carers for young people of the SCT group: randomisation (week0)

Acceptability
Weekends off
Trial Entry
Carer

BREATHER

ACCEPTABILITY QUESTIONNAIRE FOR CARERS
Weekends-off Group – TRIAL ENTRY

**Trial number:** _ _ _ _ _ _ _ _ _ ☐☐☐☐		**Date of birth:** / /
**Male** ☐ **Female** ☐	**Date of assessment:** / /	

Your child has enrolled in the BREATHER study. This study will compare two different ways of giving HIV medicines; either taking the medicines every day, or having weekends off.

Please complete this questionnaire to tell us how you feel about your child having weekends off HIV medicines.
If your child is responsible for taking their own medicines then you do not need to complete this questionnaire.

1. **What is your relationship to the child?** ______________________

2. **Who gives medicines to your child? (eg. you/ your partner/ other family member)** ______________________

3. **Have any of the following been a problem with giving your child medicines in the last year? (Please tick all that apply)**

Remembering to take medicines	☐	Side-effects of medicines (eg. diarrhoea, feeling sick)	☐
Timing of medicines	☐	Different routine - weekends	☐
Number of tablets/capsules	☐	Different routine - weekdays	☐
Size of tablets/capsules	☐	School/college days	☐
Difficulty swallowing	☐	School/college holidays	☐
Amount of syrup	☐	Staying with friends or other family members	☐
Taste of medicines	☐	Going out with friends	☐

Other: ______________________

4. **How do you think stopping medicines at weekends will make things <u>for your child</u> compared to giving medicines all the time?**
   - A lot easier ☐
   - A little easier ☐
   - No difference ☐
   - A little more difficult ☐
   - A lot more difficult ☐

5. **How do you think stopping medicines at weekends will make things <u>for you, and the rest of the family</u> compared to giving medicines all the time?**
   - A lot easier ☐
   - A little easier ☐
   - No difference ☐
   - A little more difficult ☐
   - A lot more difficult ☐

6. **Did you complete this questionnaire alone?** Yes ☐ NO ☐

   If no, who else was involved? (e.g. nurse, doctor, child) ______________________

Thank you for taking the time to complete this questionnaire. Please add any comments that you may have here or on the back of this sheet:

Acceptability questionnaire for carers for young people of the SCT group: on re-starting continuous ART or at end of study

Acceptability
Weekends off
Re-start/trial end
Carer

BREATHER

ACCEPTABILITY QUESTIONNAIRE FOR CARERS
Weekends-off Group - RE-STARTING CONTINUOUS ART OR AT END OF STUDY

Version 1.5
July 2011

**Trial number:** __ __ __ __ __ __ __ __ __ ☐☐☐☐		Date of birth: / /
**Male** ☐ **Female** ☐	Date of assessment: / /	
Please tick one: Re-starting continuous ART ☐ Final visit ☐		

Thank you for taking part in the BREATHER study. This study was comparing two different ways of giving HIV medicines; either taking the medicines all of the time, or having weekends off.

Please complete this questionnaire to tell us how you feel about your child having weekends off HIV medicines.
If your child is responsible for taking their own medicines then you do not need to complete this questionnaire.

1. What is your relationship to the child? ______________________

2. Who gives medicines to your child? (eg. you/ your partner/ other family member) ______________________

3. How did stopping medicines at weekends make things for your child, compared to giving medicines all the time?
- A lot easier ☐
- A little easier ☐
- No difference ☐
- A little more difficult ☐
- A lot more difficult ☐
- I don't know ☐

4. How did stopping medicines at weekends make things for you and the rest of your family, compared to giving medicines all the time?
- A lot easier ☐
- A little easier ☐
- No difference ☐
- A little more difficult ☐
- A lot more difficult ☐

5a. When having weekends off medicine, did your child find any difference in taking medicines immediately before the weekend compared to taking medicines all the time?
- No difference ☐
- More difficult before the study ☐
- More difficult during the study ☐
- I don't know ☐

5b. When having weekends off medicine, did your child find any difference in taking medicines immediately after the weekend compared to taking medicines all the time?
- No difference ☐
- More difficult before the study ☐
- More difficult during the study ☐
- I don't know ☐

6a. When having weekends off medicine, did you find any difference in giving medicines immediately before the weekend compared to giving medicines all the time?
- No difference ☐
- More difficult before the study ☐
- More difficult during the study ☐

6b. When having weekends off medicine, did you find any difference in giving medicines immediately after the weekend compared to giving medicines all the time?
- No difference ☐
- More difficult before the study ☐
- More difficult during the study ☐

Please turn over

7. Overall, was there any difference giving medicines before the study compared to during the study?

- No difference ☐
- More difficult before the study ☐
- More difficult during the study ☐

8. During the study, were any of the following a problem with giving medicines (Please tick all that apply)?

Remembering to take medicines	☐	More clinic visits	☐
Timing of medicines	☐	Different routine - weekends	☐
Number of tablets/capsules	☐	Different routine - weekdays	☐
Size of tablets/capsules	☐	School/college days	☐
Difficulty swallowing	☐	School/college holidays	☐
Amount of syrup	☐	Staying with friends or other family members	☐
Taste of medicines	☐	Going out with friends	☐
Side-effects of medicines (eg. diarrhoea, feeling sick)	☐	Your child did not want to start medicines again	☐
Your child felt ill during the weekend	☐	You felt anxious about your child not taking medicines	☐
Your child felt ill after restarting	☐	Remembering to restart after the weekend	☐
Your child had more illnesses	☐	Other:______________________________	

9. Overall, how did your child feel before the study compared to during the study?

- No difference ☐
- Better before the study than during it ☐
- Better during the study than before it ☐
- I don't know ☐

10. Would you be happy for your child to have further weekends off medicines, if your doctor said they could?

- Yes ☐
- No ☐
- Not sure ☐

11. Did you complete this questionnaire alone?

- Yes ☐
- No ☐

If no, who else was involved?
(e.g. nurse, doctor, child)

____

Thank you for taking the time to complete this questionnaire. Please add any comments that you may have here:

Acceptability questionnaire for young people in the SCT group: randomisation (week0)

Acceptability
Weekends off
Trial Entry
Young People

BREATHER

ACCEPTABILITY QUESTIONNAIRE FOR YOUNG PEOPLE
Weekends-off Group – TRIAL ENTRY

**Trial number:** __ __ __ __ __ __ __ __ __ ☐☐☐☐		Date of birth: / /
**Male** ☐ **Female** ☐	Date of assessment: / /	

You have enrolled in the BREATHER study. This study will compare two different ways of taking HIV medicines; either taking the medicines every day, or having weekends off.

Please complete this questionnaire to tell us how you feel about having weekends off HIV medicines.

1. **Who gives you your medicines?** (eg. you, parent, guardian) ____________________

2. **Have any of the following been a problem with taking your medicines in the last year?** (Please tick all that apply)

Remembering to take medicines	☐	Side-effects of medicines (eg. diarrhoea, feeling sick)	☐
Timing of medicines	☐	Different routine - weekends	☐
Number of tablets/capsules	☐	Different routine - weekdays	☐
Size of tablets/capsules	☐	School/college days	☐
Difficulty swallowing	☐	School/college holidays	☐
Amount of syrup	☐	Staying with friends or other family members	☐
Taste of medicines	☐	Going out with friends	☐

Other:____

3. **How do you think stopping medicines at weekends will make things for you compared to taking medicines all the time?**
   - A lot easier ☐
   - A little easier ☐
   - No difference ☐
   - A little more difficult ☐
   - A lot more difficult ☐

4. **Did you complete this questionnaire alone?** Yes ☐ No ☐

   If no, who else was involved? (e.g. nurse, doctor, guardian) ____________________

Thank you for taking the time to complete this questionnaire. Please add any comments that you may have here:

Acceptability questionnaire for young people in the SCT group: on re-starting continuous ART or at end of study

Acceptability
Weekends off
Re-start/trial end
Young People

BREATHER

ACCEPTABILITY QUESTIONNAIRE FOR YOUNG PEOPLE
Weekends-off Group – RE-STARTING CONTINUOUS ART OR AT END OF STUDY

Version 1.5
July 2011

**Trial number:** __ __ __ __ __ __ __ __ __ ☐☐☐☐		Date of birth: / /
**Male** ☐ **Female** ☐	Date of assessment: / /	
Please tick one: Re-starting continuous ART ☐ Final visit ☐		

Thank you for taking part in the BREATHER study. This study was comparing two different ways of taking HIV medicines; either taking the medicines every day, or having weekends off.

Please complete this questionnaire to tell us how you feel about having weekends off HIV medicines.

1. Who gives you your medicines? (eg. you, parent, guardian) ______________________________

2. How did stopping medicines at weekends make things for you compared to taking medicines all the time?

- A lot easier ☐
- A little easier ☐
- No difference ☐
- A little more difficult ☐
- A lot more difficult ☐

3a. When having weekends off medicine, did you find any difference in taking medicines immediately before the weekend compared to taking medicines all the time?

- No difference ☐
- More difficult before the study ☐
- More difficult during the study ☐

3b. When having weekends off medicine, did you find any difference in taking medicines immediately after the weekend compared to taking medicines all the time?

- No difference ☐
- More difficult before the study ☐
- More difficult during the study ☐

4. Overall, was there any difference for you in the ease of taking medicines before the study compared to during the study?

- No difference ☐
- More difficult before the study ☐
- More difficult during the study ☐

5. During the study, were any of the following a problem with taking your medicines (Please tick all that apply)?

Remembering to take medicines	☐	More clinic visits	☐
Timing of medicines	☐	Different routine - weekends	☐
Number of tablets/capsules	☐	Different routine - weekdays	☐
Size of tablets/capsules	☐	School/college days	☐
Difficulty swallowing	☐	School/college holidays	☐
Amount of syrup	☐	Staying with friends or other family members	☐
Taste of medicines	☐	Going out with friends	☐
Side-effects of medicines (eg. diarrhoea, feeling sick)	☐	You did not want to start medicines again	☐
You felt ill during the weekend	☐	You felt anxious about not taking medicines	☐
You felt ill after restarting	☐	Remembering to restart after the weekend	☐
You had more illnesses	☐	Other:______________________	

Please turn over

6. Overall, how did you feel before the study compared to during the study?

- No difference ☐
- Better before the study than during it ☐
- Better during the study than before it ☐

7. Would you be happy to have further weekends off medicines if your doctor said you could?

- Yes ☐
- No ☐
- Not sure ☐

8. Did you complete this questionnaire alone?

- Yes ☐
- No ☐

If no, who else was involved?
(e.g. nurse, doctor, guardian)

____

Thank you for taking the time to complete this questionnaire. Please add any comments that you may have here:

APPENDIX 6: BREATHER – QUALITY OF LIFE (PEDSQL™) QUESTIONNAIRES

I. Young adult report (18-25 years)
II. Parent/carer report for Teens (13 to 18 years)
III. Parent/carer report for children (8 to 12 years)
IV. Teenager report (13 to 18 years)
V. Child report (8 to 12 years)

ID# ______________________

Date:______________________

PedsQL™

Paediatric Quality of Life Inventory

Version 4.0 - UK English

YOUNG ADULT REPORT (ages **18-25**)

DIRECTIONS

On the following page is a list of things that might be a problem for you. Please tell us **how much of a problem** each one has been for you during the **past ONE month** by circling:

0 if it is **never** a problem
1 if it is **almost never** a problem
2 if it is **sometimes** a problem
3 if it is **often** a problem
4 if it is **almost always** a problem

There are no right or wrong answers.
If you do not understand a question, please ask for help.

*In the **past ONE month**, how much of a **problem** has this been for you …*

About My Health and Activities *(PROBLEMS WITH…)*	Never	Almost Never	Some-times	Often	Almost Always
**1.** It is hard for me to walk more than a couple of streets (about 100 metres)	0	1	2	3	4
**2.** It is hard for me to run	0	1	2	3	4
**3.** It is hard for me to do sports activities or exercise	0	1	2	3	4
**4.** It is hard for me to lift heavy things	0	1	2	3	4
**5.** It is hard for me to have a bath or shower by myself	0	1	2	3	4
**6.** It is hard for me to do chores around the house	0	1	2	3	4
**7.** I have aches and pains	0	1	2	3	4
**8.** I feel tired	0	1	2	3	4

About My Feelings *(PROBLEMS WITH…)*	Never	Almost Never	Some-times	Often	Almost Always
**1.** I feel afraid or scared	0	1	2	3	4
**2.** I feel sad	0	1	2	3	4
**3.** I feel angry	0	1	2	3	4
**4.** I have trouble sleeping	0	1	2	3	4
**5.** I worry about what will happen to me	0	1	2	3	4

How I Get Along with Others *(PROBLEMS WITH…)*	Never	Almost Never	Some-times	Often	Almost Always
**1.** I have trouble getting along with other young adults	0	1	2	3	4
**2.** Other young adults do not want to be friends with me	0	1	2	3	4
**3.** Other young adults tease me	0	1	2	3	4
**4.** I cannot do things that others my age can do	0	1	2	3	4
**5.** It is hard to keep up with other people my age	0	1	2	3	4

About My Work/Studies *(PROBLEMS WITH…)*	Never	Almost Never	Some-times	Often	Almost Always
**1.** It is hard to pay attention at work or college	0	1	2	3	4
**2.** I forget things	0	1	2	3	4
**3.** I have trouble keeping up with my work or studies	0	1	2	3	4
**4.** I miss work or college because of not feeling well	0	1	2	3	4
**5.** I miss work or college to go to the doctor or hospital	0	1	2	3	4

ID#______________________

Date:____________________

PedsQL™

Paediatric Quality of Life Inventory

Version 4.0 - UK English

PARENT REPORT for **TEENS** (ages **13-18**)

DIRECTIONS

On the following page is a list of things that might be a problem for **your teen**. Please tell us **how much of a problem** each one has been for **your teen** during the **past ONE month** by circling:

0 if it is **never** a problem
1 if it is **almost never** a problem
2 if it is **sometimes** a problem
3 if it is **often** a problem
4 if it is **almost always** a problem

There are no right or wrong answers.
If you do not understand a question, please ask for help.

*In the past **ONE month,** how much of a **problem** has your teen had with*

Physical Functioning *(PROBLEMS WITH...)*	Never	Almost Never	Some-times	Often	Almost Always
1. Walking 100 metres	0	1	2	3	4
2. Running	0	1	2	3	4
3. Participating in sports activities or exercise	0	1	2	3	4
4. Lifting something heavy	0	1	2	3	4
5. Taking a bath or shower by him or herself	0	1	2	3	4
6. Doing chores around the house	0	1	2	3	4
7. Having aches or pains	0	1	2	3	4
8. Low energy levels	0	1	2	3	4

Emotional Functioning *(PROBLEMS WITH...)*	Never	Almost Never	Some-times	Often	Almost Always
1. Feeling afraid or scared	0	1	2	3	4
2. Feeling sad	0	1	2	3	4
3. Feeling angry	0	1	2	3	4
4. Trouble sleeping	0	1	2	3	4
5. Worrying about what will happen to him or her	0	1	2	3	4

Social Functioning *(PROBLEMS WITH...)*	Never	Almost Never	Some-times	Often	Almost Always
1. Getting on with other teens	0	1	2	3	4
2. Other teens not wanting to be his or her friend	0	1	2	3	4
3. Getting teased by other teens	0	1	2	3	4
4. Not being able to do things that other teens his or her age can do	0	1	2	3	4
5. Keeping up with other teens	0	1	2	3	4

School Functioning *(PROBLEMS WITH...)*	Never	Almost Never	Some-times	Often	Almost Always
1. Paying attention in class	0	1	2	3	4
2. Forgetting things	0	1	2	3	4
3. Keeping up with schoolwork	0	1	2	3	4
4. Missing school because of not feeling well	0	1	2	3	4
5. Missing school to go to the doctor or hospital	0	1	2	3	4

ID#______________________

Date:______________________

PedsQL™

Paediatric Quality of Life Inventory

Version 4.0 - UK English

PARENT REPORT for **CHILDREN** (ages **8-12**)

DIRECTIONS

On the following page is a list of things that might be a problem for **your child**. Please tell us **how much of a problem** each one has been for **your child** during the **past ONE month** by circling:

0 if it is **never** a problem
1 if it is **almost never** a problem
2 if it is **sometimes** a problem
3 if it is **often** a problem
4 if it is **almost always** a problem

There are no right or wrong answers.
If you do not understand a question, please ask for help.

*In the past **ONE month,** how much of a **problem** has your child had with*

Physical Functioning *(PROBLEMS WITH…)*	Never	Almost Never	Some-times	Often	Almost Always
1. Walking 100 metres	0	1	2	3	4
2. Running	0	1	2	3	4
3. Participating in sports activities or exercise	0	1	2	3	4
4. Lifting something heavy	0	1	2	3	4
5. Taking a bath or shower by him or herself	0	1	2	3	4
6. Doing chores around the house	0	1	2	3	4
7. Having aches or pains	0	1	2	3	4
8. Low energy levels	0	1	2	3	4

Emotional Functioning *(PROBLEMS WITH…)*	Never	Almost Never	Some-times	Often	Almost Always
1. Feeling afraid or scared	0	1	2	3	4
2. Feeling sad	0	1	2	3	4
3. Feeling angry	0	1	2	3	4
4. Trouble sleeping	0	1	2	3	4
5. Worrying about what will happen to him or her	0	1	2	3	4

Social Functioning *(PROBLEMS WITH…)*	Never	Almost Never	Some-times	Often	Almost Always
1. Getting on with other children	0	1	2	3	4
2. Other children not wanting to be his or her friend	0	1	2	3	4
3. Getting teased by other children	0	1	2	3	4
4. Not being able to do things that other children his or her age can do	0	1	2	3	4
5. Keeping up when playing with other children	0	1	2	3	4

School Functioning *(PROBLEMS WITH…)*	Never	Almost Never	Some-times	Often	Almost Always
1. Paying attention in class	0	1	2	3	4
2. Forgetting things	0	1	2	3	4
3. Keeping up with schoolwork	0	1	2	3	4
4. Missing school because of not feeling well	0	1	2	3	4
5. Missing school to go to the doctor or hospital	0	1	2	3	4

ID# ______________________

Date:______________________

PedsQL™

Paediatric Quality of Life Inventory

Version 4.0 - UK English

TEENAGER REPORT (ages **13-18**)

DIRECTIONS

On the following page is a list of things that might be a problem for you. Please tell us **how much of a problem** each one has been for you during the **PAST MONTH** by circling:

0 if it is **never** a problem
1 if it is **almost never** a problem
2 if it is **sometimes** a problem
3 if it is **often** a problem
4 if it is **almost always** a problem

There are no right or wrong answers.
If you do not understand a question, please ask for help.

*In the **PAST MONTH**, how much of a **problem** has this been for you …*

About My Health and Activities *(PROBLEMS WITH…)*	Never	Almost Never	Some-times	Often	Almost Always
1. It is hard for me to walk more than a couple of streets (about 100 metres)	0	1	2	3	4
2. It is hard for me to run	0	1	2	3	4
3. It is hard for me to do sports activities or exercise	0	1	2	3	4
4. It is hard for me to lift heavy things	0	1	2	3	4
5. It is hard for me to have a bath or shower by myself	0	1	2	3	4
6. It is hard for me to do chores around the house	0	1	2	3	4
7. I have aches and pains	0	1	2	3	4
8. I feel tired	0	1	2	3	4

About My Feelings *(PROBLEMS WITH…)*	Never	Almost Never	Some-times	Often	Almost Always
1. I feel afraid or scared	0	1	2	3	4
2. I feel sad	0	1	2	3	4
3. I feel angry	0	1	2	3	4
4. I have trouble sleeping	0	1	2	3	4
5. I worry about what will happen to me	0	1	2	3	4

How I Get On with Others *(PROBLEMS WITH…)*	Never	Almost Never	Some-times	Often	Almost Always
1. I have trouble getting on with other teenagers	0	1	2	3	4
2. Other teenagers do not want to be my friend	0	1	2	3	4
3. Other teenagers tease me	0	1	2	3	4
4. I cannot do things that other teenagers my age can do	0	1	2	3	4
5. It is hard to keep up with other teenagers my age	0	1	2	3	4

About School / College *(PROBLEMS WITH…)*	Never	Almost Never	Some-times	Often	Almost Always
1. It is hard to pay attention in class	0	1	2	3	4
2. I forget things	0	1	2	3	4
3. I have trouble keeping up with my school / college work	0	1	2	3	4
4. I miss school / college because of not feeling well	0	1	2	3	4
5. I miss school / college to go to the doctor or hospital	0	1	2	3	4

ID# ______________________

Date:______________________

PedsQL™

Paediatric Quality of Life Inventory

Version 4.0 - UK English

CHILD REPORT (ages **8-12**)

DIRECTIONS

On the following page is a list of things that might be a problem for you. Please tell us **how much of a problem** each one has been for you during the **PAST MONTH** by circling:

0 if it is **never** a problem
1 if it is **almost never** a problem
2 if it is **sometimes** a problem
3 if it is **often** a problem
4 if it is **almost always** a problem

There are no right or wrong answers.
If you do not understand a question, please ask for help.

*In the **PAST MONTH**, how much of a **problem** has this been for you …*

About My Health and Activities *(PROBLEMS WITH…)*	Never	Almost Never	Some-times	Often	Almost Always
1. It is hard for me to walk more than a couple of streets (about 100 metres)	0	1	2	3	4
2. It is hard for me to run	0	1	2	3	4
3. It is hard for me to do sports activities or exercise	0	1	2	3	4
4. It is hard for me to lift heavy things	0	1	2	3	4
5. It is hard for me to have a bath or shower by myself	0	1	2	3	4
6. It is hard for me to do chores around the house	0	1	2	3	4
7. I have aches and pains	0	1	2	3	4
8. I feel tired	0	1	2	3	4

About My Feelings *(PROBLEMS WITH…)*	Never	Almost Never	Some-times	Often	Almost Always
1. I feel afraid or scared	0	1	2	3	4
2. I feel sad	0	1	2	3	4
3. I feel angry	0	1	2	3	4
4. I have trouble sleeping	0	1	2	3	4
5. I worry about what will happen to me	0	1	2	3	4

How I Get On with Others *(PROBLEMS WITH…)*	Never	Almost Never	Some-times	Often	Almost Always
1. I have trouble getting on with other children	0	1	2	3	4
2. Other children do not want to be my friend	0	1	2	3	4
3. Other children tease me	0	1	2	3	4
4. I cannot do things that other children my age can do	0	1	2	3	4
5. It is hard to keep up when I play with other children	0	1	2	3	4

About School *(PROBLEMS WITH…)*	Never	Almost Never	Some-times	Often	Almost Always
1. It is hard to pay attention in class	0	1	2	3	4
2. I forget things	0	1	2	3	4
3. I have trouble keeping up with my schoolwork	0	1	2	3	4
4. I miss school because of not feeling well	0	1	2	3	4
5. I miss school to go to the doctor or hospital	0	1	2	3	4

APPENDIX 7: BREATHER – SAMPLE DIARIES FOR RECORDING ANTIRETROVIRALS TAKEN

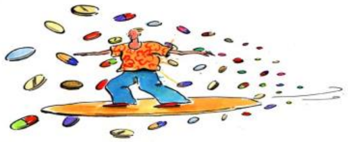

Sites will be able to choose the most appropriate diary for their participants.

Please fill out the dates under each day; along with the names of the drugs you are taking and the times you take them (please see example on next page).

Name: Joe Bloggs

Week beginning (date): 14th January 2008

Joe was randomised to the SCT arm and chose to NOT take anti-retroviral medications on Friday's and Saturday's throughout the trial. Here is his diary for the week commencing 14th Jan 2008..

	**MORNING: drug names and times**			**EVENING: drug names and times**			**COMMENTS**
	Efavirenz 08:30 am	Abacavir 08:30 am	Lamivudine 08:30 am	Efavirenz 9pm	Abacavir 9pm	Lamivudine 9pm	Issues, reasons for not taking drugs etc
**Monday Date:** *14/01/08*	☒**Yes** ☐ **NO**	☒ **Yes** ☐ **NO**	☒ **Yes** ☐ **NO**	☐ **Yes** ☒ **NO**	☒ **Yes** ☐ **NO**	☒ **Yes** ☐ **NO**	*EFV taken in morning only*
**Tuesday Date:** *15/01/08*	☒ **Yes** ☐ **NO**	☒ **Yes** ☐ **NO**	☒ **Yes** ☐ **NO**	☒ **Yes** ☐ **NO**	☒ **Yes** ☐ **NO**	☒ **Yes** ☐ **NO**	
**Wednesday Date:** *16/01/08*	☒ **Yes** ☐ **NO**	☒ **Yes** ☐ **NO**	☒ **Yes** ☐ **NO**	☒ **Yes** ☐ **NO**	☒ **Yes** ☐ **NO**	☒ **Yes** ☐ **NO**	
**Thursday Date:** *17/01/08*	☒ **Yes** ☐ **NO**	☒ **Yes** ☐ **NO**	☒ **Yes** ☐ **NO**	☒ **Yes** ☐ **NO**	☒ **Yes** ☐ **NO**	☒ **Yes** ☐ **NO**	
**Friday Date:** *18/01/08*	☐ **Yes** ☒ **NO**	☐ **Yes** ☒ **NO**	☐ **Yes** ☒ **NO**	☐ **Yes** ☒ **NO**	☐ **Yes** ☒ **NO**	☐ **Yes** ☒ **NO**	*SCT break*
**Saturday Date:** *19/01/08*	☐ **Yes** ☒ **NO**	☐ **Yes** ☒ **NO**	☐ **Yes** ☒ **NO**	☐ **Yes** ☒ **NO**	☐ **Yes** ☒ **NO**	☐ **Yes** ☒ **NO**	*SCT break*
**Sunday Date:** *20/01/08*	☒ **Yes** ☐ **NO**	☒ **Yes** ☐ **NO**	☒ **Yes** ☐ **NO**	☒ **Yes** ☐ **NO**	☒ **Yes** ☐ **NO**	☒ **Yes** ☐ **NO**	

BREATHER STUDY DIARY

- Fill in the month in the space given, and add the dates in to the small boxes.
- Add the time you took your doses on each day and put a cross (x) if you didn't take your doses that day in the big box.
- E.g. if you took the morning dose but not the evening dose write 09:00, x
- Anything different or unusual e.g. you forgot to take one of your doses, make a note in the comments box on the back page

BREATHER NUMBER:

NAME:

Please add any comments you have here:

MONTH(S): September/October/November

MON	TUES	WED	THURS	FRI	SAT	SUN
		1<br>08:30, x	2<br>08:30, 20:30	3<br>08:25, 20:35	4<br>x x	5<br>x x
6<br>08:20, 20:40	7<br>08:22, 20:37	8<br>x, 20:00	9<br>08:15, 20:15	10<br>08:30, 20:30	11<br>x x	12<br>x x
13	14	15	16	17	18	19
20	21	22	23	24	25	26
27	28	29	30	1	2	3
4	5	6	7	8	9	10
11	12	13	14	15	16	17
18	19	20	21	22	23	24
25	26	27	28	29	30	31
1	2	3	4	5	6	7
8	9	10	11	12	13	14
15	16	17	18	19	20	21
22	23	24				

APPENDIX 8: CDC CLASSIFICATIONS

CDC 1994 REVISED CLASSIFICATION SYSTEM FOR HIV-INFECTION IN CHILDREN (MMWR 1994; 43 [RR-12]:1-10)

Clinical Categories for Children with Human Immunodeficiency Virus (HIV) Infection

CATEGORY N: NOT SYMPTOMATIC

Children who have no signs or symptoms considered to be the result of HIV infection or who have only one of the conditions listed in Category A.

CATEGORY A: MILDLY SYMPTOMATIC

Children with two or more of the conditions listed below but none of the conditions listed in Categories B and C.

- Lymphadenopathy ($\geq$ 0.5 cm at more than two sites; bilateral = one site)
- Hepatomegaly
- Splenomegaly
- Dermatitis
- Parotitis
- Recurrent or persistent upper respiratory infection, sinusitis, or otitis media

CATEGORY B: MODERATELY SYMPTOMATIC

Children who have symptomatic conditions other than those listed for Category A or C that are attributed to HIV infection. Examples of conditions in clinical Category B include but are not limited to:

- Anaemia (< 8 g/dL), neutropaenia (< 1.0 x 10^9/l), or thrombocytopaenia (<100 x 10^9/l) persisting $\geq$ 30 days
- Bacterial meningitis, pneumonia, or sepsis (single episode)
- Candidiasis, oropharyngeal (thrush), persisting (> 2 months) in children > 6 months of age
- Cardiomyopathy
- Cytomegalovirus infection, with onset before 1 month of age
- Diarrhoea, recurrent or chronic
- Hepatitis
- Herpes simplex virus (HSV) stomatitis, recurrent (more than two episodes within 1 year)
- HSV bronchitis, pneumonitis, or oesophagitis with onset before 1 month of age
- Herpes zoster (shingles) involving at least two distinct episodes or more than one dermatome
- Leimyosarcoma
- Lymphoid interstitial pneumonia (LIP) or pulmonary lymphoid hyperplasia complex
- Nephropathy
- Nocardiosis
- Persistent fever (lasting > 1 month)
- Toxoplasmosis, onset before 1 month of age
- Varicella, disseminated (complicated chickenpox)

CATEGORY C: SEVERELY SYMPTOMATIC*

Serious bacterial infections, multiple or recurrent (i.e., any combination of at least two culture-confirmed infections within a 2-year period), of the following types: septicaemia, pneumonia, meningitis, bone or joint infection, or abscess of an internal organ or body cavity (excluding otitis media, superficial skin or mucosal abscesses, and indwelling catheter-related infections)

- Candidiasis, oesophageal or pulmonary (bronchi, trachea, lungs)
- Coccidioidomycosis, disseminated (at site other than or in addition to lungs or cervical or hilar lymph nodes)
- Cryptococcosis, extrapulmonary
- Cryptosporidiosis or isosporiasis with diarrhoea persisting > 1 month
- Cytomegalovirus disease with onset of symptoms at age > 1 month (at a site other than liver, spleen, or lymph nodes)
- Encephalopathy (at least one of the following progressive findings present for at least 2 months in the absence of a concurrent illness other than HIV infection that could explain the findings):
  a) failure to attain or loss of developmental milestones or loss of intellectual ability, verified by standard developmental scale or neuropsychological tests;
  b) impaired brain growth or acquired microcephaly demonstrated by head circumference measurements or brain atrophy demonstrated by computerised tomography or magnetic resonance imaging (serial imaging is required for children < 2 years of age);
  c) acquired symmetric motor deficit manifested by two or more of the following: paresis, pathological reflexes, ataxia, or gait disturbance
- Herpes simplex virus infection causing a mucocutaneous ulcer that persists for > 1 month; or bronchitis, pneumonitis, or oesophagitis for any duration affecting a child > 1 month of age
- Histoplasmosis, disseminated (at a site other than or in addition to lungs or cervical or hilar lymph nodes)
- Kaposi's sarcoma
- Lymphoma, primary, in brain
- Lymphoma, small, noncleaved cell (Burkitt's), or immunoblastic or large cell lymphoma of B-cell or unknown immunological phenotype
- *Mycobacterium tuberculosis*, disseminated or extrapulmonary
- *Mycobacterium*, other species or unidentified species, disseminated (at a site other than or in addition to lungs, skin, or cervical or hilar lymph nodes)
- *Mycobacterium avium* complex or Mycobacterium kansasii, disseminated (at site other than or in addition to lungs, skin, or cervical or hilar lymph nodes)
- *Pneumocystis jiroveci* pneumonia (formerly *carinii*)
- Progressive multifocal leukoencephalopathy
- Salmonella (nontyphoid) septicaemia, recurrent
- Toxoplasmosis of the brain with onset at > 1 month of age
- Wasting syndrome in the absence of a concurrent illness other than HIV infection that could explain the following findings:
  a) persistent weight loss > 10% of baseline OR
  b) downward crossing of at least two of the following percentile lines on the weight-for-age chart (e.g., 95th, 75th, 50th, 25th, 5th) in a child ≥ 1 year of age OR
  c) < 5th percentile on weight-for-height chart on two consecutive measurements, ≥30 days apart PLUS a) chronic diarrhoea (i.e., at least two loose stools per day for ≥30 days) OR b) documented fever (for ≥ 30 days, intermittent or constant)

Definitive diagnosis: microscopy (histology or cytology); culture; antigen detection.
Presumptive diagnosis: characteristic clinical presentation, supported by investigations other than microscopy or culture and after exclusion of other causes in the differential diagnosis.

APPENDIX 9: TOXICITY GRADINGS

(Adapted from National Institute of Health (Division of AIDs USA) toxicity table for grading severity of adult and paediatric adverse events (December, 2004) Clarification August 2009)

CLINICAL				
**PARAMETER**	**GRADE 1 MILD**	**GRADE 2 MODERATE**	**GRADE 3 SEVERE**	**GRADE 4 POTENTIALLY LIFE-THREATENING**
**ESTIMATING SEVERITY GRADE**				
Clinical adverse event NOT identified elsewhere in this DAIDS AE grading table	Symptoms causing no or minimal interference with usual social & functional activities	Symptoms causing greater than minimal interference with usual social & functional activities	Symptoms causing inability to perform usual social & functional activities	Symptoms causing inability to perform basic self-care functions OR Medical or operative intervention indicated to prevent permanent impairment, persistent disability, or death
**SYSTEMIC**				
Acute systemic allergic reaction	Localized urticaria (wheals) with no medical intervention indicated	Localized urticaria with medical intervention indicated OR Mild angioedema with no medical intervention indicated	Generalized urticaria OR Angioedema with medical intervention indicated OR Symptomatic mild bronchospasm	Acute anaphylaxis OR Life-threatening bronchospasm OR laryngeal edema
Chills	Symptoms causing no or minimal interference with usual social & functional activities	Symptoms causing greater than minimal interference with usual social & functional activities	Symptoms causing inability to perform usual social & functional activities	NA
Fatigue<br>Malaise	Symptoms causing no or minimal interference with usual social & functional activities	Symptoms causing greater than minimal interference with usual social & functional activities	Symptoms causing inability to perform usual social & functional activities	Incapacitating fatigue/ malaise symptoms causing inability to perform basic self-care functions
Fever (nonaxillary)	37.7 – 38.6°C	38.7 – 39.3°C	39.4 – 40.5°C	> 40.5°C
Pain (indicate body site)<br>DO NOT use for pain due to injection (See Injection Site Reactions: Injection site pain)<br>See also Headache, Arthralgia, and Myalgia	Pain causing no or minimal interference with usual social & functional activities	Pain causing greater than minimal interference with usual social & functional activities	Pain causing inability to perform usual social & functional activities	Disabling pain causing inability to perform basic self-care functions OR Hospitalization (other than emergency room visit) indicated
Unintentional weight loss	NA	5 – 9% loss in body weight from baseline	10 – 19% loss in body weight from baseline	≥ 20% loss in body weight from baseline OR Aggressive intervention indicated [e.g., tube feeding or total parenteral nutrition (TPN)]

CLINICAL				
**PARAMETER**	**GRADE 1**<br>**MILD**	**GRADE 2**<br>**MODERATE**	**GRADE 3**<br>**SEVERE**	**GRADE 4**<br>**POTENTIALLY LIFE-THREATENING**
**INFECTION**				
Infection (any other than HIV infection)	Localized, no systemic antimicrobial treatment indicated AND Symptoms causing no or minimal interference with usual social & functional activities	Systemic antimicrobial treatment indicated OR Symptoms causing greater than minimal interference with usual social & functional activities	Systemic antimicrobial treatment indicated AND Symptoms causing inability to perform usual social & functional activities OR Operative intervention (other than simple incision and drainage) indicated	Life-threatening consequences (e.g., septic shock)
**INJECTION SITE REACTIONS**				
Injection site pain (pain without touching)<br>Or<br>Tenderness (pain when area is touched)	Pain/tenderness causing no or minimal limitation of use of limb	Pain/tenderness limiting use of limb OR Pain/tenderness causing greater than minimal interference with usual social & functional activities	Pain/tenderness causing inability to perform usual social & functional activities	Pain/tenderness causing inability to perform basic self-care function OR Hospitalization (other than emergency room visit) indicated for management of pain/tenderness
Injection site reaction (localized)				
**Adult > 15 years**	Erythema OR Induration of 5x5 cm – 9x9 cm (or 25 cm^2 – 81cm^2)	Erythema OR Induration OR Edema > 9 cm any diameter (or > 81 cm^2)	Ulceration OR Secondary infection OR Phlebitis OR Sterile abscess OR Drainage	Necrosis (involving dermis and deeper tissue)
**Paediatric ≤ 15 years**	Erythema OR Induration OR Edema present but ≤ 2.5 cm diameter	Erythema OR Induration OR Edema > 2.5 cm diameter but < 50% surface area of the extremity segment (e.g., upper arm/thigh)	Erythema OR Induration OR Edema involving ≥ 50% surface area of the extremity segment (e.g., upper arm/thigh) OR Ulceration OR Secondary infection OR Phlebitis OR Sterile abscess OR Drainage	Necrosis (involving dermis and deeper tissue)
Pruritis associated with injection<br>See also Skin: Pruritis (itching - no skin lesions)	Itching localized to injection site AND Relieved spontaneously or with < 48 hours treatment	Itching beyond the injection site but not generalized OR Itching localized to injection site requiring ≥ 48 hours treatment	Generalized itching causing inability to perform usual social & functional activities	NA
**SKIN – DERMATOLOGICAL**				
Alopecia	Thinning detectable by study participant (or by caregiver for young children and disabled adults)	Thinning or patchy hair loss detectable by health care provider	Complete hair loss	NA

CLINICAL				
**PARAMETER**	**GRADE 1 MILD**	**GRADE 2 MODERATE**	**GRADE 3 SEVERE**	**GRADE 4 POTENTIALLY LIFE-THREATENING**
Cutaneous reaction – rash	Localized macular rash	Diffuse macular, maculopapular, or morbilliform rash OR Target lesions	Diffuse macular, maculopapular, or morbilliform rash with vesicles or limited number of bullae OR Superficial ulcerations of mucous membrane limited to one site	Extensive or generalized bullous lesions OR Stevens-Johnson syndrome OR Ulceration of mucous membrane involving two or more distinct mucosal sites OR Toxic epidermal necrolysis (TEN)
Hyperpigmentation	Slight or localized	Marked or generalized	NA	NA
Hypopigmentation	Slight or localized	Marked or generalized	NA	NA
Pruritis (itching – no skin lesions)<br>(See also Injection Site Reactions: Pruritis associated with injection)	Itching causing no or minimal interference with usual social & functional activities	Itching causing greater than minimal interference with usual social & functional activities	Itching causing inability to perform usual social & functional activities	NA
**CARDIOVASCULAR**				
Cardiac arrhythmia (general)<br>(By ECG or physical exam)	Asymptomatic AND No intervention indicated	Asymptomatic AND Non-urgent medical intervention indicated	Symptomatic, non-life-threatening AND Non-urgent medical intervention indicated	Life-threatening arrhythmia OR Urgent intervention indicated
Cardiac-ischemia/infarction	NA	NA	Symptomatic ischemia (stable angina) OR Testing consistent with ischemia	Unstable angina OR Acute myocardial infarction
Hemorrhage (significant acute blood loss)	NA	Symptomatic AND No transfusion indicated	Symptomatic AND Transfusion of ≤ 2 units packed RBCs (for children ≤ 10 cc/kg) indicated	Life-threatening hypotension OR Transfusion of > 2 units packed RBCs (for children > 10 cc/kg) indicated
Hypertension				
**Adult > 17 years**<br>**(with repeat testing at same visit)**	140-159 mm Hg systolic<br>OR<br>90-99 mmHg diastolic	160-179 mm Hg systolic<br>OR<br>100-109 mmHg diastolic	≥ 180 mm Hg systolic<br>OR<br>≥ 110 mmHg diastolic	Life-threatening consequences (e.g., malignant hypertension)<br>OR Hospitalization indicated (other than emergency room visit)
**Paediatric ≤ 17 years**<br>(with repeat testing at same visit)	NA	91st – 94th percentile adjusted for age, height, and gender (systolic and/or diastolic)	≥ 95th percentile adjusted for age, height, and gender (systolic and/or diastolic)	Life-threatening consequences (e.g., malignant hypertension) OR Hospitalization indicated (other than emergency room visit)

CLINICAL				
**PARAMETER**	**GRADE 1 MILD**	**GRADE 2 MODERATE**	**GRADE 3 SEVERE**	**GRADE 4 POTENTIALLY LIFE-THREATENING**
Hypotension	NA	Symptomatic, corrected with oral fluid replacement	Symptomatic, IV fluids indicated	Shock requiring use of vasopressors or mechanical assistance to maintain blood pressure
Pericardial effusion	Asymptomatic, small effusion requiring no intervention	Asymptomatic, moderate or larger effusion requiring no intervention	Effusion with non-life threatening physiologic consequences OR Effusion with non-urgent intervention indicated	Life-threatening consequences (e.g., tamponade) OR Urgent intervention indicated
Prolonged PR interval				
**Adult > 16 years**	PR interval 0.21 – 0.25 sec	PR interval > 0.25 sec	Type II 2nd degree AV block OR Ventricular pause > 3.0 sec	Complete AV block
**Paediatric ≤ 16 years**	1st degree AV block (PR > normal for age and rate)	Type I 2nd degree AV block	Type II 2nd degree AV block	Complete AV block
Prolonged QTc				
**Adult > 16 years**	Asymptomatic, QTc interval 0.45 – 0.47 sec OR Increase interval < 0.03 sec above baseline	Asymptomatic, QTc interval 0.48 – 0.49 sec OR Increase in interval 0.03 – 0.05 sec above baseline	Asymptomatic, QTc interval ≥ 0.50 sec OR Increase in interval ≥ 0.06 sec above baseline	Life-threatening consequences, e.g. Torsade de pointes or other associated serious ventricular dysrhythmia
**Paediatric ≤ 16 years**	Asymptomatic, QTc interval 0.450 – 0.464 sec	Asymptomatic, QTc interval 0.465 – 0.479 sec	Asymptomatic, QTc interval ≥ 0.480 sec	Life-threatening consequences, e.g. Torsade de pointes or other associated serious ventricular dysrhythmia
Thrombosis/embolism	NA	Deep vein thrombosis AND No intervention indicated (e.g., anticoagulation, lysis filter, invasive procedure)	Deep vein thrombosis AND Intervention indicated (e.g., anticoagulation, lysis filter, invasive procedure)	Embolic event (e.g., pulmonary embolism, life-threatening thrombus)
Vasovagal episode (associated with a procedure of any kind)	Present without loss of consciousness	Present with transient loss of consciousness	NA	NA
Ventricular dysfunction (congestive heart failure)	NA	Asymptomatic diagnostic finding AND intervention indicated	New onset with symptoms OR Worsening symptomatic congestive heart failure	Life-threatening congestive heart failure
**GASTROINTESTINAL**				
Anorexia	Loss of appetite without decreased oral intake	Loss of appetite associated with decreased oral intake without significant weight loss	Loss of appetite associated with significant weight loss	Life-threatening consequences OR Aggressive intervention indicated [e.g., tube feeding or total parenteral nutrition (TPN)]

CLINICAL				
**PARAMETER**	**GRADE 1 MILD**	**GRADE 2 MODERATE**	**GRADE 3 SEVERE**	**GRADE 4 POTENTIALLY LIFE-THREATENING**
Ascites	Asymptomatic	Symptomatic AND Intervention indicated (e.g., diuretics or therapeutic paracentesis)	Symptomatic despite intervention	Life-threatening consequences
Cholecystitis	NA	Symptomatic AND Medical intervention indicated	Radiologic, endoscopic, or operative intervention indicated	Life-threatening consequences (e.g., sepsis or perforation)
Constipation	NA	Persistent constipation requiring regular use of dietary modifications, laxatives, or enemas	Obstipation with manual evacuation indicated	Life-threatening consequences (e.g., obstruction)
Diarrhea				
**Adult and Paediatric ≥ 1 year**	Transient or intermittent episodes of unformed stools OR Increase of ≤ 3 stools over baseline per 24-hour period	Persistent episodes of unformed to watery stools OR Increase of 4 – 6 stools over baseline per 24-hour period	Bloody diarrhea OR Increase of ≥ 7 stools per 24-hour period OR IV fluid replacement indicated	Life-threatening consequences (e.g., hypotensive shock)
Dysphagia-Odynophagia	Symptomatic but able to eat usual diet	Symptoms causing altered dietary intake without medical intervention indicated	Symptoms causing severely altered dietary intake with medical intervention indicated	Life-threatening reduction in oral intake
Mucositis/stomatitis (clinical exam)<br>Indicate site (e.g., larynx, oral)<br>See Genitourinary for Vulvovaginitis<br>See also Dysphagia-Odynophagia and Proctitis	Erythema of the mucosa	Patchy pseudomembranes or ulcerations	Confluent pseudomembranes or ulcerations OR Mucosal bleeding with minor trauma	Tissue necrosis OR Diffuse spontaneous mucosal bleeding OR Life-threatening consequences (e.g., aspiration, choking)
Nausea	Transient (< 24 hours) or intermittent nausea with no or minimal interference with oral intake	Persistent nausea resulting in decreased oral intake for 24 – 48 hours	Persistent nausea resulting in minimal oral intake for > 48 hours OR Aggressive rehydration indicated (e.g., IV fluids)	Life-threatening consequences (e.g., hypotensive shock)
Pancreatitis	NA	Symptomatic AND Hospitalization not indicated (other than emergency room visit)	Symptomatic AND Hospitalization indicated (other than emergency room visit)	Life-threatening consequences (e.g., circulatory failure, hemorrhage, sepsis)
Proctitis (functional-symptomatic)<br>Also see Mucositis/stomatitis for clinical exam	Rectal discomfort AND No intervention indicated	Symptoms causing greater than minimal interference with usual social & functional activities OR Medical intervention indicated	Symptoms causing inability to perform usual social & functional activities OR Operative intervention indicated	Life-threatening consequences (e.g., perforation)

CLINICAL				
**PARAMETER**	**GRADE 1 MILD**	**GRADE 2 MODERATE**	**GRADE 3 SEVERE**	**GRADE 4 POTENTIALLY LIFE-THREATENING**
Vomiting	Transient or intermittent vomiting with no or minimal interference with oral intake	Frequent episodes of vomiting with no or mild dehydration	Persistent vomiting resulting in orthostatic hypotension OR Aggressive rehydration indicated (e.g., IV fluids)	Life-threatening consequences (e.g., hypotensive shock)
**NEUROLOGIC**				
Alteration in personality-behavior or in mood (e.g., agitation, anxiety, depression, mania, psychosis)	Alteration causing no or minimal interference with usual social & functional activities	Alteration causing greater than minimal interference with usual social & functional activities	Alteration causing inability to perform usual social & functional activities	Behavior potentially harmful to self or others (e.g., suicidal and homicidal ideation or attempt, acute psychosis) OR Causing inability to perform basic self-care functions
Altered Mental Status<br>For Dementia, see Cognitive and behavioral/attentional disturbance (including dementia and attention deficit disorder)	Changes causing no or minimal interference with usual social & functional activities	Mild lethargy or somnolence causing greater than minimal interference with usual social & functional activities	Confusion, memory impairment, lethargy, or somnolence causing inability to perform usual social & functional activities	Delirium OR obtundation, OR coma
Ataxia	Asymptomatic ataxia detectable on exam OR Minimal ataxia causing no or minimal interference with usual social & functional activities	Symptomatic ataxia causing greater than minimal interference with usual social & functional activities	Symptomatic ataxia causing inability to perform usual social & functional activities	Disabling ataxia causing inability to perform basic self-care functions
Cognitive and behavioral/attentional disturbance (including dementia and attention deficit disorder)	Disability causing no or minimal interference with usual social & functional activities OR Specialized resources not indicated	Disability causing greater than minimal interference with usual social & functional activities OR Specialized resources on part-time basis indicated	Disability causing inability to perform usual social & functional activities OR Specialized resources on a full-time basis indicated	Disability causing inability to perform basic self-care functions OR Institutionalization indicated
CNS ischemia (acute)	NA	NA	Transient ischemic attack	Cerebral vascular accident (CVA, stroke) with neurological deficit
Developmental delay – **Paediatric ≤ 16 years**	Mild developmental delay, either motor or cognitive, as determined by comparison with a developmental screening tool appropriate for the setting	Moderate developmental delay, either motor or cognitive, as determined by comparison with a developmental screening tool appropriate for the setting	Severe developmental delay, either motor or cognitive, as determined by comparison with a developmental screening tool appropriate for the setting	Developmental regression, either motor or cognitive, as determined by comparison with a developmental screening tool appropriate for the setting

CLINICAL				
**PARAMETER**	**GRADE 1 MILD**	**GRADE 2 MODERATE**	**GRADE 3 SEVERE**	**GRADE 4 POTENTIALLY LIFE-THREATENING**
Headache	Symptoms causing no or minimal interference with usual social & functional activities	Symptoms causing greater than minimal interference with usual social & functional activities	Symptoms causing inability to perform usual social & functional activities	Symptoms causing inability to perform basic self-care functions OR Hospitalization indicated (other than emergency room visit) OR Headache with significant impairment of alertness or other neurologic function
Insomnia	NA	Difficulty sleeping causing greater than minimal interference with usual social & functional activities	Difficulty sleeping causing inability to perform usual social & functional activities	Disabling insomnia causing inability to perform basic self-care functions
Neuromuscular weakness (including myopathy & neuropathy)	Asymptomatic with decreased strength on exam OR Minimal muscle weakness causing no or minimal interference with usual social & functional activities	Muscle weakness causing greater than minimal interference with usual social & functional activities	Muscle weakness causing inability to perform usual social & functional activities	Disabling muscle weakness causing inability to perform basic self-care functions OR Respiratory muscle weakness impairing ventilation
Neurosensory alteration (including paresthesia and painful neuropathy)	Asymptomatic with sensory alteration on exam or minimal paresthesia causing no or minimal interference with usual social & functional activities	Sensory alteration or paresthesia causing greater than minimal interference with usual social & functional activities	Sensory alteration or paresthesia causing inability to perform usual social & functional activities	Disabling sensory alteration or paresthesia causing inability to perform basic self-care functions
Seizure: (new onset)<br>- **Adult ≥18 years**<br>See also Seizure:<br>(known pre-existing seizure disorder)	NA	1 seizure	2-4 seizures	Seizures of any kind which are prolonged, repetitive (e.g. , status epilepticus), or difficult to control (e.g. refractory epilepsy)
Seizure: (known pre-existing seizure disorder)<br>- **Adult ≥18 years**<br>For worsening of existing epilepsy the grades should be based on an increase from previous level of control to any of these levels	NA	Increased frequency of pre-existing seizures (non-repetitive) without change in seizure character OR<br>Infrequent break-through seizures while on stable medication in a previously controlled seizure disorder	Change in seizure character from baseline either in duration or quality (e.g., severity or focality)	Seizures of any kind which are prolonged, repetitive (e.g. , status epilepticus), or difficult to control (e.g. refractory epilepsy)
Seizure **– Paediatric < 18 years**	Seizure, generalized onset with or without secondary generalization, lasting < 5 minutes with < 24 hours post ictal state	Seizure, generalized onset with or without secondary generalization, lasting 5 – 20 minutes with < 24 hours post ictal state	Seizure, generalized onset with or without secondary generalization, lasting > 20 minutes	Seizure, generalized onset with or without secondary generalization, requiring intubation and sedation

CLINICAL				
**PARAMETER**	**GRADE 1 MILD**	**GRADE 2 MODERATE**	**GRADE 3 SEVERE**	**GRADE 4 POTENTIALLY LIFE-THREATENING**
Syncope (not associated with a procedure)	NA	Present	NA	NA
Vertigo	Vertigo causing no or minimal interference with usual social & functional activities	Vertigo causing greater than minimal interference with usual social & functional activities	Vertigo causing inability to perform usual social & functional activities	Disabling vertigo causing inability to perform basic self-care functions
**RESPIRATORY**				
Bronchospasm (acute)	FEV1 or peak flow reduced to 70 – 80%	FEV1 or peak flow 50 – 69%	FEV1 or peak flow 25 – 49%	Cyanosis OR FEV1 or peak flow < 25% OR Intubation
Dyspnea or respiratory distress				
**Adult ≥ 14 years**	Dyspnea on exertion with no or minimal interference with usual social & functional activities	Dyspnea on exertion causing greater than minimal interference with usual social & functional activities	Dyspnea at rest causing inability to perform usual social & functional activities	Respiratory failure with ventilatory support indicated
**Paediatric < 14 years**	Wheezing OR minimal increase in respiratory rate for age	Nasal flaring OR Intercostal retractions OR Pulse oximetry 90 – 95%	Dyspnea at rest causing inability to perform usual social & functional activities OR Pulse oximetry < 90%	Respiratory failure with ventilatory support indicated
**MUSCULOSKELETAL**				
Arthralgia<br>See also Arthritis	Joint pain causing no or minimal interference with usual social & functional activities	Joint pain causing greater than minimal interference with usual social & functional activities	Joint pain causing inability to perform usual social & functional activities	Disabling joint pain causing inability to perform basic self-care functions
Arthritis<br>See also Arthralgia	Stiffness or joint swelling causing no or minimal interference with usual social & functional activities	Stiffness or joint swelling causing greater than minimal interference with usual social & functional activities	Stiffness or joint swelling causing inability to perform usual social & functional activities	Disabling joint stiffness or swelling causing inability to perform basic self-care functions
Bone Mineral Loss				
**Paediatric < 21 years**	BMD z-score -2.5 to -1.0	BMD z-score < -2.5	Pathological fracture (including loss of vertebral height)	Pathologic fracture causing life-threatening consequences
Myalgia (non-injection site)	Muscle pain causing no or minimal interference with usual social & functional activities	Muscle pain causing greater than minimal interference with usual social & functional activities	Muscle pain causing inability to perform usual social & functional activities	Disabling muscle pain causing inability to perform basic self-care functions
Osteonecrosis	NA	Asymptomatic with radiographic findings AND No operative intervention indicated	Symptomatic bone pain with radiographic findings OR Operative intervention indicated	Disabling bone pain with radiographic findings causing inability to perform basic self-care functions

CLINICAL				
**PARAMETER**	**GRADE 1 MILD**	**GRADE 2 MODERATE**	**GRADE 3 SEVERE**	**GRADE 4 POTENTIALLY LIFE-THREATENING**
**GENITOURINARY**				
Cervicitis (symptoms)<br>(For use in studies evaluating topical study agents)<br>For other cervicitis see Infection: Infection (any other than HIV infection)	Symptoms causing no or minimal interference with usual social & functional activities	Symptoms causing greater than minimal interference with usual social & functional activities	Symptoms causing inability to perform usual social & functional activities	Symptoms causing inability to perform basic self-care functions
Cervicitis (clinical exam)<br>(For use in studies evaluating topical study agents)<br>For other cervicitis see Infection: Infection (any other than HIV infection)	Minimal cervical abnormalities on examination (erythema, mucopurulent discharge, or friability) OR Epithelial disruption < 25% of total surface	Moderate cervical abnormalities on examination (erythema, mucopurulent discharge, or friability) OR Epithelial disruption of 25 – 49% total surface	Severe cervical abnormalities on examination (erythema, mucopurulent discharge, or friability) OR Epithelial disruption 50 – 75% total surface	Epithelial disruption > 75% total surface
Inter-menstrual bleeding (IMB)	Spotting observed by participant OR Minimal blood observed during clinical or colposcopic examination	Inter-menstrual bleeding not greater in duration or amount than usual menstrual cycle	Inter-menstrual bleeding greater in duration or amount than usual menstrual cycle	Hemorrhage with life-threatening hypotension OR Operative intervention indicated
Urinary tract obstruction (e.g., stone)	NA	Signs or symptoms of urinary tract obstruction without hydronephrosis or renal dysfunction	Signs or symptoms of urinary tract obstruction with hydronephrosis or renal dysfunction	Obstruction causing life-threatening consequences
Vulvovaginitis (symptoms)<br>(Use in studies evaluating topical study agents)<br>For other vulvovaginitis see Infection: Infection (any other than HIV infection)	Symptoms causing no or minimal interference with usual social & functional activities	Symptoms causing greater than minimal interference with usual social & functional activities	Symptoms causing inability to perform usual social & functional activities	Symptoms causing inability to perform basic self-care functions

CLINICAL				
**PARAMETER**	**GRADE 1 MILD**	**GRADE 2 MODERATE**	**GRADE 3 SEVERE**	**GRADE 4 POTENTIALLY LIFE-THREATENING**
Vulvovaginitis (clinical exam) (Use in studies evaluating topical study agents) For other vulvovaginitis see Infection: Infection (any other than HIV infection)	Minimal vaginal abnormalities on examination OR Epithelial disruption < 25% of total surface	Moderate vaginal abnormalities on examination OR Epithelial disruption of 25 - 49% total surface	Severe vaginal abnormalities on examination OR Epithelial disruption 50 - 75% total surface	Vaginal perforation OR Epithelial disruption > 75% total surface
**OCULAR/VISUAL**				
Uveitis	Asymptomatic but detectable on exam	Symptomatic anterior uveitis OR Medical intervention indicated	Posterior or pan-uveitis OR Operative intervention indicated	Disabling visual loss in affected eye(s)
Visual changes (from baseline)	Visual changes causing no or minimal interference with usual social & functional activities	Visual changes causing greater than minimal interference with usual social & functional activities	Visual changes causing inability to perform usual social & functional activities	Disabling visual loss in affected eye(s)
**ENDOCRINE/METABOLIC**				
Abnormal fat accumulation (e.g., back of neck, breasts, abdomen)	Detectable by study participant (or by caregiver for young children and disabled adults)	Detectable on physical exam by health care provider	Disfiguring OR Obvious changes on casual visual inspection	NA
Diabetes mellitus	NA	New onset without need to initiate medication OR Modification of current medications to regain glucose control	New onset with initiation of medication indicated OR Diabetes uncontrolled despite treatment modification	Life-threatening consequences (e.g., ketoacidosis, hyperosmolar non-ketotic coma)
Gynecomastia	Detectable by study participant or caregiver (for young children and disabled adults)	Detectable on physical exam by health care provider	Disfiguring OR Obvious on casual visual inspection	NA
Hyperthyroidism	Asymptomatic	Symptomatic causing greater than minimal interference with usual social & functional activities OR Thyroid suppression therapy indicated	Symptoms causing inability to perform usual social & functional activities OR Uncontrolled despite treatment modification	Life-threatening consequences (e.g., thyroid storm)
Hypothyroidism	Asymptomatic	Symptomatic causing greater than minimal interference with usual social & functional activities OR Thyroid replacement therapy indicated	Symptoms causing inability to perform usual social & functional activities OR Uncontrolled despite treatment modification	Life-threatening consequences (e.g., myxedema coma)

CLINICAL				
**PARAMETER**	**GRADE 1<br>MILD**	**GRADE 2<br>MODERATE**	**GRADE 3<br>SEVERE**	**GRADE 4<br>POTENTIALLY LIFE-THREATENING**
Lipoatrophy<br>(e.g., fat loss from the face, extremities, buttocks)	Detectable by study participant (or by caregiver for young children and disabled adults)	Detectable on physical exam by health care provider	Disfiguring OR Obvious on casual visual inspection	NA

LABORATORY				
**PARAMETER**	**GRADE 1<br>MILD**	**GRADE 2<br>MODERATE**	**GRADE 3<br>SEVERE**	**GRADE 4<br>POTENTIALLY LIFE-THREATENING**
**HAEMATOLOGY**	***Standard International Units are listed in italics***			
Absolute neutrophil count (ANC) [9, 44]				
**Adult and Paediatric, > 7 days**	750 –< 1,000/mm^3<br>*0.75 x 10^9 – <1.0. x 10^9/L*	500 – 749/mm^3<br>*0.5 x 10^9 – 0.749 x 10^9/L*	250 – 499/mm^3<br>*0.25 x 10^9 – 0.499 x 10^9/L*	< 250/mm^3<br>*< 0.250 x 10^9/L*
Fibrinogen, decreased	100 – 200 mg/dL<br>*1.00 – 2.00 g/L*<br>OR<br>0.75 – 0.99 x LLN	75 – 99 mg/dL<br>*0.75 – 0.99 g/L*<br>OR<br>0.50 – 0.74 x LLN	50 – 74 mg/dL<br>*0.50 – 0.74 g/L*<br>OR<br>0.25 – 0.49 x LLN	< 50 mg/dL<br>*< 0.50 g/L*<br>OR<br>< 0.25 x LLN<br>OR<br>Associated with gross bleeding
Haemoglobin (Hgb)				
**Adult and Paediatric ≥ 57 days** (HIV POSITIVE ONLY)	8.5 – 10.0 g/dL<br>*5.24-6.23 mmol/L*	7.5 – 8.4 g/dL<br>*4.62-5.23 mmol/L*	6.50 – 7.4 g/dL<br>*4.03-4.61 mmol/L*	< 6.5 g/dL<br>*< 4.03 mmol/L*
International Normalized Ratio of prothrombin time (INR)	1.1 – 1.5 x ULN	1.6 – 2.0 x ULN	2.1 – 3.0 x ULN	> 3.0 x ULN
Methemoglobin	5.0 – 10.0%	10.1 – 15.0%	15.1 – 20.0%	> 20.0%
Prothrombin Time (PT)	1.1 – 1.25 x ULN	1.26 – 1.50 x ULN	1.51 – 3.00 x ULN	> 3.00 x ULN
Partial Thromboplastin Time (PTT)	1.1 – 1.66 x ULN	1.67 – 2.33 x ULN	2.34 – 3.00 x ULN	> 3.00 x ULN
Platelets, decreased	100,000 – 124,999/mm^3<br>*100.000 x 10^9 – 124.999 x 10^9/L*	50,000 – 99,999/mm^3<br>*50.000 x 10^9 – 99.999 x 10^9/L*	25,000 – 49,999/mm^3<br>*25.000 x 10^9 – 49.999 x 10^9/L*	< 25,000/mm^3<br>*< 25.000 x 10^9/L*
WBC, decreased	2,000 – 2,500/mm^3<br>*2.000 x 10^9 – 2.500 x 10^9/L*	1,500 – 1,999/mm^3<br>*1.500 x 10^9 – 1.999 x 10^9/L*	1,000 – 1,499/mm^3<br>*1.000 x 10^9 – 1.499 x 10^9/L*	< 1,000/mm^3<br>*< 1.000 x 10^9/L*
**CHEMISTRIES**	***Standard International Units are listed in italics***			
Acidosis	NA	pH < normal, but ≥ 7.3	pH < 7.3 without life-threatening consequences	pH < 7.3 with life-threatening consequences

CLINICAL				
**PARAMETER**	**GRADE 1 MILD**	**GRADE 2 MODERATE**	**GRADE 3 SEVERE**	**GRADE 4 POTENTIALLY LIFE-THREATENING**
Albumin, serum, low	3.0 g/dL – < LLN<br>*30 g/L – < LLN*	2.0 – 2.9 g/dL<br>*20 – 29 g/L*	< 2.0 g/dL<br>*< 20 g/L*	NA
Alkaline Phosphatase	1.25 – 2.5 x ULN†	2.6 – 5.0 x ULN†	5.1 – 10.0 x ULN†	> 10.0 x ULN†
Alkalosis	NA	pH > normal, but ≤ 7.5	pH > 7.5 without life-threatening consequences	pH > 7.5 with life-threatening consequences
ALT (SGPT)	1.25 – 2.5 x ULN	2.6 – 5.0 x ULN	5.1 – 10.0 x ULN	> 10.0 x ULN
AST (SGOT)	1.25 – 2.5 x ULN	2.6 – 5.0 x ULN	5.1 – 10.0 x ULN	> 10.0 x ULN
Bicarbonate, serum, low	16.0 mEq/L – < LLN<br>*16.0 mmol/L – < LLN*	11.0 – 15.9 mEq/L<br>*11.0 – 15.9 mmol/L*	8.0 – 10.9 mEq/L<br>*8.0 – 10.9 mmol/L*	< 8.0 mEq/L<br>*< 8.0 mmol/L*
Bilirubin (Total)				
**Adult and Paediatric > 14 days**	1.1 – 1.5 x ULN	1.6 – 2.5 x ULN	2.6 – 5.0 x ULN	> 5.0 x ULN
Calcium, serum, high (corrected for albumin)				
**Adult and Paediatric ≥ 7 days**	10.6 – 11.5 mg/dL<br>*2.65 – 2.88 mmol/L*	11.6 – 12.5 mg/dL<br>*2.89 – 3.13 mmol/L*	12.6 – 13.5 mg/dL<br>*3.14 – 3.38 mmol/L*	> 13.5 mg/dL<br>*> 3.38 mmol/L*
Calcium, serum, low (corrected for albumin)				
**Adult and Paediatric ≥ 7 days**	7.8 – 8.4 mg/dL<br>*1.95 – 2.10 mmol/L*	7.0 – 7.7 mg/dL<br>*1.75 – 1.94 mmol/L*	6.1 – 6.9 mg/dL<br>*1.53 – 1.74 mmol/L*	< 6.1 mg/dL<br>*< 1.53 mmol/L*
Cardiac troponin I (cTnI)	NA	NA	NA	Levels consistent with myocardial infarction or unstable angina as defined by the manufacturer
Cardiac troponin T (cTnT)	NA	NA	NA	≥ 0.20 ng/mL<br>OR<br>Levels consistent with myocardial infarction or unstable angina as defined by the manufacturer
Cholesterol (fasting)				
**Paediatric < 18 years**	170 – 199 mg/dL<br>*4.40 – 5.15 mmol/L*	200 – 300 mg/dL<br>*5.16 – 7.77 mmol/L*	> 300 mg/dL<br>*> 7.77 mmol/L*	NA
Creatine Kinase	3.0 – 5.9 x ULN†	6.0 – 9.9 x ULN†	10.0 – 19.9 x ULN†	≥ 20.0 x ULN†
Creatinine	1.1 – 1.3 x ULN†	1.4 – 1.8 x ULN†	1.9 – 3.4 x ULN†	≥ 3.5 x ULN†
Glucose, serum, high				
Nonfasting	116 – 160 mg/dL<br>*6.44 – 8.88 mmol/L*	161 – 250 mg/dL<br>*8.89 – 13.88 mmol/L*	251 – 500 mg/dL<br>*13.89 – 27.75 mmol/L*	> 500 mg/dL<br>*> 27.75 mmol/L*
Fasting	110 – 125 mg/dL<br>*6.11 – 6.94 mmol/L*	126 – 250 mg/dL<br>*6.95 – 13.88 mmol/L*	251 – 500 mg/dL<br>*13.89 – 27.75 mmol/L*	> 500 mg/dL<br>*> 27.75 mmol/L*
Glucose, serum, low				

CLINICAL				
**PARAMETER**	**GRADE 1 MILD**	**GRADE 2 MODERATE**	**GRADE 3 SEVERE**	**GRADE 4 POTENTIALLY LIFE-THREATENING**
**Adult and Paediatric ≥ 1 month**	55 – 64 mg/dL<br>*3.05 – 3.55 mmol/L*	40 – 54 mg/dL<br>*2.22 – 3.06 mmol/L*	30 – 39 mg/dL<br>*1.67 – 2.23 mmol/L*	< 30 mg/dL<br>*< 1.67 mmol/L*
Lactate	< 2.0 x ULN without acidosis	≥ 2.0 x ULN without acidosis	Increased lactate with pH < 7.3 without life-threatening consequences	Increased lactate with pH < 7.3 with life-threatening consequences
LDL cholesterol (fasting)				
**Adult ≥ 18 years**	130 – 159 mg/dL<br>*3.37 – 4.12 mmol/L*	160 – 190 mg/dL<br>*4.13 – 4.90 mmol/L*	≥ 190 mg/dL<br>*≥ 4.91 mmol/L*	NA
**Paediatric > 2 - < 18 years**	110 – 129 mg/dL<br>*2.85 – 3.34 mmol/L*	130 – 189 mg/dL<br>*3.35 – 4.90 mmol/L*	≥ 190 mg/dL<br>*≥ 4.91 mmol/L*	NA
Lipase	1.1 – 1.5 x ULN	1.6 – 3.0 x ULN	3.1 – 5.0 x ULN	> 5.0 x ULN
Magnesium, serum, low	1.2 – 1.4 mEq/L<br>*0.60 – 0.70 mmol/L*	0.9 – 1.1 mEq/L<br>*0.45 – 0.59 mmol/L*	0.6 – 0.8 mEq/L<br>*0.30 – 0.44 mmol/L*	< 0.60 mEq/L<br>*< 0.30 mmol/L*
Pancreatic amylase	1.1 – 1.5 x ULN	1.6 – 2.0 x ULN	2.1 – 5.0 x ULN	> 5.0 x ULN
Phosphate, serum, low				
**Adult and Paediatric > 14 years**	2.5 mg/dL – < LLN<br>*0.81 mmol/L – < LLN*	2.0 – 2.4 mg/dL<br>*0.65 – 0.80 mmol/L*	1.0 – 1.9 mg/dL<br>*0.32 – 0.64 mmol/L*	< 1.00 mg/dL<br>*< 0.32 mmol/L*
**Paediatric 1 year – 14 years**	3.0 – 3.5 mg/dL<br>*0.97 – 1.13 mmol/L*	2.5 – 2.9 mg/dL<br>*0.81 – 0.96 mmol/L*	1.5 – 2.4 mg/dL<br>*0.48 – 0.80 mmol/L*	< 1.50 mg/dL<br>*< 0.48 mmol/L*
**Paediatric < 1 year**	3.5 – 4.5 mg/dL<br>*1.13 – 1.45 mmol/L*	2.5 – 3.4 mg/dL<br>*0.81 – 1.12 mmol/L*	1.5 – 2.4 mg/dL<br>*0.48 – 0.80 mmol/L*	< 1.50 mg/dL<br>*< 0.48 mmol/L*
Potassium, serum, high	5.6 – 6.0 mEq/L<br>*5.6 – 6.0 mmol/L*	6.1 – 6.5 mEq/L<br>*6.1 – 6.5 mmol/L*	6.6 – 7.0 mEq/L<br>*6.6 – 7.0 mmol/L*	> 7.0 mEq/L<br>*> 7.0 mmol/L*
Potassium, serum, low	3.0 – 3.4 mEq/L<br>*3.0 – 3.4 mmol/L*	2.5 – 2.9 mEq/L<br>*2.5 – 2.9 mmol/L*	2.0 – 2.4 mEq/L<br>*2.0 – 2.4 mmol/L*	< 2.0 mEq/L<br>*< 2.0 mmol/L*
Sodium, serum, high	146 – 150 mEq/L<br>*146 – 150 mmol/L*	151 – 154 mEq/L<br>*151 – 154 mmol/L*	155 – 159 mEq/L<br>*155 – 159 mmol/L*	≥ 160 mEq/L<br>*≥ 160 mmol/L*
Sodium, serum, low	130 – 135 mEq/L<br>*130 – 135 mmol/L*	125 – 129 mEq/L<br>*125 – 129 mmol/L*	121 – 124 mEq/L<br>*121 – 124 mmol/L*	≤ 120 mEq/L<br>*≤ 120 mmol/L*
Triglycerides (fasting)	NA	500 – 750 mg/dL<br>*5.65 – 8.48 mmol/L*	751 – 1,200 mg/dL<br>*8.49 – 13.56 mmol/L*	> 1,200 mg/dL<br>*> 13.56 mmol/L*
Uric acid	7.5 – 10.0 mg/dL<br>*0.45 – 0.59 mmol/L*	10.1 – 12.0 mg/dL<br>*0.60 – 0.71 mmol/L*	12.1 – 15.0 mg/dL<br>*0.72 – 0.89 mmol/L*	> 15.0 mg/dL<br>*> 0.89 mmol/L*
**URINALYSIS**	***Standard International Units are listed in italics***			
Hematuria (microscopic)	6 – 10 RBC/HPF	> 10 RBC/HPF	Gross, with or without clots OR with RBC casts	Transfusion indicated
Proteinuria, random collection	1 +	2 – 3 +	4 +	NA
Proteinuria, 24 hour collection				
**Adult and Paediatric ≥ 10 years**	200 – 999 mg/24 h<br>*0.200 – 0.999 g/d*	1,000 – 1,999 mg/24 h<br>*1.000 – 1.999 g/d*	2,000 – 3,500 mg/24 h<br>*2.000 – 3.500 g/d*	> 3,500 mg/24 h<br>*> 3.500 g/d*

CLINICAL				
**PARAMETER**	**GRADE 1 MILD**	**GRADE 2 MODERATE**	**GRADE 3 SEVERE**	**GRADE 4 POTENTIALLY LIFE-THREATENING**
**Paediatric > 3 mo - < 10 years**	201 – 499 mg/m^2/24 h<br>*0.201 – 0.499 g/d*	500 – 799 mg/m^2/24 h<br>*0.500 – 0.799 g/d*	800 – 1,000 mg/m^2/24 h<br>*0.800 – 1.000 g/d*	> 1,000 mg/ m^2/24 h<br>*> 1.000 g/d*

Comments: *Both amylase and lipase must be elevated to the same grade or higher (i.e. if total amylase is Grade 4, but lipase is only Grade 1, the Toxicity Grade is 1. In paediatric HIV patients, the most common source of serum amylase is the salivary glands. Salivary amylase elevations are generally not clinically significant. When amylase is released from damaged pancreatic cells, it can be a marker of pancreatitis. In most cases of clinical pancreatitis, lipase will also be elevated. However, lipase is also a non-specific marker. Combined elevation of amylase and lipase (each >5 x normal) often indicates pancreatic disease and requires evaluation. However, in the absence of pancreatic disease, drug can be resumed even at Grade 3 and 4 toxicity.

If fasting values for C-peptide and serum insulin are above your centre's normal ranges, please consult with an endocrinologist locally and report on the follow-up form.

Basic Self-care functions – Adult: Activities such as bathing, dressing, toileting, transfer/movement, continence and feeding

Basic Self-care functions –Young children: Activities that are age and culturally appropriate (e.g. feeding self)

Usual Social and Functional Activities – **Adult**: Adaptive tasks and desirable activities, such as going to work, shopping, cooking, use of transportation, pursuing a hobby, etc.

Usual Social and Functional Activities – **Young children**: Activities that are age and culturally appropriate (e.g. social interactions, play activities, learning tasks, etc).

APPENDIX 10: TANNER SCALES

The Five Stages of Female Breast and Pubic Hair Development

Breast and pubic hair development should be staged separately

Stage		Female Breast	Pubic Hair
1	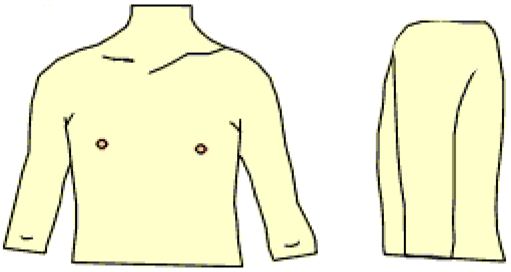	Breasts during childhood. The breasts are flat and show no signs of development.	None
2	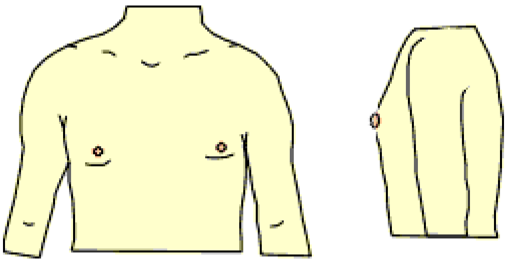	Breast bud stage. Milk ducts and fat tissue forms a small mound.	Sparse, lightly pigmented, straight, medial border of labia.
3	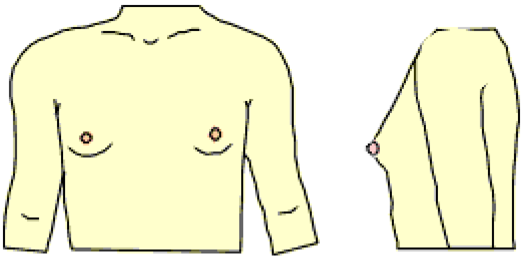	Breasts continue to grow. Breasts become rounder and fuller.	Darker, beginning to curl, increased amount.
4	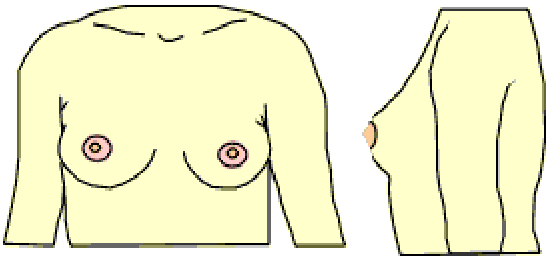	Nipple and areola form separate small mound. Not all girls go through this stage. Some skip stage 4 and go directly to stage 5.	Coarse, curly, abundant but amount less than in adult.
5	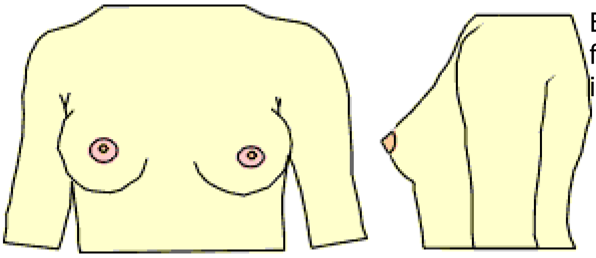	Breast growth enters final stage. Adult breast is full and round shaped.	Adult feminine triangle, spread to medial surface of thighs

Copyright© 1998,1999 VirtualKid All Rights Reserved.

The Five Stages of Male Genitalia and Pubic Hair Development

Genitalia and Pubic hair should be staged separately

Stage		Male Genitalia	Pubic Hair
1	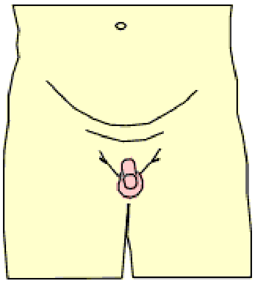	Penis and testicles of a child. Testicles between 1 and 3 milliliters in volume.	No pubic hair.
2	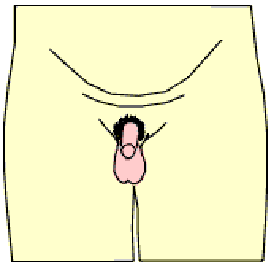	First signs for penis and testicle growth, Testicles become larger. Testicles between 4 and 6 millilitres in volume.	Pubic hair beginning to grow: appears sparse and downy straight.
3	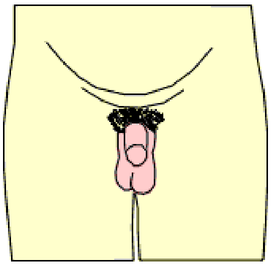	Penis continues to grow getting wider and longer. Testicles continue to grow larger. Testicles between 7 and 16 millilitres in volume.	Pubic hair appears curlier and coarser with increased pigmentation.
4	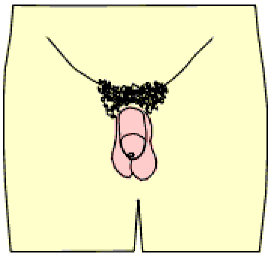	Penis continues to grow getting wider and longer. Testicles continue to grow larger. Penis gland or head is more developed. Testicles between 12 and 24 milliliters in volume. Testicles are about 1 1/2 inches long.	Pubic hair becomes adult type, but less.
5	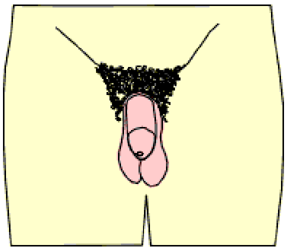	Penis growth enters final stage. Average erect penis length 6 1/4 inches. 90% are 5 - 7 inches. Glans penis or head is fully developed. Testicles 16 - 27 millilitres in volume. Testicles are about 1-3/4 inches.	Pubic hair is thick spreading to medial thighs.

APPENDIX 11: VIROLOGY/IMMUNOLOGY SUBSTUDY

Virology
Proviral DNA will be quantified in all young people at randomisation (week 0) to ascertain whether it can predict response in both arms. To determine whether proviral DNA load differs between arms it will be assayed again in all young people at 48 weeks, and in young people in the SCT arm at 4 weeks. Low level viral loads (<10 c/ml) will also be carried out at the same time points, as well as in young people in the pilot phase of the SCT arm will also be assayed at phlebotomy visits at week 1, 2 and 3 (Monday after interrupting therapy on Saturday/Sunday).

Resistance testing will be performed on all young people who lose virological suppression in either arm at the point of loss of suppression (≥50 c/ml), and any subsequent samples with HIV-1 RNA ≥1000 c/ml. Resistance tests will be performed locally; where this is not possible or the amino acid sequence in FASTA file format cannot be provided, they will be performed centrally.

Therefore, samples will be used to determine if differences in proviral DNA during the study correlate with virological outcome and whether SCT will reduce the subsequent drug options through increased number of drug resistant mutations emerging during the study.

Immunology
The effects of the short interruption, if any, are likely to be subtle. Ascertainment of naïve/ memory phenotypes, recent thymic emigrants (RTE), and evidence of activation/proliferation will be determined on frozen cells in batches at the end of the trial. The arms will be compared to see if SCT alters these parameters as compared to continual therapy. Proportions of naïve:memory, activated:quiescent and RTE:CD4 cells will be analysed to ascertain whether they correlate with total and cell subset proviral DNA levels and virological rebound. Cells will also be analysed for telomere shortening. Plasma biomarkers including D-dimers, IL6 and CRP will be determined from samples collected.

APPENDIX 12: QUALITATIVE SUBSTUDY: YOUNG PEOPLE AND HIV TREATMENT ADHERENCE: A MULTI-METHOD QUALITATIVE STUDY

Investigators

The qualitative study will be managed as an integral component of the trial by Co-Investigators Professor Tim Rhodes and Dr Sarah Bernays, London School of Hygiene and Tropical Medicine (LSHTM).

Rationale

Whereas HIV treatment research is predominantly focused on estimating the extent of adherence and its predictors, qualitative studies explore how participants experience their treatment in the context of their everyday lives. In conjunction with the BREATHER trial, this qualitative research thus aims to understand the way in which SCT is experienced and valued, and the combination of factors which may hinder or facilitate its implementation if scaled-up. How young people understand and orientate to the process of adaptation from continuous to SCT is a key concern, given that this will constitute a break from previously experienced treatment routine and rationale. The qualitative evidence produced by this study will guide the future evaluation and implementation of SCT.

Key areas of investigation

The table below summarises the key (overlapping) themes of investigation which will frame the overall direction of the study.

Theme	*Description*
Identity	We will generate data on how young people present, and reflect upon, their identities, including regarding: self; HIV; health; adaptation and disruption following diagnosis; and disclosure.
Treatment experience	We will generate in-depth accounts of HIV treatment experience, and the meaning that treatment has in the context of everyday life, including regarding: medical effects; medication; mode of delivery; health care setting; change over time; and future expectations. We will also explore treatment experience with a specific focus on week days and weekends. We will also generate data specific to short-cycle treatment (by the end of the study).
Adherence	We will explore contextual factors in adherence, including related to: treatment strategy; treatment delivery; self and mediated treatment; medication; treatment setting; personal and situational factors; social conditions; change over time; and accounts of adherence slip-up, interruption, failure and expectation. At follow up this will extend to their experience in their assigned arm of the study.
Transition	We will use 'transition' as a key guiding concept for informing the study, both regarding youth transitions in identity, childhood and adolescence, and treatment transitions in relation to mode of delivery and setting (especially transitions from paediatric to adult care, and from mediated to self-adherence). We will also investigate the transition from continuous to SCT as a process of adaptation.
Expectation	We will use 'expectation' as a guiding concept for understanding young people's accounts of their HIV treatment engagement and adherence, including in the context of transitions to adulthood.
Trial experience	At the end of the study we will explore accounts of the trial, process factors influencing participation, and its acceptability, including issues of

	consent, and understandings of the risks and benefits of participation.

Methods

Design

Our approach to data collection is to capture young people's experiences of HIV treatment *as they describe and depict them*. In this way the qualitative study, although embedded within the trial, will be differentiated by using a 'non-directive' approach. The study aims to facilitate story-telling and conversation which is led as much by the participants as the researchers. This is a multi-method qualitative design, combining semi-structured face-to-face interviews with a life history component, audio diaries, and focus groups (subject to feasibility). A multi-method design maximises validity through triangulation across methods and data sources. It also enables the study to capture accounts of current as well as past treatment experience.

Data collection with trial participants

Sample

All young people recruited into the BREATHER trial in the UK, Ireland, the USA and Uganda are eligible to participate, subject to consent (see below) and self-awareness of HIV infection (for at least 6 months). To be eligible they will need to be 10—24 years old. We envisage a sample of at least 40 for the qualitative study (20 from UK, Ireland and the USA and 20 from Uganda), of whom at least 20 will be in the SCT arm. Within this, purposive sampling will give primary emphasis to 'responsibility for medication' (sole; shared; carer), with secondary dimensions including: age; gender; ethnicity; and family background. The sample will be drawn across all of the participating UK, Irish and American sites. A sample size of at least 40 is judged sufficient to reach adequate 'data saturation' during analyses, assuming the generation of quality audio-recorded interviews (see below).

Interviews

Where feasible we will undertake three interviews. The first interview will take place in the early stages of the trial. The second interview will take place towards the end of the main trial or if a young person on the SCT arm restarts continuous therapy (for example, due to a virological rebound). The third interview will be carried out three to six months after the end of the main trial.

Interview methods will be adapted according to age and situation. Such methods, which are tailored to participants and may not be used with all participants alike, seek to create an atmosphere which is at once engaging and non-intimidating. All interviews will be audio-recorded, subject to consent. Interview topic guides and related materials will be developed in close collaboration with the trial team to ensure that the qualitative study does not influence the likelihood of a participant remaining adherent to their randomised arm. All interviews will be undertaken by the qualitative research team (who have extensive experience in qualitative interviewing).

Audio diaries

We will explore with all participants in the study their interest in contributing a personal audio diary (using provided Dictaphones) generated over two week periods. For those interested, these will take place twice: in the early stages of the trial; and in the latter half of the trial. The direction that personal diaries take is flexible and participant-led.

Focus groups

In Uganda, where many of the young people are active in the same support group, and as they are all attending the same clinic are more likely to know each other, if feasible we will conduct one focus group towards the end of the study. This will be with about 8 participants to access participants' feedback on emerging findings and explore participatory plans for disseminating study findings.

Data collection with non-trial participants

Focus groups with non-trial participants
To be able to engage with exploring the acceptability of the intervention more broadly, we will conduct 3-5 focus groups with young people living with HIV in the UK, Ireland and Uganda who have not participated in the trial. These focus groups will explore what these young people think about the idea of short cycle therapy, its perceived relevance and capacity to engage with adherence challenges and how the intervention, should it be rolled out, could best be explained to young people and their carers. These focus groups will be conducted after the end of the main trial.

Carers' interviews: In Uganda only at this stage, 15 carers will be invited to be interviewed after the main trial is completed. The interview will take place at the clinic, to protect their anonymity with their communities, but at a time that is convenient to them. The interview will explore their decision making process in consenting for their child to participate in the trial, their reflections on how their expectations aligned with their experience of the trial and their perceptions of the barriers and facilitators to adolescents' adherence.

Ethical approval of focus groups with non-trial participants and carers' interviews and the associated information sheets and consent forms will be sought from the London School of Hygiene and Tropical Medicine.

Ethics
Consent procedure for all qualitative study participants
As with the main trial, written informed consent will be obtained from participants and parents/carers. Consent to participate in the qualitative study for all young people will only be sought from participants who are aware of their HIV infection, and have been so for at least six months. All children who are deemed competent by the clinician will be given an age-specific information sheet and asked to sign an assent form. For 16-17 year olds, consent should be obtained according to national regulations. For participants aged 18 years or older, consent from the parent/carer is not required.

Carers in Uganda participating in the interviews will be given a specific information sheet and consent form. The inclusion criteria for carers mean that they do not need to be HIV positive themselves.

Ethical approval for the data collection with non-trial participants will be sought from the London School of Hygiene and Tropical Medicine ethics committee.

Confidentiality, data protection and archiving
All data collected from both trial and non-trial participants will be completely confidential, and anonymity guaranteed. We will ensure that quoted extracts are not attributable to an individual. The only condition under which confidentiality can be breached is when an issue of child protection is disclosed. This will be made clear during the consent procedure. If child protection issues, such as abuse or suicide, are disclosed during the data collection, then the participant will be informed that it is not possible to keep this information confidential and it will be passed on to the appropriate member of clinic staff, ideally with the participant's consent.

Incentives (UK and Ireland only)
We will provide gift vouchers to participating young people to a value of £15 for first interviews and £20 for second and third interviews, as well as covering related travel expenses). Incentives will not be provided for participating in the focus groups, although food and refreshments will be provided and travel costs will be reimbursed.
Travel reimbursements in Uganda

As is commensurate with the BREATHER trial, all participants taking part in Uganda will have their travel costs reimbursed.

Information sheets and consent forms for the BREATHER QUALITATIVE STUDY
(Interviews and audio/written diaries)

INFORMATION SHEETS

1. Parents/ carers
2. Young adults (18-24 years old)
3. Young adults (16-17 years old)
4. Young people (approx 12-15 years old)
5. Children (approx 8-11 years old)
6. Carer interviews (Uganda)

CONSENT FORMS

7. Parents/ carers
8. Young adults (18-24 years old)
9. Young adults (16-17 years old)
10. Carer interviews (Uganda)

ASSENT FORMS

11. Anyone participating under 16 years old.

BREATHER Qualitative study- Young people and HIV treatment adherence
AN INFORMATION SHEET FOR PARENTS/ CARERS

As part of the BREATHER trial, we are carrying out a qualitative interview study to hear from children and young people (aged 8-24 years) about their experiences of HIV treatment. At least 40 children and young people from the PENTA trial will take part in this study.
Before you decide whether you would like your child to participate it is important that you understand why the research is being done and what it involves. Please read this information sheet carefully and ask any questions you may have. You can also ask one for of the study researchers to telephone you should you like to discuss the study in more detail before deciding.
Thank you for taking the time to read this information.

What is the purpose of the study?
The study aims to gain a better understanding of what the day-to-day experience of HIV treatment is like for children and young people. There is little research asking children and young people themselves of their experiences, and this is why we are doing the study. We are especially interested in how they manage taking their treatment day-to-day and any difficulties they have with this. We are also interested in how they experience being involved in the study. By listening to what children and young people tell us, we hope to suggest ways to improve the experience of treatment including supporting long term adherence to taking HIV treatment.

Who is carrying out the study?
This research is part of the BREATHER study. It is therefore organised by the Paediatric European Network for the Treatment of AIDS (PENTA). This qualitative study is being run by [researcher names] at the London School of Hygiene and Tropical Medicine.

What is involved?

This is a 'qualitative' study. The approach assumes that the best way to learn about the experiences of children and young people is to listen carefully to what they have to say. We would therefore like to talk to your child about what their experience of HIV treatment is like, and whether they experience difficulties in maintaining adherence to their prescribed medications. We would also like to learn about their experience of this study, either through 'short-cycle therapy' or continuous therapy.
Children and young people in the study will participate in three interviews and if they would like to, in keeping an audio diary.

Interviews

The first interview will take place in the early stages of the trial. The second interview will be towards the end of their participation in the trial. There may be a third interview, which will take place 3-6 months after the study has ended. All interviews will take place at a place and time convenient to you and your child. It will be conducted by one of the qualitative researchers and will last about an hour. We ask that we record the interview unless your child does not want this. Your child does not have to talk about things that he or she does not want to talk about, and they can end the interview at any time and without explaining why.

Audio diaries

In addition to the interviews, we would like to invite your child to keep an audio diary, which is like a spoken diary. We will provide your child with a Dictaphone and ask him/ her to record an audio diary over two fortnightly periods. This will be in the early stages of the study and the second period will be around 36 weeks.

Written Diaries

If your child happens to be keeping a written diary relating to their HIV and treatment and would like to share this with the research team, this could also be used as part of the research to better understand their experience.
Both audio and written diaries are options. If preferred, your child can participate in the study interviews only.

Is the research confidential?

Yes. Any information that your child shares with us will only be seen by members of the research team. This means that whenever we write or talk about anything we have been told we never use your child's real name. All information about your child will be stored securely.
The only information that we may have to pass on is information about children and young people who are at risk of serious harm, such as physical harm or neglect.

How will the research be used?

The findings of this study will be reported to a wide range of people, including those who provide treatment, care and support for children and young people living with HIV. The findings will be used to guide the future implementation of HIV treatment, short-cycle therapy, and HIV treatment studies involving children and young people.

What are the benefits and risks of taking part?

Your child will be part of a study that aims to help young people maintain long term adherence. Your child may see the opportunity to talk about their HIV treatment as an entirely positive experience. For some, however, this is a sensitive topic which may be difficult to talk about. Your child will not need to answer any questions that they do not want to. They are free to withdraw from the interview and/or study at any time. Great care is given to asking questions as sensitively as possible. The content of the interviews is also led by the child (as well as the researcher) and what they wish to talk about. Support is also available to participants through their clinic, including immediately after the interview.

Does my child have to take part?

No, your child does not have to take part in the study. Your child's participation in the BREATHER study will not be affected in anyway if you prefer for your child not to take part. It will not affect any of the services your child receives.

Will my child receive anything from taking part?

Yes, all children and young people who take part will receive a £15 gift-voucher for the first interview and £20 for the second and third interview.
Travel expenses to attend interviews will be reimbursed. *[Country specific, delete if not applicable]*

Can I have some more information before I decide?

If you would like some more information, the staff at your clinic can arrange for you to speak to the research team about the study and answer any further questions you may have.

Ok, I'm happy for my child to take part. Now what?

Please sign the consent form and give it to the staff at your clinic. We will not be inviting everyone to take part who is participating in the study. Instead we will invite a representative group of

young people to take part in this qualitative study. If your child is selected [researcher names] will contact you on the phone number that you have given the clinic to arrange the first interview.

Thank you for reading this!
If you do decide to participate your help will be very valuable to us.

BREATHER QUALITATIVE STUDY- YOUNG PEOPLE AND HIV TREATMENT ADHERENCE AN INFORMATION SHEET FOR YOUNG ADULTS (18+)

As part of the BREATHER study, we are doing a qualitative interview study to hear from children and young people (aged 8-24 years) about their experiences of HIV treatment. At least 40 children and young people from the PENTA study will take part in this study.
Before you decide whether you want to participate it is important that you understand why the research is being done and what it involves. Please read this information sheet carefully and ask any questions you may have. You can also ask for one of the study researchers to telephone you should you like to discuss the study in more detail before deciding.
Thank you for taking the time to read this information.

What is the purpose of the study?
The study aims to gain a better understanding of what the day-to-day experience of HIV treatment is like for children and young people. There is little research asking young people themselves of their experiences, and this is why we are doing the study. We are especially interested in how they manage taking their treatment day-to-day and any difficulties they have with this. We are also interested in how you find being involved in the study. By listening to what you and other young people tell us, we hope to suggest ways to improve the experience of treatment including long term adherence to taking medications.

Who is carrying out the study?
This research is part of the BREATHER study. It is therefore organised by the Paediatric European Network for the Treatment of AIDS (PENTA). This qualitative study is being run by [researcher names] at the London School of Hygiene and Tropical Medicine.

What is involved?
This is a 'qualitative' study. The approach assumes that the best way to learn about the experiences of young people is to listen carefully to what they have to say. We would therefore like to talk to you about what your experience of HIV treatment is like, and whether you experience any difficulties in maintaining adherence to their prescribed medications. We would also like to learn about your experience of this study, either through 'short-cycle therapy' or continuous therapy.
If you choose to take part the study will involve participating in three interviews and if you would like to, in keeping an audio diary.

Interviews
The first interview will take place in the early stages of the trial. The second interview will be towards the end of your participation in the trial. There may be a third interview, which will take place 3-6 months after the study has ended. All interviews will take place at a place and time convenient to you. It will be conducted by one of the researchers and will last about an hour. We ask that we record the interview unless you do not want this. You do not have to talk about things that you do not want to talk about. You can end the interview at any time, without explaining why.

Diaries
Audio diaries
In addition to the interviews, we would like to invite you to keep an audio diary, which is like a spoken diary. We will provide you with a Dictaphone and ask you to record an audio diary over two fortnightly periods. This will be in the early stages of the trial and the second period will be around 36 weeks.

Written Diaries

If you happen to be keeping a written diary relating to your HIV and treatment and would like to share this with the research team, this could be used as part of the research to better understand your experience.
Both audio and written diaries are options. If you prefer, you can participate in the study interviews only.

Is the research confidential?
Yes. Any information that you share with us will only be seen by members of the research team. This means that whenever we write or talk about anything we have been told we never use your real name. All information about you will be stored securely.

How will the research be used?
The findings of this study will be reported to a wide range of people, including those who provide treatment, care and support for children and young people living with HIV. The findings will be used to guide the future implementation of HIV treatment, short-cycle therapy and HIV treatment studies involving children and young people.

What are the benefits and risks of taking part?
You will be part of a study that aims to help young people maintain long term adherence. You may see the opportunity to talk about your HIV treatment as an entirely positive experience. However as this is a sensitive issue it may be difficult to talk about. Great care is given to asking questions as sensitively as possible. The content of the interviews is also led by you (as well as the researcher) and what you wish to talk about. Support is also available to you through your clinic, including immediately after the interview.

Do I have to take part?
No, you do not have to take part in the study. Your participation in the BREATHER study will not be affected in anyway if you prefer not to take part. It will not affect any of the services you currently receive.

Will I receive anything for taking part?
Yes, you will receive a £15 gift-voucher for the first interview and £20 for the second and third interview. Travel expenses to attend interviews will be reimbursed.[Country specific, delete if not applicable]

Can I have some more information before I decide?
If you would like some more information, the staff at your clinic can arrange for you to speak to the research team about the study and answer any further questions you may have.

Ok, I'm happy for to take part. Now what?
Please sign the consent form and give it to the staff at your clinic. We will not be inviting everyone to take part who is participating in the study. Instead we will invite a representative group of young people to take part in this qualitative study. If you are selected [researcher names] will contact you on the phone number that you have given the clinic to arrange the first interview.

Thank you for reading this!
If you do decide to participate your help will be very valuable to us.

BREATHER Qualitative study- Young people and HIV treatment adherence
AN INFORMATION SHEET FOR YOUNG ADULTS (16-17 YEARS OLD)

As part of the BREATHER study, we are doing a qualitative interview study to hear from children and young people (aged 8-24 years) about their experiences of HIV treatment. At least 40 children and young people from the PENTA study will take part in this study.
Before you decide whether you want to participate it is important that you understand why the research is being done and what it involves. Please read this information sheet carefully and ask any questions you may have. You can also ask for one of the study researchers to telephone you should you like to discuss the study in more detail before deciding.
Thank you for taking the time to read this information.

What is the purpose of the study?
The study aims to gain a better understanding of what the day-to-day experience of HIV treatment is like for children and young people. There is little research asking young people themselves of their experiences, and this is why we are doing the study. We are especially interested in how they manage taking their treatment day-to-day and any difficulties they have with this. We are also interested in how you find being involved in the study. By listening to what you and other young people tell us, we hope to suggest ways to improve the experience of treatment including long term adherence to taking medications.

Who is carrying out the study?
This research is part of the BREATHER study. It is therefore organised by the Paediatric European Network for the Treatment of AIDS (PENTA). This qualitative study is being run by [researcher names] at the London School of Hygiene and Tropical Medicine.

What is involved?
This is a 'qualitative' study. The approach assumes that the best way to learn about the experiences of young people is to listen carefully to what they have to say. We would therefore like to talk to you about what your experience of HIV treatment is like, and whether you experience any difficulties in maintaining adherence to their prescribed medications. We would also like to learn about your experience of this study, either through 'short-cycle therapy' or continuous therapy.
If you choose to take part the study will involve participating in three interviews and if you would like to, in keeping an audio diary.

Interviews
The first interview will take place in the early stages of the trial. The second interview will be towards the end of your participation in the trial. There may be a third interview, which will take place 3-6 months after the study has ended. All interviews will take place at a place and time convenient to you. It will be conducted by one of the researchers and will last about an hour. We ask that we record the interview unless you do not want this. You do not have to talk about things that you do not want to talk about. You can end the interview at any time, without explaining why.

Diaries
Audio diaries
In addition to the interviews, we would like to invite you to keep an audio diary, which is like a spoken diary. We will provide you with a Dictaphone and ask you to record an audio diary over two fortnightly periods. This will be in the early stages of the study and the second period will be around 36 weeks.
Written Diaries
If you happen to be keeping a written diary relating to your HIV and treatment and would like to share this with the research team, this could be used as part of the research to better understand your experience.

Both audio and written diaries are options. If you prefer, you can participate in the study interviews only.

Is the research confidential?
Yes. Any information that you share with us will only be seen by members of the research team. This means that whenever we write or talk about anything we have been told we never use your real name. All information about you will be stored securely.
The only information that we may have to pass on is information about young people who are at risk of serious harm, such as physical harm or neglect.

How will the research be used?
The findings of this study will be reported to a wide range of people, including those who provide treatment, care and support for children and young people living with HIV. The findings will be used to guide the future implementation of HIV treatment, short-cycle therapy and HIV treatment studies involving children and young people.

What are the benefits and risks of taking part?
You will be part of a study that aims to help young people maintain long term adherence. You may see the opportunity to talk about your HIV treatment as an entirely positive experience. However as this is a sensitive issue it may be difficult to talk about. Great care is given to asking questions as sensitively as possible. The content of the interviews is also led by you (as well as the researcher) and what you wish to talk about. Support is also available to you through your clinic, including immediately after the interview.

Do I have to take part?
No, you do not have to take part in the study. Your participation in the BREATHER study will not be affected in anyway if you prefer not to take part. It will not affect any of the services you currently receive.

Will I receive anything for taking part?
Yes, you will receive a £15 gift-voucher for the first interview and £20 for the second and third interview. Travel expenses to attend interviews will be covered. [Country specific, delete if not applicable]

Can I have some more information before I decide?
If you would like some more information, the staff at your clinic can arrange for you to speak to the research team about the study and answer any further questions you may have.
Ok, I'm happy for to take part. Now what?
Please sign the consent form and give it to the staff at your clinic. We will not be inviting everyone to take part who is participating in the study. Instead we will invite a representative group of young people to take part in this qualitative study. If you are selected [researcher names] will contact you on the phone number that you have given the clinic to arrange the first interview.

Thank you for reading this!
If you do decide to participate your help will be very valuable to us.

BREATHER Qualitative study- Young people and HIV treatment adherence
An information sheet for young people

As part of the BREATHER study, we are doing a study to hear from children and young people (aged 8-24 years) about their experiences of HIV treatment. At least 40 children and young people from the PENTA study will take part in this study. We would like you to join our study.
Before you decide if you want to take part please read this information sheet carefully and ask any questions you may have.

What is the purpose of the study?
This is a study to find out about what it is like for young people to take HIV treatment. This will be done by talking with young people, like you, about their experiences of HIV treatment. This is because we think that the best way to learn about the experiences of young people is to listen carefully to what they have to say.

Why is the study being done?
Until now there hasn't been much research done which asks young people themselves about their experiences of taking HIV treatment. This is why we are doing the study. By listening to what young people tell us we hope to make some suggestions about how to help young people maintain long term adherence to taking their HIV treatment medicines

Who is doing the study?
This is study is part of the BREATHER study and is organised by the same group. It is being conducted by [researcher names] from the London School of Hygiene and Tropical Medicine.

What would I be asked to do?

Interviews
If you do take part in the study you would meet the research team for an interview two times during the study. The first interview would be in the early stages of the study. The second interview will be towards the end of the study. There may be a third interview, which will take place 3-6 months after the study has ended. These interviews are like conversations and will each last for about an hour. There are no wrong or right answers. You do not have to answer any questions that you do not want to. You can stop the interview at any time, without having to explain why.
We would like to audio record these interviews, if this is ok with you. The interviews will take place somewhere that you feel comfortable.

Diaries

Audio diaries
In addition to the two interviews we would also like you to keep an audio diary. This is like a spoken diary. We would provide you with a Dictaphone and give guidance to what topics we would like you to talk about. We would want you to record your audio diary for two weeks near the start of the study and again for two weeks around the 36th week of the study.

Written diaries
If you are already keeping a diary in which you talk about taking HIV treatment and would like to share it with the research team, then we could use this to better understand your experiences.
However, both the audio and written diaries are options. If you want to take part in the interviews only then that is absolutely okay.

Is the research confidential?

Yes. Any information that you share with us will only be seen by members of the research team. This means that whenever we write or talk about anything you have told us we never use your real name. All information about you will be stored securely.
The only exception to this is if you tell us something that makes us worried about you or another young person's safety, such as physical harm or neglect. If we have to tell someone else, we will talk to you about it first.

How will the research be used?

The findings of this study will be reported to a wide range of people who are involved in providing treatment, care and support for children and young people living with HIV. The findings will be used to guide future interventions to support young people to take their HIV treatment effectively for a long time. It will also be used to help people who are conducting other HIV treatment studies with young people.

What are the benefits and risks of taking part?

You will be part of a study that aims to help young people maintain long term adherence. Some young people may find the opportunity to talk about their HIV treatment to be a really good thing. Some young people though might find it difficult to talk about. The research team have done research with young people and will take care to ask questions sensitively. It is up to you what you want to tell us. Also if you want, there can be someone available from your clinic to talk to, including after the interview.

Do I have to take part?

No, you do not have to take part in the study. Your participation in the BREATHER study will not be affected in anyway if you decide not to take part. It will not affect any of the services you currently receive.

Will I receive anything for taking part?

Yes, you will receive a £15 gift-voucher for the first interview and £20 for the second and third interview. Travel expenses to attend interviews will be covered. *[Country specific, delete if not applicable]*

Can I have some more information before I decide?

If you would like to find out more before deciding the staff at your clinic can arrange for you to speak to the research team more about it.

Ok, I'm happy to take part. Now what?

About half of the young people in the study can take part in this study. We will be inviting a representative group from amongst the participants in the study. If you are selected [researcher names] will contact you and your parents/ carer to arrange the first interview.

Thank you for reading this!
If you do decide to take part your help will be very helpful to us.

BREATHER Qualitative study- Young people and HIV treatment adherence
An information sheet for children

We would like you to join our study. Please read this information sheet carefully. You can ask any questions you may have.

What is the study?

This is a research study to find out about what it is like for children to take anti-HIV medicines. This will be done by talking with children, like you, about their experiences. This is because we think that the best way to learn about the experiences of children is to listen to what they have to say.

Why is this study being done?

Until now there hasn't been much research done which asks children themselves about what it is like to take anti-HIV medicines. This is why we are doing the study. By listening carefully to what children tell us we hope to make some suggestions about how to make it easier for children to take anti-HIV medicines.

Who is doing the study?

This study is part of the BREATHER study and is organised by the same group. It is being conducted by [researcher names] from the London School of Hygiene and Tropical Medicine.

Do I have to take part?

No. You can say no and this is perfectly fine. If you do not want to take part you will still be part of the PENTA study. Your doctor will still look after you as they do now. So, please don't feel you have to take part if you don't want to.

What would I be asked to do?

Interviews

If you do take part in the study you would meet the research team for an interview two times. The first interview would be near to the start of the study. The second interview will be near the end of the study. There may be a third interview 3-6 months after the study has ended. These interviews are like conversations and will each last for about an hour. There are no wrong or right answers.

We would like to audio record these interviews, if this is ok with you. The interviews will take place somewhere that you feel comfortable.

Diaries

Audio diaries: If you wanted to we would like you to keep a spoken diary for some of the time that you are doing the study. This is called an audio diary. We would provide you with a tape recorder for you to speak into about what it's like taking your anti-HIV medicines. We would like you to keep this diary for two weeks near to the beginning of the study and for two weeks towards the end (at 36 weeks).

***Written diaries**:* If you keep a diary about your anti-medicines and would like to show it to us then we can use this as well to help us understand more about your experiences.
If you want to take part in the interviews but not do the diaries, then that is absolutely okay.

Will people know what I've said?
Only the study team will listen to what you have said. If we tell people what you tell us we will never use your real name, so no one will know that it is you. You can choose the name we use if you like.
Your interviews and diaries will be kept locked away safely. The only time that we may have to share what you have told us with someone else is if we are worried about you or another child's safety.

Are there any risks?
Some children might find it hard to talk about taking their anti-HIV medicines. It is up to you what you want to tell us. You do not have to answer any questions that you do not want to. You can stop the interview or being part of the study at any time. You won't have to say why. If you want to you can talk to someone from the clinic after the interview.

Will I be given anything for taking part?

Yes, you will be given a £15 gift-voucher for the first interview and £20 for the second and third interview. Your travel to come to interviews will be paid for. *[Country specific, delete if not applicable]*

Can I have some more information before I decide?

If you would like to find out more, the staff at your clinic can arrange for you to speak to the research team about it.

Ok, I'm happy to take part. Now what?

About half of the children in the study can take part in this study. If you are selected [researcher names] will contact you and your parents/ carer to arrange the first interview.

Thank you for reading this!
If you do decide to take part your help will be very helpful to us.

SUBSTUDY CONSENT FORM FOR PARENTS/CARERS

Title of study: BREATHER Qualitative study- Young people and HIV treatment adherence.

Please initial each box if you agree:

I have read and understood the information sheet on the BREATHER qualitative study, version……..	
I have been given the opportunity to ask questions and I am satisfied with the answers that you have given me.	
I ____ agree to my child taking part in this study.	
I agree to my contact details being given to the qualitative study team and understand that they will contact me should my child be selected to take part in the study.	
I understand that the researcher will interview my child two to three times over the course of the study. This will involve them talking with my child for about an hour each time about my child's experience of HIV treatment	
I agree to this interview being audio-recorded.	
I know that my child does not have to talk about things that he/she does not want to talk about. My child can stop talking to the researchers at any time and without giving a reason for this.	
I understand that everything that my child talks to the researchers about is confidential. However if the researchers are told that a young person is at serious risk of harm, they may have to pass this information on to someone else.	
I agree to my child keeping an audio diary for the study, if he/ she would like to.	
I agree to my child giving a copy of her personal diary as it relates to HIV and their treatment, if he/ she would like to.	
I agree that anonymous direct quotes from the interviews or diaries may be used	

My questions have been answered by …………………………………………………………

Name of child……………………………………………………………………………………..

Name of Parent/ Guardian………………………………………………………………………

Signature of Parent/ Guardian (or thumbprint)……………………………………. Date……………………

Name of Witness (if thumbprint used above)………………………………………………………………

Signature of Witness (if thumbprint used above)……………………………………. Date…………………..

Name of Clinician……………………………………………….. .. Date……………………

Signature of Clinician…………………………………………….. Date…………………..

SUBSTUDY CONSENT FORM FOR YOUNG ADULTS

Title of study: BREATHER Qualitative study- Young people and HIV treatment adherence.

Please initial each box if you agree:

I have read and understood the information sheet on the BREATHER qualitative study, version……..	
I have been given the opportunity to ask questions and I am satisfied with the answers that you have given me.	
I ____ agree to taking part in this study.	
I agree to my contact details being given to the qualitative study team and understand that they will contact me should my child be selected to take part in the study.	
I understand that the researcher will interview me two to three times over the course of the study. This will involve them talking with me for about an hour each time about my experience of taking HIV treatment.	
I agree to this interview being audio-recorded.	
I know that I do not have to talk about things that I do not want to talk about. I understand that I can stop talking to the researchers at any time and without giving a reason for this.	
I understand that everything that I talk to the researchers about is confidential.	
I agree to keeping an audio diary for the study.	
I agree to giving a copy of my personal diary as it relates to HIV and my treatment.	
I agree that anonymous direct quotes from the interviews or diaries may be used.	

My questions have been answered by …………………………………………………………

Name ………………………………………………………………………………

Signature (or thumbprint)……………………………………………………….. Date……………………

Name of Witness (if thumbprint used above)……………………………………………………………………

Signature of Witness (if thumbprint used above)……………………………………. Date…………………

Name of Clinician……………………………………………… Date……………………

Signature of Clinician……………………………………….. Date…………………..

SUBSTUDY CONSENT FORM FOR YOUNG ADULTS

Young adults 16-17 years old

Title of study: BREATHER Qualitative study- Young people and HIV treatment adherence.

Please initial each box if you agree:

I have read and understood the information sheet on the BREATHER qualitative study, version…….	
I have been given the opportunity to ask questions and I am satisfied with the answers that you have given me.	
I ____ agree to taking part in this study.	
I agree to my contact details being given to the qualitative study team and understand that they will contact me should my child be selected to take part in the study.	
I understand that the researcher will interview me two to three times over the course of the study. This will involve them talking with me for about an hour each time about my experience of taking HIV treatment.	
I agree to this interview being audio-recorded.	
I know that I do not have to talk about things that I do not want to talk about. I understand that I can stop talking to the researchers at any time and without giving a reason for this.	
I understand that everything that I talk to the researchers about is confidential. However if the researchers are told that a child is at serious risk of harm, they may have to pass this information on to someone else.	
I agree to keeping an audio diary for the study.	
I agree to giving a copy of my personal diary as it relates to HIV and my treatment.	
I agree that anonymous direct quotes from the interviews or diaries may be used.	

My questions have been answered by ………………………………………………………….

Name ………………………………………………………………………………

Signature (or thumbprint) ……………………………………. Date……………………

Name of Witness (if thumbprint used above)………………………………………………………………………

Signature of Witness (if thumbprint used above)……………………………………. Date…………………..

Name of Clinician……………………………………………… Date……………………

Signature of Clinician………………………………………. Date…………………..

SUBSTUDY ASSENT FORM

Title of study: BREATHER Qualitative study- Young people and HIV treatment adherence.

Please tick ONE box for each question:

1. Have you read (or had read to you) the information about this study?	YES ☐ NO ☐
2. Has somebody explained the study to you?	YES ☐ NO ☐
3. Do you understand what the study is about?	YES ☐ NO ☐
4. Have you asked all the questions you want?	YES ☐ NO ☐
5. Have your questions been answered in a way you understand?	YES ☐ NO ☐
6. Do you understand it's OK to stop taking part at any time?	YES ☐ NO ☐
7. Do you understand that you don't have to answer any questions that you do not want to?	YES ☐ NO ☐
8. Do you agree to this interview being audio-recorded?	YES ☐ NO ☐

For the next two questions you can answer no and still take part in the study.

9. Do you want to keep an audio diary for this study?	YES ☐ NO ☐
10. Do you want your personal diary to be included in the study?	YES ☐ NO ☐
And finally,<br>11. Are you happy to take part?	YES ☐ NO ☐

If you **do** want to take part, you can sign your name below.

Your name (or thumbprint where appropriate)…………………………………………………………….
Date……………………………

Name of Witness (if thumbprint used above)……………………………………………………………….
Signature of Witness (if thumbprint used above)……………………….   Date…………………..

The doctor who explained the project to you needs to sign too:

Doctor's name (print) ……………………………………………

Signature ……………………………………………………………………… Date …………………………….

Focus groups- Information sheets and consent forms -BREATHER QUALITATIVE STUDY

Focus groups- INFORMATION SHEETS

12. Parents/ carers
13. Young adults (16-24 years old)
14. Young people and children

Focus groups- CONSENT FORMS

15. Parents/ carers
16. Young adults (16-24 years old)

Focus groups- ASSENT FORM

17. Anyone participating under 16 years old.

Focus groups for Non trial participants - INFORMATION SHEETS

18. Parents/ carers
19. Young people

Focus groups for Non trial participants - CONSENT FORMS

20. Parents/ carers
21. Young Adults 18+

Focus groups for Non trial participants - ASSENT FORM

22. Anyone participating under 16 years old.

BREATHER Qualitative study- Young people and HIV treatment adherence
Focus group: an information sheet for parents/ carers

We have listened carefully to what your child and the other young people have told us. We would now like to invite your child to participate in a focus group to find out what they think of the results of the qualitative study. We would also like to hear what they think that we should do with our results.
We appreciate the time and effort they have given to the study so far.
Before you decide if you are happy for them to take part in these focus groups please read this information sheet carefully and ask any questions you may have.

What is a focus group?
A focus group is a lot like an interview, but instead of being just your child and the researcher having a conversation, like before, it will be with a group of people. This means that there will be 5-8 young people, who have been taking part in the same study as your child, and they will all be taking part in the discussion with the researcher.

How is it different to what they have done before in the study?
We will ask questions but everyone in the group will be able to answer. Your child will still be able to have their say, but they will also be able to hear and respond to what the other young people say.

What would I be asked to do?
The focus group will last no more than two hours. There will be about 5-8 young people there. [researcher names] will ask questions about what everyone in the group thinks about the results of the study and what should be done with them to best support young people taking HIV treatment. As in the rest of the qualitative study your child does not have to answer any questions that they do not want to. They can also stop taking part at any time.

Is the research confidential?
Yes, everything that is said will be treated in the same way as the other information that they have given us. This means that any information that your child share with us will only be seen by members of the research team. Whenever we write or talk about anything your child has told us we never use their real name. All information about you will be stored securely.
The only exception to this is if they tell us something that makes us worried about their or another young person's safety, such as physical harm or neglect.
Everyone taking part will be asked to keep all the information they hear during the focus group private. So they should not talk about the details of the focus group afterwards with their friends or anyone other than the staff at their clinic and the study team. If they would like to talk about anything from the focus group they will be able to talk about it with them.

Why are we holding focus groups?
We think it's important for young people to be involved in every stage of the research, including dissemination. We will talk about the findings of the research and ask the group questions about how they think the research should be used. In particular we would like to learn how your child thinks the research can best be used to inform the design and implementation of adherence interventions for young people. We would also like to learn about how they found being part of the study to inform future approaches to conducting paediatric HIV treatment studies.

What are the benefits and risks to taking part?
All the young people taking part in the focus group will be HIV positive. Your child might find that hearing about other young people's experiences is helpful. Your child will also have the opportunity to be involved in thinking about how to use the research which will mean they are helping to improve services for young people.

By taking part in the focus group your child will be disclosing to the other young people in the room that they are also HIV positive. Your child and all the young people taking part will be asked to agree to respect the confidentiality of the others in the focus group.

Does my child have to take part?
No, your child does not have to take part in a focus group. It will not affect any of the services they currently receive.

Will my child receive anything for taking part?

Yes, your child will receive a £15 gift-voucher. *[Country specific, delete if not applicable]*

Can I have some more information before I decide?
If you would like to find out more before deciding the staff at your clinic can arrange for you to speak to the research team more about it.

Ok, I'm happy to take part. Now what?

Please sign the consent form. [researcher names] will then contact you to arrange the focus group.

Thank you for reading this!

BREATHER Qualitative study- Young people and HIV treatment adherence
Focus group: an information sheet for young adults

Thank you for taking part in the qualitative study so far. We really appreciate the time and energy you have put into it. We have listened carefully to what you and the other young people have told us. We would now like to invite you to a focus group to find out what you think of our results. We would also like to hear from you what you think that we should do with our results.
Before you decide if you want to take part in these focus groups please read this information sheet carefully and ask any questions you may have.

What is a focus group?

A focus group is a lot like an interview, but instead of being just you and the researcher having a conversation, like before, it will be with a group of people. This means that there will be 5-8 young people, who have been taking part in the same study as you, all taking part in the discussion with the researcher.

How is it different to what I have done before in the study?

We will ask questions but everyone in the group will be able to answer. You will still be able to have your say, but you will also be able to hear and respond to what the other young people say.

What would I be asked to do?

The focus group will last no more than two hours. There will be about 5-7 other young people there. [researcher names] will ask questions about what everyone in the group thinks about the results of the study and what should be done with them to best support young people taking HIV treatment. You do not have to answer any questions that you do not want to and can stop taking part at any time.

Is the research confidential?

Yes, everything that is said will be treated in the same way as the other information that you have given us. This means that any information that you share with us will only be seen by members of the research team. Whenever we write or talk about anything you have told us we never use your real name. All information about you will be stored securely.
The only exception to this is if you tell us something that makes us worried about you or another young person's safety, such as physical harm or neglect.
Everyone taking part will be asked to keep all the information they hear during the focus group private. So, like you, they will not be allowed to talk about the focus group afterwards with anyone other than the staff at their clinic and the study team. If you would like to talk about anything from the focus group it will be fine to talk about it with them.

Why are we holding focus groups?

We think it's important for you to be involved in every stage of the research, including dissemination. We will talk about the findings of the research and ask you questions about how you think the research should be used. In particular we would like to learn how you think the research can best be used to inform the design and implementation of adherence interventions for young people.

What are the benefits and risks to taking part?

All the young people taking part in the focus group are also HIV positive. You might find that hearing about other young people's experiences is helpful. You will also have the opportunity to be involved in thinking about how to use the research which will mean you are helping to improve services for young people.
By taking part in the focus group you will be disclosing to the other young people in the room that you are HIV positive. Like you, all the young people will be asked to respect the right to confidentiality of everyone in the focus group.

Do I have to take part?

No, you do not have to take part in a focus group. It will not affect any of the services you currently receive. Thank you for all your help by taking part in the qualitative study.

Will I receive anything for taking part?

Yes, you will receive a £15 gift-voucher. *[Country specific, delete if not applicable]*

Can I have some more information before I decide?

If you would like to find out more before deciding the staff at your clinic can arrange for you to speak to the research team more about it.

Ok, I'm happy to take part. Now what?

Please sign the consent form. [researcher names] will contact you to arrange the focus group.

Thank you for reading this!
If you do decide to take part your help will be very helpful to us.

BREATHER Qualitative study- Young people and HIV treatment adherence
Focus group: an information sheet for young people

Thank you for taking part in the qualitative study so far. We really appreciate the time and energy you have put into it. We have listened carefully to what you and the other young people have told us. We would now like to invite you to a focus group to find out what you think of our results. We would also like to hear from you what you think that we should do with our results.
Before you decide if you want to take part in these focus groups please read this information sheet carefully and ask any questions you may have.

What is a focus group?
A focus group is a lot like an interview, but instead of being just you and the researcher having a conversation, like before, it will be with a group of people. This means that there will be 5-8 young people, who have been taking part in the same study as you, all taking part in the discussion with the researcher.

How is it different to what I have done before in the study?
We will ask questions but everyone in the group will be able to answer. You will still be able to have your say, but you will also be able to hear and respond to what the other young people say.

What would I be asked to do?
The focus group will last no more than two hours. There will be about 5-7 other young people there. [researcher names] will ask questions about what everyone in the group thinks about the results of the study and what should be done with them to best support young people taking HIV treatment. You do not have to answer any questions that you do not want to and can stop taking part at any time.

Is the research confidential?
Yes, everything that is said will be treated in the same way as the other information that you have given us. This means that any information that you share with us will only be seen by members of the research team. Whenever we write or talk about anything you have told us we never use your real name. All information about you will be stored securely.
The only exception to this is if you tell us something that makes us worried about you or another young person's safety, such as physical harm or neglect.
Everyone taking part will be asked to keep all the information they hear during the focus group private. So, like you, they will not be allowed to talk about the focus group afterwards with anyone other than the staff at their clinic and the study team. If you would like to talk about anything from the focus group it will be fine to talk about it with them.

Why are we holding focus groups?
We would like you to help us make the findings as helpful to other young people and their families as we can. The focus group will be an opportunity for you to hear about what all the young people in the study think about what it is like taking HIV treatment. The information from the focus group will be used to guide how we think about how best to use the research to support young people.

What are the benefits and risks to taking part?
All the young people taking part in the focus group are also HIV positive. You might find that hearing about other young people's experiences is helpful. You will also have the opportunity to be involved in thinking about how to use the research which will mean you are helping to improve services for young people.
By taking part in the focus group you will be disclosing to the other young people in the room that you are HIV positive. Like you, all the young people will be asked to respect the confidentiality of everyone in the focus group.

Do I have to take part?
No, you do not have to take part in a focus group. It will not affect any of the services you currently receive. Thank you for all your help by taking part in the qualitative study.

Will I receive anything for taking part?

Yes, you will receive a £15 gift-voucher. *[Country specific, delete if not applicable]*

Can I have some more information before I decide?
If you would like to find out more before deciding the staff at your clinic can arrange for you to speak to the research team more about it.

Ok, I'm happy to take part. Now what?

Please sign the assent form. [researcher names] will contact you and your parents/ carer to arrange the focus group.

Thank you for reading this!
If you do decide to take part your help will be very helpful to us.

SUBSTUDY CONSENT FORM FOR PARENTS/CARERS
Participating in a focus group

Title of study: BREATHER Qualitative study- Young people and HIV treatment adherence.

Please initial each box if you agree:

I have read and understood the information sheet on the focus groups for the BREATHER qualitative study, version……..	
I have been given the opportunity to ask questions and I am satisfied with the answers that you have given me.	
I ____ agree to my child taking part in this focus group.	
I understand that my child will take part in a focus group with other young people. This will involve them talking as a group for about an hour. I agree to this interview being audio-recorded.	
I know that my child does not have to talk about things that he/she does not want to talk about. My child can stop talking to the researchers at any time and without giving a reason for this.	
I understand that everything that my child talks to the researchers about is confidential. However if the researchers are told that a child is at serious risk of harm, they may have to pass this information on to someone else.	
I understand that by participating in the focus group my child will be disclosing to the rest of the group that they are HIV positive. All the participants will be asked to agree to not discuss what they hear from other young people in the focus group with anyone outside the study or their clinic.	
I agree that anonymous direct quotes from the focus group may be used.	

My questions have been answered by ……………………………………………………………
Name of child………………………………………………………………………………………….

Name of Parent/ Guardian………………………………………………………………………………

Signature of Parent/ Guardian (or thumbprint) …………………………………….. Date……………………

Name of Witness (if thumbprint used above)…………………………………………………..

Signature of Witness (if thumbprint used above)………………………………………. Date………………

Name of Clinician…………………………………………………….. Date……………………

Signature of Clinician……………………………………………….. Date…………………..

SUBSTUDY CONSENT FORM FOR YOUNG ADULTS
Participating in a focus group

Title of study: BREATHER Qualitative study- Young people and HIV treatment adherence.

Please initial each box if you agree:

I have read and understood the information sheet on the focus groups for the BREATHER qualitative study, version……..	
I have been given the opportunity to ask questions and I am satisfied with the answers that you have given me.	
I ____ agree to take part in this focus group.	
I agree to this interview being audio-recorded.	
I know that I do not have to talk about things that I do not want to talk about. I can stop talking to the researchers at any time and without giving a reason for this.	
I understand that everything that I and the other young people in the focus group talk to the researchers about is confidential. However if the researchers are told that a child is at serious risk of harm, they may have to pass this information on to someone else.	
All the participants will be asked to agree to not discuss what they hear from other young people in the focus group with anyone outside the study or clinic. I understand that by participating in the focus group I will be disclosing to the rest of the group that I am HIV positive.	
I agree to respect the confidentiality of the others in the focus group. I will not discuss what I hear in the focus group with anyone outside of the study or clinic team.	
I agree that anonymous direct quotes from the focus group may be used.	

My questions have been answered by …………………………………………………………
Name ………………………………………………………………………………

Signature (or thumbprint)……………………………………..   Date……………………

Name of witness (if thumbprint used above)………………………………………………………………………

Signature of Witness…………………………………………………………   Date……………………

Name of Clinician………………………………………………   Date……………………

Signature of Clinician…………………………………………..   Date…………………..

SUBSTUDY ASSENT FORM for focus groups

Title of study: BREATHER Qualitative study- Young people and HIV treatment adherence.

Please tick ONE box for each question:

Have you read (or had read to you) the information sheet about the focus groups?	YES ☐ NO ☐
Has somebody explained the focus groups to you?	YES ☐ NO ☐
Have you asked all the questions you want?	YES ☐ NO ☐
Have your questions been answered in a way you understand?	YES ☐ NO ☐
Do you understand it's OK to stop taking part at any time?	YES ☐ NO ☐
Do you agree to this interview being audio-recorded?	YES ☐ NO ☐
Do you understand that you don't have to answer any questions that you do not want to?	YES ☐ NO ☐
Do you understand that by taking part in this focus group the other young people will know that you are HIV positive?	YES ☐ NO ☐
Do you understand that you must keep what you hear about other people in the focus group private, but that you are able to talk about it to the study team and the staff at your clinic?	YES ☐ NO ☐
Are you happy to take part?	YES ☐ NO ☐

If you **do** want to take part, you can sign your name below.

Your name (or thumbprint if appropriate)……………………………………………… Date……………………

Name of Witness (if thumbprint used above)…………………………………………………………………

Signature of Witness (if thumbprint used above)……………………………………. Date…………………..

The doctor who explained the project to you needs to sign too:

Doctor's name (print) …………………………………………….

Signature ………………………………………………………………… Date …………………………….

Thank you for your help.

PENTA Breather Qualitative study- Young people and HIV treatment adherence
An information sheet for parents/ carers

Focus groups

As part of the PENTA BREATHER trial, we are doing a qualitative study to hear from children and young people about their experiences of HIV treatment. As well as those participating in the trial we would also like to include children and young people (10-21 years old) who are taking HIV treatment but did not participate in the trial.

Before you decide whether you would like your child to participate it is important that you understand why the research is being done and what it involves. Please read this information sheet carefully and ask any questions you may have. You can also ask for one of the study researchers to telephone you should you like to discuss the study in more detail before deciding.

Thank you for taking the time to read this information.

What is the purpose of the study?

The study aims to gain a better understanding of what the day-to-day experience of HIV treatment is like for children and young people. There is little research asking children and young people themselves of their experiences, and this is why we are doing the study. We are especially interested in how they manage taking their treatment day-to-day and any difficulties they have with this, as well as what they think might help support them to take their treatment as prescribed. We are also interested in whether they have heard about the PENTA Breather trial and whether it has made any difference to how they think about HIV treatment and their own adherence. By listening to what children and young people tell us, we hope to suggest ways to improve the experience of treatment including supporting long term adherence to taking HIV treatment.

Who is carrying out the study?

This research is part of the PENTA Breather study. It is therefore organised by the Paediatric European Network for the Treatment of AIDS (PENTA). This qualitative study is being run by a small team of researchers at the London School of Hygiene and Tropical Medicine.

What is involved?

This is a ‘qualitative’ study. The approach assumes that the best way to learn about the experiences of children and young people is to listen carefully to what they have to say. We would therefore like to talk to your child about what their experience of HIV treatment is like and whether the PENTA Breather trial has had any effect on their own experience of HIV treatment.

Your child is being invited to participate in a focus group, which is a group discussion facilitated by the researcher. There will be a maximum of 7 other young people present and the researcher/s. It will last about an hour. We ask that we record the interview unless your child does not want this. Your child does not have to talk about things that he or she does not want to talk about, and they can end the interview at any time and without explaining why.

Is the research confidential?

Yes. All the participants will be asked to agree to not share any information that they hear in the focus group, except to the researchers and the study counsellors.

Any information that your child shares with us will only be seen by members of the research team. This means that whenever we write or talk about anything we have been told we never use your child's real name. All information about your child will be stored securely.

The only information that we may have to pass on is information about children who are at risk of serious harm, such as physical harm or neglect.

How will the research be used?

The findings of this study will be reported to a wide range of people, including those who provide treatment, care and support for children and young people living with HIV. The findings will be used to guide the future implementation of HIV treatment adherence support and HIV treatment trials involving children and young people.

What are the benefits and risks of taking part?

Your child will be part of a study that aims to help young people maintain long term adherence. Your child may see the opportunity to talk about their HIV treatment as an entirely positive experience. For some, however, this is a sensitive topic which may be difficult to talk about. Your child will not need to answer any questions that they do not want to. They are free to withdraw from the focus group at any time. Great care is given to asking questions as sensitively as possible. Support is also available to participants through their clinic, including immediately after the focus group.

Does my child have to take part?

No, your child does not have to take part in the study. It will not affect any of the services your child receives.

Will my child receive anything from taking part?

Yes, all children and young people who take part in the focus group will have their travel expenses covered. If they are accompanied to the focus group venue their escort's travel expenses will also be covered. They will also receive a gift voucher.

Can I have some more information before I decide?

If you would like some more information, the staff at your clinic can arrange for you to speak to a researcher about the study and answer any further questions you may have.

Ok, I'm happy for my child to take part. Now what?

Please sign the consent form and give it to the staff at your clinic. The clinic staff and researcher will be in touch to arrange the focus group.

Thank you for reading this! If you do decide to participate your help will be very valuable to us.

PENTA Breather Qualitative study- Young people and HIV treatment adherence
An information sheet for young people

Focus groups
As part of the PENTA BREATHER trial, we are doing a qualitative study to hear from children and young people about their experiences of HIV treatment. As well as those participating in the trial we would also like to include children and young people (aged 10-21) who are taking HIV treatment but did not participate in the trial. We would like you to join our study.

Before you decide if you want to take part please read this information sheet carefully and ask any questions you may have.

Thank you for taking the time to read this information.

What is the purpose of the study?

This is a study to find out about what it is like for young people to take HIV treatment. This will be done by talking with young people, like you, about their experiences of HIV treatment. This is because we think that the best way to learn about the experiences of young people is to listen carefully to what they have to say.

Why is the study being done?

Until now there hasn't been much research done which asks young people themselves about their experiences of taking HIV treatment. This is why we are doing the study. By listening to what young people tell us we hope to make some suggestions about how to help young people maintain long term adherence to taking their HIV treatment medicines.

Who is doing the study?

This is study is part of the PENTA Breather trial and is organised by the same group. It is being conducted by a small team of researchers at the London School of Hygiene and Tropical Medicine.

What would I be asked to do?

We would like to talk to you in a group with other young people also on HIV treatment about what it is like taking treatment every day. We will also talk about the PENTA Breather trial and whether it has made any difference to how you think about taking treatment every day.

This is called a focus group. There will be a maximum of 7 other young people present and the researcher/s. All the young people will be from the HIV support group that you are in contact with. It will last about an hour. We would like to tape record what you say unless you do not want this. You do not have to talk about things that you do not want to talk about, and you can leave the focus group at any time and without explaining why.

Is the research confidential?

Yes. Everyone in the group will be asked not to say anything about what they hear in the focus group, except to the researchers or the study counsellors. All the participants are involved in a young person HIV support group.

Any information that you share with us will only be seen by members of the research team. This means that whenever we write or talk about anything you have told us we never use your real name. All information about you will be stored securely.

The only exception to this is if you tell us something that makes us worried about you or another young person's safety, such as physical harm or neglect. If we have to tell someone else, we will talk to you about it first.

How will the research be used?

The findings of this study will be reported to a wide range of people who are involved in providing treatment, care and support for children and young people living with HIV. The findings will be used to guide future interventions to support young people to take their HIV treatment effectively for a long time. It will also be used to help people who are conducting other HIV treatment trials with young people.

What are the benefits and risks of taking part?

You will be part of a study that aims to help young people maintain long term adherence. Some young people may find the opportunity to talk about their HIV treatment to be a really good thing. Some young people though might find it difficult to talk about. The researcher is experienced in doing research with young people and will take care to ask questions sensitively. It is up to you what you want to tell us. Also if you want, there can be someone available from your clinic to talk to, including after the focus group.

Do I have to take part?

No, you do not have to take part in the study. It will not affect any of the services you currently receive.

Will I receive anything for taking part?

Yes, all children and young people who take part in the focus group will have their travel expenses covered. If they are accompanied to the focus group venue their escort's travel expenses will also be covered. You will also receive a gift voucher.

Can I have some more information before I decide?

If you would like to find out more before deciding the staff at your clinic can arrange for you to speak to the researcher, Sarah Bernays, more about it.

Ok, I'm happy to take part. Now what?

The clinic staff and researcher will contact you and your parents/ carer to arrange the focus group.

Thank you for reading this!

If you do decide to take part your help will be very helpful to us.

CONSENT FORM FOR PARENTS/ CARERS for focus groups

Title of study: PENTA Breather Qualitative study- Young people and HIV treatment adherence.

Date: May 2014

Version: 1.0

I have read and understood the information sheet on the PENTA Breather qualitative study, version 1.0.	YES	NO
I have been given the opportunity to ask questions and I am satisfied with the answers that you have given me.	YES	NO

I ____ agree to my child taking part in this study.

I agree to my contact details being given to the qualitative study team and understand that they will contact me should my child be selected to take part in the study.	YES	NO
I understand that my child will participate in a focus group. This will involve them talking with my child and up to seven other young people for about an hour about their experiences of HIV treatment.	YES	NO
I agree to the focus group being audio-recorded.	YES	NO
I know that my child does not have to talk about things that he/she does not want to talk about . My child can stop talking to the researchers at any time and without giving a reason for this.	YES	NO
I understand that everything that my child talks to the researchers about is confidential and that my child and other participants will agree to not talking about what anyone else says in the focus group, except for with the researchers and counsellors.	YES	NO
However if the researchers are told that a child is at serious risk of harm, they may have to pass this information on to someone else.	YES	NO
I agree that anonymised direct quotes from the focus group may be used.	YES	NO

My questions have been answered by …………………………………………………………

Name of Parent/ Guardian…………………………………………………………………………

Signature of Parent/ Guardian …………………………………….. Date……………………

CONSENT FORM FOR YOUNG ADULTS for focus group- 18 +

Title of study: PENTA Breather Qualitative study- Young people and HIV treatment adherence.

Date: May 2014

Version: 1.0

I have read and understood the information sheet on the PENTA Breather qualitative study, version……..	YES	NO
I have been given the opportunity to ask questions and I am satisfied with the answers that you have given me.	YES	NO

I ____ agree to taking part in this study.

I agree to my contact details being given to the qualitative study team and understand that they will contact me.	YES	NO
I understand that I will participate in a focus group. This will involve the researcher talking with me and up to seven other young people for about an hour about their experiences of HIV treatment.	YES	NO
I agree to the focus group being audio-recorded.	YES	NO
I know that I do not have to talk about things that I do not want to talk about. I understand that I can stop talking to the researchers at any time and without giving a reason for this.	YES	NO
I understand that everything that I to the group about is confidential. I understand that I and the other participants will agree to not talk about what anyone else says in the focus group, except for with the researchers and counsellors.	YES	NO
I agree that anonymised direct quotes from the focus group may be used.	YES	NO

My questions have been answered by ……………………………………………………………

Name of participant ………………………………………………………………………………

Signature of participant …………………………………….. Date……………………

ASSENT FORM – focus group

Title of study: PENTA Breather Qualitative study- Young people and HIV treatment adherence.

Date: May 2014

Version: 1.0

Please tick yes or no to answer the questions below.

1. Have you read (or had read to you) the information about this study?
……………………………………………………………………………. YES ☐ NO ☐

2. Has somebody explained the study to you? YES ☐ NO ☐

3. Do you understand what the study is about? YES ☐ NO ☐

4. Have you asked all the questions you want? YES ☐ NO ☐

5. Have your questions been answered in a way you understand? YES ☐ NO ☐

6. Do you understand it's OK to stop taking part at any time? YES ☐ NO ☐

7. Do you understand that you don't have to answer any questions that you do not want to?
YES ☐ NO ☐

8. Do you understand that you should not tell anyone what you hear about other people in the focus group, but that you can talk about it with the researchers and clinic counselors?
YES ☐ NO ☐

And finally,

9. Are you happy to take part? YES ☐ NO ☐

If you **do** want to take part, you can sign your name below.

Your name_________________________
Date _________________________

Thank you for your help.

APPENDIX 13: COMMONLY USED ANTIRETROVIRAL DRUGS GROUPED ACCORDING TO THEIR HALF LIVES

From: Taylor et al, 2007 [43]. See original paper for references given in this table.

1676 AIDS 2007, Vol 21 No 13

Table 1. Commonly used antiretroviral drugs grouped according to their half lives.

Drug name	Plasma half-life (h)	Intracellular half-life (h)	Comment	Reference
Nucleoside reverse transcriptase inhibitors				
Zidovudine	0.5–3[d]		Healthy volunteers	[8]
		7[f]	HIV patients	[9]
	1.9 ± 0.73[a]	4.07 ± 1.13[a]	$n = 21$ HIV patients	[10]
Lamivudine		22[f]	HIV patients	[9]
	5–7[d]		Healthy volunteers	[11]
	8.65 (8.53–9.80)[c]	18.4[g]	60 Healthy volunteers	[12]
Stavudine	1.3–1.4[b]			[13]
		7[g]	28 HIV patients	[14]
Didanosine	0.6–2.8[d]			[15]
		8–40[d]		[16]
Abacavir	0.8–1.5[d]			[17]
		18 (12–19)[c]		[18]
	2.59[f]	20.6[f]		[19]
		>12[f]	5 HIV patients	[20]
Tenofovir	12–15[d]		HIV patients	[21,22]
		150 (60–>175)[c]	15 HIV patients	[18,22]
Emcitritabine	8.24 (31)[h]	39[g]	8 HIV patients on 200 mg/day	[23]
				[24]
	10.2 (19)[h]		6 Healthy volunteeers 200 mg/day	
Non-nucleoside reverse transcriptase inhibitors				
Efavirenz	40–55[f]	n/a	Multiple dose	[25]
	36–100[d]		10 HIV patients	[3]
Nevirapine	25–30[f]	n/a	Multiple dose	[26]
	56.7 (25.6–164)[c]		Single dose to 44 female healthy volunteers	[27]
TMC125[i]	29.35 (20.50–87.11)[c]	n/a	16 HIV patients on 900 mg bid	[28]
TMC278[i]	43.6 ± 12.2[a]	n/a	Healthy volunteers fed state	[29]
Protease inhibitors				
Saquinavir	2.79[e]		1000/100 mg bid HIV patients	[30]
	4.5 (2.5–9.3)[c]	5.9 (4.0–17.7)[c]	1600/100 mg/day in 12 HIV patients	[31]
Ritonavir	3.7[e]	n/a	100 mg bid with saquinavir 1000 mg	[30]
	4.1 (2.6–8.3)[c]	6.2 (3.9–18.6)[c]	100 mg/day with saquinavir 1600 mg	[31]
Indinavir	1.8 ± 0.4[a]		Unboosted	[32]
	2.12[e]		800/100 mg bid	[33]
	1.2 ± 0.09[a]	2.0 ± 0.3[a]	Unboosted 800 mg tid, 10 HIV patients	[34]
Nelfinavir	3.5–5[d]			[35]
	3.8 (2.0–6.4)[c]	3.1 (1.6–4.4)[c]		[36]
M8[k]	3.4 (2.1–10.1)[c]	3.5 (1.5–5.5)[c]		[36]
Atazanavir	7.9 ± 2.9[a]		400 Healthy volunteers	[37]
	6.5 ± 2.6[a]		HIV	
	18.1 ± 6.2[a]		300/100 Healthy volunteers	
	8.6 ± 2.3[a]		HIV patients	
	9.80[e]		200 mg/day boosted plus saquinavir	[38]
	7.5 (4.6–19.5)[c]	12.1 (3.3–22.3)[c]		[39]
Fosamprenavir	7.7[f]	n/a		[40]
	6.17[e]		700/100 mg bid in 18 HIV patients	[41]
Lopinavir	5–6[f]		400/100 mg bid	[42]
		6.5[c]		J. Ford (unpublished)
Tipranavir	6.0[b]	n/a	Boosted by 200 mg ritonavir bid in HIV patients	[43]
Darunavir	10.9–17.2[d]	n/a	Different ritonavir 100 mg boosted doses	[44]
Fusion entry inhibitor				
Enfuvirtide	3.8 ± 0.6[a]	n/a	Subcutaneous administration of 90 mg	[45]

Table 1 (*continued*)

Drug name	Plasma half-life (h)	Intracellular half-life (h)	Comment	Reference
CCR5 antagonists				
Maraviroc[i]	16–23[f]	n/a	Data obtained from different dose studies in healthy volunteers – chosen dose 150 mg bid	[46]
Vicriviroc[i]	28–33[b]	n/a	HIV patients	[47]

[a]Mean ± SD.
[b]Mean.
[c]Median (range).
[d]Range.
[e]Geometric mean.
[f]Not reported.
[g]Median.
[h]Mean (% coefficient of variation).
[i]Investigational agent.
[k]Active metabolite of nelfinavir.

APPENDIX 14: PENTA COMMITTEE STRUCTURE

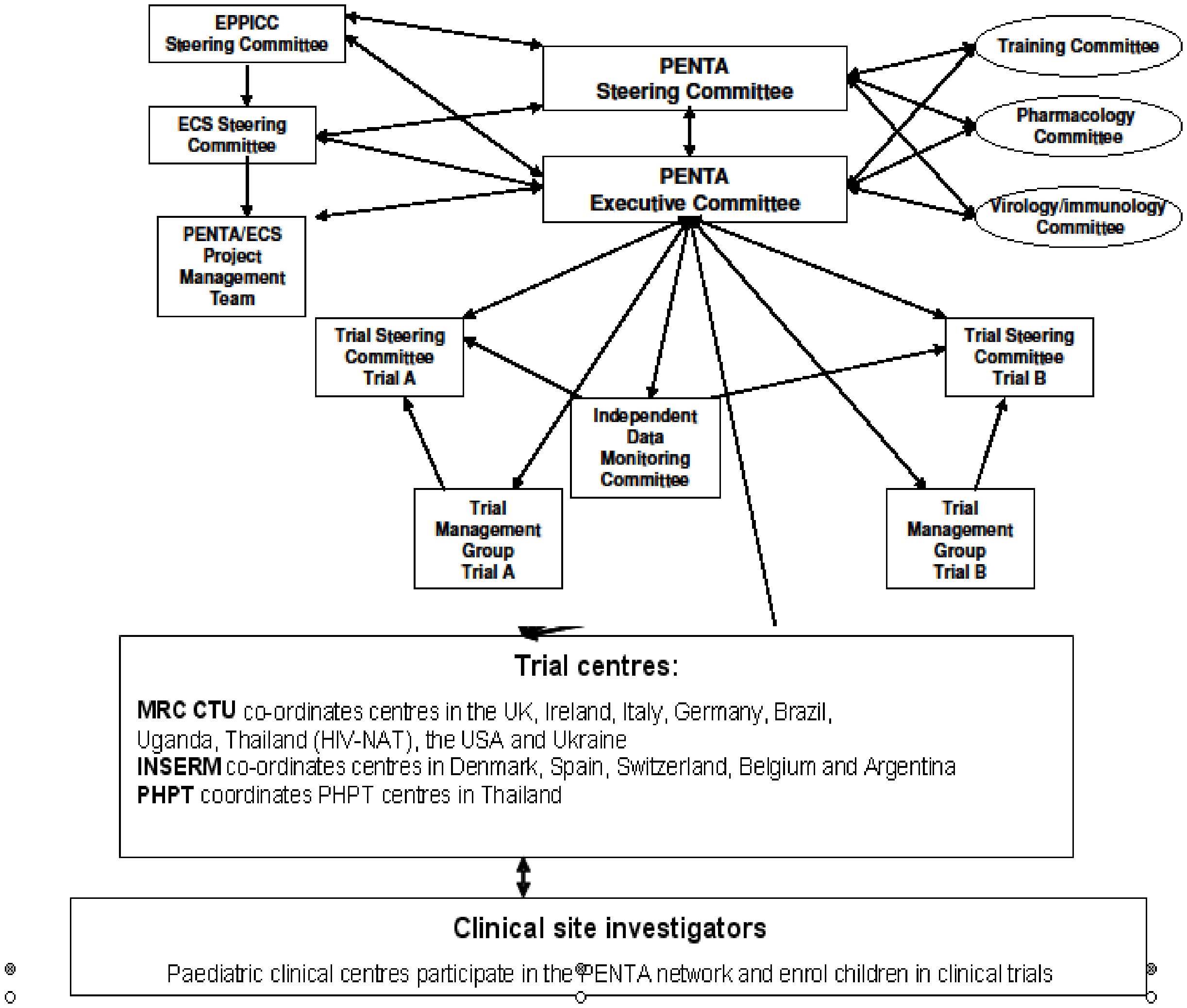

APPENDIX 15: FLOW CHART FOR MANAGING VIRAL LOADS

PILOT PHASE (SCT GROUP ONLY)

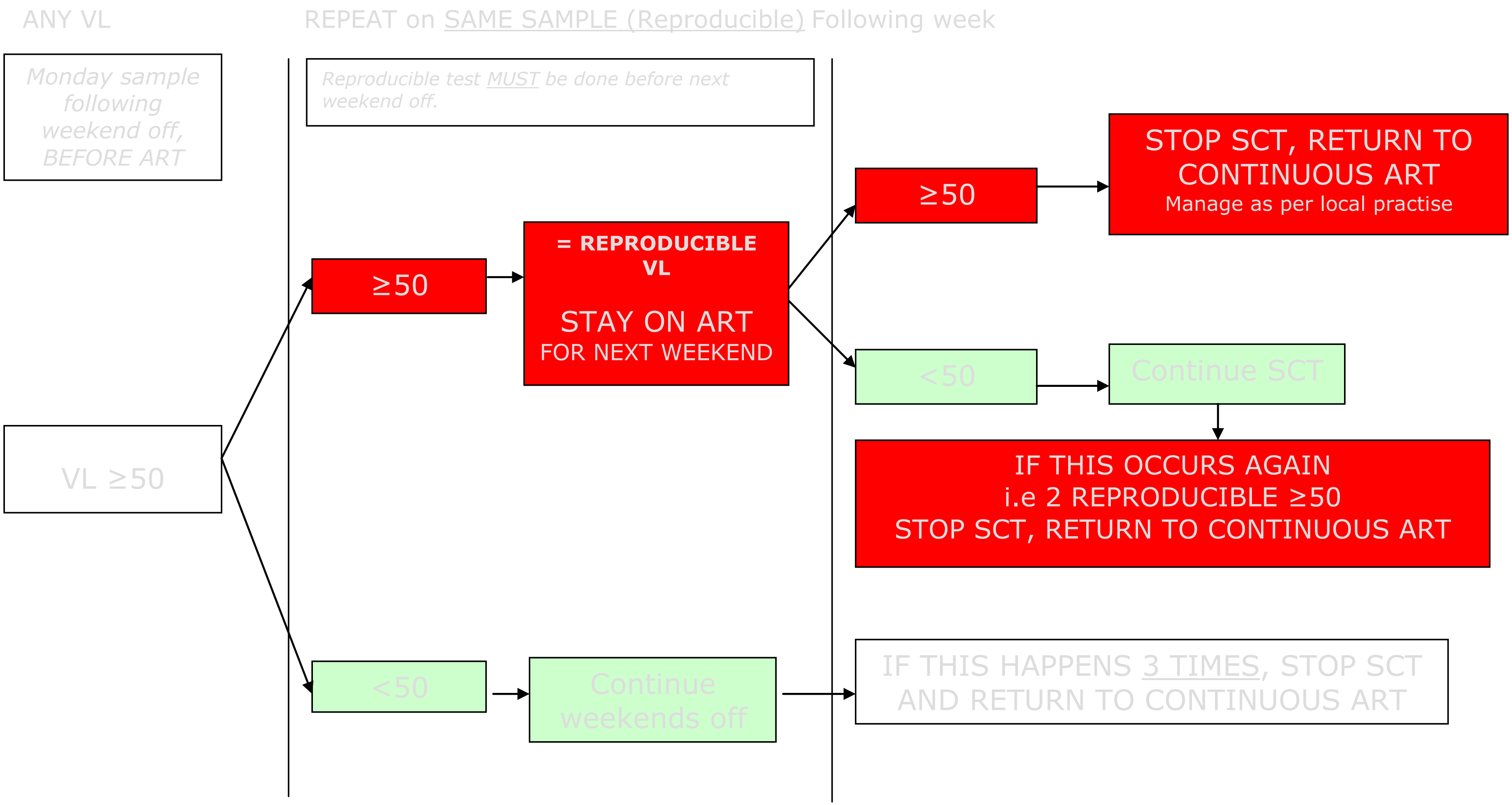

MAIN TRIAL AND LONG TERM FOLLOW UP (All participants)

ANY VL
(Week 4 onwards)

REPEAT on SEPARATE SAMPLE (within 1 week of VL ≥50 being reported)

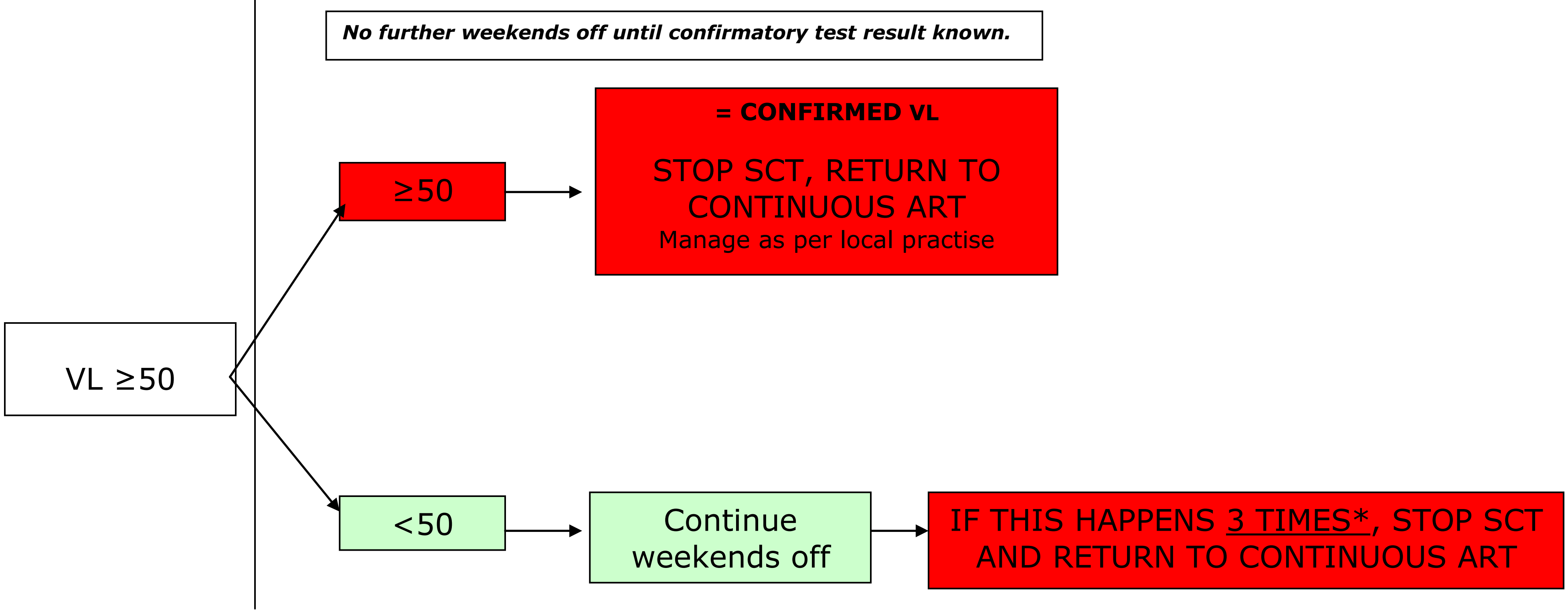

NOTE: Participants in the CT arm should have a repeat VL on a separate sample within 1 week if any VL ≥ 50 is reported. If confirmed VL ≥ 50, manage as per local practice

Where a VL is reported as <XX where XX > 50 (e.g. <80 copies/ml), this should be treated as a possible VL >50 copies/ml. The participant should have a repeat VL on a separate sample within 1 week to confirm if VL < 50, or a retest on a stored sample. If in the SCT arm, the participant should not continue weekends off until the repeat result is known.

APPENDIX 16: TRIAL MANAGEMENT GROUP

- **Chief Investigator**

Karina Butler, MB
Consultant Paediatrician
Our Lady's Children's Hospital
Crumlin
Dublin 12
Ireland

Tel: + 353 1 4096 338
Fax: + 353 1 4096 376
E-mail: karina.butler@olhsc.ie

- **Medical experts**

Karina Butler, MB
Consultant Paediatrician
Our Lady's Children's Hospital
Crumlin
Dublin 12
Ireland

Tel: + 353 1 4096 338
Fax: + 353 1 4096 376
E-mail: karina.butler@olhsc.ie

Diana Gibb, MD
Professor of Epidemiology /
Honorary Consultant Paediatrician
MRC Clinical Trials Unit at UCL
Aviation House, 125 Kingsway
London, WC2B 6NH, UK

Tel: + 44 20 7670 4709
Fax: + 44 20 7670 4818
E-mail: Diana.Gibb@ucl.ac.uk

Alexandra Compagnucci, MD
INSERM SC10-US019
16 Avenue Paul Vaillant Couturier
Villejuif Cedex, 94807, France

Tel: + 33 1 4559 5290
Fax: + 33 1 4559 5180
E-mail: alexandra.compagnucci@inserm.fr

Victor Musiime, MD
Joint Clinical Research Centre
P.O.Box 10005 Kampala, Uganda

Tel: + 256 414 270283
Fax: + 256 41 342632
Email:vmusiime@jcrc.org.ug

Esse Menson, MD
Consultant Paediatrician
Evelina Children's Hospital
St. Thomas' Hospital
Lambeth Palace Road
London, SE1 7EH, UK

Tel: + 44 20 7188 4677
Fax: + 44 20 7188 4612
Email: esse.menson@gsst.nhs.uk

Julia Kenny
Senior Clinical Research Fellow
Infectious Diseases and Microbiology
Institute of Child Health
30 Guilford Street
London, WC1N 1EH

Tel: +44 20 7905 2892
Fax: +44 20 7905 2882
Email: j.kenny@ucl.ac.uk

Kulkanya Chokephaibulkit, MD
Consultant Paediatrician
Department of Paediatrics, Faculty of Medicine
Siriraj Hospital
Mahidol University
2 Prannok Rd, Bangkok-noi
Bangkok 10700, Thailand

Tel: +66 (0) 2418 0544
Email: sikch@mahidol.ac.th

Anna Turkova
Honorary Clinical Fellow
MRC Clinical Trials Unit at UCL
Aviation House, 125 Kingsway
London, WC2B 6NH, UK
Tel: +44 20 7670 4786
Fax: +44 20 7670 4814
Email: a.turkova@ucl.ac.uk

- **Trial centres - Trial management, randomisations & SAE notification**

For UK, Ireland, Germany, Thailand (HIV-NAT), Uganda, USA, Ukraine

Karen Scott
Lynda Harper
MRC Clinical Trials Unit at UCL
Aviation House, 125 Kingsway
London, WC2B 6NH, UK
All queries: penta.mrcctu@ucl.ac.uk
Tel: + 44 20 7670 4939
Fax: + 44 20 7670 4814
Email: karen.scott@ucl.ac.uk
Email: Lynda.Harper@ucl.ac.uk

For Spain, Denmark, Belgium, Argentina

All queries: penta.sc10@inserm.fr
Yoann Riault
INSERM SC10-US019
16 Avenue Paul Vaillant Couturier
Villejuif Cedex, 94807, France
Tel: + 33 1 4559 5255
Fax: + 33 1 4559 5180
Email: yoann.riault@inserm.fr

For Thailand (PHPT sites)

Suwalai Chalermpantmetagul
Program for HIV Prevention and
Treatment (PHPT)/IRD 174
187/10, Changklan Road
Changklan, Muang
Chiang Mai 50100
Thailand
Tel: +66 (0) 5381 91 25/26/27/28/29
Fax: + 66 (0) 5381 9130
Email: Suwalai@phpt.org

- **Trial statisticians**

Abdel Babiker
MRC Clinical Trials Unit at UCL
Aviation House, 125 Kingsway
London, WC2B 6NH, UK
Tel: + 44 20 7670 4733
Fax: + 44 20 7670 4818
E-mail: A.Babiker@ucl.ac.uk

Yacine Saidi
16 Avenue Paul Vaillant Couturier
INSERM SC10-US019
Villejuif Cedex, 94807, France
Tel: + 33 1 4559 5284
Fax: + 33 1 4559 5180
Email: yacine.saidi@inserm.fr

Jamie Inshaw
MRC Clinical Trials Unit at UCL
Aviation House, 125 Kingsway
London, WC2B 6NH, UK
Tel: + 44 20 7670 4650
Fax: + 44 20 7670 4814
E-mail: j.inshaw@ucl.ac.uk

- **Immunology and Virology advisory group**

Eleni Nastouli, UCL, London, UK
Anita De Rossi, University of Padova, Italy
Nigel Klein, Institute of Child Health, London, UK
Maria Angeles Munoz Fernandez, Hôpital General, Madrid, Spain
Nicole Ngo, Chiang Mai, Thailand

- **Qualitative substudy group**

Tim Rhodes, London School of Hygiene and Tropical Medicine, London, UK
Sarah Bernays, London School of Hygiene and Tropical Medicine, London, UK
Ali Judd, MRC Clinical Trials Unit, London, UK
Victor Musiime, JCRC, Uganda

- **Trial Management Group (TMG) for the BREATHER trial**

Karina Butler	Carlo Giaquinto	Sharon Storey
Diana Gibb	Tim Cressey	Julia Kenny
Alexandra Compagnucci	Victor Musiime	Esse Menson
Jintanat Ananworanich	José Ramos Amador	Anna Turkova

Plus, trial management and statistics representatives from trials units

Trial Steering Committee (TSC) for the BREATHER trial

Karina Butler
Diana Gibb
Jintanat Ananworanich
Victor Musiime
Ian Weller (Chair)
José Ramos Amador
Polly Clayden
Valériane Leroy
Janet Darbyshire

Independent Data Monitoring Committee for the BREATHER trial

- Genevieve Chène –INSERM, France
- Anton Pozniak –Chelsea & Westminster Hospital, UK (Chair)
- Stefano Vella –Istituto Superiore di Sanità, Italy
- Timo Vesikari –University of Tampere, Finland

APPENDIX 17: SAMPLE PATIENT INFORMATION SHEETS FOR LONG TERM FOLLOW UP (FOR YOUNG PEOPLE ON SCT)

i. Parents/carers
ii. Young adults (16 years and over)
iii. Young people (11 to 15 years)
iv. Children (8 to 10 years)
v. Children (unaware of diagnosis) – for use at local care team's discretion

All to be printed on local headed paper, include page numbers and include local requirements

BREATHER (PENTA 16)
Long term follow up for young people on SCT

AN INFORMATION SHEET FOR PARENTS/CARERS

We would like to thank you and your child for taking part in the BREATHER study which is looking at two different ways of giving anti-HIV medicines; either taking the medicines every day (the current standard way of treating HIV), or taking two days a week off treatment. This way of taking anti-HIV medicines is called **Short Cycle Therapy** (SCT).

1. HOW IS THE STUDY PROGRESSING?

Your child is now finishing the main BREATHER study and we thank you both for all your time and for completing the questionnaires and allowing us to take some extra blood samples. As it takes several months to check and combine all the information from everyone in the study, we are not yet able to give any results of the study. However as soon as we have these results, your doctor will discuss them with you. This is likely to be in early 2015.

2. WHAT HAPPENS NOW?

Your child was in the group taking two days a week off anti-HIV treatment and is doing well with a very low (suppressed) level of HIV virus in the blood (viral load). The Scientific Committees which monitor the study feel happy for children like yours to continue taking two days a week off treatment if:

- you, your child and your doctor are happy about this
- your child continues to come to clinic every 12-16 weeks to check the virus is still low and will come back to clinic for retesting if the level of virus increases
- your child continues on anti-HIV treatment which includes efavirenz (Sustiva)
- your child continues to take the same two days off treatment as agreed with your doctor
- Your child does not take days off treatment unless your doctor agrees

The decision for your child to continue taking 2 days off treatment must be discussed with your doctor before you go ahead. It is important that other children or young people do not change their anti-HIV medication by themselves.
When results from the study are available we will let you know whether or not it is OK for your child to continue taking weekends off.

3. WHAT IS LONG TERM FOLLOW UP OF THE BREATHER STUDY, AND WHY IS IT BEING DONE?

As BREATHER is the only study of its kind in children and young people, we would like to continue to collect further information about your child's health each year for the next two years. This information will cover any illnesses s/he may have as well as any changes in his/her HIV drugs and blood results from your regular clinic visits including viral load. It's important that your child's viral load is tested every 12-16 weeks.

As part of this follow up, one 6ml blood sample will be stored at the final visit in two years' time. The blood will be tested to find out how the HIV virus responded to SCT. Some of these tests may look at resistance to the medicines, but new tests are being developed all the time. We are therefore asking for you to agree that this stored blood sample can be used in future for tests relevant to HIV. These would be decided by a group of experts in PENTA.

We would like you to complete adherence questionnaires every 12-16 weeks for the next two years and acceptability questionnaires if your child re-starts continuous therapy or at the end of the study.

4. PARTICIPATION

Your continued participation in this study is entirely voluntary. If you decide that you do not wish your child to take part that is entirely your right. Your decision will in no way affect your present or future treatment.
If you decide for your child not to take part, he/she should return to continuous therapy.

You can withdraw your child from the study at any time without giving a reason. Withdrawal will not affect your child's medical care. However, if you decide against attending for 12-16 weekly visits, your child should return to continuous therapy.

Before you decide, it is important for you to understand why the research is being done and what it will involve. Please take time to read this information carefully and discuss it with your friends, relatives and your doctor or clinic nurse if you wish. Ask us if there is anything that is not clear or if you would like more information. Take as much time as you want to decide whether or not you wish your child to take part.

5. WHAT ARE THE POSSIBLE BENEFITS AND DISADVANTAGES OF TAKING PART?

You can continue not to give anti-HIV medicines 2 days a week if you, your child and your doctor agree, but you will have to come to clinic every 12-16 weeks to check your child's viral load, which may be more often than routine standard of care at your clinic.

We cannot promise that participating will directly help your child. However, all the information we get might help children and young people with HIV in the future.

6. CONFIDENTIALITY

All information collected during the BREATHER study will continue to be anonymised and confidential. Names will not be used, and we will continue to just use your child's BREATHER study number and date of birth for identification. Hospital notes will only be made available to study staff and confidentiality will be maintained at all times. Identification will be by study number only. Should the results of this study be reported or published your child's name will not be used. The study will be conducted in compliance with the UK Data Protection Act, 1998 and the data will be stored for 20 years.

7. WHAT ELSE DO I NEED TO KNOW?

PENTA has made arrangements for compensation should your child come to any harm because of being in the study and not because of any problem with the way the study is being carried out Your hospital doctor is not receiving any payment for carrying out this research.

8. CONTACT

If you have any concerns or other questions about the study or the way it has been carried out, or if your child has an injury or illness during participation in the study, please contact the nurse at the hospital where the study is being carried out:

Name:…………………………………………..

Contact details:……………………………………………..………………………………

Thank you for taking time to consider this. Please ask any questions and let us know if there are things that you do not understand, or would like more information about (contact number above).

BREATHER
Long term follow up for young people on SCT

AN INFORMATION SHEET FOR YOUNG ADULTS

We would like to thank you for taking part in the BREATHER study which is looking at two different ways of giving anti-HIV medicines; either taking the medicines every day (the current standard way of treating HIV), or taking two days a week off treatment. This way of taking anti-HIV medicines is called **Short Cycle Therapy** (SCT).

1. HOW IS THE STUDY PROGRESSING?

You are now finishing the main BREATHER study and we thank you for all your time and for completing the questionnaires and allowing us to take some extra blood samples. As it takes several months to check and combine all the information from everyone in the study, we are not yet able to give any results of the study. However as soon as we have these results, your doctor will discuss them with you. This is likely to be in early 2015.

2. WHAT HAPPENS NOW?

You were in the group taking two days a week off anti-HIV treatment and are doing well with a very low (suppressed) level of HIV virus in the blood (viral load). The Scientific Committees which monitor the study feel happy for you to continue taking two days a week off treatment if:

- you and your doctor are happy about this
- you continue to come to clinic every 12-16 weeks to check the virus is still low and will come back to clinic for retesting if the level of virus increases
- you continue on anti-HIV treatment which includes efavirenz (Sustiva)
- you continue to take the same two days off treatment as agreed with your doctor
- You do not take days off treatment unless your doctor agrees

The decision for you to continue taking 2 days off treatment must be discussed with your doctor before you go ahead. It is important that other children or young people do not change their anti-HIV medication by themselves.
When results from the study are available we will let you know whether or not it is OK for you to continue taking weekends off.

3. WHAT IS LONG TERM FOLLOW UP OF THE BREATHER STUDY, AND WHY IS IT BEING DONE?

As BREATHER is the only study of its kind in children and young people, we would like to continue to collect further information about your health each year for the next two years. This information will cover any illnesses you may have as well as any changes in your HIV drugs and blood results from your regular clinic visits including viral load. It's important that your viral load is tested every 12-16 weeks.

As part of this follow up, one 6ml blood sample will be stored at the final visit in two years' time. The blood will be tested to find out how the HIV virus responded to SCT. Some of these tests may look at resistance to the medicines, but new tests are being developed all the time. We are therefore asking for you to agree that this stored blood sample can be used in future for tests relevant to HIV. These would be decided by a group of experts in PENTA.

We would like you to complete adherence questionnaires every 12-16 weeks for the next two years and an acceptability questionnaire if you re-start continuous therapy or at the end of the study.

4. PARTICIPATION

Your continued participation in this study is entirely voluntary. If you decide that you do not wish to take part that is entirely your right. Your decision will in no way affect your present or future treatment.
If you decide not to take part, you should return to continuous therapy.

You can withdraw from the study at any time without giving a reason. Withdrawal will not affect your medical care. However, if you decide against attending for 12-16 weekly visits, you should return to continuous therapy.

Before you decide, it is important for you to understand why the research is being done and what it will involve. Please take time to read this information carefully and discuss it with your friends, relatives and your doctor or clinic nurse if you wish. Ask us if there is anything that is not clear or if you would like more information. Take as much time as you want to decide whether or not you wish to take part.

5. WHAT ARE THE POSSIBLE BENEFITS AND DISADVANTAGES OF TAKING PART?

You can continue not to take anti-HIV medicines 2 days a week if you and your doctor agree, but you will have to come to clinic every 12-16 weeks to check your viral load, which may be more often than routine standard of care at your clinic.

We cannot promise that participating will directly help you. However, all the information we get might help children and young people with HIV in the future.

6. CONFIDENTIALITY

All information collected during the BREATHER study will continue to be anonymised and confidential. Names will not be used, and we will continue to just use your BREATHER study number and date of birth for identification. Hospital notes will only be made available to study staff and confidentiality will be maintained at all times. Identification will be by study number only. Should the results of this study be reported or published your name will not be used. The study will be conducted in compliance with the UK Data Protection Act, 1998 and the data will be stored for 20 years.

7. WHAT ELSE DO I NEED TO KNOW?

PENTA has made arrangements for compensation should you come to any harm because of being in the study and not because of any problem with the way the study is being carried out
Your hospital doctor is not receiving any payment for carrying out this research.

8. CONTACT

If you have any concerns or other questions about the study or the way it has been carried out, or if you have an injury or illness during participation in the study, please contact the nurse at the hospital where the study is being carried out:

Name:…………………………………………..

Contact details:…………………………………………..…………………………….

Thank you for taking time to consider this. Please ask any questions and let us know if there are things that you do not understand, or would like more information about (contact number above).

BREATHER
Long term follow up for young people on SCT

AN INFORMATION SHEET FOR YOUNG PEOPLE

We would like to thank you for taking part in the BREATHER study which is looking at two different ways of giving anti-HIV medicines; either taking the medicines every day (the current standard way of treating HIV), or taking two days a week off treatment. This way of taking anti-HIV medicines is called **Short Cycle Therapy** (SCT).

1. HOW IS THE STUDY PROGRESSING?

You are now finishing the main BREATHER study and we thank you for all your time and for completing the questionnaires and allowing us to take some extra blood samples. As it takes several months to check and sort all the information from everyone in the study, we are not yet able to give any results of the study. However as soon as we have these results, your doctor will discuss them with you and your family. This is likely to be in early 2015.

2. WHAT HAPPENS NOW?

You were in the group taking two days a week off anti-HIV treatment and are doing well with a very low (suppressed) level of HIV virus in the blood (viral load). The Scientific Committees which monitor the study feel happy for you to continue taking two days a week off treatment if:

- you and your doctor are happy about this
- you continue to come to clinic every 3-4 months to check the virus is still low and will come back to clinic for retesting if the level of virus increases
- you continue on anti-HIV treatment which includes efavirenz (Sustiva)
- you continue to take the same two days off treatment as agreed with your doctor
- You do not take days off treatment unless your doctor agrees

The decision for you to continue taking 2 days off treatment must be discussed with your doctor before you go ahead. It is important that other children or young people do not change their anti-HIV medication by themselves.
When results from the study are available we will let you know whether or not it is OK for you to continue taking weekends off.

3. WHAT IS THE LONG TERM FOLLOW-UP OF THE BREATHER STUDY?

As BREATHER is the only study of its kind in children and young people, we would like to continue to collect further information about your health each year for the next two years. This information

will cover any illnesses you may have as well as any changes in your HIV drugs and blood results from your normal clinic visits including viral load. It's important that your viral load is tested every 3-4 months.

4. WHAT INFORMATION WILL BE COLLECTED?

If you agree, your doctor will send information from your clinic visits):
- Any illnesses you have
- Your anti-HIV drugs
- Blood test results

Clinic visits will be every 3-4 months so that the level of virus in your blood can be checked.

One blood sample will be stored at the final visit in the study in two years' time. The blood will be used in future for tests relevant to HIV.

We would also like you to complete adherence questionnaires every 3-4 months for the next two years and acceptability questionnaires if you re-start taking anti-HIV medicines every day or at the end of the study

5. DO I HAVE TO TAKE PART?

If at any time you don't want to do the research anymore, just tell your parents, doctor or nurse. Your treatment at the clinic will not change.
However, if you do decide you don't want to take part you should take your HIV drugs as instructed by your doctor.

6. WILL MY MEDICAL DETAILS BE KEPT PRIVATE?

All information collected about you during this study will still be confidential and anonymous. This means information about you will be known by a number only. Your name will not be used.

Thank you for reading this. Please ask any questions and let us know if there are things that you do not understand, or would like more information about.

BREATHER
Long term follow up for young people on SCT

AN INFORMATION SHEET FOR CHILDREN

Thank you for taking part in the BREATHER study. The study is looking at two different ways of giving anti-HIV medicines; either taking the medicines every day (the current standard way of treating HIV), or taking two days a week off. This way of taking anti-HIV medicines is called **Short Cycle Therapy** (SCT).

1. HOW IS THE STUDY PROGRESSING?

You are now finishing the main BREATHER study and we thank you for all your time and coming to the visits. It will take several months to check and sort all the information collected during the study. Your doctor will talk to you and your family about the results once they are known.

2. WHAT HAPPENS NOW?

You were in the group taking two days a week off anti-HIV treatment and are doing well with a very low level of virus in the blood. The Scientific Committees which monitor the study feel happy for you to continue taking two days a week off treatment if:

- you and your doctor are happy about this
- you come to clinic every 3-4 months to check the virus is still low and will come back to clinic if the level of virus has gone up
- you continue on anti-HIV treatment which includes efavirenz (Sustiva)
- you continue to take the same two days off treatment as agreed with your doctor
- You do not take days off treatment unless your doctor agrees

You should only continue to take 2 days off treatment if your doctor and family agree. It is important that other children or young people do not change their anti-HIV medication by themselves.
When we have results from the study we will let you know whether or not it is OK for you to continue taking weekends off.

3. WHAT IS THE LONG TERM FOLLOW-UP OF THE BREATHER STUDY?

We would like to continue to collect further information about your health each year for the next two years. The information we get will help us understand more about how HIV affects your life. It's important that the level of virus in your blood is checked every 3-4 months.

4. WHAT INFORMATION WILL BE COLLECTED?

If you agree, your doctor will send information from your clinic visits:

- Any illnesses you have
- Your anti-HIV drugs
- Blood test results

Clinic visits will be every 3-4 months so that the level of virus in your blood can be checked.

One blood sample will be stored at the final visit in the study in two years' time.

You will need to complete an adherence questionnaire every 3-4 months for the next two years and acceptability questionnaires if you re-start taking anti-HIV medicines every day or at the end of the study.

5. DO I HAVE TO TAKE PART?

If at any time you don't want to do the research anymore, just tell your parents, doctor or nurse. Your care at the clinic will not change.
If you don't take part, you should take your anti-HIV drugs the way that your doctor tells you to.

6. WILL MY MEDICAL DETAILS BE KEPT PRIVATE?

All information collected about you during this study will still be confidential and anonymous. This means information about you will be known by a number only. Your name will not be used.

Thank you for reading this. Please ask any questions and let us know if there are things that you do not understand, or would like more information about.

BREATHER
Long term follow up for young people on SCT

AN INFORMATION SHEET FOR CHILDREN TAKING MEDICINES LONG TERM

Thank you for taking part in the BREATHER study. The study is looking at two different ways of giving medicines to children like you; either taking the medicines every day or taking two days a week off. This way of taking medicines is called **Short Cycle Therapy** (SCT).

1. HOW IS THE STUDY PROGRESSING?

You are now finishing the main BREATHER study and we thank you for all your time and coming to the visits. It will take several months to check and sort all the information collected during the study. Your doctor will talk to you and your family about the results once they are known.

2. WHAT HAPPENS NOW?

You were in the group taking two days a week off medicines and are doing well. The groups that monitor the study feel happy for you to continue taking two days a week off medicines if:

- you and your doctor are happy about this
- you come to clinic every 3-4 months so that we can check you are still doing well and come back to clinic if your doctor or nurse asks you to
- you are still taking certain medicines (you can ask your doctor or nurse about this)
- you take the same two days off treatment as agreed with your doctor
- you do not take days off treatment unless your doctor agrees

You should only continue to take 2 days off medicines if your doctor and family agree. It is important that other children or young people do not change the way they take medicines by themselves.
When we have results from the study we will let you know whether or not it is OK for you to continue taking weekends off.

3. WHAT IS THE LONG TERM FOLLOW-UP OF THE BREATHER STUDY?

We would like to continue to collect further information about your health each year for the next two years. The information we get will help us understand more about how we can help children like you. It's important that you come to clinic every 3-4 months so that we can check you are well.

4. WHAT INFORMATION WILL BE COLLECTED?

If you agree, your doctor will send information from your clinic visits:

- Any illnesses you have
- Your medicines
- Blood test results

Clinic visits will be every 3-4 months so that some blood can be taken to check you are still doing well.

One blood sample will be stored at the final visit in the study in two years' time.

An adherence questionnaire will need completing every 3-4 months over the next two years

5. DO I HAVE TO TAKE PART?

If at any time you don't want to do the research anymore, just tell your parents, doctor or nurse. Your care at the clinic will not change.
If you don't take part, you should take your medicines the way that your doctor tells you to.

6. WILL MY MEDICAL DETAILS BE KEPT PRIVATE?

All information collected about you during this study will still be confidential and anonymous. This means information about you will be known by a number only. Your name will not be used.

Thank you for reading this. Please ask any questions and let us know if there are things that you do not understand, or would like more information about.

APPENDIX 18: SAMPLE CONSENT FORMS FOR LONG TERM FOLLOW UP (FOR YOUNG PEOPLE ON SCT)

i. Parents/carers
ii. Young adults (16 years and over)
iii. Assent form for young people/children

All to be printed on local headed paper, include page numbers and include local requirements

Consent Form for parents/carers

BREATHER Long term follow for young people on SCT

Please write your initials in each box if you agree:

I have read and understood the information sheet on the BREATHER study long term follow up for SCT arm, version……, dated……..	
I understand the potential benefits and disadvantages of my child participating in the study long term follow up.	
The details of the study long term follow up have been explained by: Dr…………………………………who has answered my questions satisfactorily.	
I agree to my child's routine blood results and clinical information being collected for long term follow up	
I agree to my child's viral load being measured every 12-16 weeks	
I agree to completion of adherence questionnaires every 12-16 weeks during the long term follow up and acceptability questionnaires if my child re-starts continuous therapy or at the end of the study	
I agree to a blood sample being taken at the final visit to be used in future for tests relevant to HIV	
I agree to my child continuing not to take anti-HIV medicines on ____________ and _____________(specify days of week) if also agreed by my child and their doctor and while the viral load remains low.	
I know that my child can withdraw from the long term follow up at any time without it affecting his/her care and that he/she would need to take anti-HIV drugs as instructed by his/her doctor.	

Name of child/young person:____

Name of Parent/Guardian: ____

Signature of Parent/Guardian:_____________________________Date:______

Name of clinician:_______________________________________

Signature of clinician:____________________________________Date:_______

CONSENT FORM FOR YOUNG ADULTS

BREATHER Long term follow up foryoung people on SCT

Please write your initials in each box if you agree:

I have read and understood the information sheet on the BREATHER study long term follow up for SCT arm, version….., dated……..	
I understand the potential benefits and disadvantages of participating in the study long term follow up.	
The details of this study have been explained by: Dr…………………………………who has answered my questions satisfactorily.	
I agree to my routine blood results and clinical information being collected in the study long term follow up.	
I agree to my viral load being measured every 12-16 weeks	
I agree to completion of adherence questionnaires every 12-16 weeks during the long term follow up and acceptability questionnaires if I re-start continuous therapy or at the end of the study	
I agree to a blood sample being taken at the final visit to be used in future for tests relevant to HIV	
I agree to continue in my allocated group in the study if agreed after discussion with my doctor	
I know that I can withdraw from the long term follow-up at any time without it affecting my care and that I would need to take my anti-HIV medicines as instructed by my doctor	

Name of participant_____________________________

Signature of participant__________________________Date______________

Name of person taking consent_____________________

Signature of person taking consent________________________ Date_____________

Assent Form

BREATHER Long term follow up for young people on SCT

Please tick yes or no to the questions below

1. Have you read (or had read to you) the information about the study follow up?	YES ☐ NO ☐
2. Has somebody explained the study follow up to you?	YES ☐ NO ☐
3. Do you understand what the study follow up is about?	YES ☐ NO ☐
4. Have you asked all the questions you want?	YES ☐ NO ☐
5. Have your questions been answered in a way you understand?	YES ☐ NO ☐
6. Do you understand how the study follow up might help you?	YES ☐ NO ☐
7. Do you understand the risks of taking part?	YES ☐ NO ☐
8. Do you understand it's OK to stop taking part in the study at any time and that if you do not take part you should take your medicines every day/as your doctor tells you?	YES ☐ NO ☐
9. Do you understand that if you take part you will still miss 2 days a week off medicines if your family and doctor agree this is OK?	YES ☐ NO ☐
10. Are you happy to take part?	YES ☐ NO ☐

If any answers are **'no'** or you don't want to take part, **don't sign your name**.

If you **do** want to take part, you can sign your name below.

Your name_________________________ Date _____________

The doctor who explained the project to you needs to sign too:

Doctor's name (print) ____________________ Sign __________________Date________

APPENDIX 19: SAMPLE PATIENT INFORMATION SHEETS FOR LONG TERM FOLLOW UP (FOR YOUNG PEOPLE ON CT)

vi. Parents/carers
vii. Young adults (16 years and over)
viii. Young people (11 to 15 years)
ix. Children (8 to 10 years)
x. Children (unaware of diagnosis) – for use at local care team's discretion

All to be printed on local headed paper, include page numbers and include local requirements

BREATHER
Long term follow up for young people on CT

AN INFORMATION SHEET FOR PARENTS/CARERS

We would like to thank you and your child for taking part in the BREATHER study which is looking at two different ways of giving anti-HIV medicines; either taking the medicines every day (the current standard way of treating HIV), or taking two days a week off treatment. This way of taking anti-HIV medicines is called **Short Cycle Therapy** (SCT).

1. HOW IS THE STUDY PROGRESSING?

Your child is now finishing the main BREATHER study and we thank you both for all your time and for completing the questionnaires and allowing us to take some extra blood samples. As it takes several months to check and combine all the information from everyone in the study, we are not yet able to give any results of the study. However as soon as we have these results, your doctor will discuss them with you. This is likely to be in early 2015.

2. WHAT HAPPENS NOW?

Your child was in the group taking anti-HIV treatment every day (continuous therapy). As there are no results yet, we cannot say if it is OK for your child to take two days a week off treatment. When results from the study are available we will share these with you.

3. WHAT IS LONG TERM FOLLOW UP OF THE BREATHER STUDY, AND WHY IS IT BEING DONE?

As BREATHER is the only study of its kind in children and young people, the Scientific Committees which monitor the study feels it is important to continue to collect further information about differences that might emerge between young people and children who have remained on continuous therapy and those who had two days off treatment. It would therefore be helpful to continue to monitor your child’s health each year for the next two years. The information collected will cover any illnesses s/he may have as well as any changes in his/her HIV drugs and some blood results from your regular clinic visits including viral load. It’s important that your child’s viral load is tested every 12-16 weeks.

As part of this follow up, one 6ml blood sample will be stored at the final visit in two years’ time. The blood will be tested to find out how the HIV virus responded to SCT compared with continuous therapy. Some of these tests may look at resistance to the medicines, but new tests are being developed all the time. We are therefore asking for you to agree that this stored blood sample can be used in future for tests relevant to HIV. These would be decided by a group of experts in PENTA.

We would like you to complete adherence questionnaires every 12-16 weeks for the next two years.

4. PARTICIPATION

Your continued participation in this study is entirely voluntary. If you decide that you do not wish your child to take part that is entirely your right. Your decision will in no way affect your present or future treatment.

You can withdraw your child from the study at any time without giving a reason.
Withdrawal will not affect your child's medical care.
Before you decide, it is important for you to understand why the research is being done and what it will involve. Please take time to read the following information carefully and discuss it with your friends, relatives and your doctor or clinic nurse if you wish. Ask us if there is anything that is not clear or if you would like more information. Take as much time as you want to decide whether or not you wish your child to take part.

5. WHAT ARE THE POSSIBLE BENEFITS AND DISADVANTAGES OF TAKING PART?

Your child will have to attend clinic and have blood taken every 12-16 weeks which may be more often than routine standard of care at your clinic.

We cannot promise that participating will directly help your child. However, all the information we get might help children and young people with HIV in the future.

6. CONFIDENTIALITY

All information collected during the BREATHER study will continue to be anonymised and confidential. Names will not be used, and we will continue to just use your child's BREATHER study number and date of birth for identification. Hospital notes will only be made available to study staff and confidentiality will be maintained at all times. Identification will be by study number only. Should the results of this study be reported or published your child's name will not be used. The study will be conducted in compliance with the UK Data Protection Act, 1998 and the data will be stored for 20 years.

7. WHAT ELSE DO I NEED TO KNOW?

PENTA has made arrangements for compensation should your child come to any harm because of being in the study and not because of any problem with the way the study is being carried out
Your GP will be informed of your child's participation in the study with your permission.
Your hospital doctor is not receiving any payment for carrying out this research.

8. CONTACT

If you have any concerns or other questions about the study or the way it has been carried out, or if your child has an injury or illness during participation in the study, please contact the nurse at the hospital where the study is being carried out:

Name:………………………… …………………………..

Contact details:……………………………………………………………………

Thank you for taking time to consider this. Please ask any questions and let us know if there are things that you do not understand, or would like more information about (contact number above).

BREATHER
Long term follow up for young people on CT

AN INFORMATION SHEET FOR YOUNG ADULTS

We would like to thank you for taking part in the BREATHER study which is looking at two different ways of giving anti-HIV medicines; either taking the medicines every day (the current standard way of treating HIV), or taking two days a week off treatment. This way of taking anti-HIV medicines is called **Short Cycle Therapy** (SCT).

1. HOW IS THE STUDY PROGRESSING?

You are now finishing the main BREATHER study and we thank you for all your time and for completing the questionnaires and allowing us to take some extra blood samples. As it takes several months to check and combine all the information from everyone in the study, we are not yet able to give any results of the study. However as soon as we have these results, your doctor will discuss them with you. This is likely to be in early 2015.

2. WHAT HAPPENS NOW?

You were in the group taking anti-HIV treatment every day (continuous therapy). As there are no results yet, we cannot say if it is OK for you to take two days a week off treatment. When results from the study are available we will share these with you.

3. WHAT IS LONG TERM FOLLOW UP OF THE BREATHER STUDY, AND WHY IS IT BEING DONE?

As BREATHER is the only study of its kind in children and young people, the Scientific Committees which monitor the study feels it is important to continue to collect further information about differences that might emerge between young people and children who have remained on continuous therapy and those who had two days off treatment. It would therefore be helpful to continue to monitor your health each year for the next two years. The information collected will cover any illnesses you may have as well as any changes in your HIV drugs and some blood results from your regular clinic visits including viral load. It's important that your viral load is tested every 12-16 weeks.

As part of this follow up, one 6ml blood sample will be stored at the final visit in 2 years' time. The blood will be tested to find out how the HIV virus responded to SCT compared with continuous therapy. Some of these tests may look at resistance to the medicines, but new tests are being developed all the time. We are therefore asking for you to agree that this stored blood sample can be used in future for tests relevant to HIV. These would be decided by a group of experts in PENTA.

We would like you to complete adherence questionnaires every 12-16 weeks for the next two years.

4. PARTICIPATION

Your continued participation in this study is entirely voluntary. If you decide that you do not wish to take part that is entirely your right. Your decision will in no way affect your present or future treatment.

You can withdraw from the study at any time without giving a reason. Withdrawal will not affect your medical care.
Before you decide, it is important for you to understand why the research is being done and what it will involve. Please take time to read this information carefully and discuss it with your friends, relatives and your doctor or clinic nurse if you wish. Ask us if there is anything that is not clear or if you would like more information. Take as much time as you want to decide whether or not you wish to take part.

5. WHAT ARE THE POSSIBLE BENEFITS AND DISADVANTAGES OF TAKING PART?

You will have to attend clinic and have blood taken every 12-16 weeks which may be more often than routine standard of care at your clinic.

We cannot promise that participating will directly help you. However, all the information we get might help young people with HIV in the future.

6. CONFIDENTIALITY

All information collected during the BREATHER study will continue to be anonymised and confidential. Names will not be used, and we will continue to just use your BREATHER study number and date of birth for identification. Hospital notes will only be made available to study staff and confidentiality will be maintained at all times. Identification will be by study number only. Should the results of this study be reported or published your name will not be used. The study will be conducted in compliance with the UK Data Protection Act, 1998 and the data will be stored for 20 years.

5. WHAT ELSE DO I NEED TO KNOW?

PENTA has made arrangements for compensation should you come to any harm because of being in the study and not because of any problem with the way the study is being carried out
Your GP will be informed of your participation in the study with your and your parent/carers permission.
Your hospital doctor is not receiving any payment for carrying out this research.

6. CONTACT

If you have any concerns or other questions about the study or the way it has been carried out, or if you have an injury or illness during your participation, contact the nurse at the hospital where the study is being carried out:

Name:…………………………. ……………………………..

Contact details:…………………………………………………………………..

Thank you for taking time to consider this. Please ask any questions and let us know if there are things that you do not understand, or would like more information about (contact number above).

BREATHER
Long term follow up for young people on CT

AN INFORMATION SHEET FOR YOUNG PEOPLE

We would like to thank you for taking part in the BREATHER study which is looking at two different ways of giving anti-HIV medicines; either taking the medicines every day (the current standard way of treating HIV), or taking two days a week off treatment. This way of taking anti-HIV medicines is called **Short Cycle Therapy** (SCT).

1. HOW IS THE STUDY PROGRESSING?

You are now finishing the main BREATHER study and we thank you for all your time and for completing the questionnaires and allowing us to take some extra blood samples. As it takes several months to check and sort all the information from everyone in the study, we are not yet able to give any results of the study. However as soon as we have these results, your doctor will discuss them with you and your family. This is likely to be in early 2015.

2. WHAT HAPPENS NOW?

You were in the group taking anti-HIV treatment every day. As there are no results from the study yet, we cannot say it is OK to take two days off treatment. When results from the study are available we will let you know. This is likely to be in early 2015.

3. WHAT IS THE LONG TERM FOLLOW-UP OF THE BREATHER STUDY?

As BREATHER is the only study of its kind in children and young people, we would like to continue to collect further information about your health each year for the next two years. This information will cover any illnesses you may have as well as any changes in your HIV drugs and blood results from your normal clinic visits including viral load. It's important that your viral load is tested every 3-4 months.

4. WHAT INFORMATION WILL BE COLLECTED?

If you agree, your doctor will send information from your clinic visits):

- Any illnesses you have
- Your anti-HIV drugs
- Blood test results

Clinic visits will be every 3-4 months so that the level of virus in your blood can be checked.

One blood sample will be stored at the final visit in the study in two years' time. The blood will be used in future for tests relevant to HIV.

We would also like you to complete adherence questionnaires every 3-4 months for the next two years.

5. DO I HAVE TO TAKE PART?

If at any time you don't want to do the research anymore, just tell your parents, doctor or nurse. Your treatment at the clinic will not change.

6. WILL MY MEDICAL DETAILS BE KEPT PRIVATE?

All information collected about you during this study will still be confidential and anonymous. This means information about you will be known by a number only. Your name will not be used.

Thank you for reading this. Please ask any questions and let us know if there are things that you do not understand, or would like more information about.

BREATHER
Long term follow up for young people on CT

AN INFORMATION SHEET FOR CHILDREN

Thank you for taking part in the BREATHER study. The study is looking at two different ways of giving anti-HIV medicines; either taking the medicines every day (the current standard way of treating HIV), or taking two days a week off. This way of taking anti-HIV medicines is called **Short Cycle Therapy** (SCT).

1. HOW IS THE STUDY PROGRESSING?

You are now finishing the main BREATHER study and we thank you for all your time and coming to the visits. It will take several months to check and sort all the information collected during the study. Your doctor will talk to you and your family about the results once they are known.

2. WHAT HAPPENS NOW?

You were in the group taking anti-HIV medicines every day. As we do not have results from the study yet we cannot say it is OK to take two days off medicines. When we have results from the study we will let you know.

3. WHAT IS THE LONG TERM FOLLOW-UP OF THE BREATHER STUDY?

We would like to continue to collect further information about your health each year for the next two years. The information we get will help us understand more about how HIV affects your life. It's important that your viral load is tested every 3-4 months.

4. WHAT INFORMATION WILL BE COLLECTED?

If you agree, your doctor will send information from your clinic visits:
- Any illnesses you have
- Your anti-HIV drugs
- Blood test results

Clinic visits will be every 3-4 months so that the level of virus in your blood can be checked.

One blood sample will be stored at the final visit in the study in two years' time.

You will need to complete an adherence questionnaire every 3-4 months over the next two years.

1. DO I HAVE TO TAKE PART?

If at any time you don't want to do the research anymore, just tell your parents, doctor or nurse. Your treatment at the clinic will not change.

2. WILL MY MEDICAL DETAILS BE KEPT PRIVATE?

All information collected about you during this study will still be confidential and anonymous. This means information about you will be known by a number only. Your name will not be used.

Thank you for reading this. Please ask any questions and let us know if there are things that you do not understand, or would like more information about (your doctor or nurse has our contact number).

BREATHER
Long term follow up for young people on CT

AN INFORMATION SHEET FOR CHILDREN TAKING MEDICINES LONG TERM

Thank you for taking part in the BREATHER study. The study is looking at two different ways of giving medicines to children like you; either taking the medicines every day or taking two days a week off. This way of taking medicines is called **Short Cycle Therapy** (SCT).

1. HOW IS THE STUDY PROGRESSING?

You are now finishing the main BREATHER study and we thank you for all your time and coming to the visits. It will take several months to check and sort all the information collected during the study. Your doctor will talk to you and your family about the results once they are known.

2. WHAT HAPPENS NOW?

You were in the group taking medicines every day. When we have results from the study we will let you know whether or not it is OK to take two days a week off medicines.

3. WHAT IS THE LONG TERM FOLLOW-UP OF THE BREATHER STUDY?

We would like to continue to collect further information about your health each year for the next two years. The information we get will help us understand more about how we can help children like you. It's important that you come to clinic every 3-4 months so that we can check you are well.

4. WHAT INFORMATION WILL BE COLLECTED?

If you agree, your doctor will send information from your clinic visits:

- Any illnesses you have
- Your medicines
- Blood test results

Clinic visits will be every 3-4 months so that some blood can be taken to check you are still well.

One blood sample will be stored at the final visit in the study in two years' time.

We would like you to complete an adherence questionnaire every 3-4 months over the next two years.

5. DO I HAVE TO TAKE PART?

If at any time you don't want to do the research anymore, just tell your parents, doctor or nurse. Your treatment at the clinic will not change.
If you don't take part, you should take your medicines the way that your doctor tells you to.

6. WILL MY MEDICAL DETAILS BE KEPT PRIVATE?

All information collected about you during this study will still be confidential and anonymous. This means information about you will be known by a number only. Your name will not be used.

Thank you for reading this. Please ask any questions and let us know if there are things that you do not understand, or would like more information about.

APPENDIX 20: SAMPLE CONSENT FORMS FOR LONG TERM FOLLOW UP (FOR YOUNG PEOPLE ON CT)

xi. Parents/carers
xii. Young adults (16 years and over)
xiii. Assent form for young people

All to be printed on local headed paper, include page numbers and include local requirements

Consent Form for parents/carers

BREATHER Long term follow up for young people on CT

Please write your initials in each box if you agree:

I have read and understood the information sheet on the BREATHER study long term follow up for CT arm, version…, dated……..	
I understand the potential benefits and disadvantages of my child participating in the study long term follow up.	
The details of the study long term follow up have been explained by: Dr………………………………who has answered my questions satisfactorily.	
I agree to my child's routine blood results and clinical information being collected for long term follow up	
I agree to my child's viral load being measured every 12-16 weeks	
I agree to completion of adherence questionnaires every 12-16 weeks during the long term follow up	
I agree to a blood sample being taken at the final visit to be used in future for tests relevant to HIV	
I know that my child can be withdrawn from the study follow-up and assessment at any time without it affecting his/her care.	

Name of child/young person: ____

Name of Parent/Guardian: ____

Signature of Parent/Guardian:_____________________________Date:______

Name of clinician:____

Signature of clinician:_____________________________________Date:_______

CONSENT FORM FOR YOUNG ADULTS

BREATHER Long term follow up for young people on CT

Please write your initials in each box if you agree:

I have read and understood the information sheet on the BREATHER study long term follow up for CT arm, version…, dated……..	
I understand the potential benefits and disadvantages of participating in the study long term follow up.	
The details of this study have been explained by: Dr…………………………………who has answered my questions satisfactorily.	
I agree to my routine blood results and clinical information being collected in the study long term follow up.	
I agree to my viral load being measured every 12-16 weeks	
I agree to completion of adherence questionnaires every 12-16 weeks during the long term follow up	
I agree to a blood sample being taken at the final visit to be used in future for tests relevant to HIV	
I know that I can withdraw from the study follow-up and assessment at any time without it affecting my care.	

Name of participant_____________________________

Signature of participant__________________________Date_______________

Name of person taking consent______________________

Signature of person taking consent________________________ Date______________

Assent Form

BREATHER Long term follow up for young people on CT

Please tick yes or no to the questions below

1. Have you read (or had read to you) the information about the study follow up?	YES ☐<br>NO ☐
2. Has somebody explained the study follow up to you?	YES ☐<br>NO ☐
3. Do you understand what the study follow up is about?	YES ☐<br>NO ☐
4. Have you asked all the questions you want?	YES ☐<br>NO ☐
5. Have your questions been answered in a way you understand?	YES ☐<br>NO ☐
6. Do you understand how the study follow up might help you?	YES ☐<br>NO ☐
7. Do you understand the risks of taking part?	YES ☐<br>NO ☐
8. Do you understand it's OK to stop taking part in the study at any time?	YES ☐<br>NO ☐
9. Are you happy to take part?	YES ☐<br>NO ☐

If any answers are **'no'** or you don't want to take part, **don't sign your name**.

If you **do** want to take part, you can sign your name below.

Your name_________________________ Date _____________

The doctor who explained the project to you needs to sign too:

Doctor's name (print) _____________________ Sign ___________________Date________

APPENDIX 21: SAMPLE CONSENT FORMS FOR ADDITIONAL PLASMA (AND CELL STORAGE WHERE AVAILABLE) DURING LONG TERM FOLLOW UP

For participants where consent for long-term follow-up (with blood storage) has already been given

i. Parents/carers
ii. Young adults (16 years and over)
iii. Assent form for young people

All to be printed on local headed paper, include page numbers and include local requirements

CONSENT FORM FOR PARENTS/CARERS

BREATHER Long Term Follow Up – additional blood sample

Thank you for continuing to take part in the BREATHER study.

As you know, the BREATHER study is looking at the effectiveness of HIV medicines in young people. In particular, the body's response to 2 different ways of giving anti-HIV medicines: either taking the medicines every day or taking 2 days a week off.

The study team recently presented some of the results at an international scientific and clinical meeting. It was agreed that this was a very important study and particularly the need to collect further information over a longer period of time. The researchers recommended we try to collect an extra blood sample this summer (2015), in addition to the planned blood sample next summer (2016), to understand better the body's response.

The additional blood sample will not involve any extra visit or needles. The blood will be taken alongside usual clinic bloods as it was in the main part of the study.

Please write your initials in the box and sign this form if you consent to the additional blood sample:

I agree to a linked anonymised blood sample being taken and processed during 2015 and to be stored for ethically approved studies to help understand more about HIV and effective treatment.	

Name of child/young person: ______________________________

Name of Parent/Guardian: ______________________________

Signature of Parent/Guardian:____________________ Date:__________

Name of person taking consent:____________________

Signature of person taking consent:____________________ Date:__________

CONSENT FORM FOR YOUNG ADULTS

BREATHER Long Term Follow Up– additional blood sample

Thank you for continuing to take part in the BREATHER study.

As you know, the BREATHER study is looking at the effectiveness of HIV medicines in young people. In particular, the body's response to 2 different ways of giving anti-HIV medicines: either taking the medicines every day or taking 2 days a week off.

The study team recently presented some of the results at an international scientific and clinical meeting. It was agreed that this was a very important study and particularly the need to collect further information over a longer period of time. The researchers recommended we try to collect an extra blood sample this summer (2015), in addition to the planned blood sample next summer (2016), to understand better the body's response.

The additional blood sample will not involve any extra visit or needles. The blood will be taken alongside usual clinic bloods as it was in the main part of the study.

Please write your initials in the box and sign this form if you consent to the additional blood sample:

I agree to a linked anonymised blood sample being taken and processed during 2015 and to be stored for ethically approved studies to help understand more about HIV and effective treatment	

Name of participant_____________________________

Signature of participant__________________________Date:_________________

Name of person taking consent________________________

Signature of person taking consent________________________ Date:______________

ASSENT FORM FOR YOUNG PEOPLE

BREATHER Long Term Follow Up– additional blood sample

Thank you for continuing to take part in the BREATHER study.

As you know, the BREATHER study is looking at how well medicines work in young people, either taking the medicines every day or taking 2 days a week off.

The study team recently presented some results at an international scientific and clinical meeting. It was agreed that this was a very important study but we need to collect more information over a longer period of time. The researchers recommended we collect an extra blood sample this summer (2015), in addition to the planned blood sample next summer (2016), to better understand the body's response.

The additional blood sample will not involve any extra visit or needles. The blood will be taken alongside usual clinic bloods as it was in the main part of the study.

Please tick "Yes" or "No" to the questions below

1. Do you understand why an additional blood sample is being taken?	YES ☐<br>NO ☐
2. Have you asked all the questions you want?	YES ☐<br>NO ☐
3. Have your questions been answered in a way you understand?	YES ☐<br>NO ☐
4. Are you happy for the additional blood sample to be taken?	YES ☐<br>NO ☐

If any answers are **"No"** or you don't want to give an additional sample, **don't sign your name**.

If you **are happy to give an additional sample**, you can sign your name below.

Your name________________________ Date: _____________

The doctor or nurse who explained this to you needs to sign too:

Doctor/Nurse's name (print) ____________________

Doctor/Nurse's name (Signature):__________________Date:___________
