# Supplementary material for: Weekends-off efavirenz-based antiretroviral therapy in HIV-infected children, adolescents and young adults (BREATHER): Extended follow-up results of a randomised, open-label, non-inferiority trial
Source: PLoS One. 2018 Apr 23;13(4):e0196239. doi: 10.1371/journal.pone.0196239 (PMC5912750; doi:10.1371/journal.pone.0196239)
Supplement: S1 Table — (DOCX) [file pone.0196239.s004.docx]

**Centres participating in the BREATHER trial**

| **Centre name** | **Country** | **Number of participants enrolled** | **Total number of participants enrolled from country** |
| --- | --- | --- | --- |
| Helios Salud, Buenos Aires | Argentina | 7 | 11 |
| Hospital Dr. J.P. Garrahan, Buenos Aires | Argentina | 4 |  |
| St. Pierre University Hospital, Brussels | Belgium | 2 | 2 |
| Hvidovre Hospital | Denmark | 3 | 3 |
| J W Goethe University Frankfurt | Germany | 3 | 3 |
| Our Lady’s Children’s Hospital, Dublin | Ireland | 3 | 3 |
| 12 de Octubre, Madrid | Spain | 5 | 11 |
| La Paz/Carlos III, Madrid | Spain | 2 |  |
| Universitario de Getafe | Spain | 2 |  |
| Hospital La Fe, Valencia | Spain | 1 |  |
| Sant Joan de Déu, Barcelona | Spain | 1 |  |
| HIV-NAT, Bangkok | Thailand | 25 | 36 |
| Kalasin hospital | Thailand | 7 |  |
| Khon Kaen | Thailand | 4 |  |
| Joint Clinical Research Centre (JCRC), Kampala | Uganda | 70 | 70 |
| Kiev City AIDS Centre | Ukraine | 20 | 20 |
| Great Ormond Street Hospital, London | United Kingdom | 10 | 26 |
| Heartlands Hospital, Birmingham | United Kingdom | 4 |  |
| St. George’s Hospital, London | United Kingdom | 3 |  |
| St. Thomas’ Hospital, London | United Kingdom | 3 |  |
| Bristol Royal Hospital for Children | United Kingdom | 2 |  |
| Leicester University Hospital | United Kingdom | 2 |  |
| Queen’s Medical Centre, Nottingham | United Kingdom | 2 |  |
| St. Jude Children’s Research Hospital, Memphis | United States of America | 14 | 14 |
